# Supplementary figures and images for: Mechanism of gene network in the treatment of intracerebral hemorrhage by natural plant drugs in Lutong granules (part 2 of 2)
Source: PLoS One. 2022 Nov 28;17(11):e0274639. doi: 10.1371/journal.pone.0274639 (PMC9704616; doi:10.1371/journal.pone.0274639)

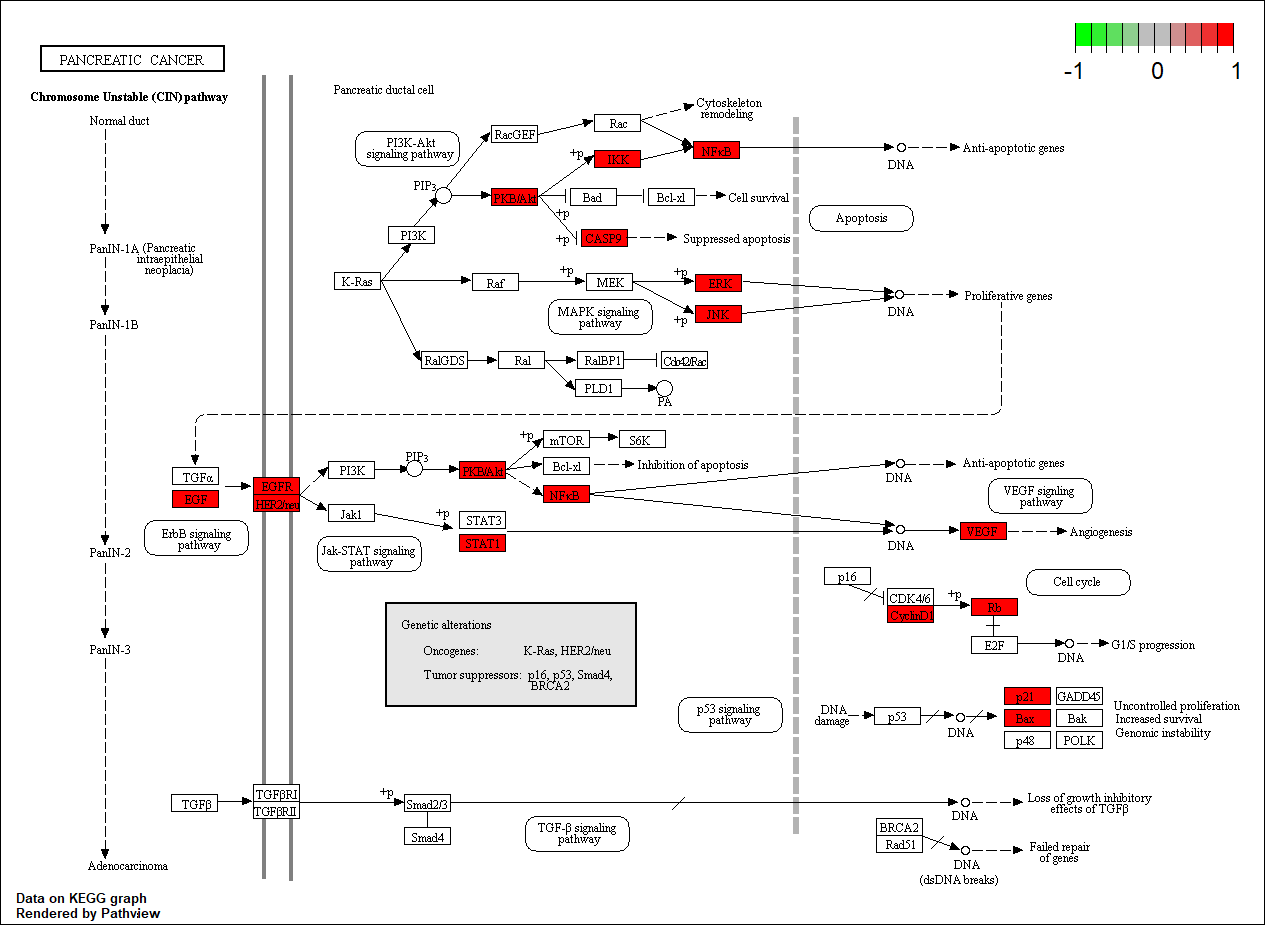

Supplement: S1 Data — (ZIP) [file pone.0274639.s001.zip › minimal data/GO+KEGG/R.KEGG/hsa05212.pathview.png]

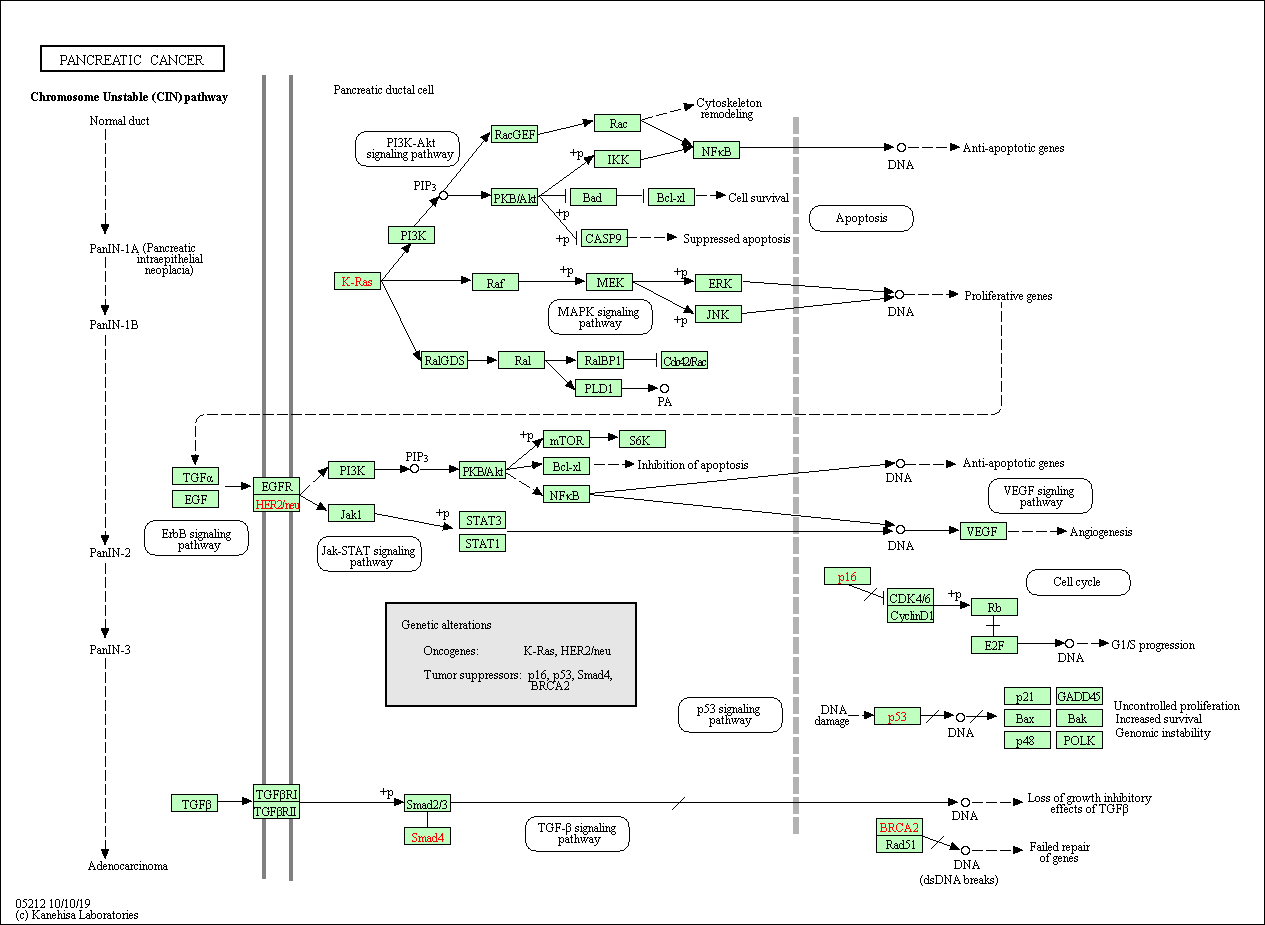

Supplement: S1 Data — (ZIP) [file pone.0274639.s001.zip › minimal data/GO+KEGG/R.KEGG/hsa05212.png]

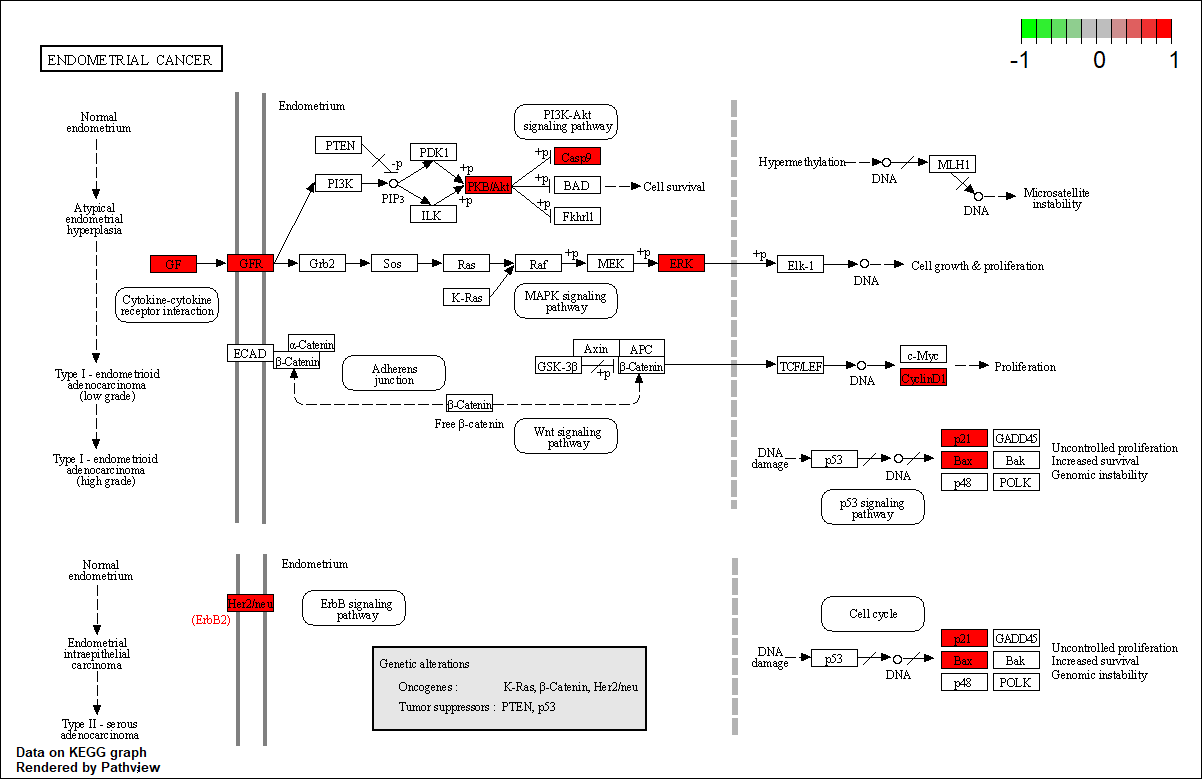

Supplement: S1 Data — (ZIP) [file pone.0274639.s001.zip › minimal data/GO+KEGG/R.KEGG/hsa05213.pathview.png]

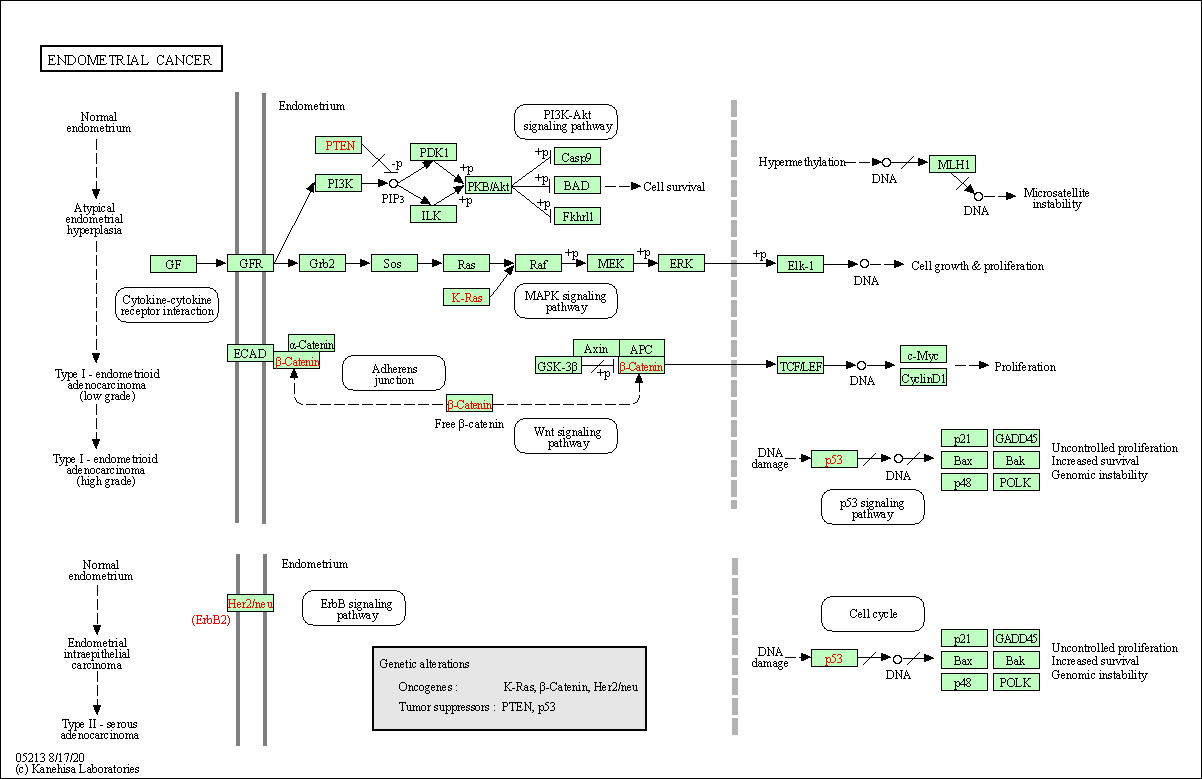

Supplement: S1 Data — (ZIP) [file pone.0274639.s001.zip › minimal data/GO+KEGG/R.KEGG/hsa05213.png]

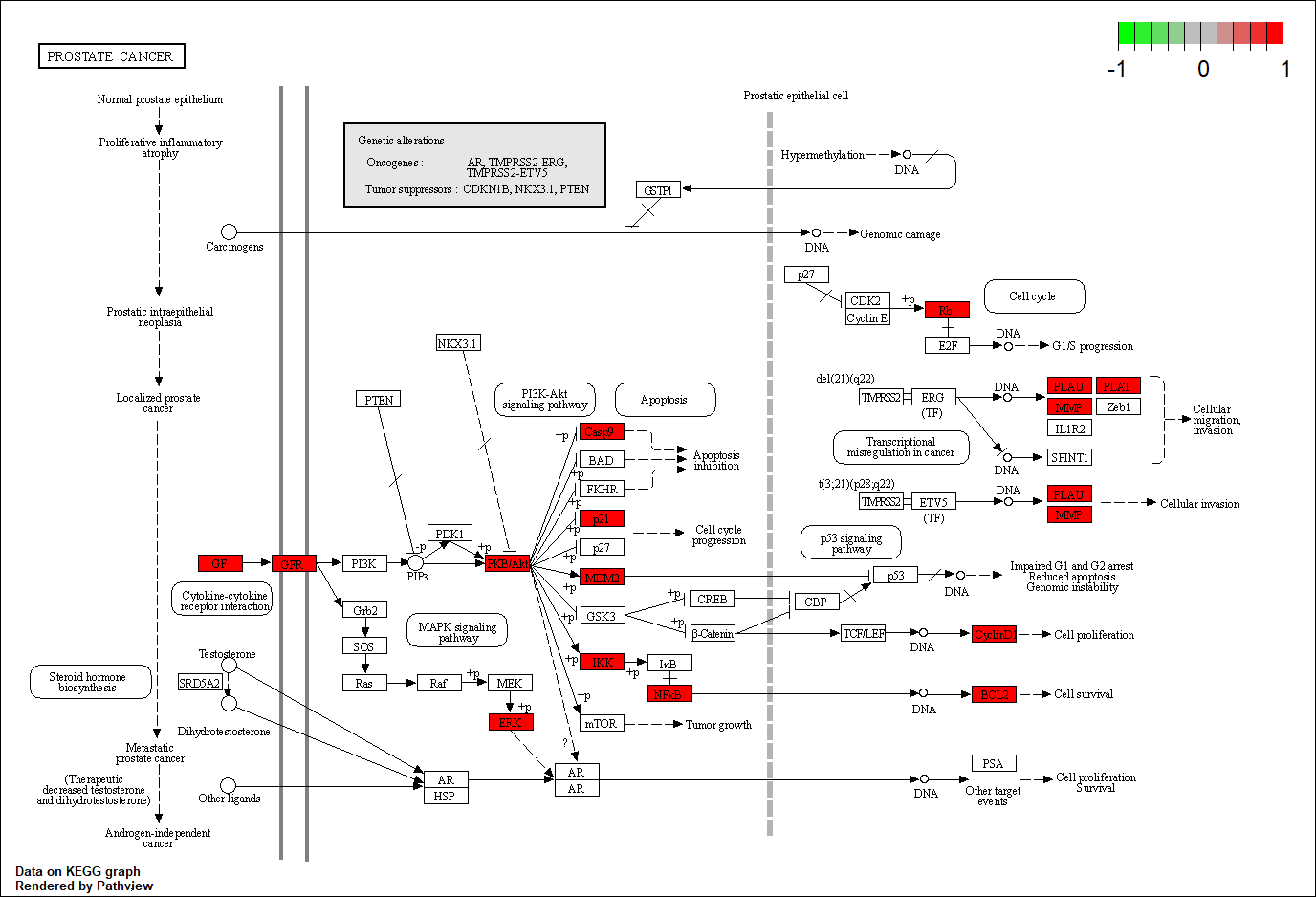

Supplement: S1 Data — (ZIP) [file pone.0274639.s001.zip › minimal data/GO+KEGG/R.KEGG/hsa05215.pathview.png]

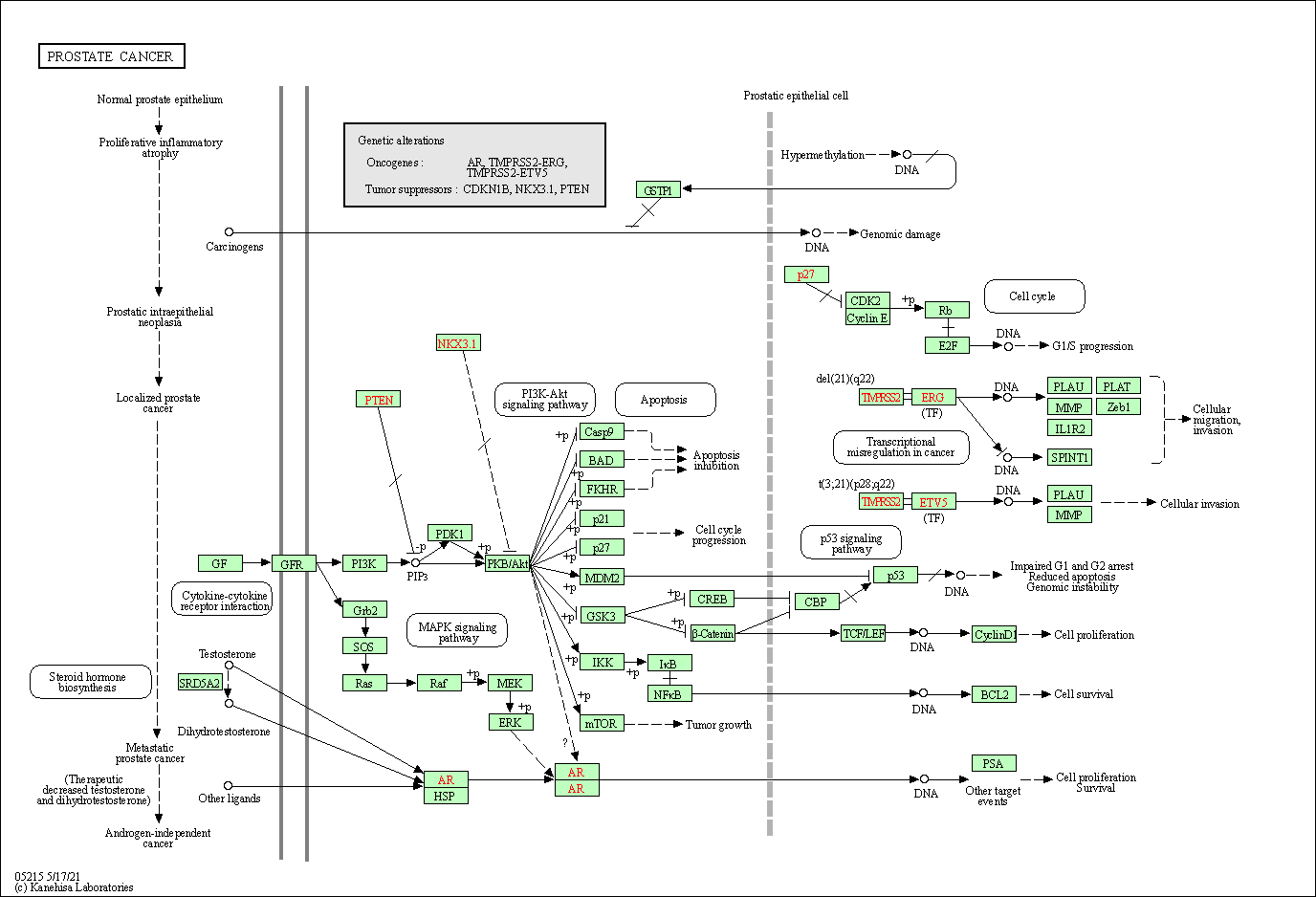

Supplement: S1 Data — (ZIP) [file pone.0274639.s001.zip › minimal data/GO+KEGG/R.KEGG/hsa05215.png]

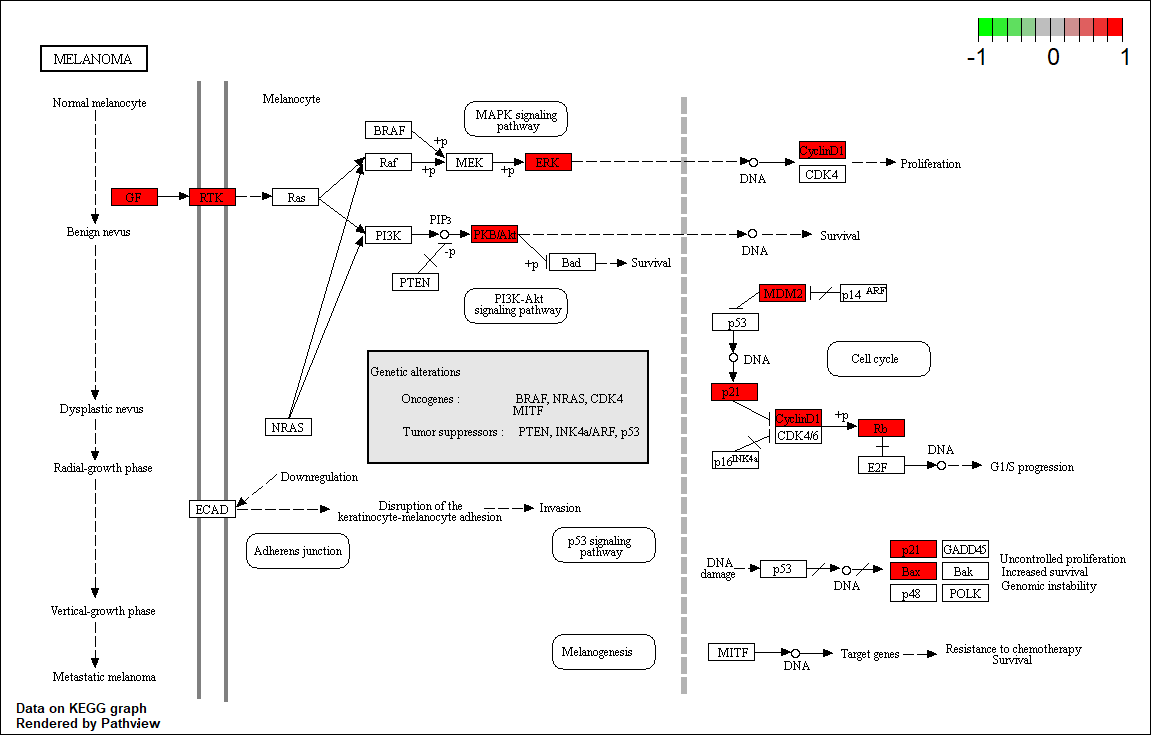

Supplement: S1 Data — (ZIP) [file pone.0274639.s001.zip › minimal data/GO+KEGG/R.KEGG/hsa05218.pathview.png]

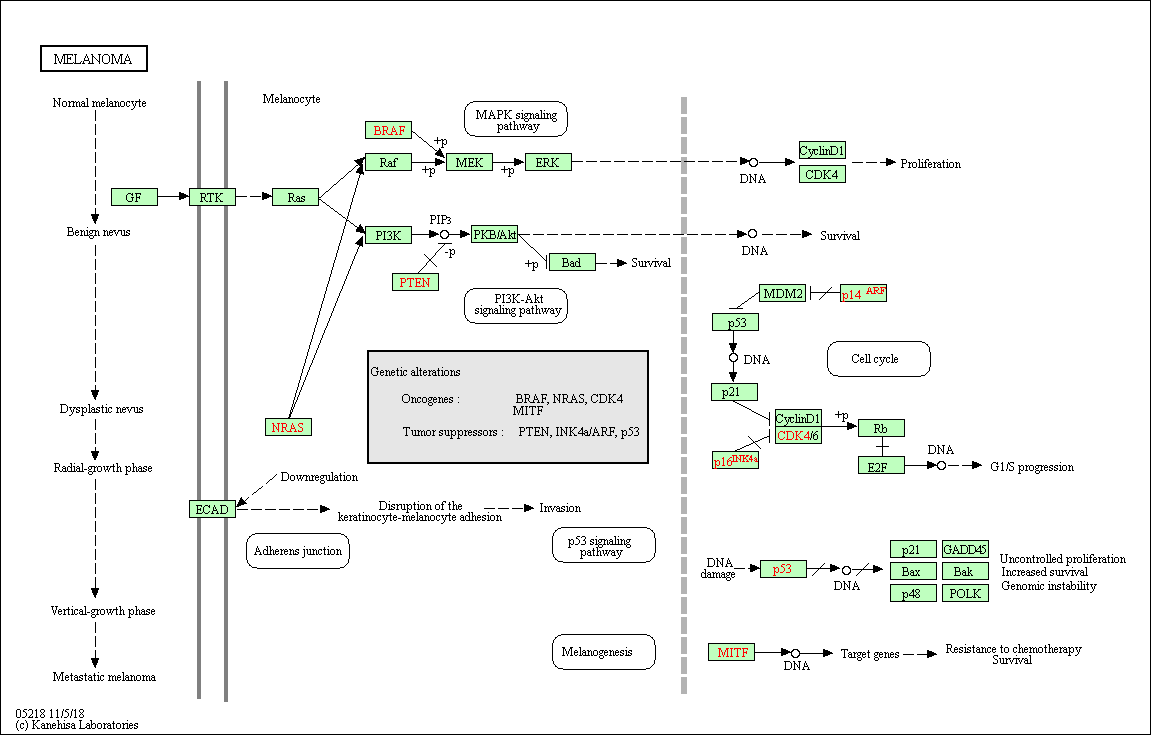

Supplement: S1 Data — (ZIP) [file pone.0274639.s001.zip › minimal data/GO+KEGG/R.KEGG/hsa05218.png]

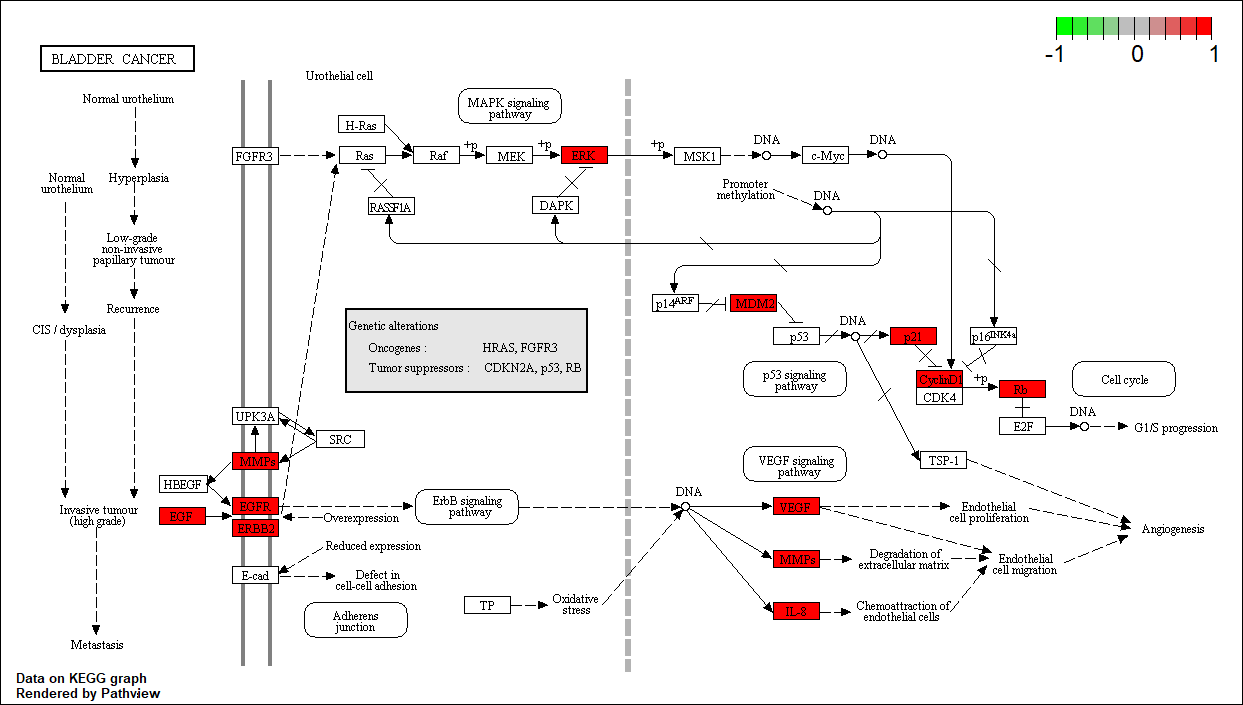

Supplement: S1 Data — (ZIP) [file pone.0274639.s001.zip › minimal data/GO+KEGG/R.KEGG/hsa05219.pathview.png]

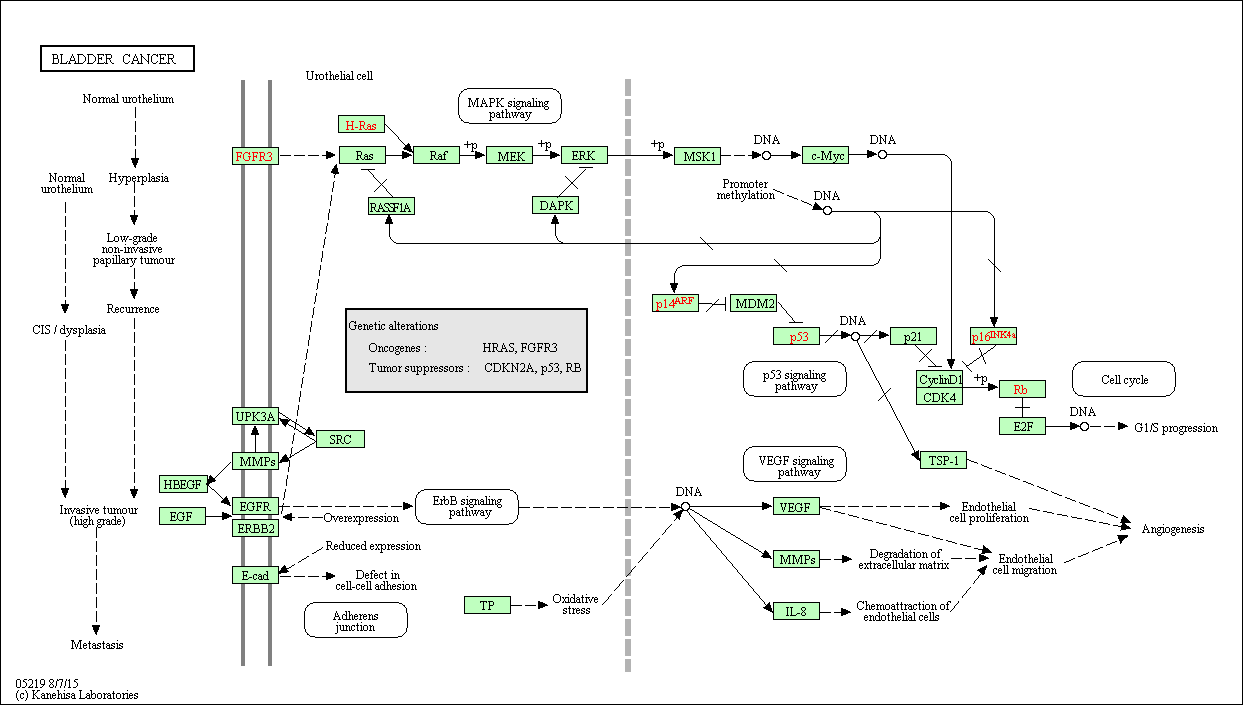

Supplement: S1 Data — (ZIP) [file pone.0274639.s001.zip › minimal data/GO+KEGG/R.KEGG/hsa05219.png]

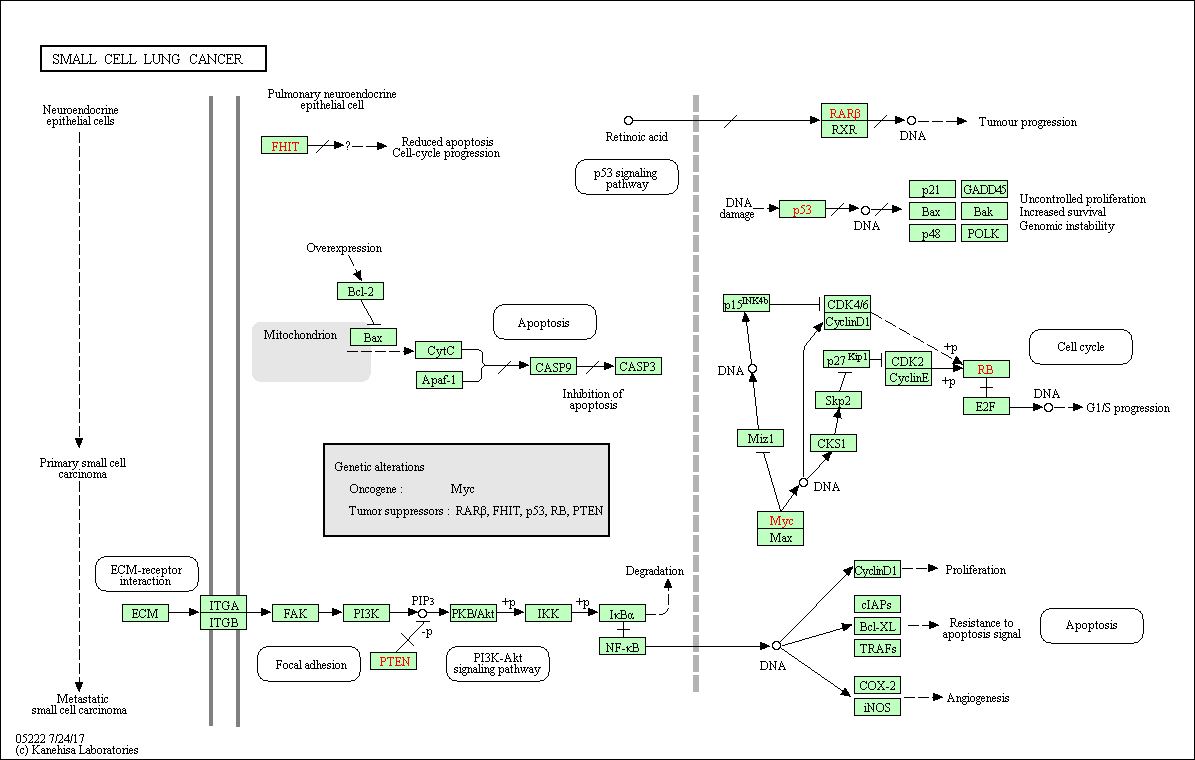

Supplement: S1 Data — (ZIP) [file pone.0274639.s001.zip › minimal data/GO+KEGG/R.KEGG/hsa05222.png]

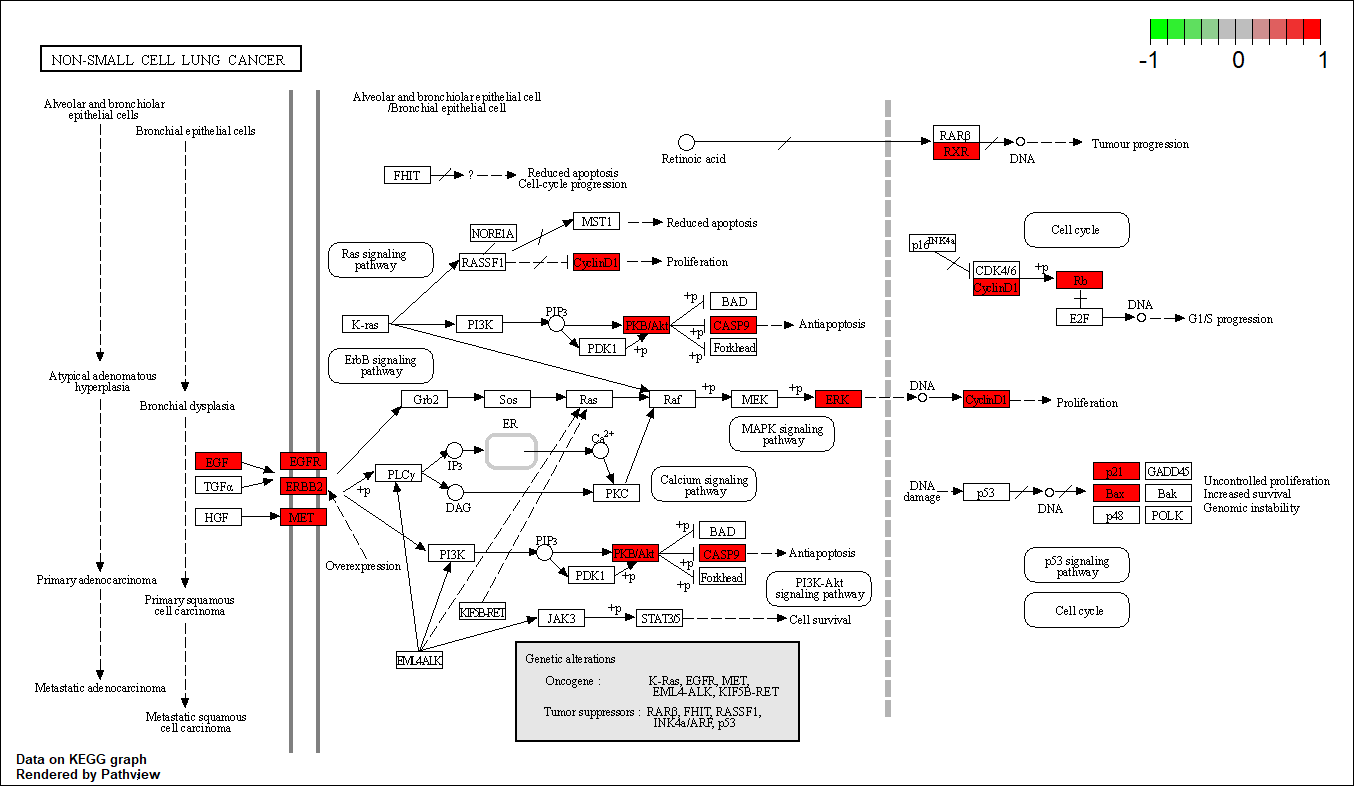

Supplement: S1 Data — (ZIP) [file pone.0274639.s001.zip › minimal data/GO+KEGG/R.KEGG/hsa05223.pathview.png]

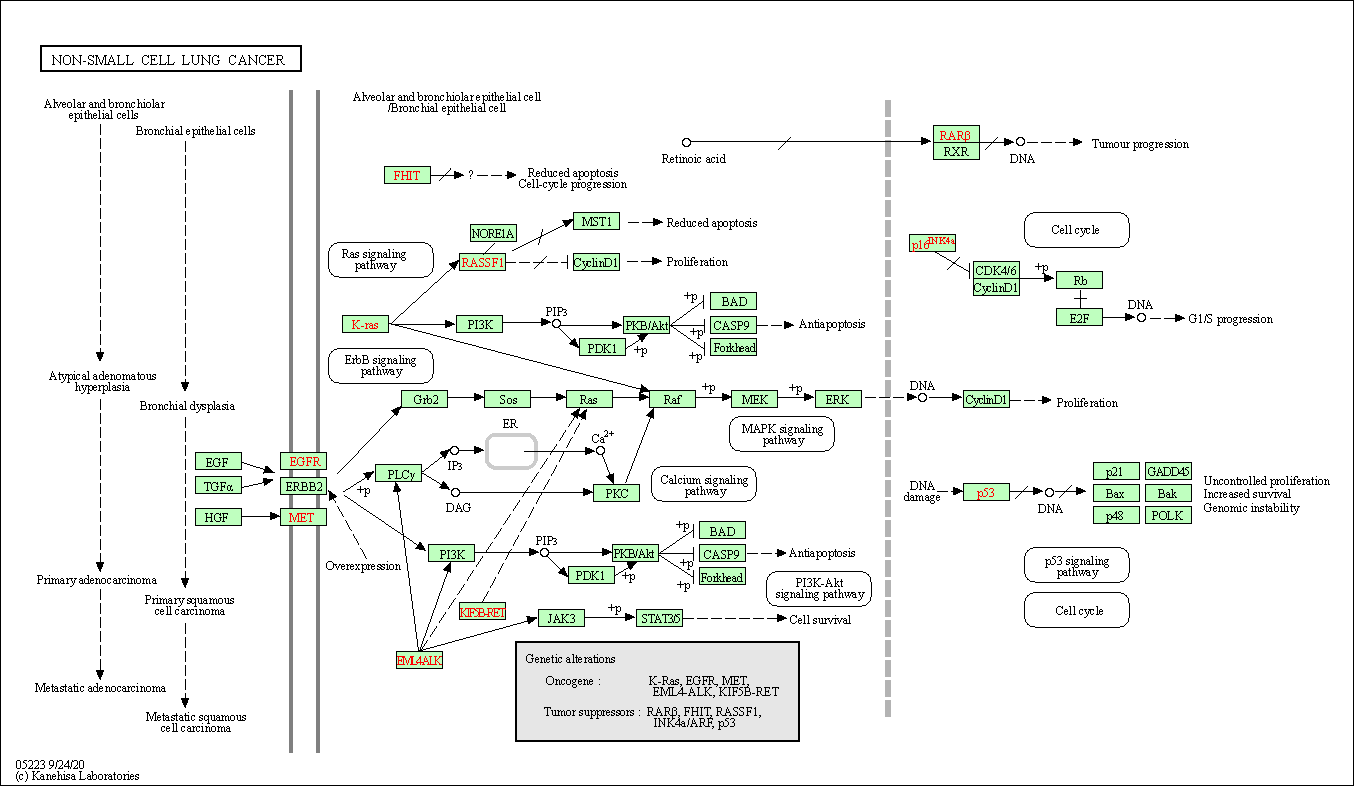

Supplement: S1 Data — (ZIP) [file pone.0274639.s001.zip › minimal data/GO+KEGG/R.KEGG/hsa05223.png]

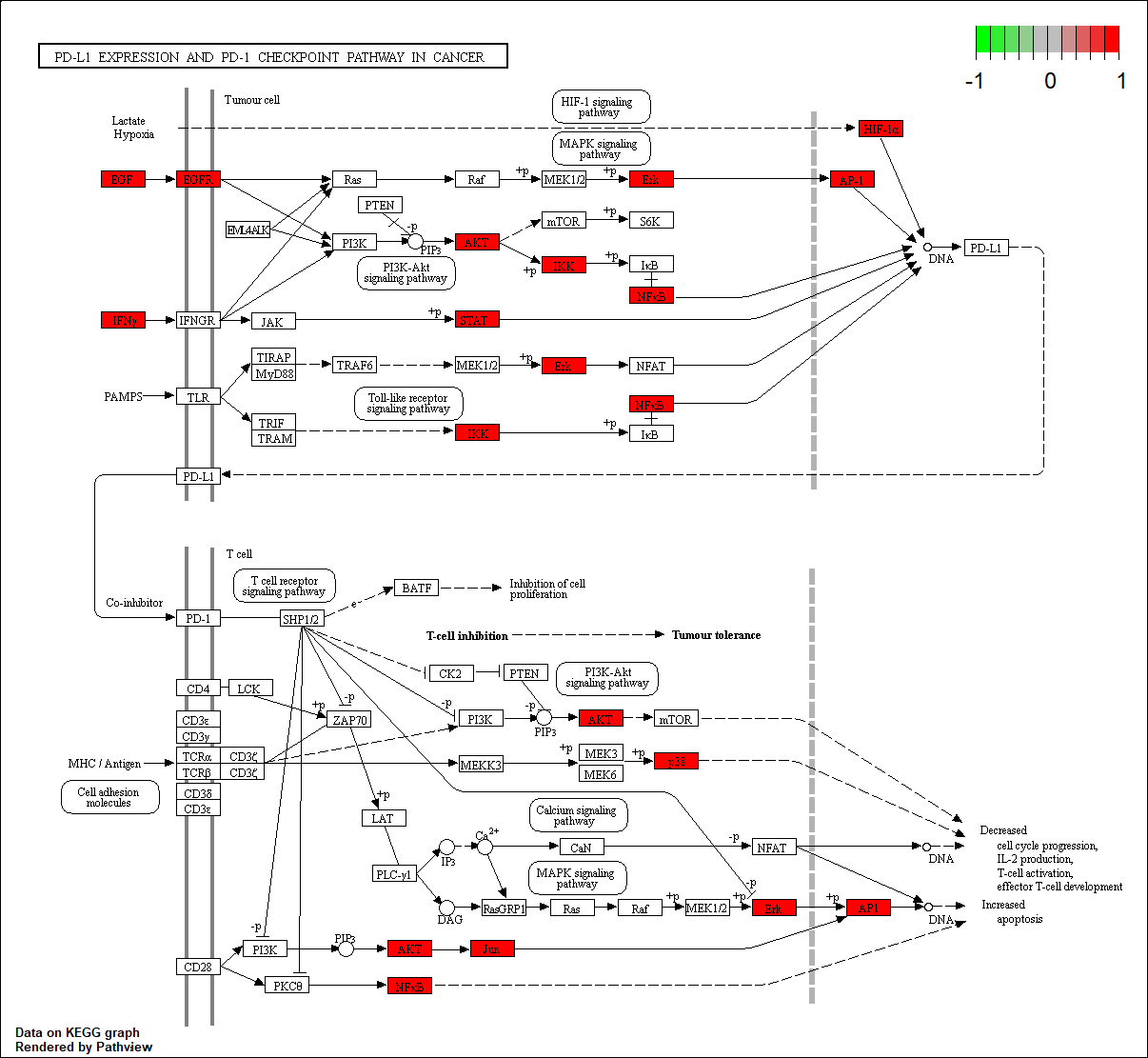

Supplement: S1 Data — (ZIP) [file pone.0274639.s001.zip › minimal data/GO+KEGG/R.KEGG/hsa05235.pathview.png]

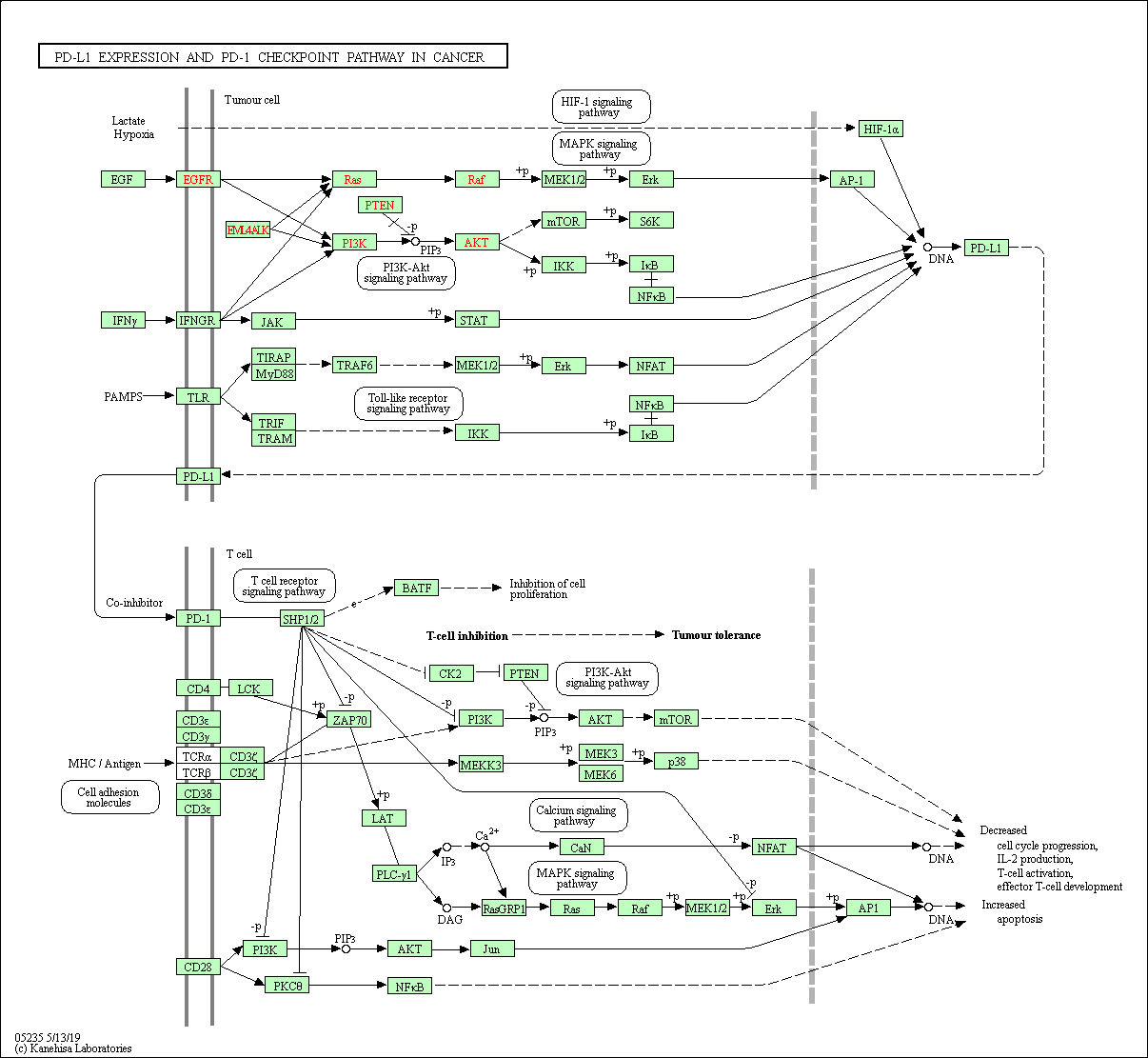

Supplement: S1 Data — (ZIP) [file pone.0274639.s001.zip › minimal data/GO+KEGG/R.KEGG/hsa05235.png]

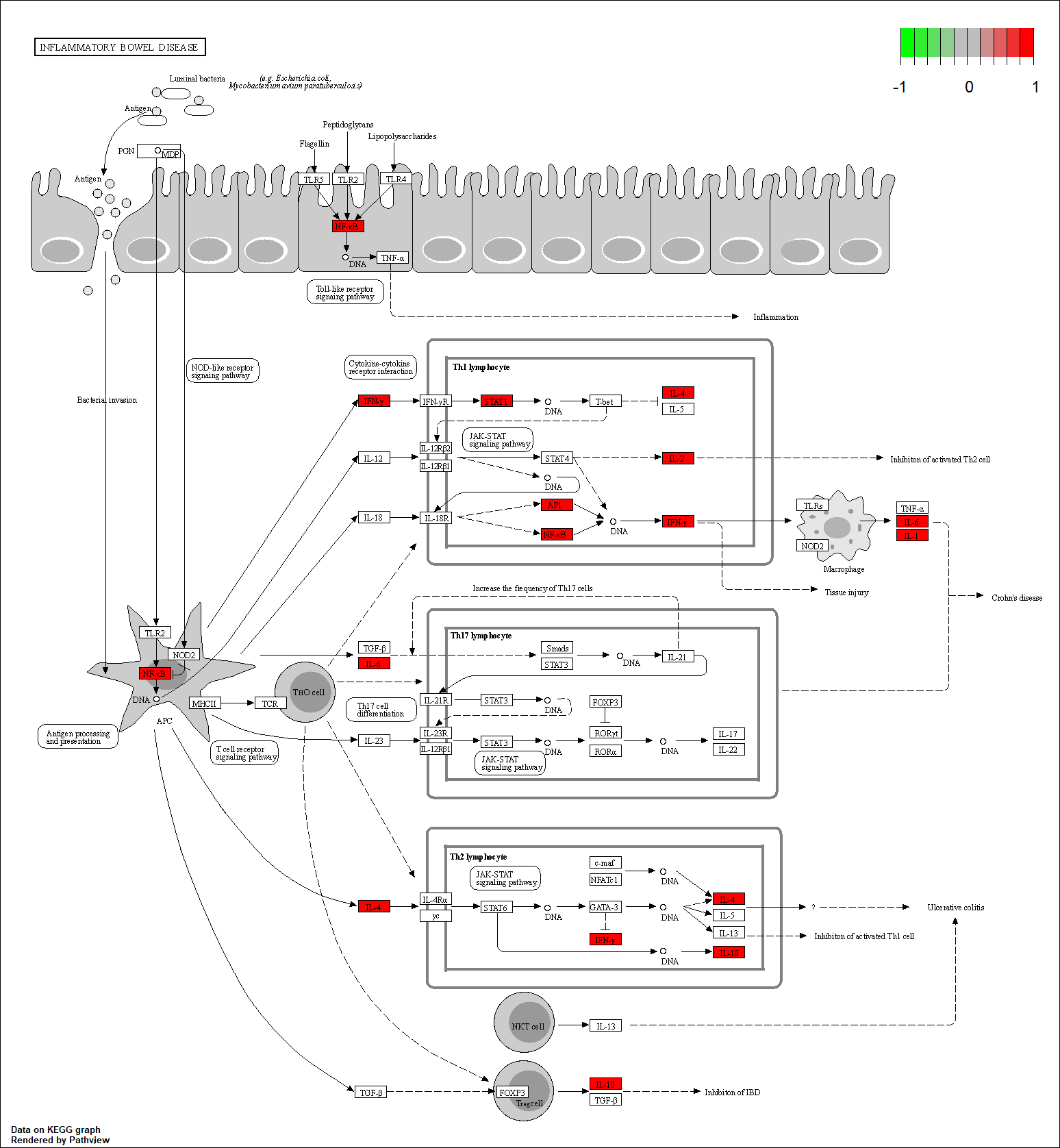

Supplement: S1 Data — (ZIP) [file pone.0274639.s001.zip › minimal data/GO+KEGG/R.KEGG/hsa05321.pathview.png]

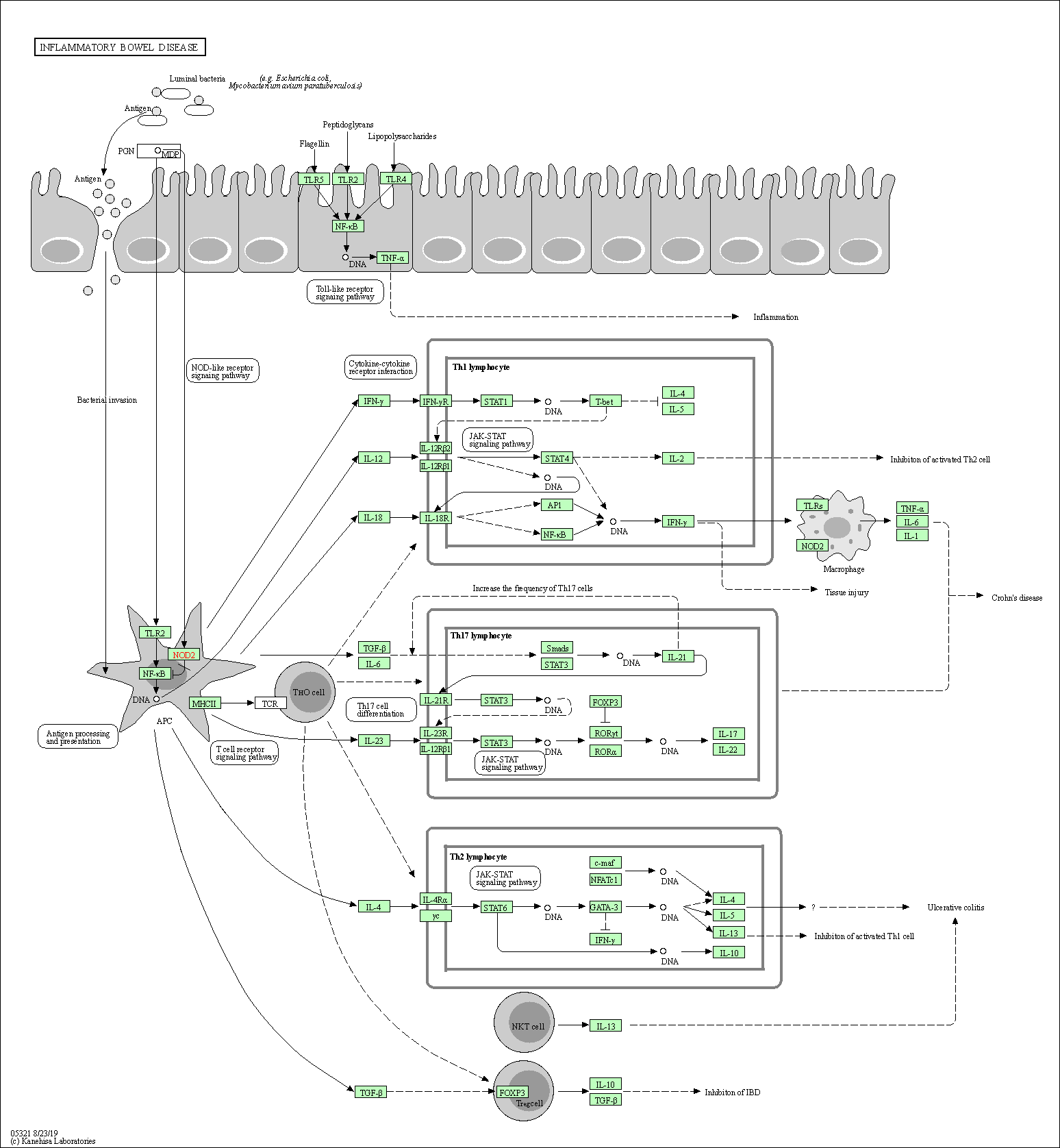

Supplement: S1 Data — (ZIP) [file pone.0274639.s001.zip › minimal data/GO+KEGG/R.KEGG/hsa05321.png]

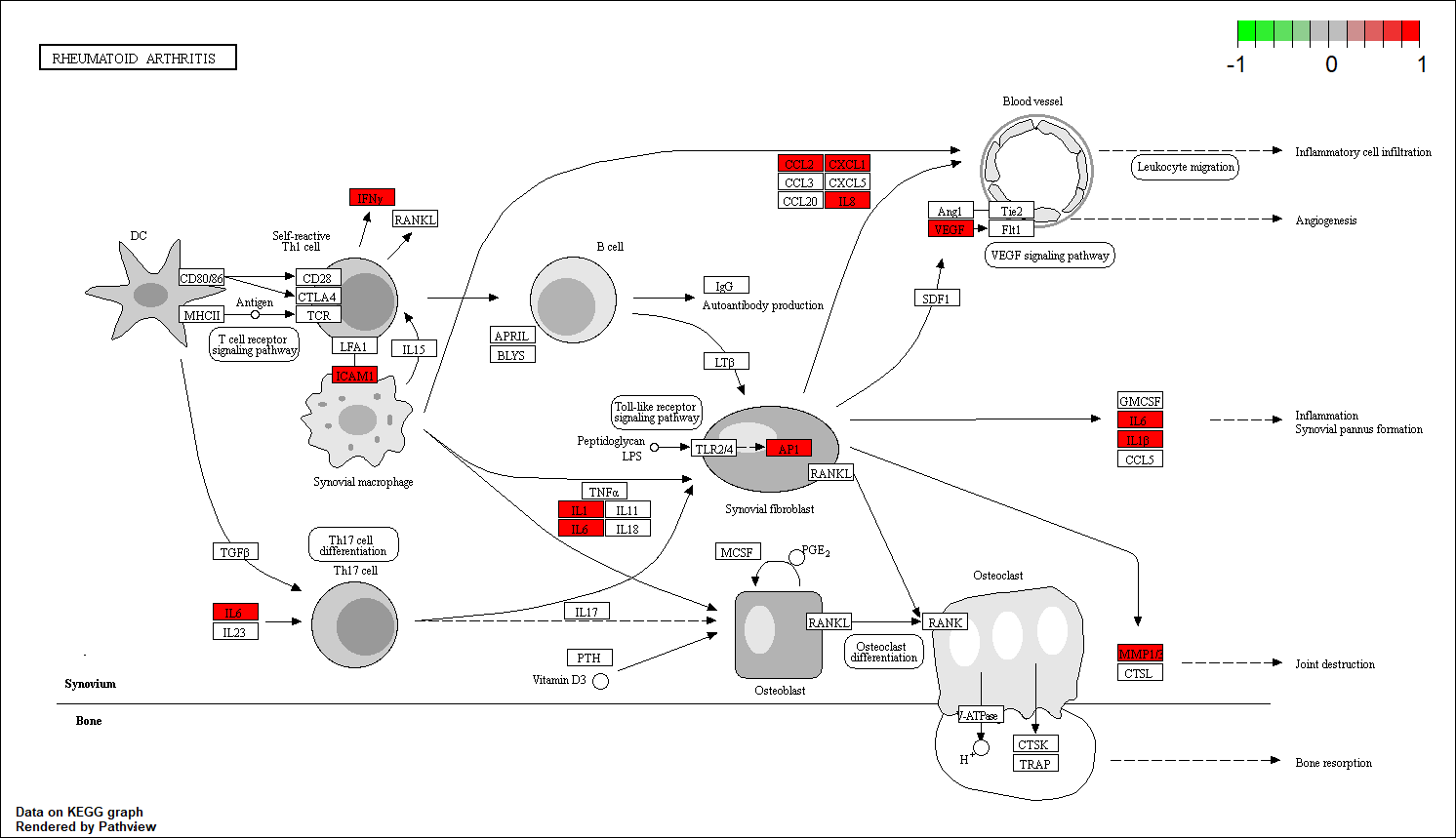

Supplement: S1 Data — (ZIP) [file pone.0274639.s001.zip › minimal data/GO+KEGG/R.KEGG/hsa05323.pathview.png]

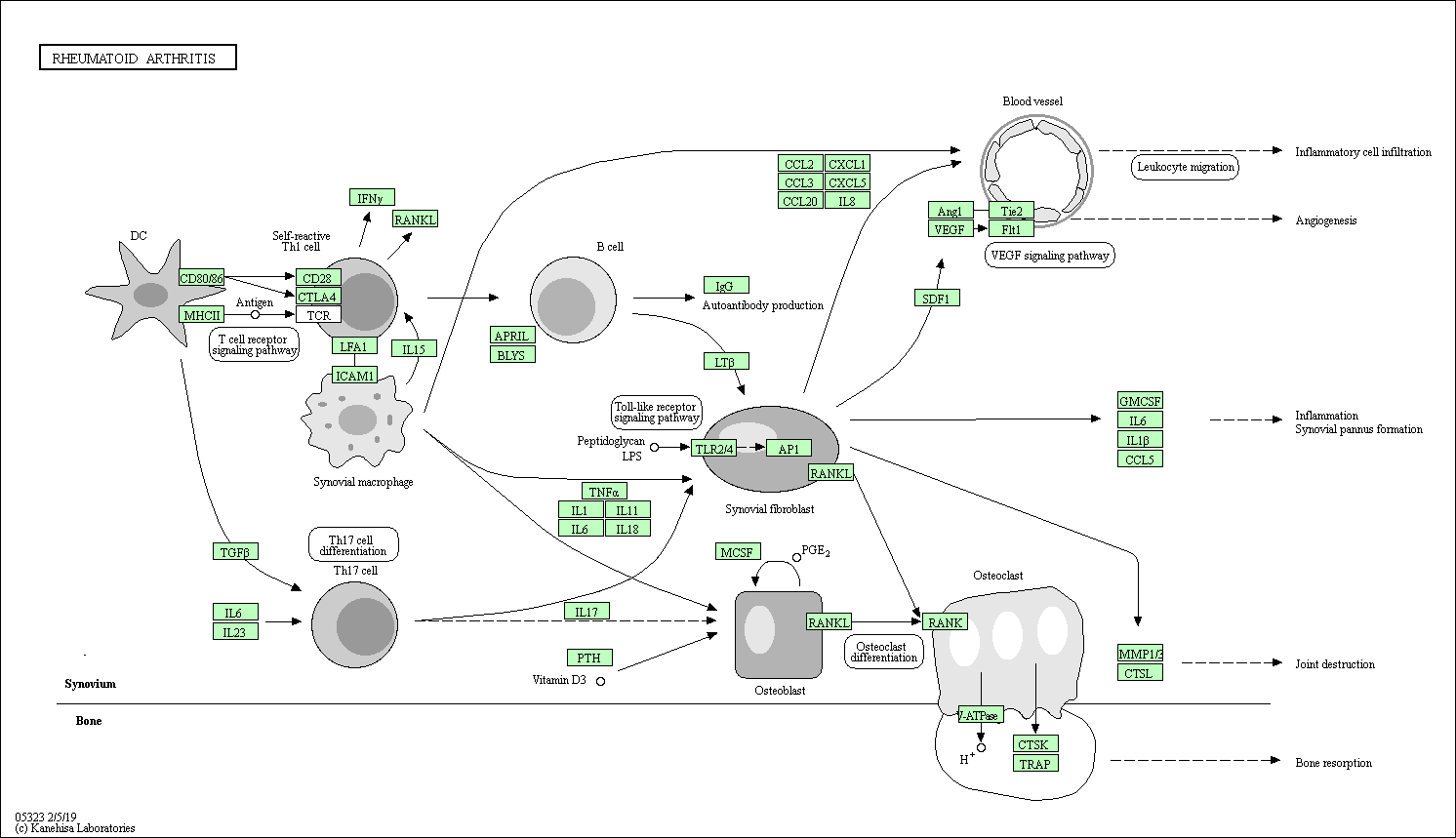

Supplement: S1 Data — (ZIP) [file pone.0274639.s001.zip › minimal data/GO+KEGG/R.KEGG/hsa05323.png]

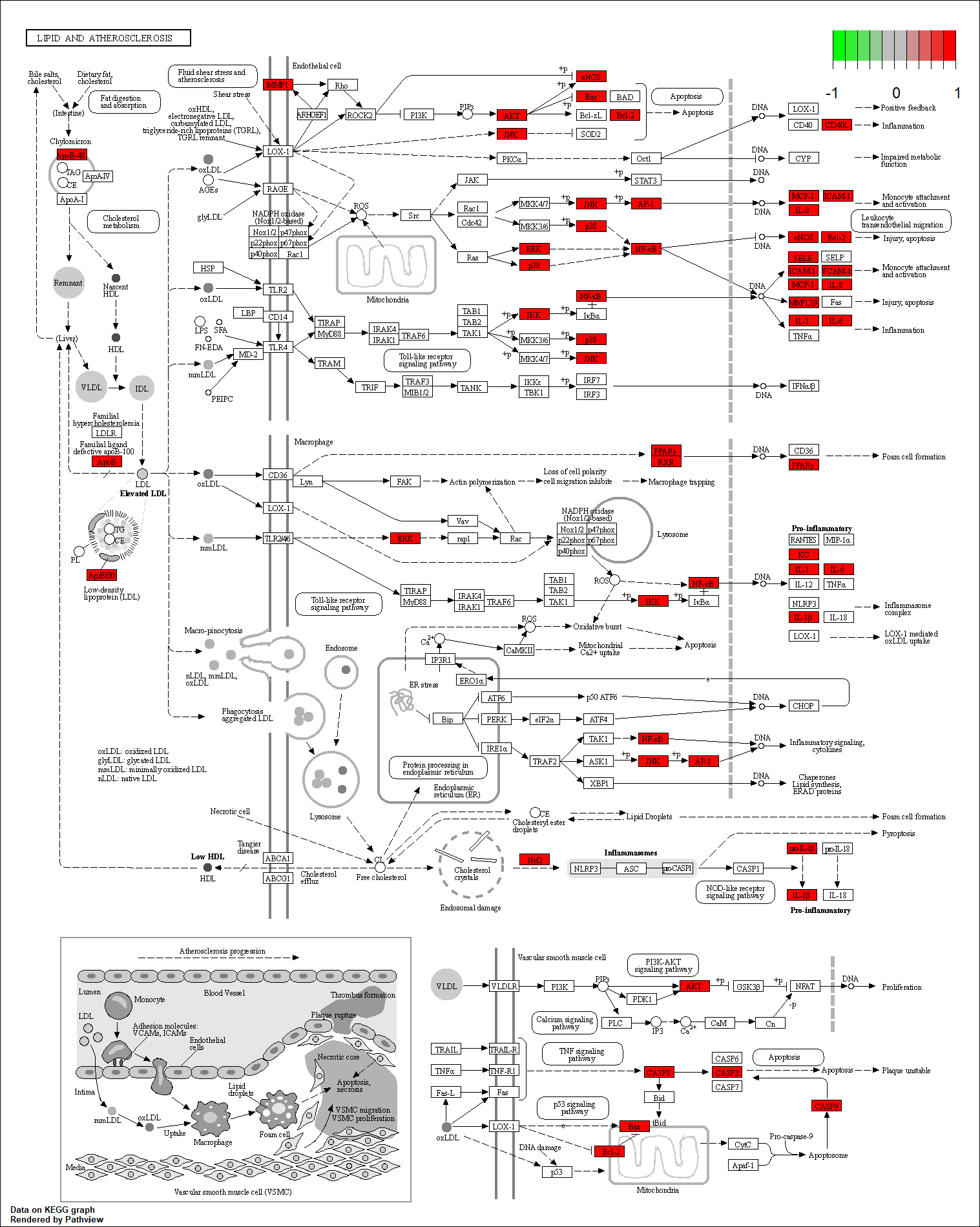

Supplement: S1 Data — (ZIP) [file pone.0274639.s001.zip › minimal data/GO+KEGG/R.KEGG/hsa05417.pathview.png]

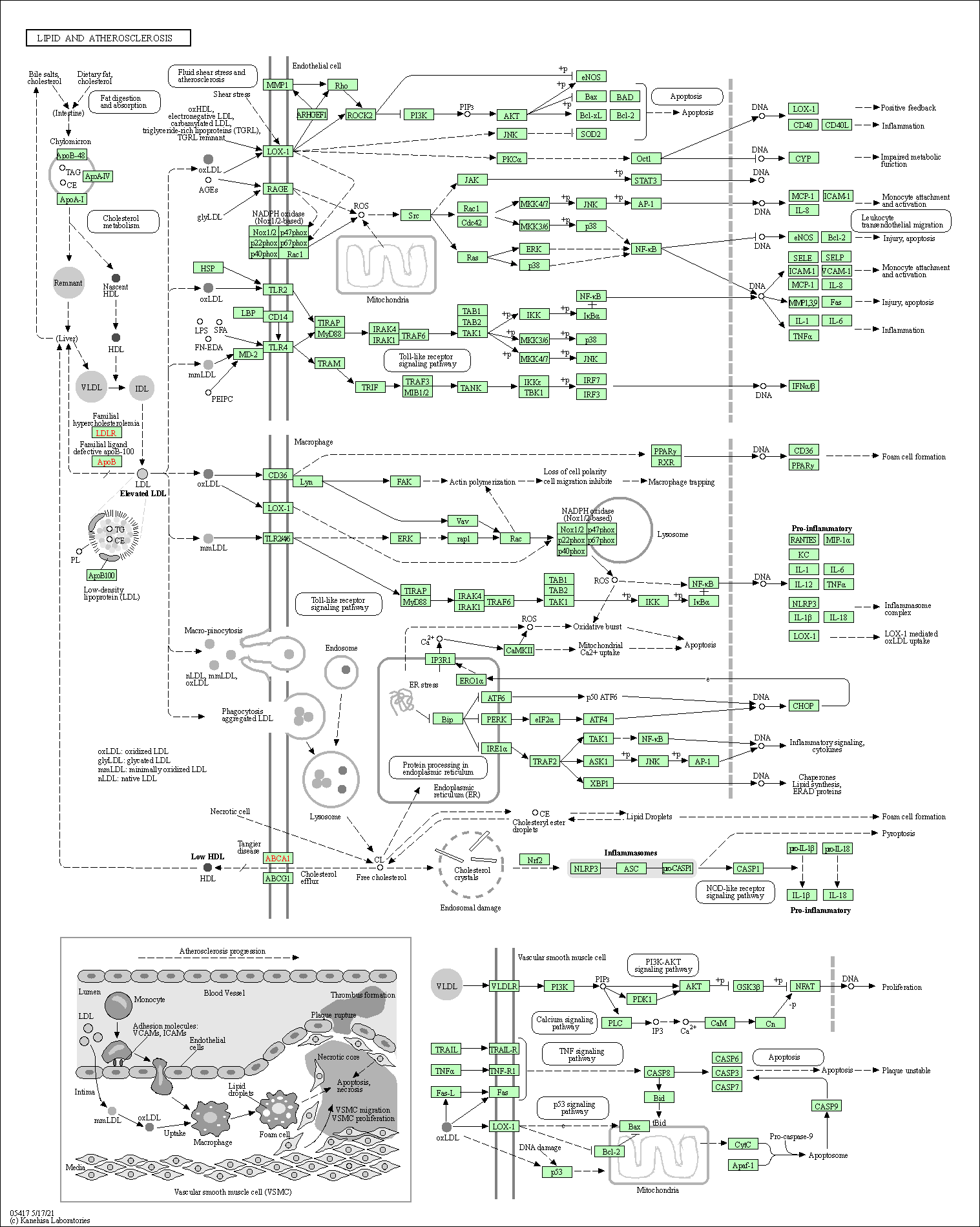

Supplement: S1 Data — (ZIP) [file pone.0274639.s001.zip › minimal data/GO+KEGG/R.KEGG/hsa05417.png]

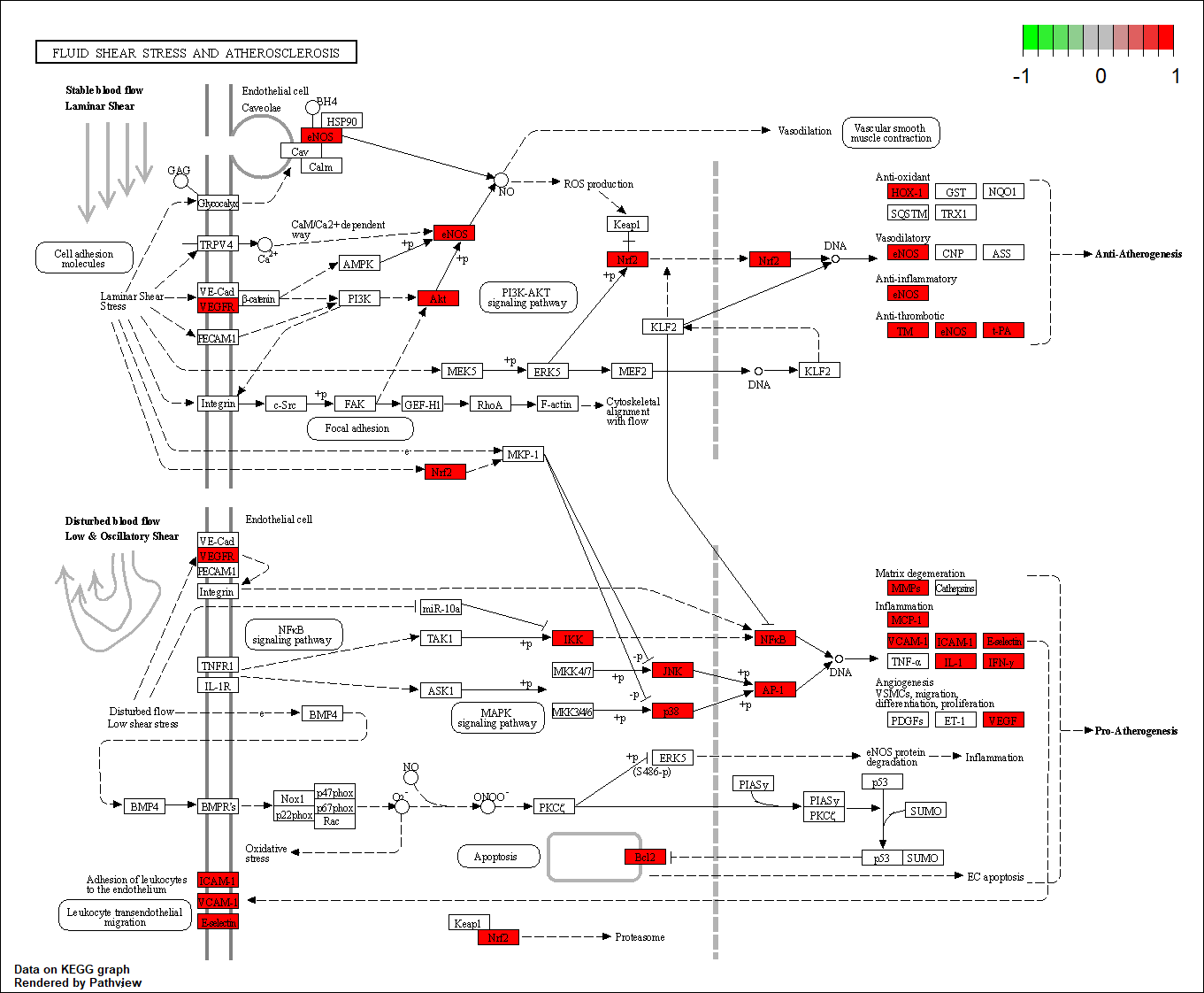

Supplement: S1 Data — (ZIP) [file pone.0274639.s001.zip › minimal data/GO+KEGG/R.KEGG/hsa05418.pathview.png]

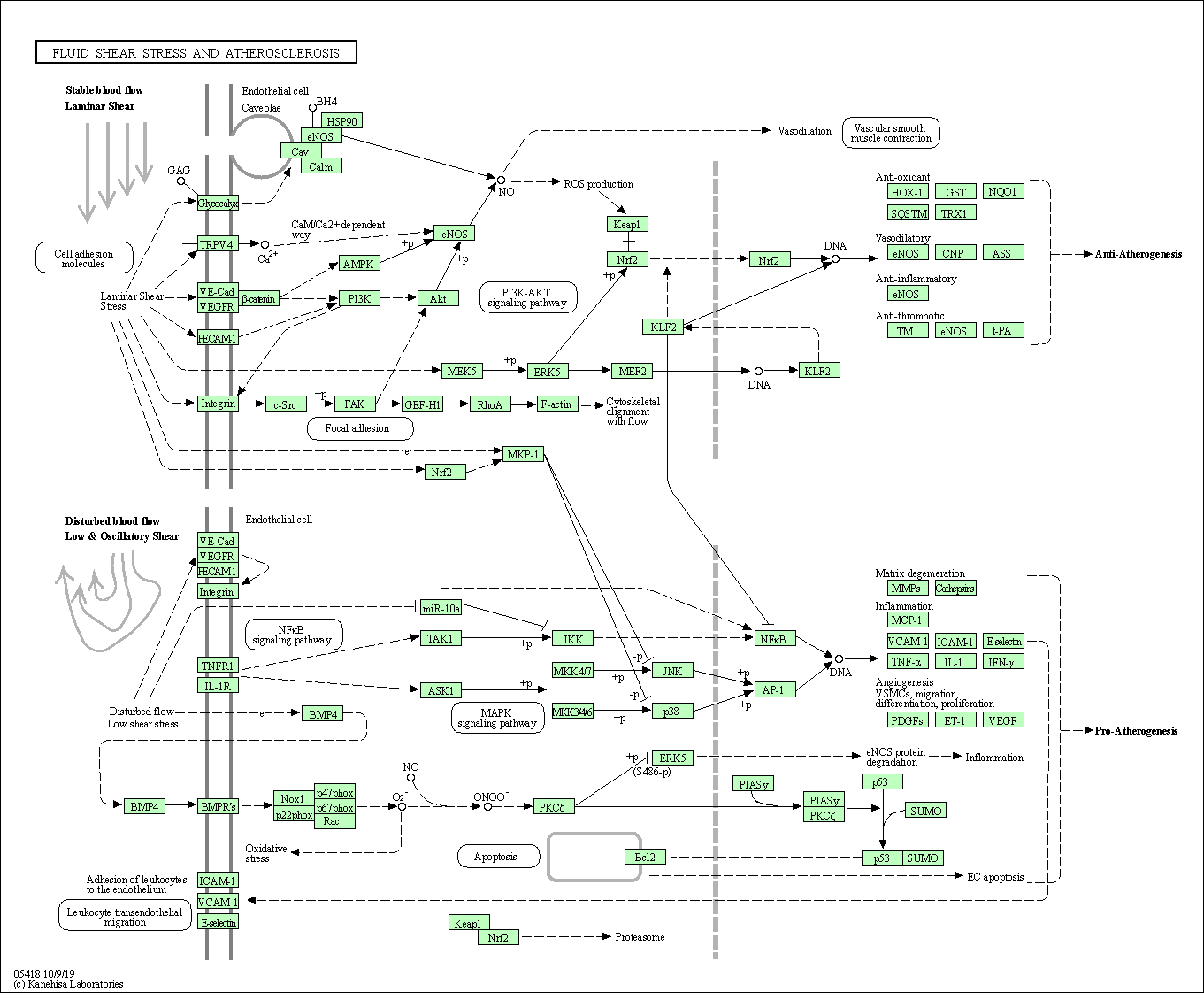

Supplement: S1 Data — (ZIP) [file pone.0274639.s001.zip › minimal data/GO+KEGG/R.KEGG/hsa05418.png]

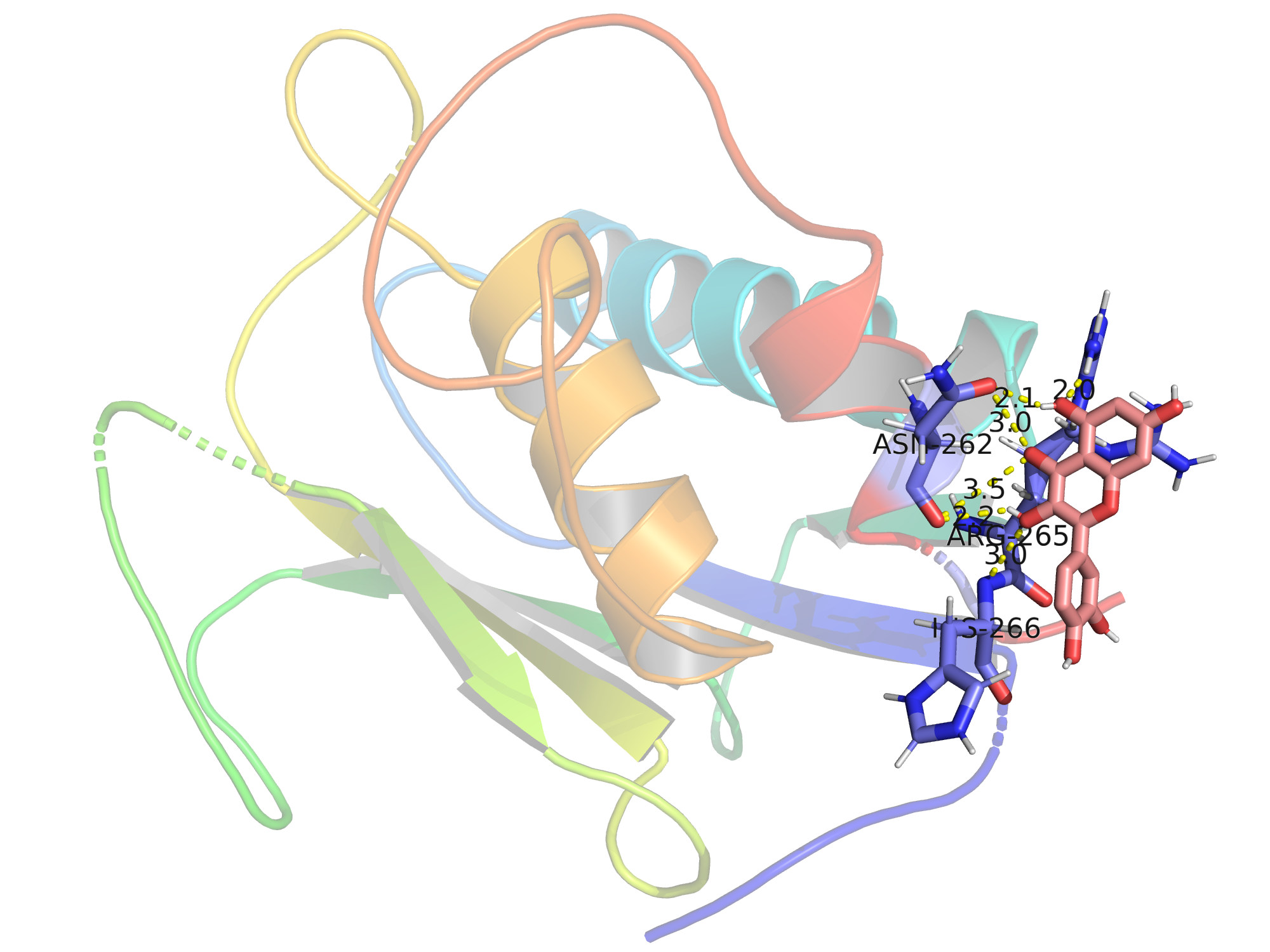

Supplement: S1 Data — (ZIP) [file pone.0274639.s001.zip › minimal data/Molecular Docking/b.Quercetin-MMP9(6ESM)/Overall Image.png]

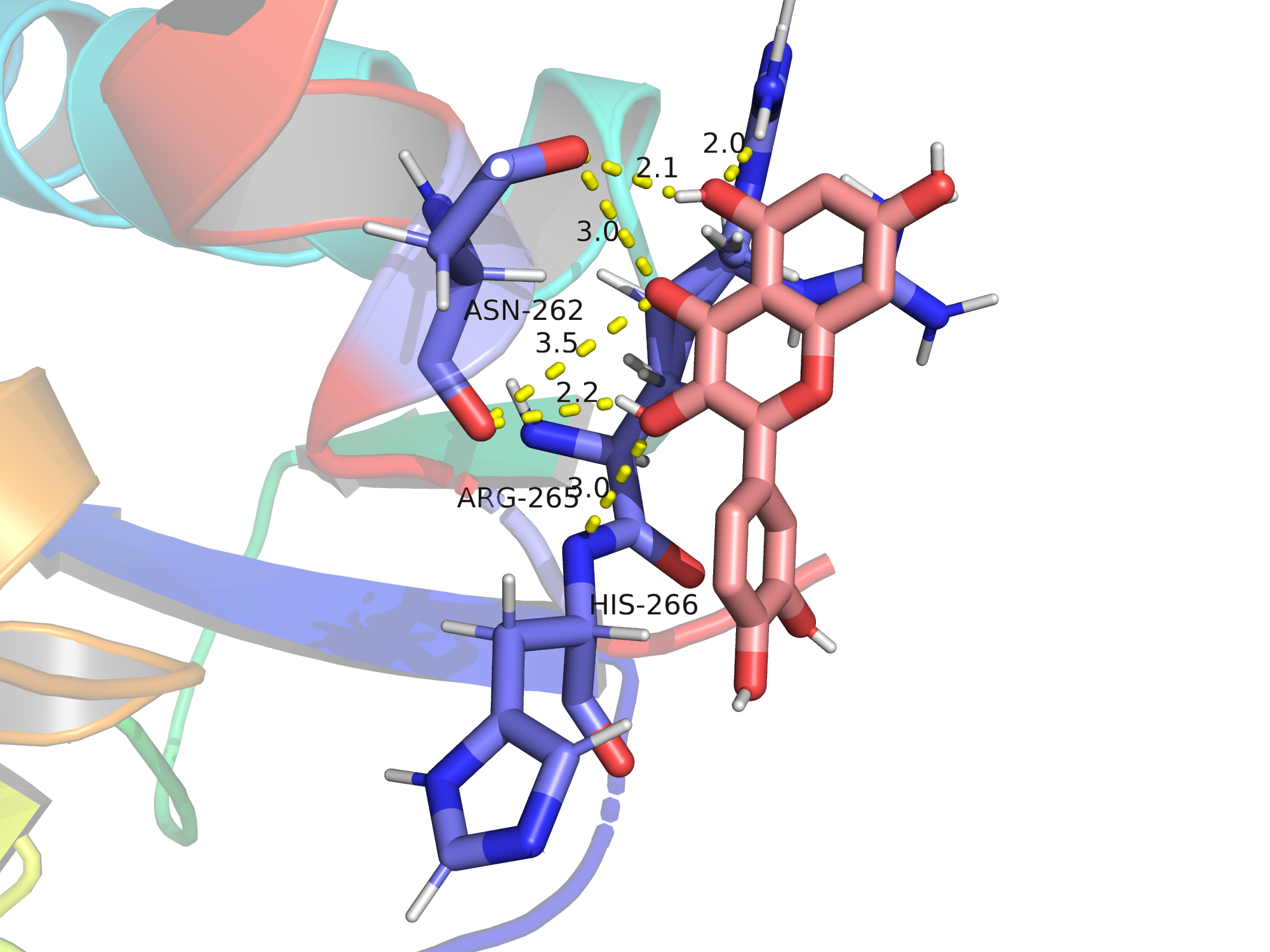

Supplement: S1 Data — (ZIP) [file pone.0274639.s001.zip › minimal data/Molecular Docking/b.Quercetin-MMP9(6ESM)/Partial image.png]
