# Supplementary figures and images for: Mechanism of gene network in the treatment of intracerebral hemorrhage by natural plant drugs in Lutong granules (part 1 of 2)
Source: PLoS One. 2022 Nov 28;17(11):e0274639. doi: 10.1371/journal.pone.0274639 (PMC9704616; doi:10.1371/journal.pone.0274639)

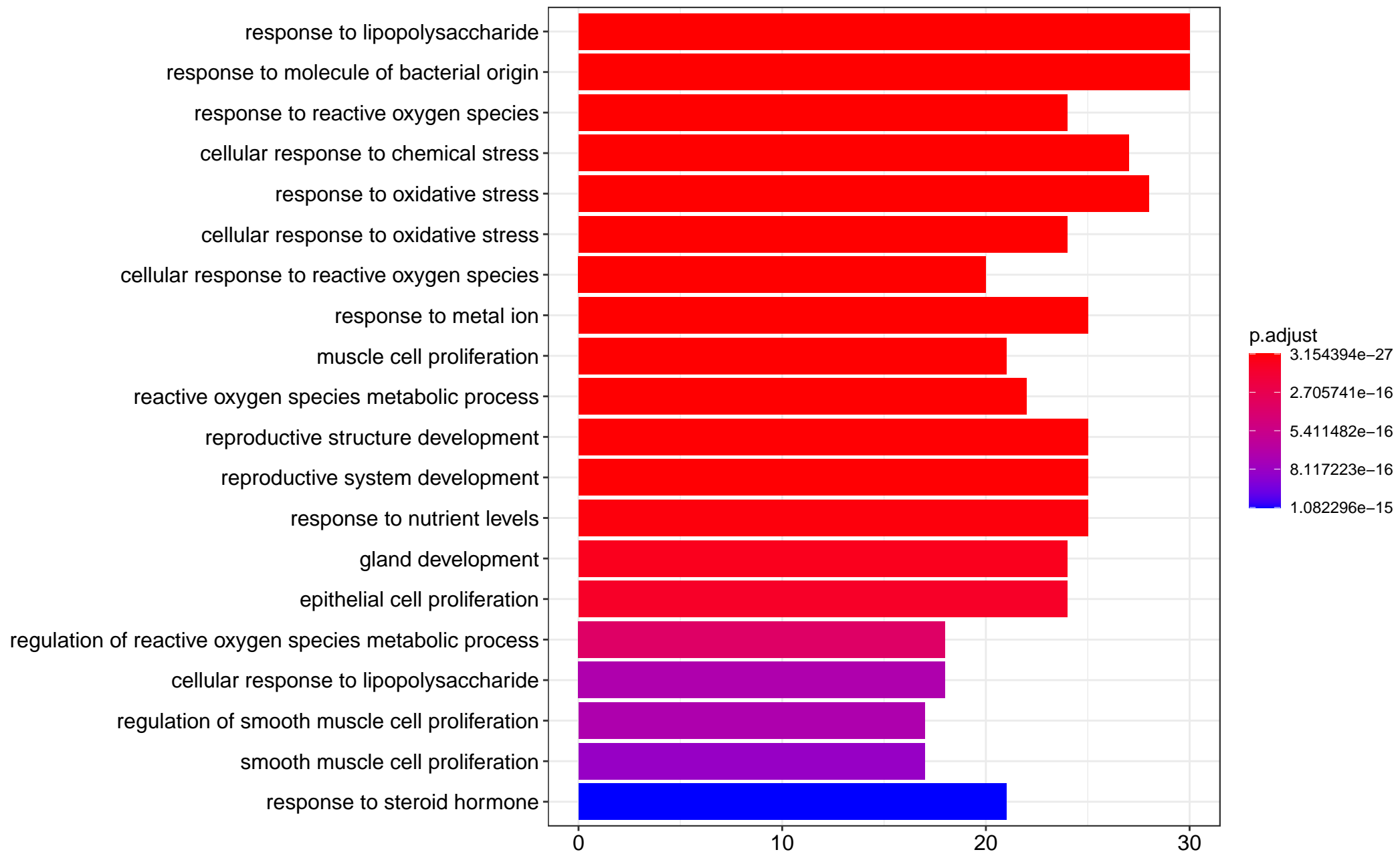

Supplement: S1 Data — (ZIP) [file pone.0274639.s001.zip › minimal data/GO+KEGG/R.GO/BP.barplot.pdf]

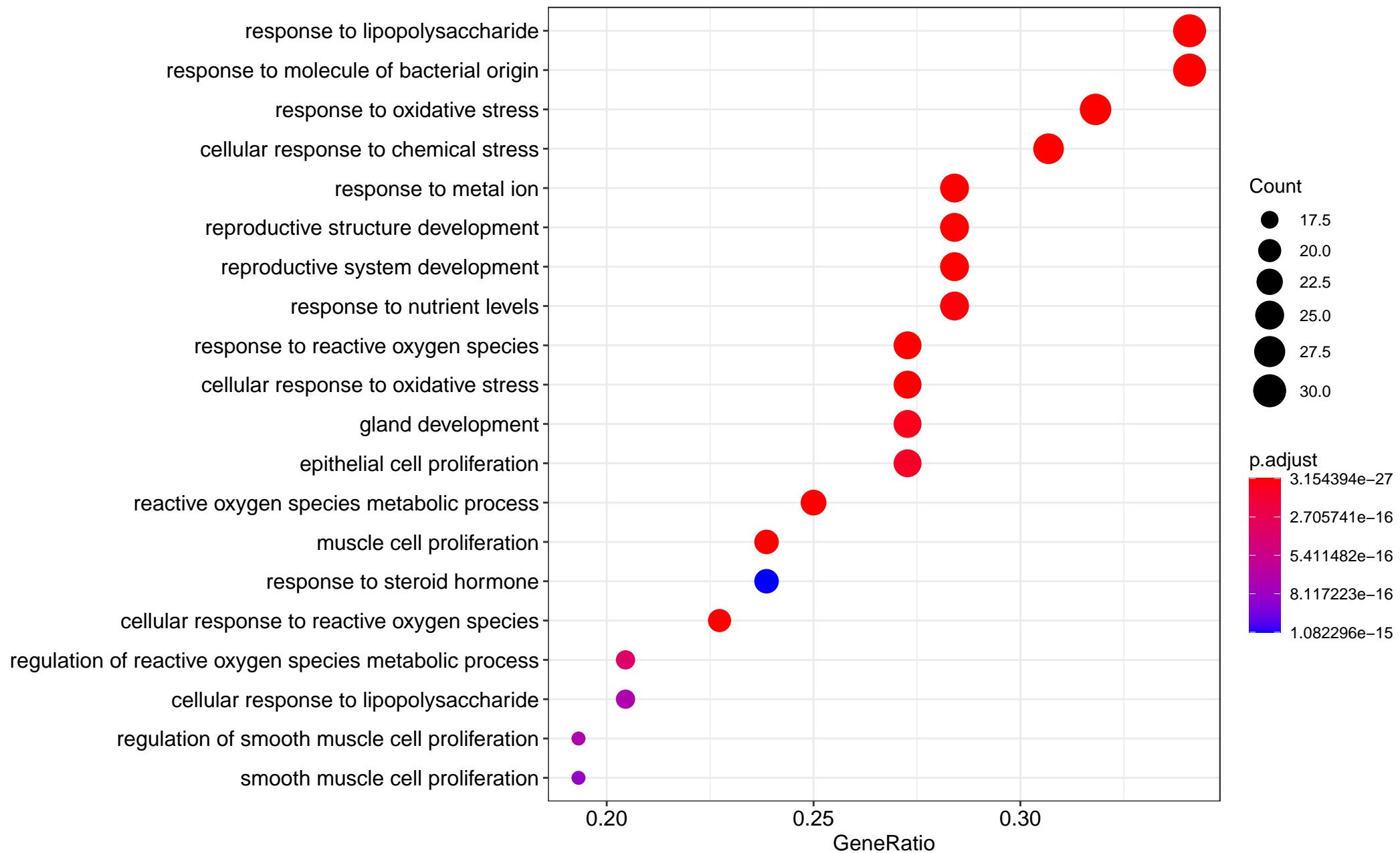

Supplement: S1 Data — (ZIP) [file pone.0274639.s001.zip › minimal data/GO+KEGG/R.GO/BP.bubble.pdf]

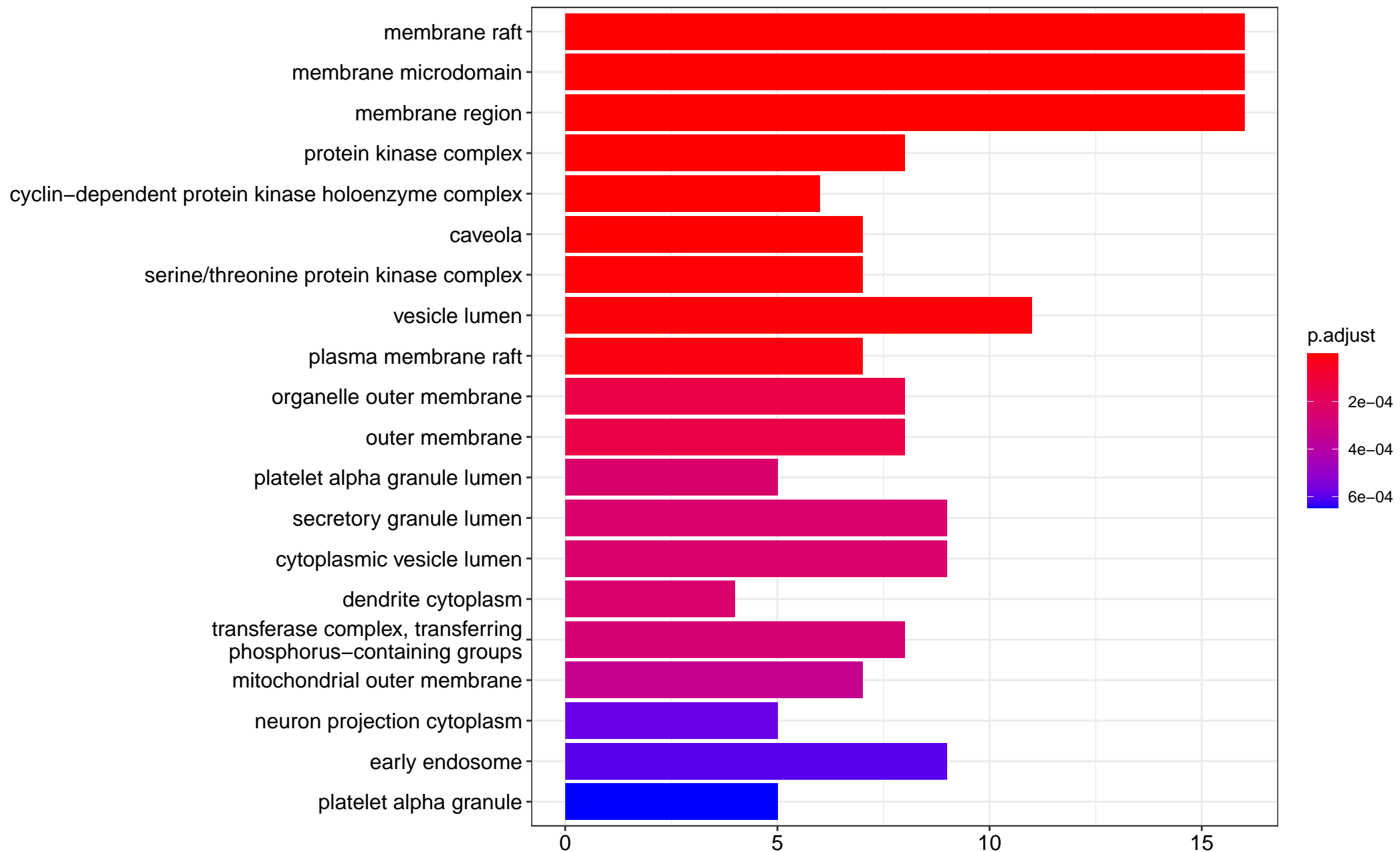

Supplement: S1 Data — (ZIP) [file pone.0274639.s001.zip › minimal data/GO+KEGG/R.GO/CC.barplot.pdf]

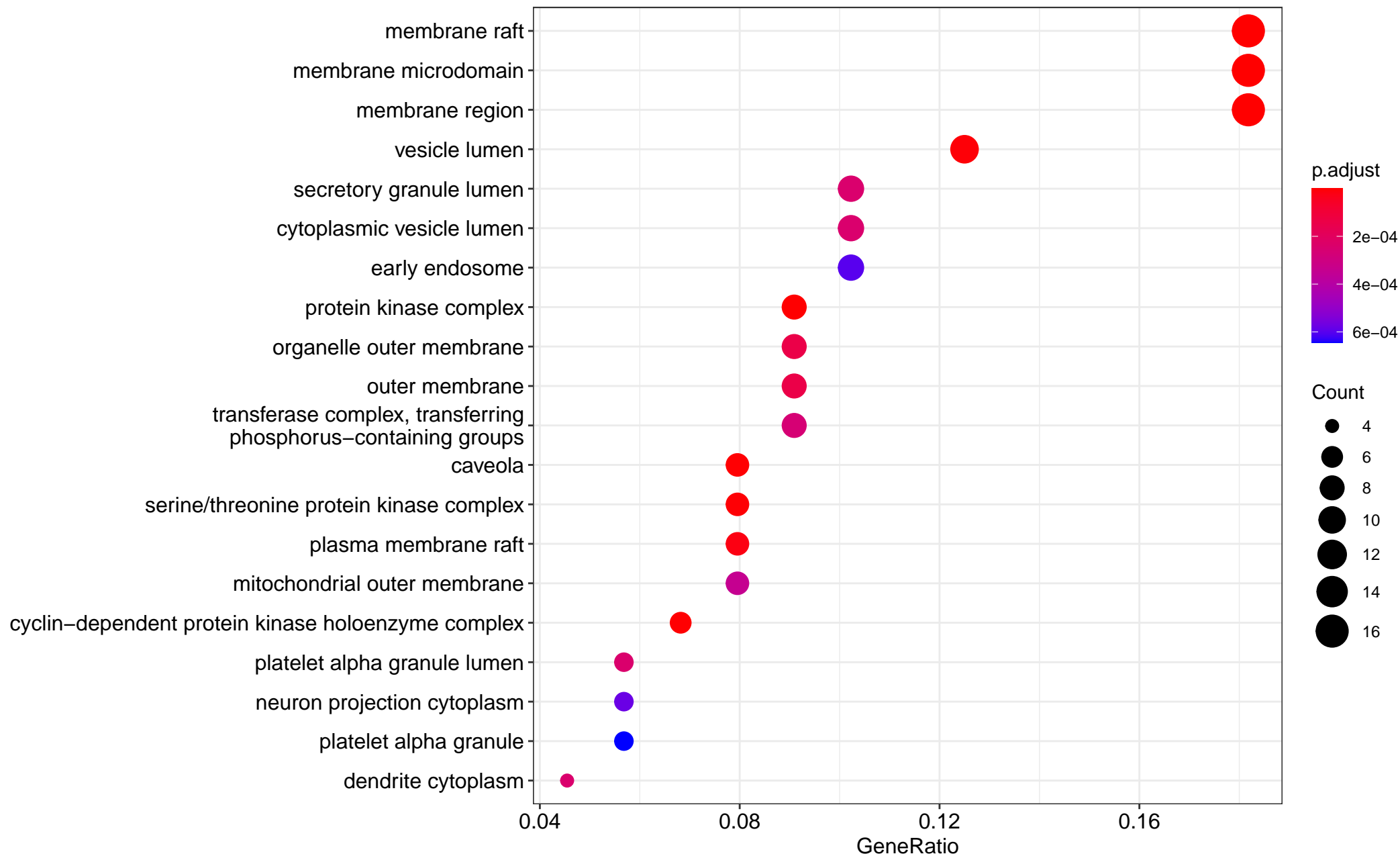

Supplement: S1 Data — (ZIP) [file pone.0274639.s001.zip › minimal data/GO+KEGG/R.GO/CC.bubble.pdf]

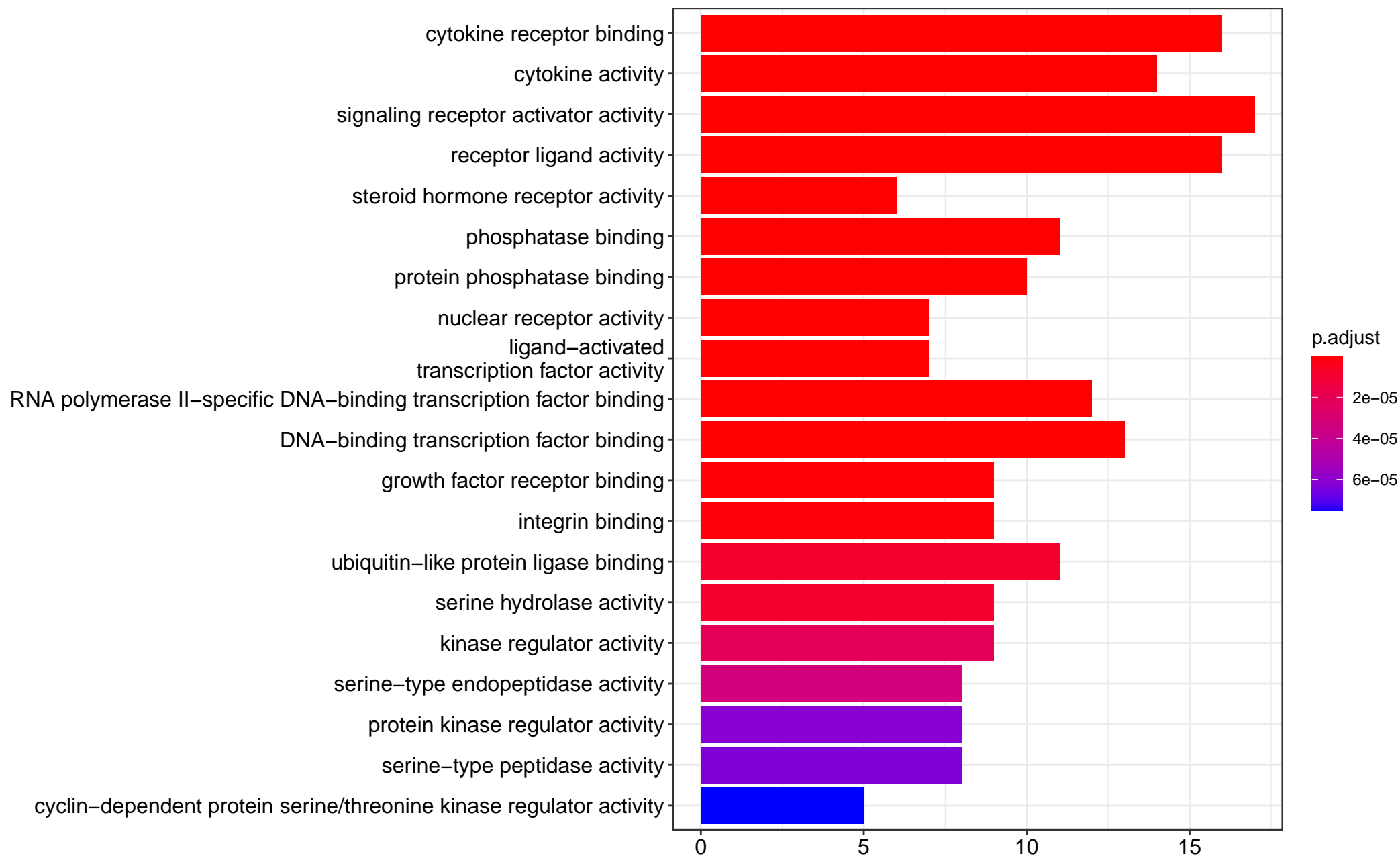

Supplement: S1 Data — (ZIP) [file pone.0274639.s001.zip › minimal data/GO+KEGG/R.GO/MF.barplot.pdf]

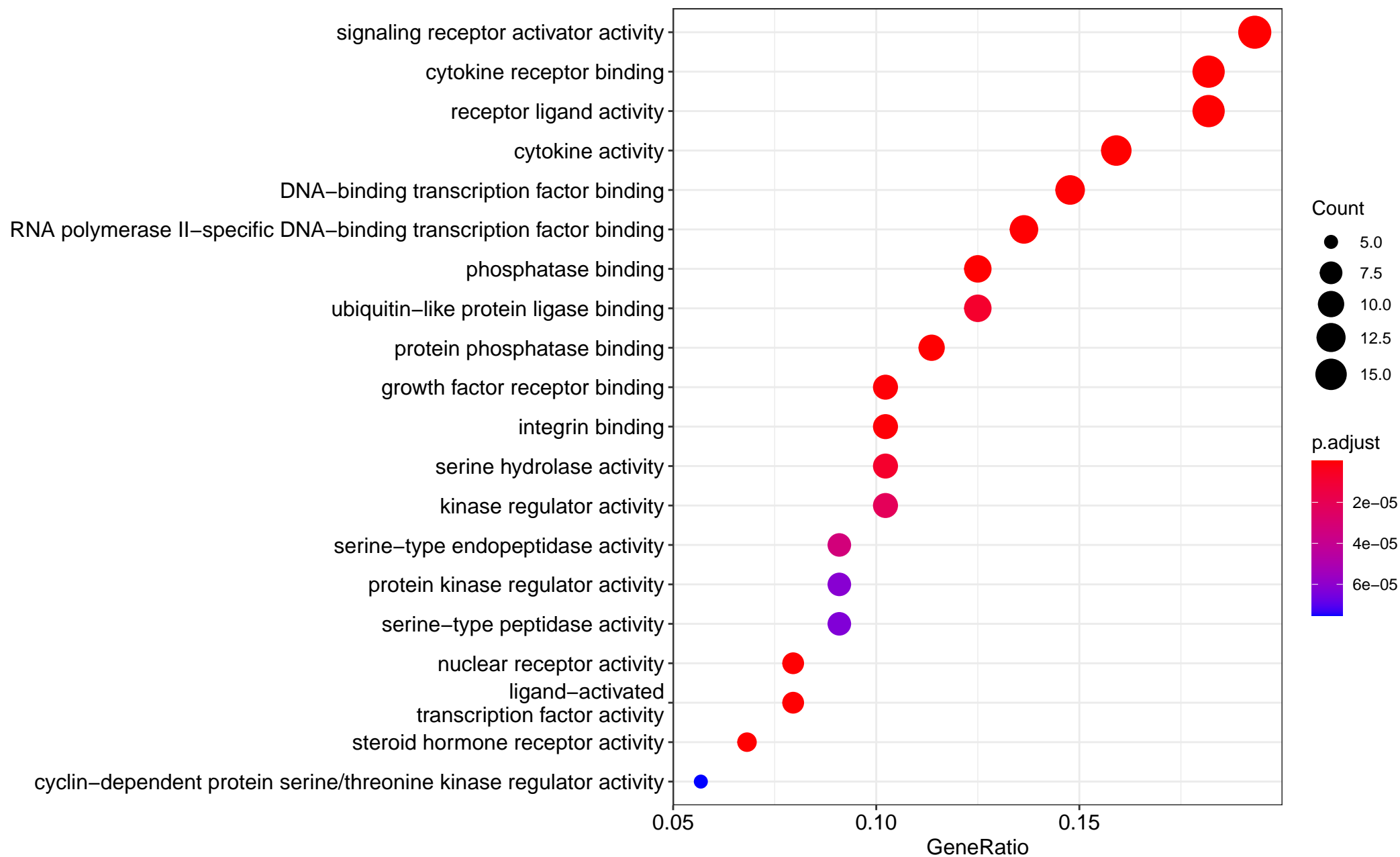

Supplement: S1 Data — (ZIP) [file pone.0274639.s001.zip › minimal data/GO+KEGG/R.GO/MF.bubble.pdf]

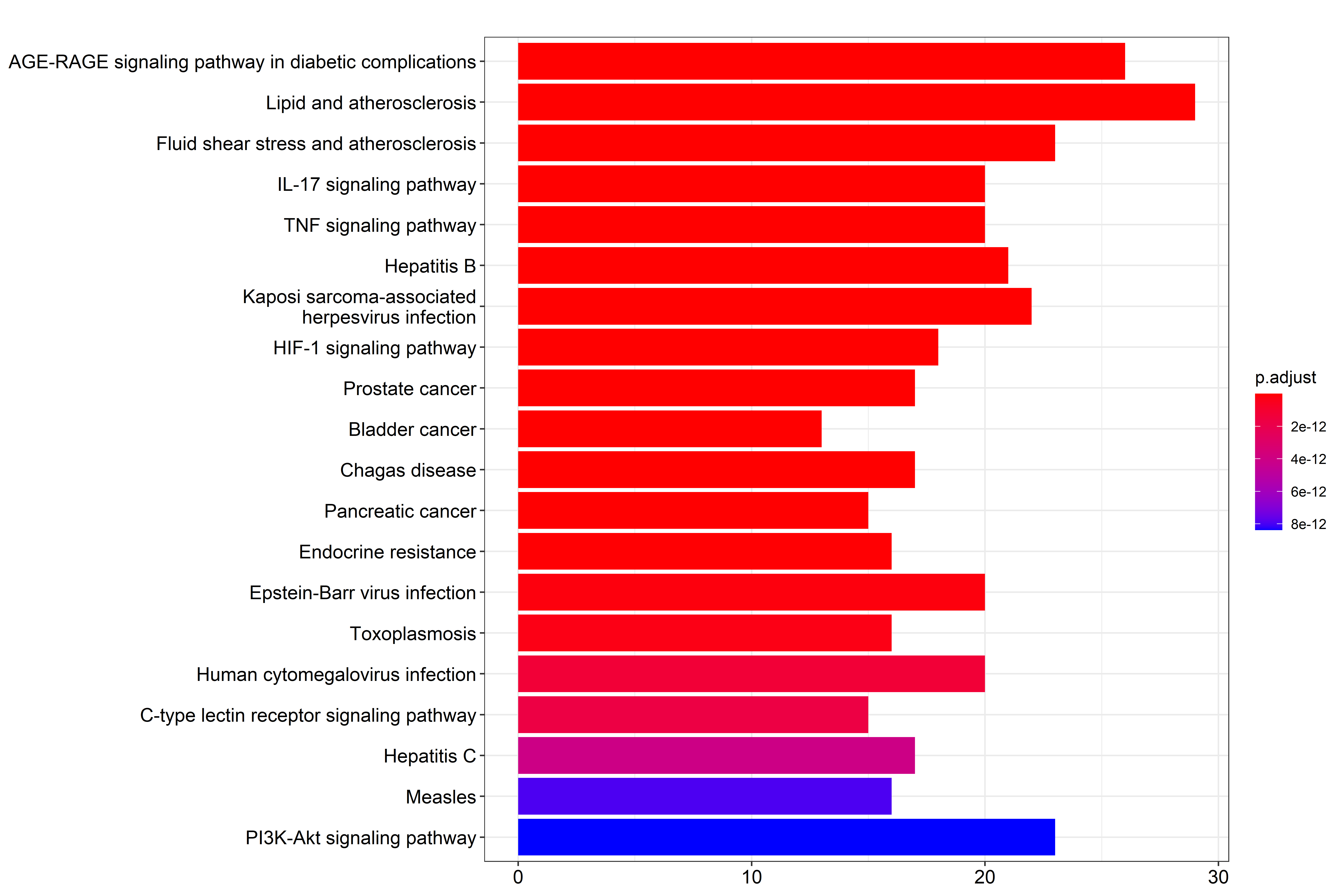

Supplement: S1 Data — (ZIP) [file pone.0274639.s001.zip › minimal data/GO+KEGG/R.KEGG/barplot.tiff]

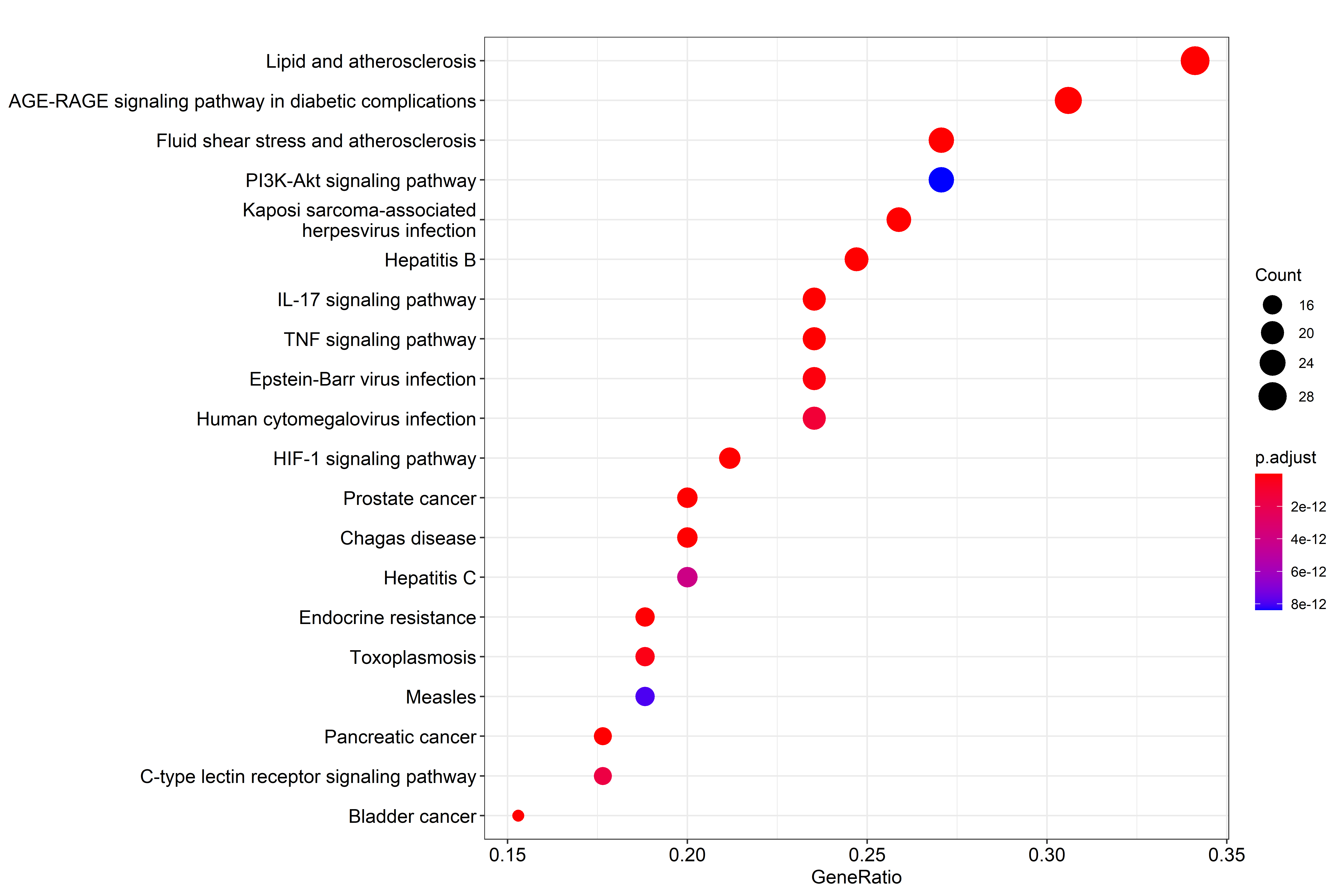

Supplement: S1 Data — (ZIP) [file pone.0274639.s001.zip › minimal data/GO+KEGG/R.KEGG/dotplot.tiff]

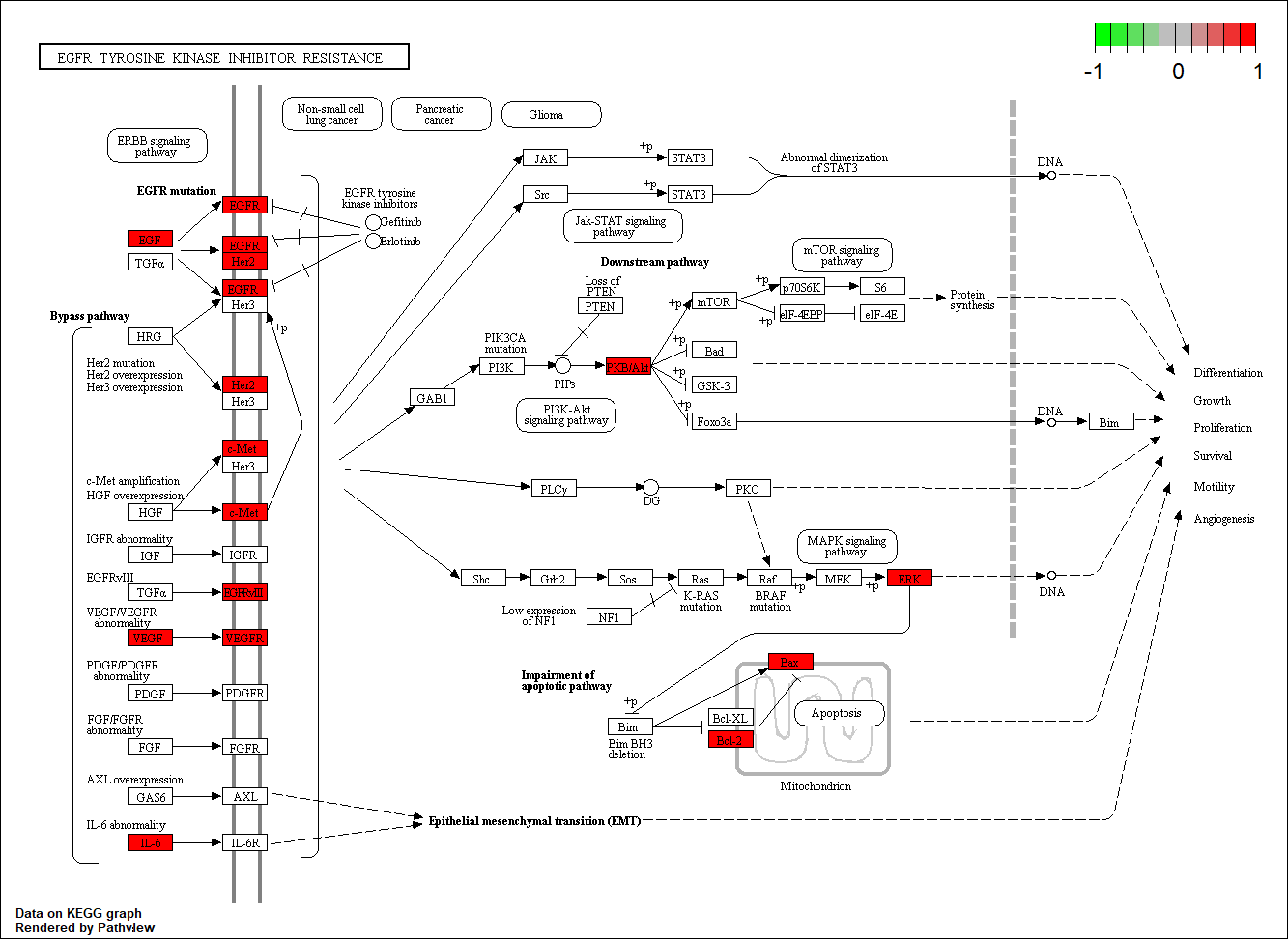

Supplement: S1 Data — (ZIP) [file pone.0274639.s001.zip › minimal data/GO+KEGG/R.KEGG/hsa01521.pathview.png]

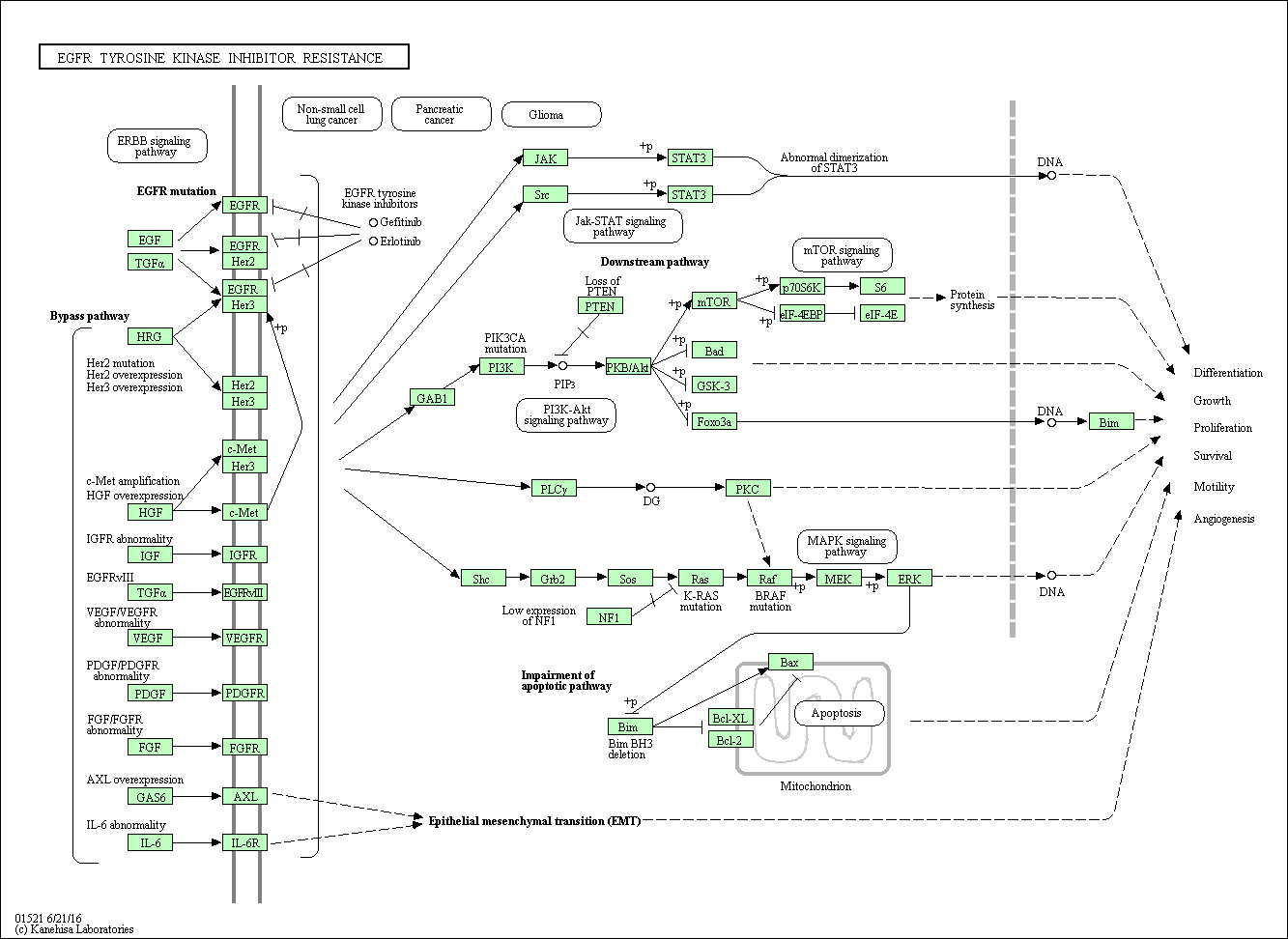

Supplement: S1 Data — (ZIP) [file pone.0274639.s001.zip › minimal data/GO+KEGG/R.KEGG/hsa01521.png]

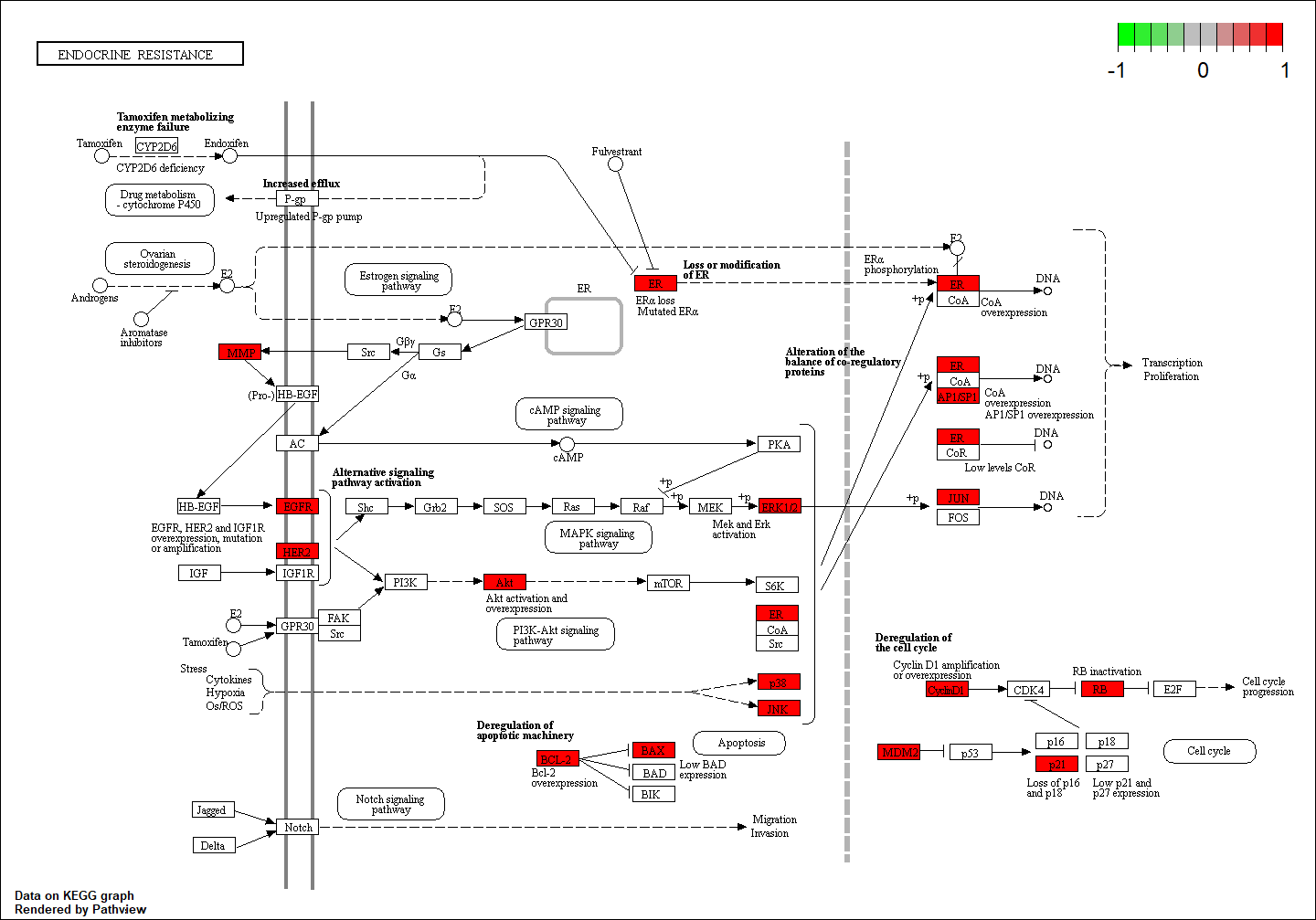

Supplement: S1 Data — (ZIP) [file pone.0274639.s001.zip › minimal data/GO+KEGG/R.KEGG/hsa01522.pathview.png]

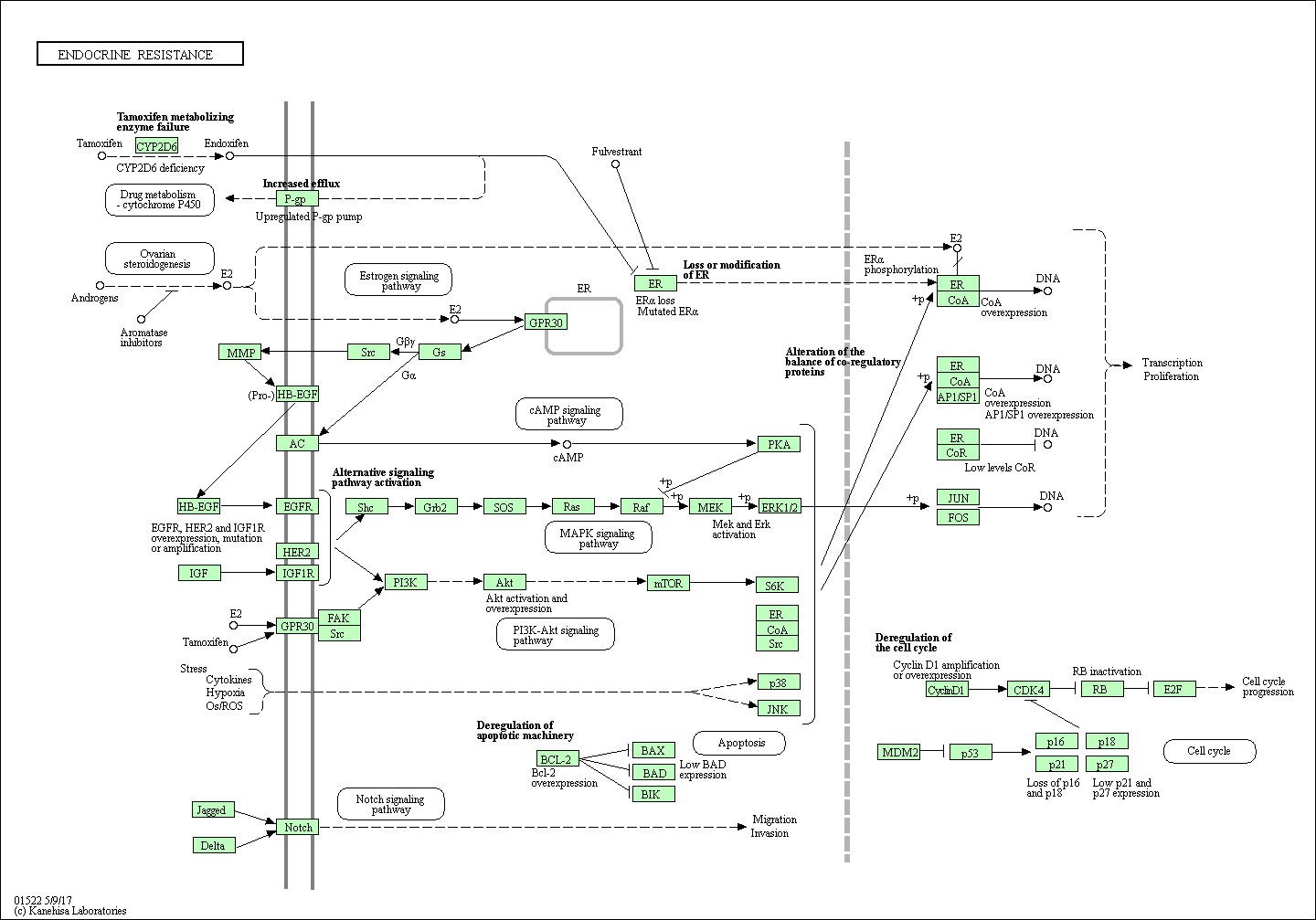

Supplement: S1 Data — (ZIP) [file pone.0274639.s001.zip › minimal data/GO+KEGG/R.KEGG/hsa01522.png]

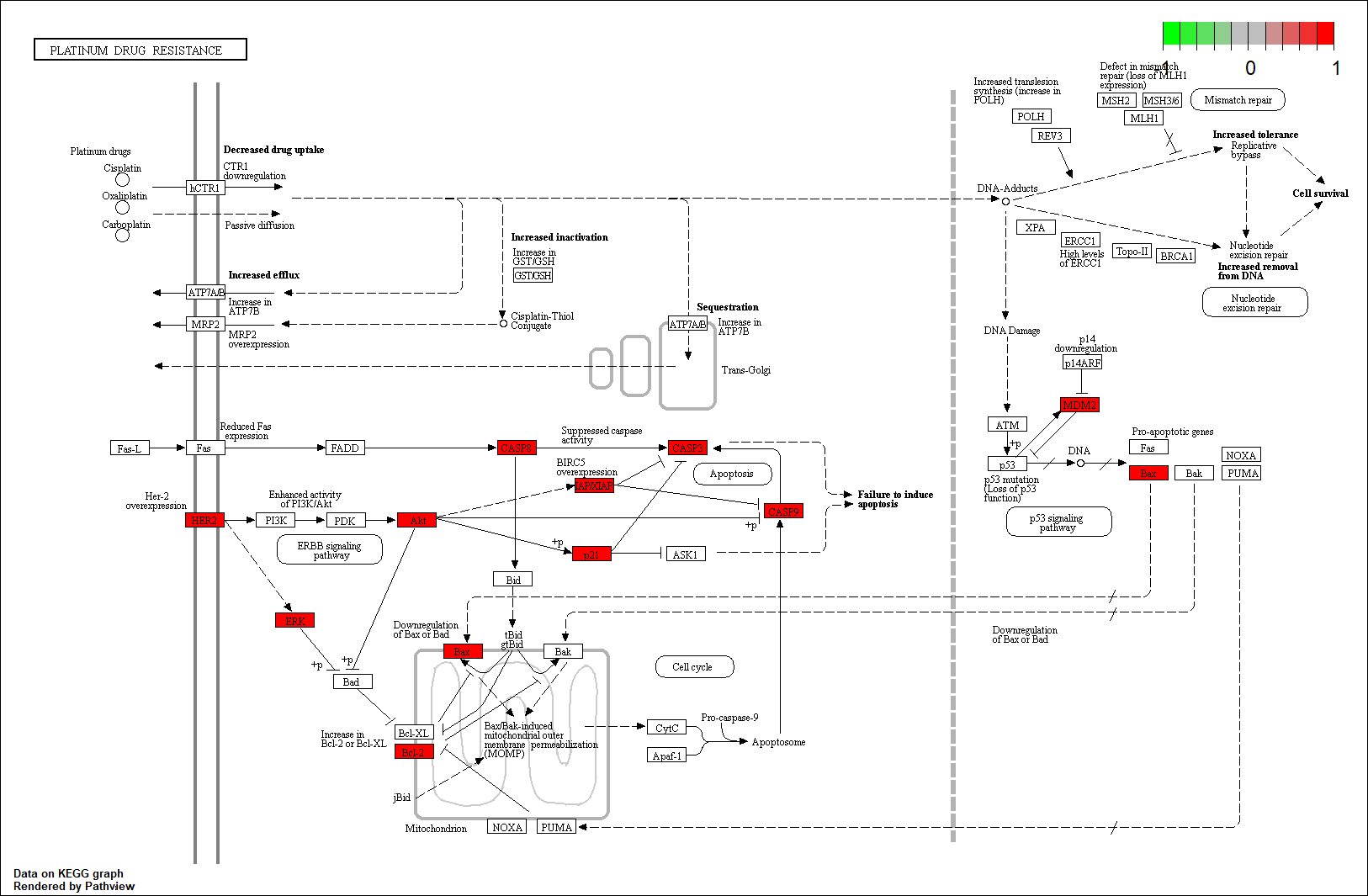

Supplement: S1 Data — (ZIP) [file pone.0274639.s001.zip › minimal data/GO+KEGG/R.KEGG/hsa01524.pathview.png]

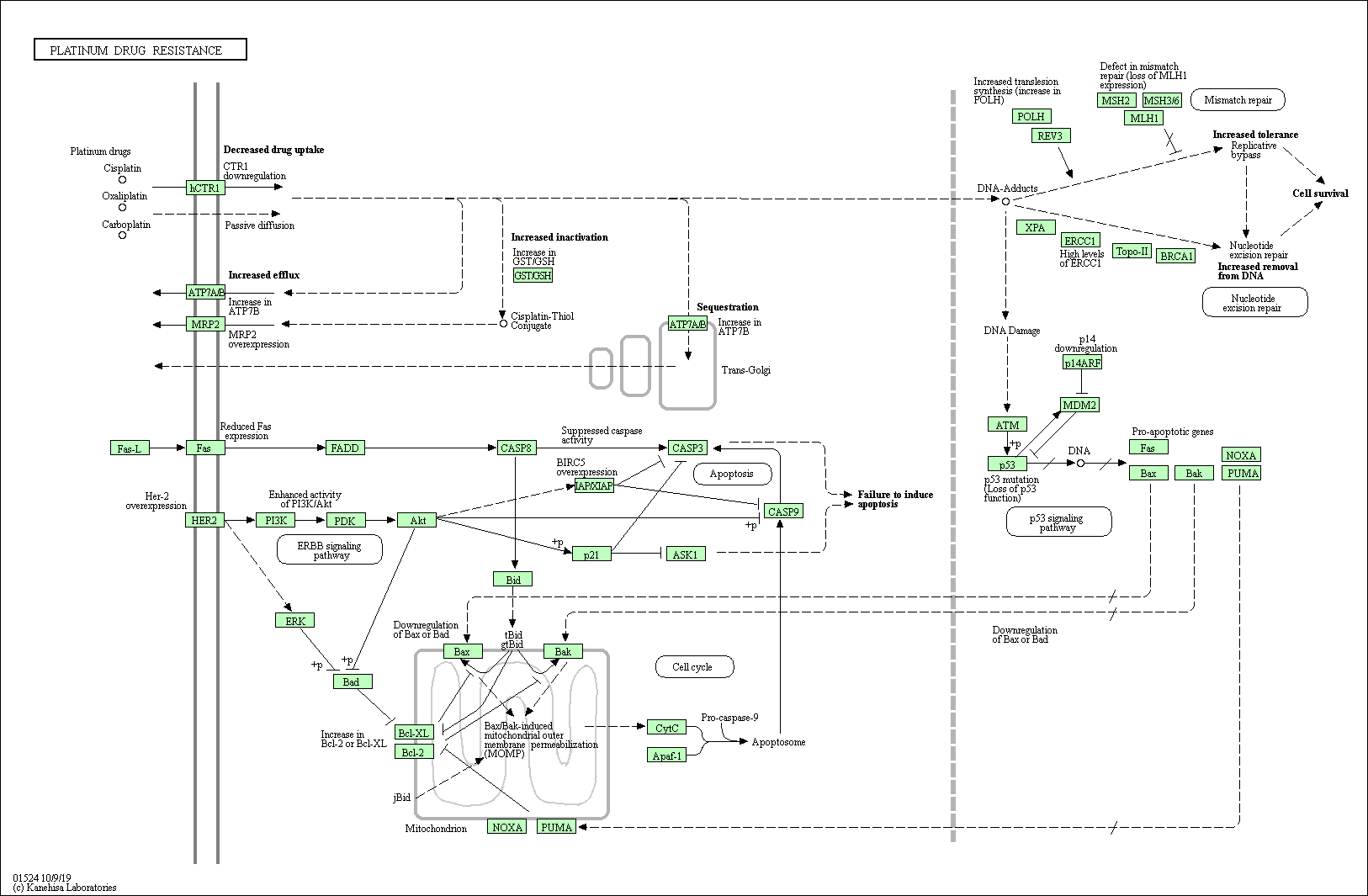

Supplement: S1 Data — (ZIP) [file pone.0274639.s001.zip › minimal data/GO+KEGG/R.KEGG/hsa01524.png]

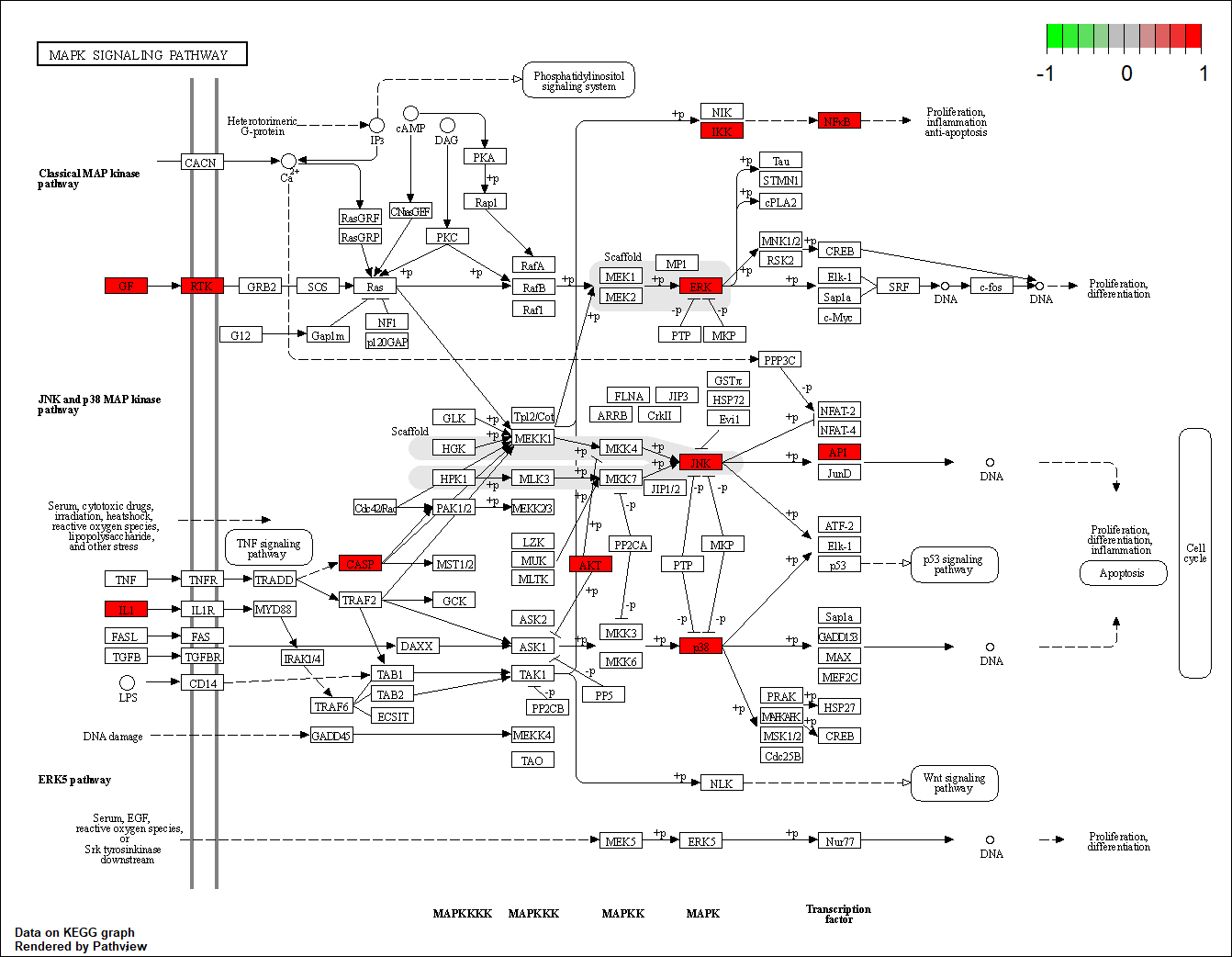

Supplement: S1 Data — (ZIP) [file pone.0274639.s001.zip › minimal data/GO+KEGG/R.KEGG/hsa04010.pathview.png]

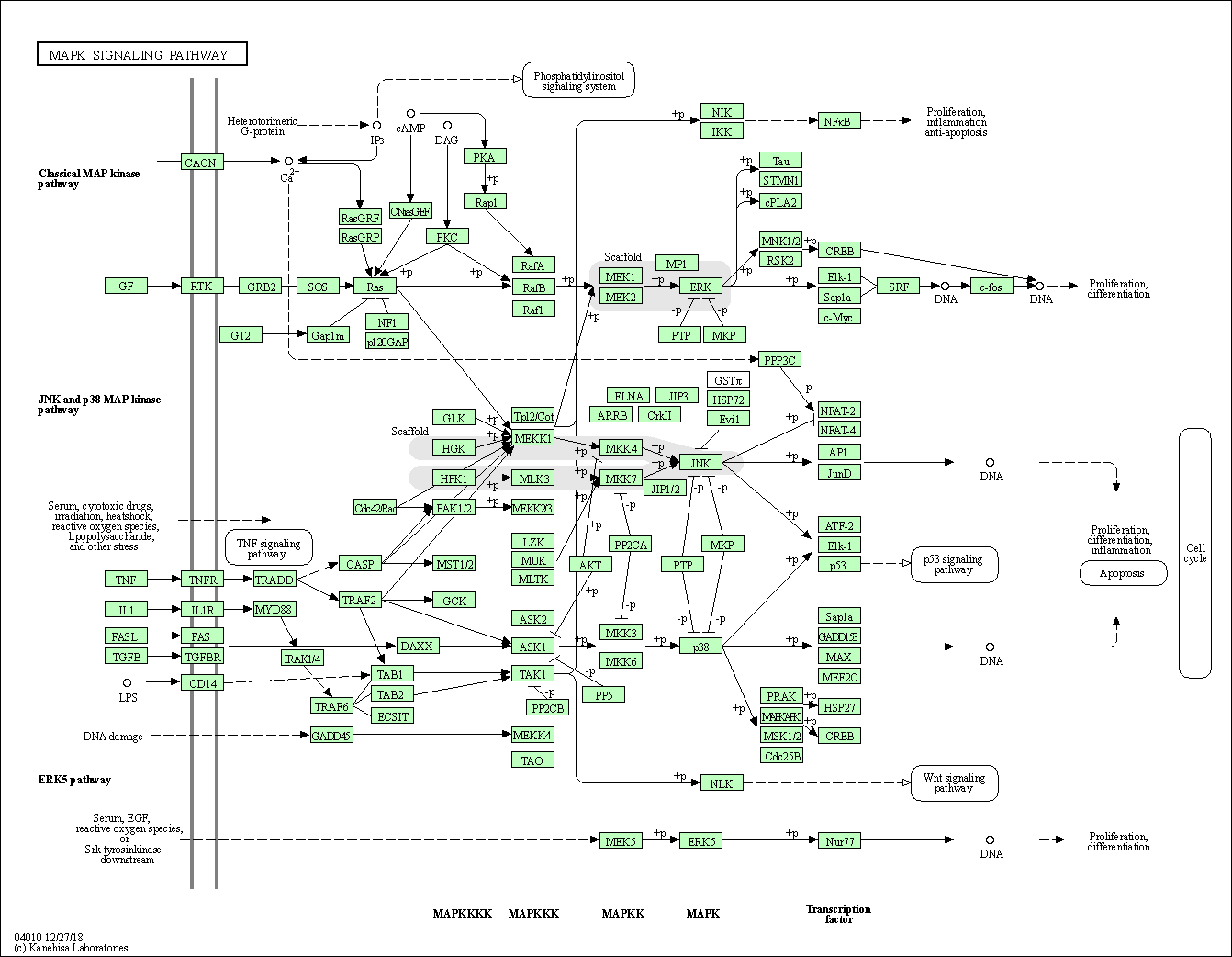

Supplement: S1 Data — (ZIP) [file pone.0274639.s001.zip › minimal data/GO+KEGG/R.KEGG/hsa04010.png]

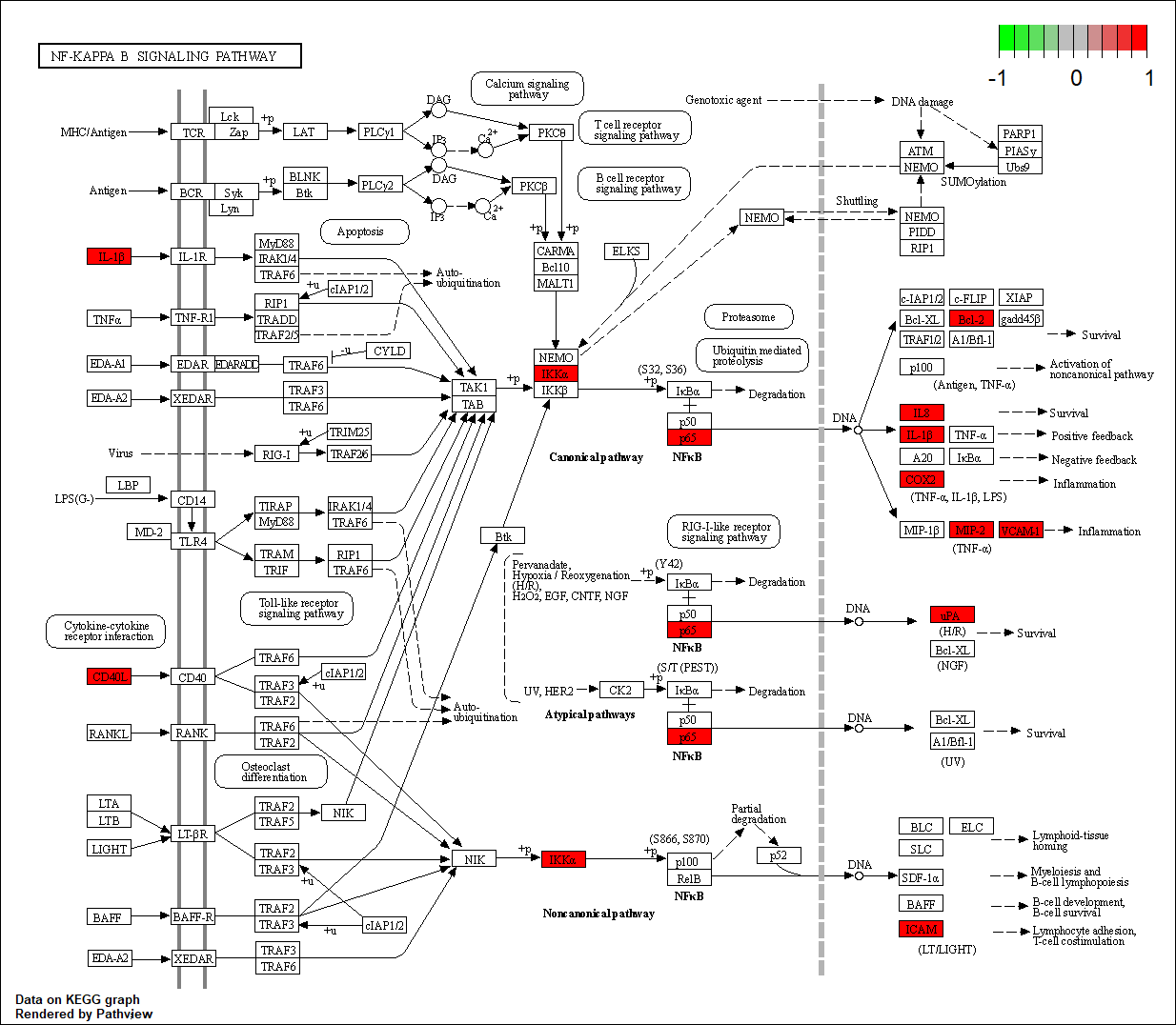

Supplement: S1 Data — (ZIP) [file pone.0274639.s001.zip › minimal data/GO+KEGG/R.KEGG/hsa04064.pathview.png]

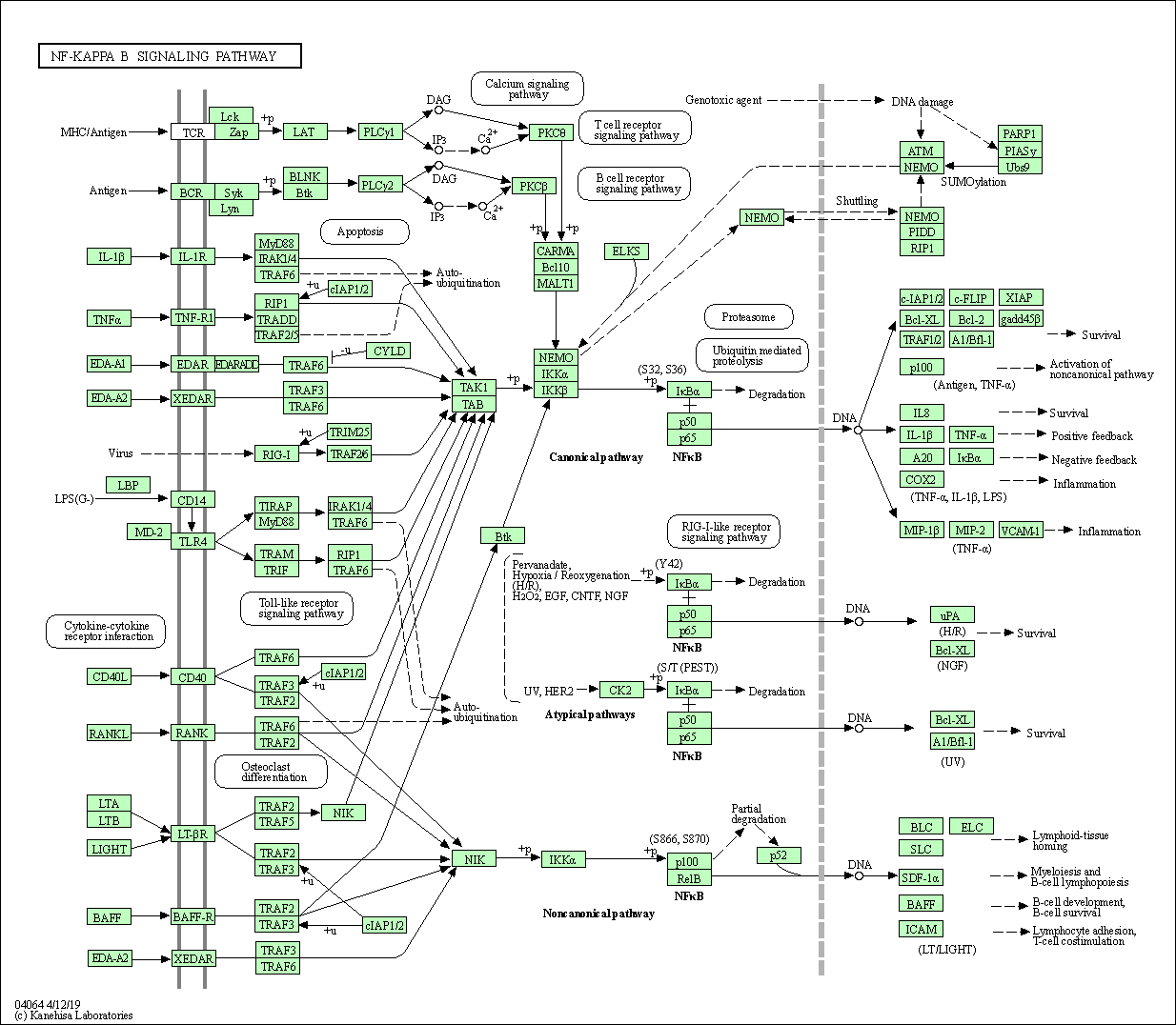

Supplement: S1 Data — (ZIP) [file pone.0274639.s001.zip › minimal data/GO+KEGG/R.KEGG/hsa04064.png]

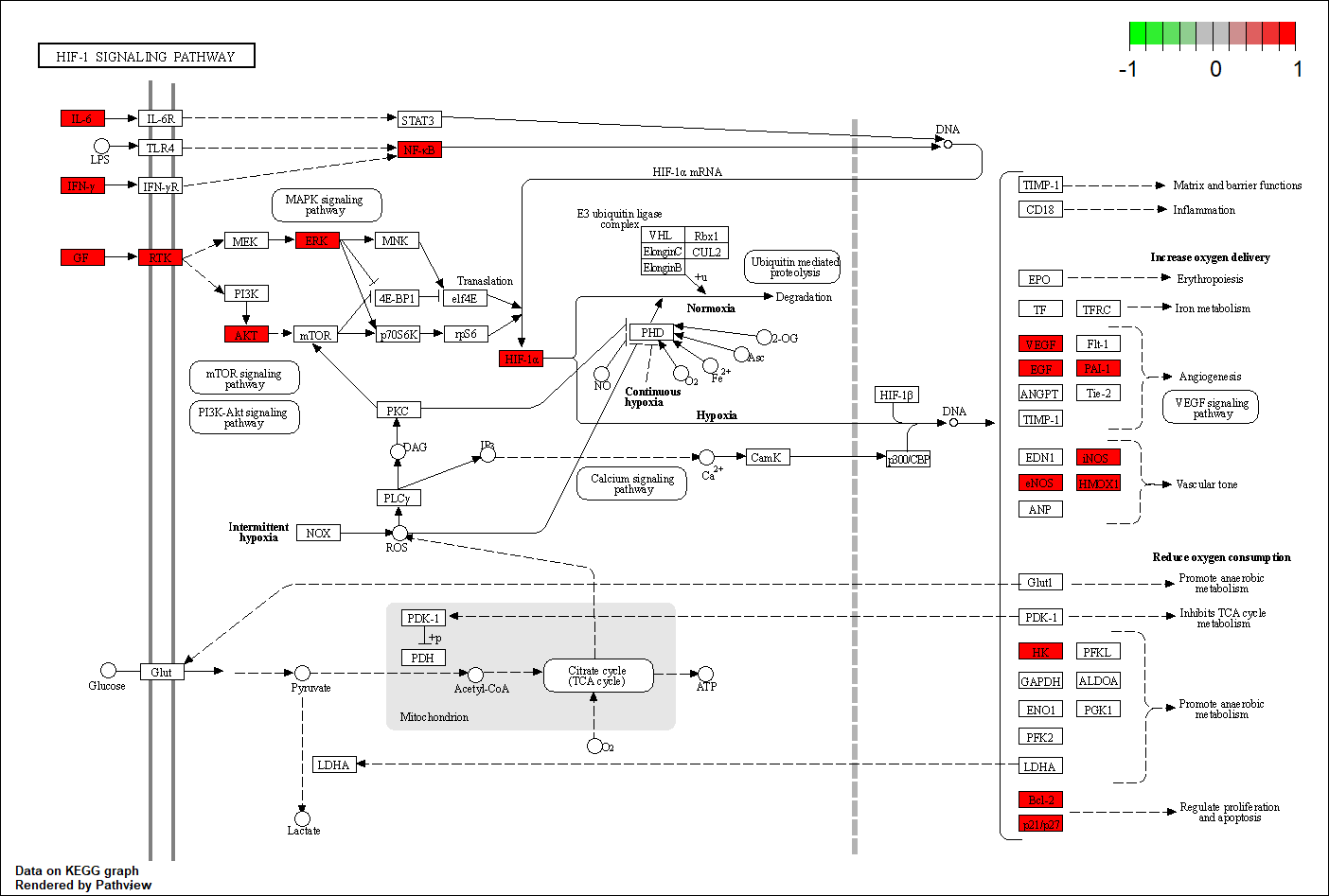

Supplement: S1 Data — (ZIP) [file pone.0274639.s001.zip › minimal data/GO+KEGG/R.KEGG/hsa04066.pathview.png]

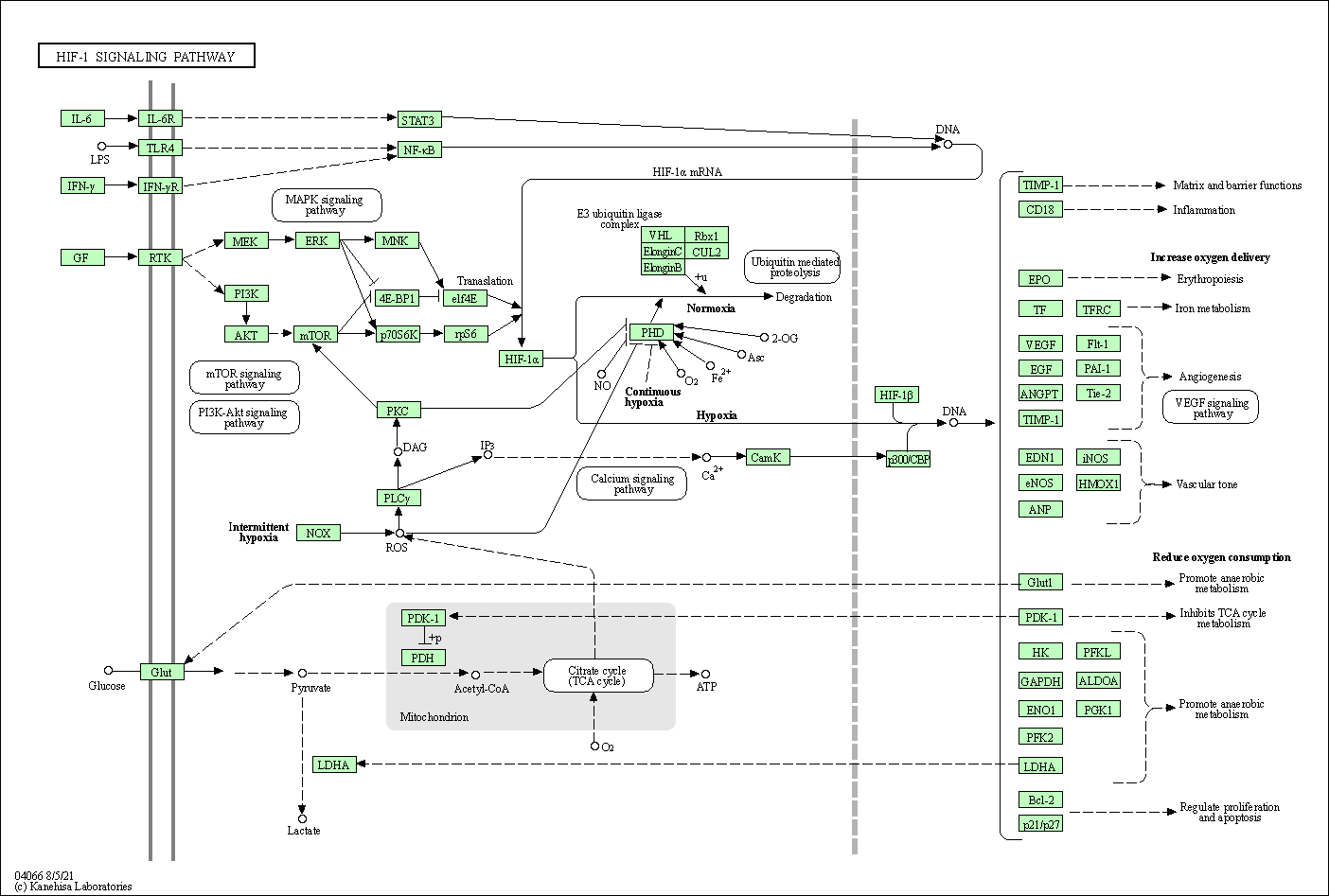

Supplement: S1 Data — (ZIP) [file pone.0274639.s001.zip › minimal data/GO+KEGG/R.KEGG/hsa04066.png]

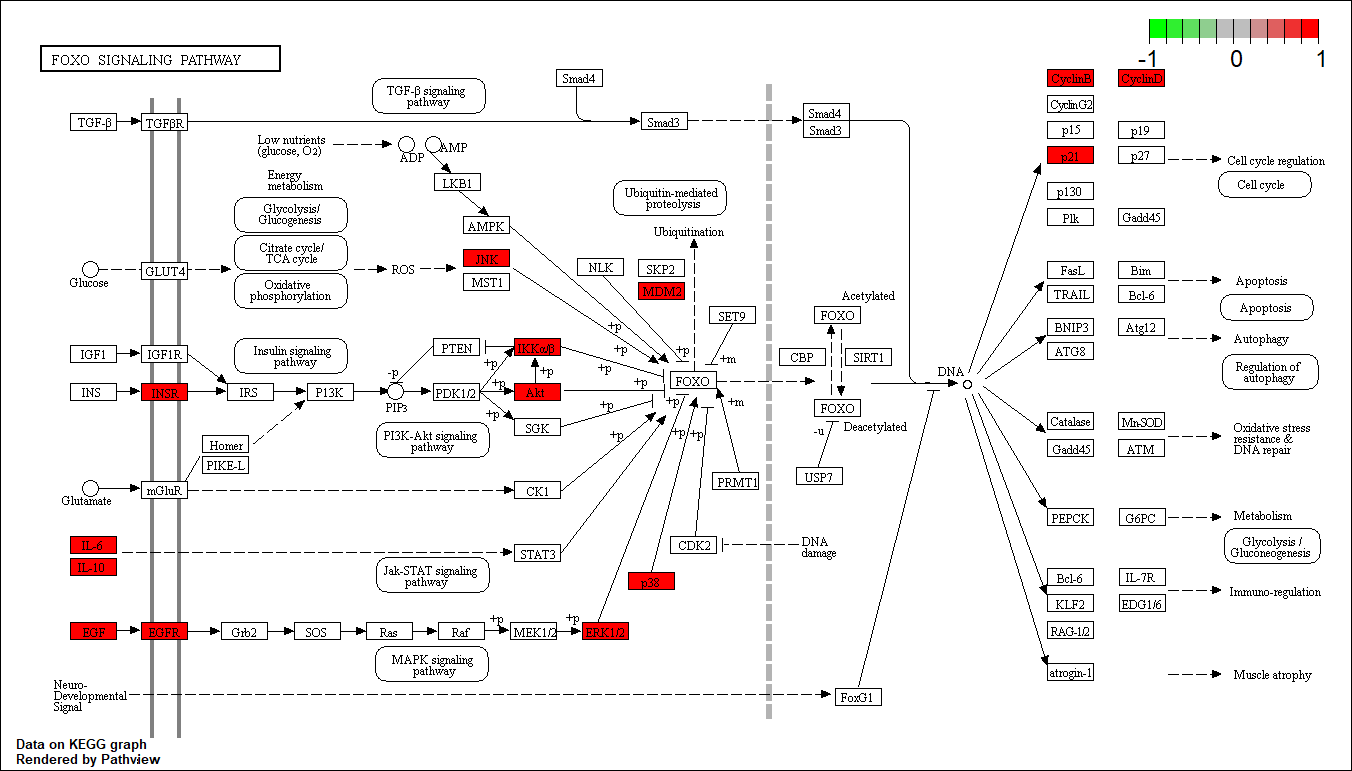

Supplement: S1 Data — (ZIP) [file pone.0274639.s001.zip › minimal data/GO+KEGG/R.KEGG/hsa04068.pathview.png]

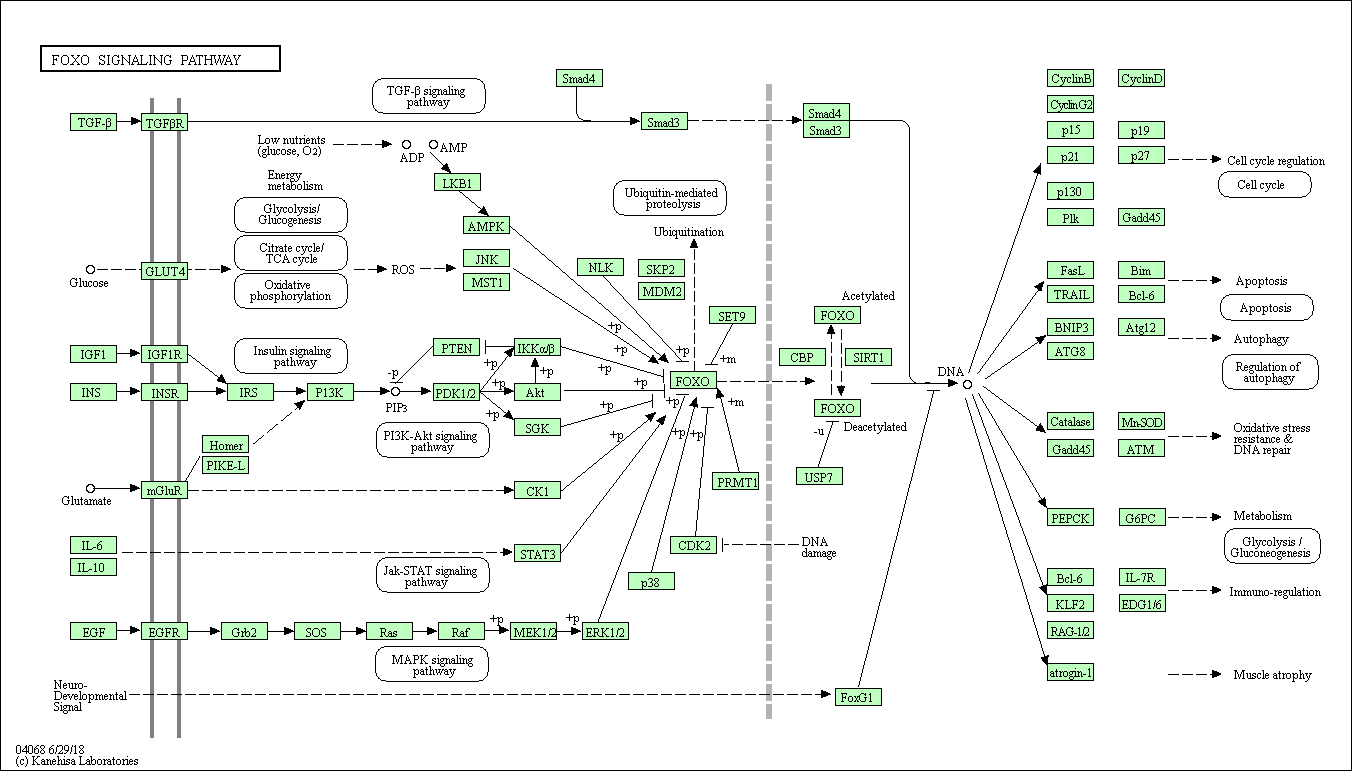

Supplement: S1 Data — (ZIP) [file pone.0274639.s001.zip › minimal data/GO+KEGG/R.KEGG/hsa04068.png]

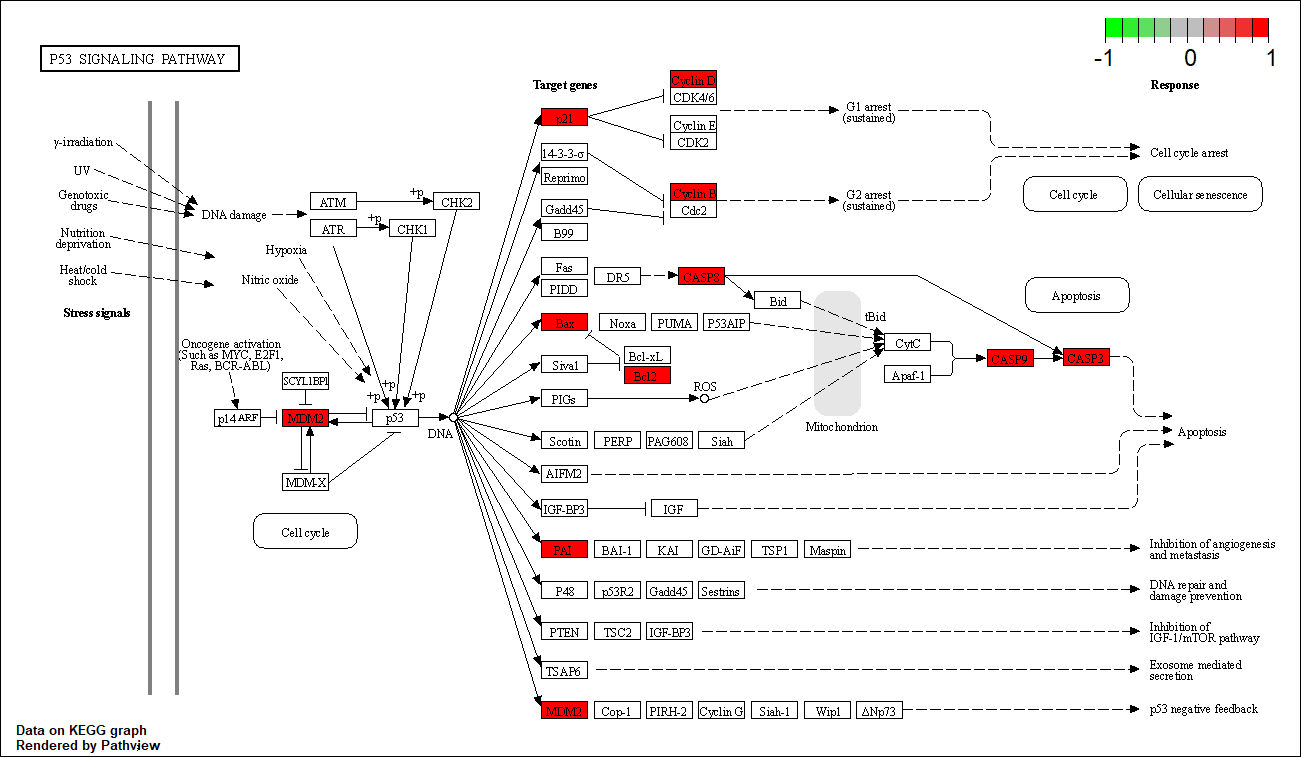

Supplement: S1 Data — (ZIP) [file pone.0274639.s001.zip › minimal data/GO+KEGG/R.KEGG/hsa04115.pathview.png]

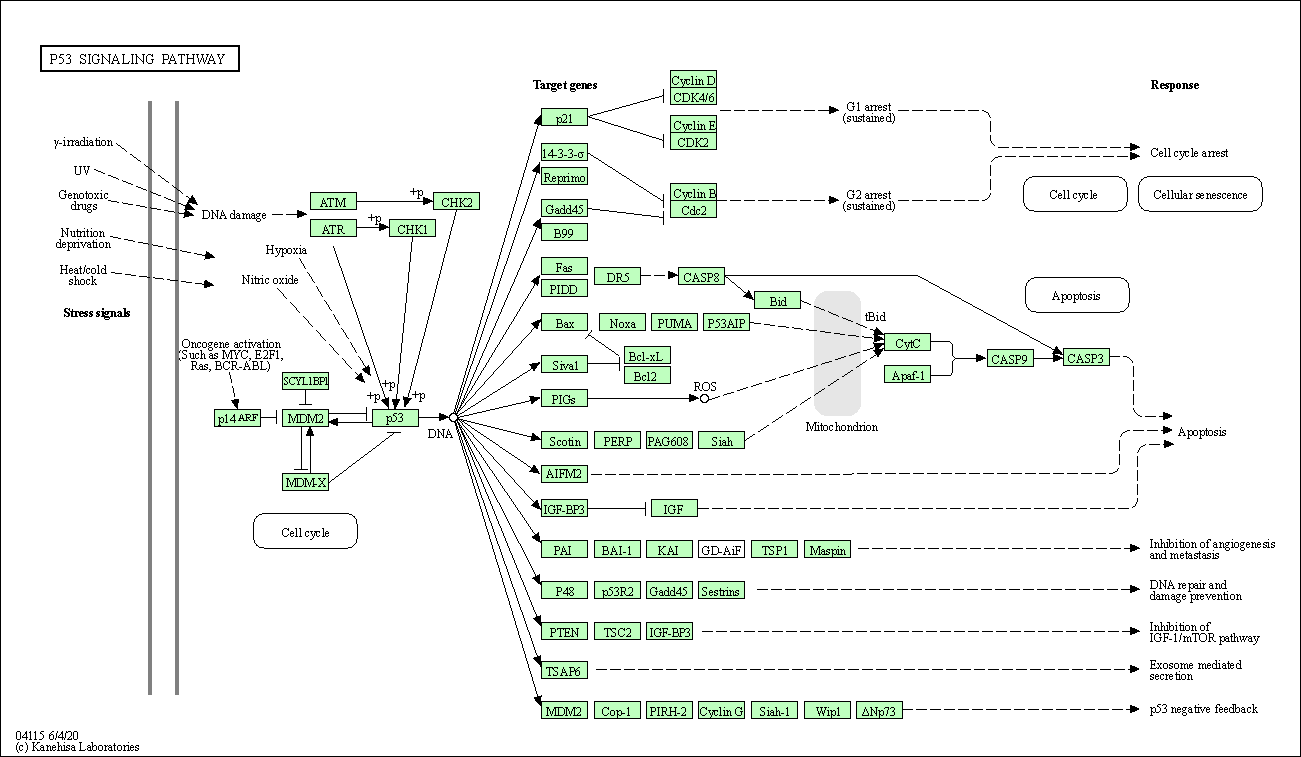

Supplement: S1 Data — (ZIP) [file pone.0274639.s001.zip › minimal data/GO+KEGG/R.KEGG/hsa04115.png]

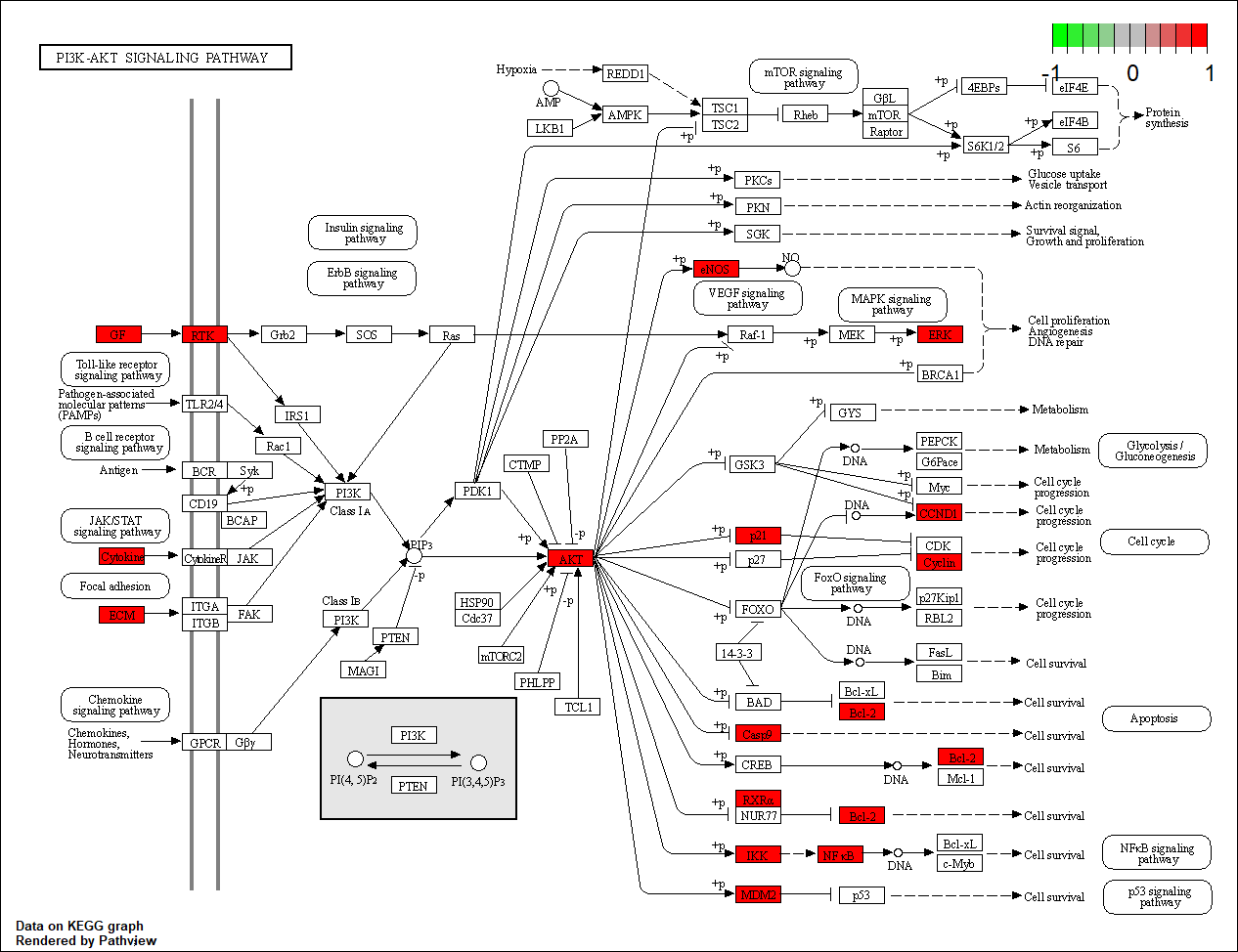

Supplement: S1 Data — (ZIP) [file pone.0274639.s001.zip › minimal data/GO+KEGG/R.KEGG/hsa04151.pathview.png]

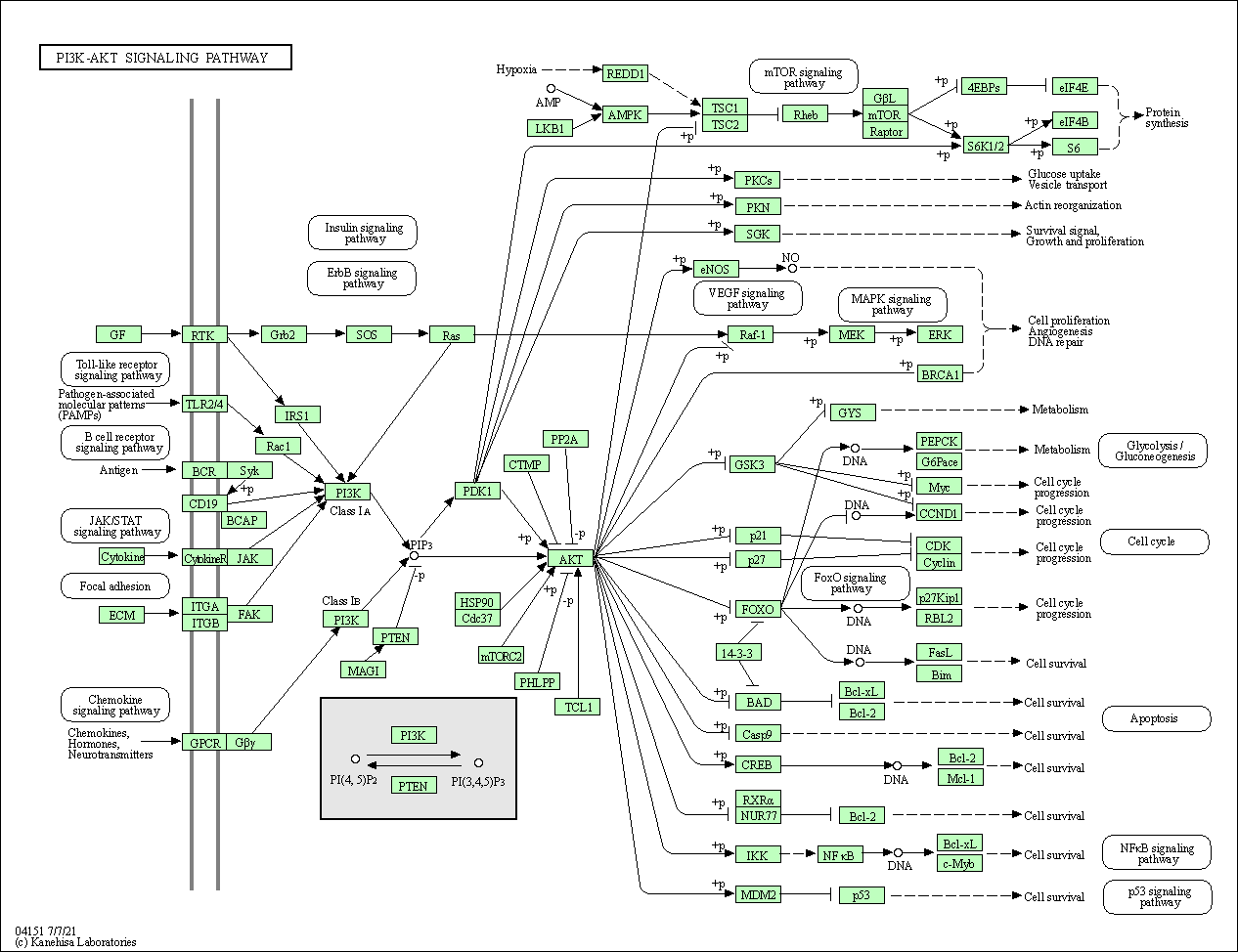

Supplement: S1 Data — (ZIP) [file pone.0274639.s001.zip › minimal data/GO+KEGG/R.KEGG/hsa04151.png]

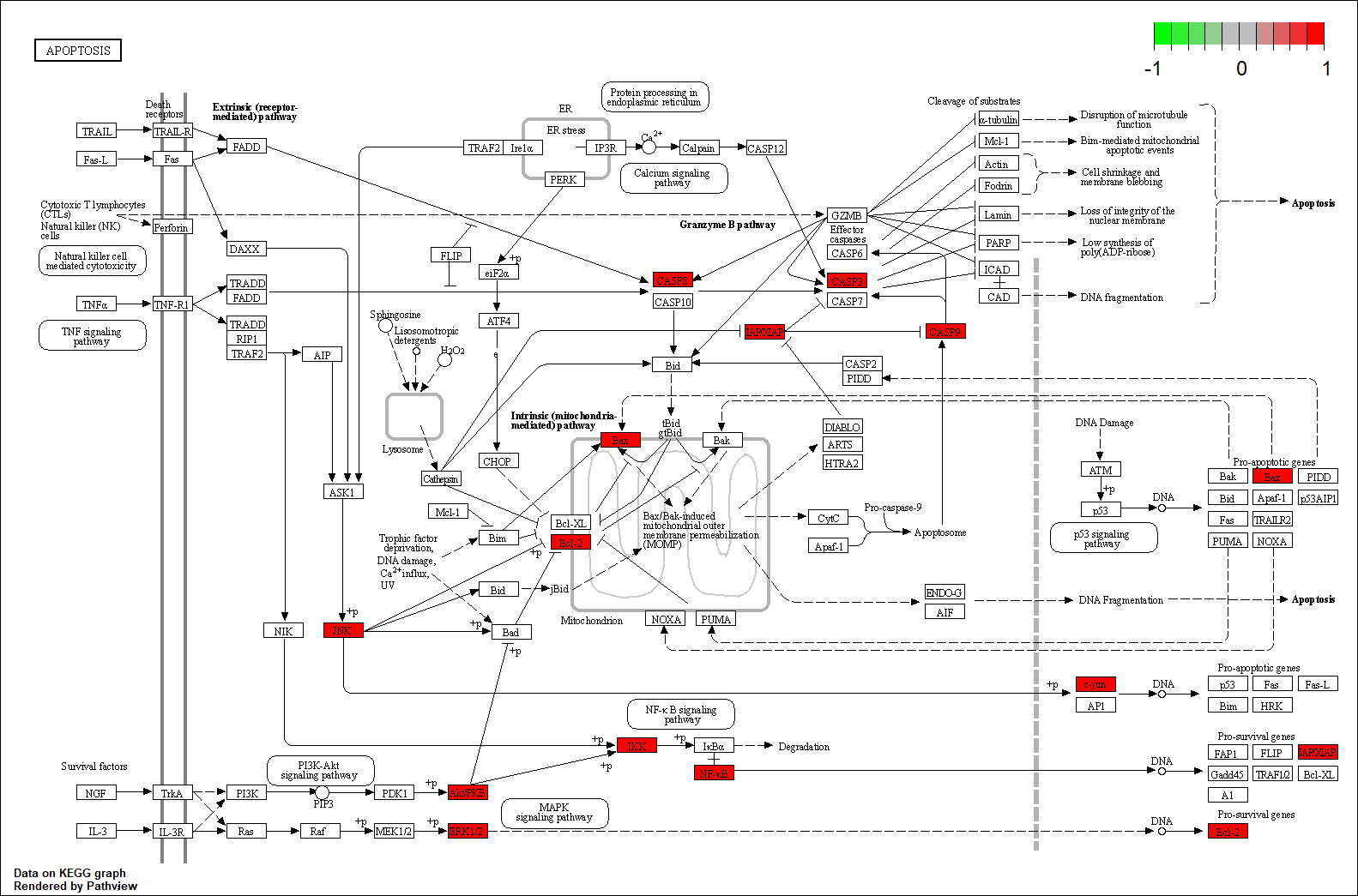

Supplement: S1 Data — (ZIP) [file pone.0274639.s001.zip › minimal data/GO+KEGG/R.KEGG/hsa04210.pathview.png]

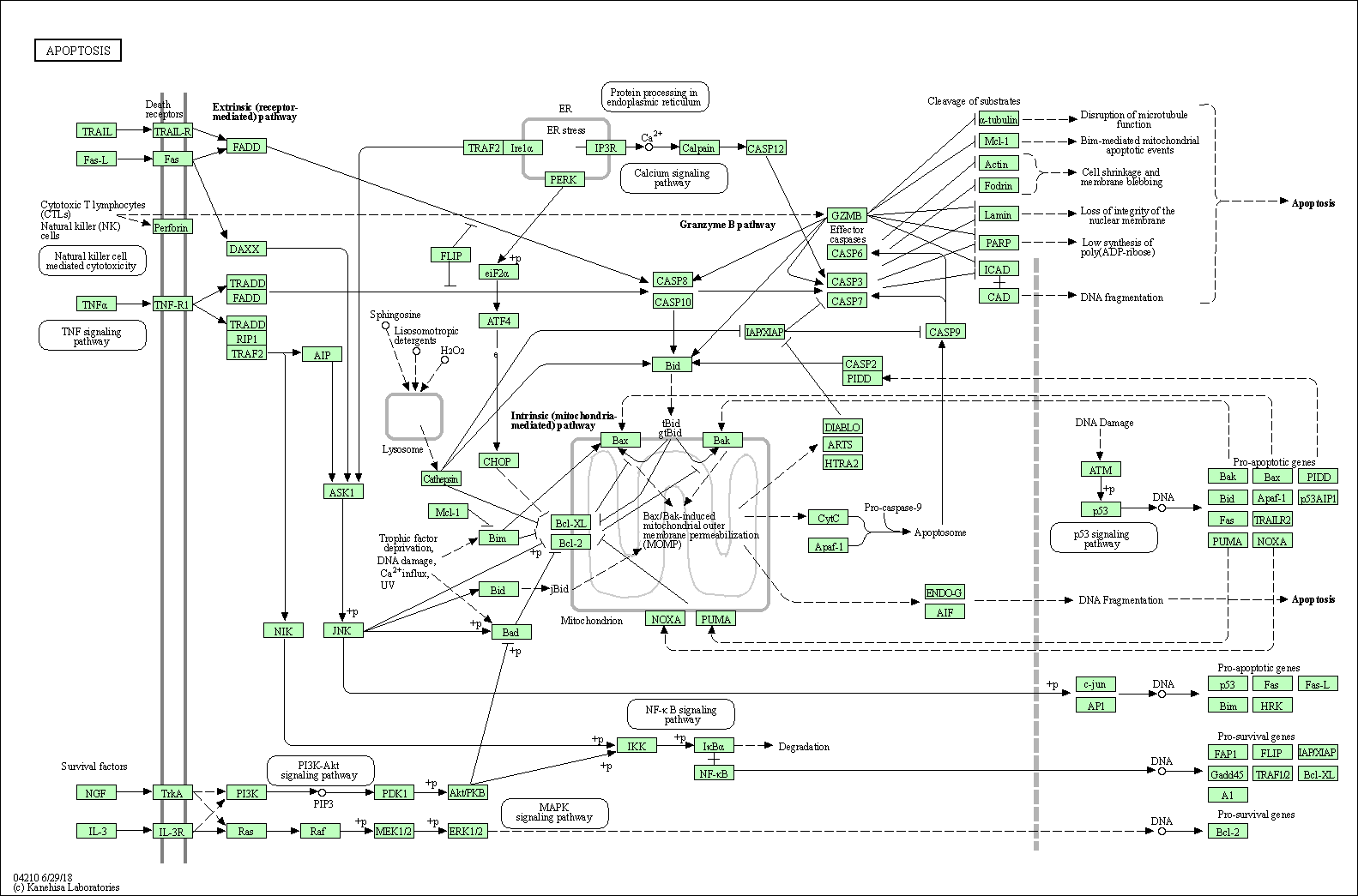

Supplement: S1 Data — (ZIP) [file pone.0274639.s001.zip › minimal data/GO+KEGG/R.KEGG/hsa04210.png]

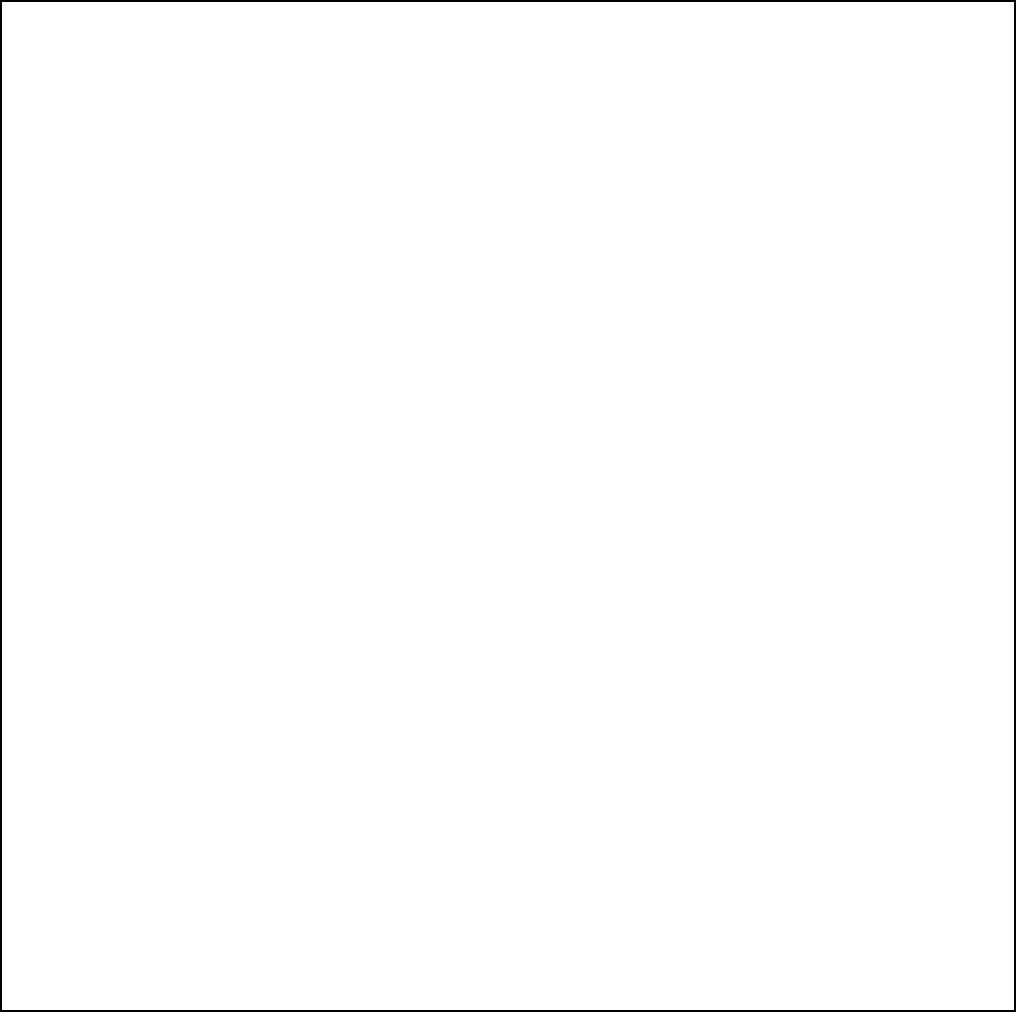

Supplement: S1 Data — (ZIP) [file pone.0274639.s001.zip › minimal data/GO+KEGG/R.KEGG/hsa04215.pathview.png]

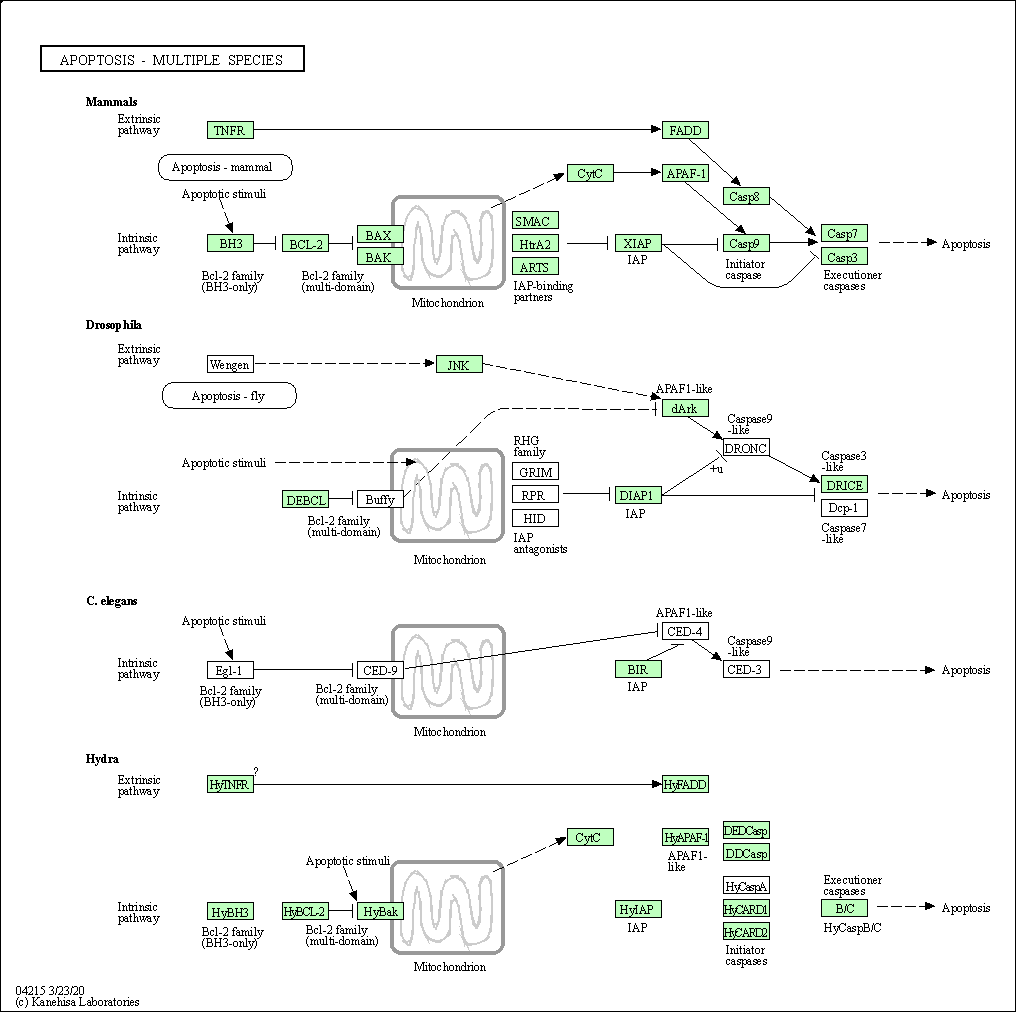

Supplement: S1 Data — (ZIP) [file pone.0274639.s001.zip › minimal data/GO+KEGG/R.KEGG/hsa04215.png]

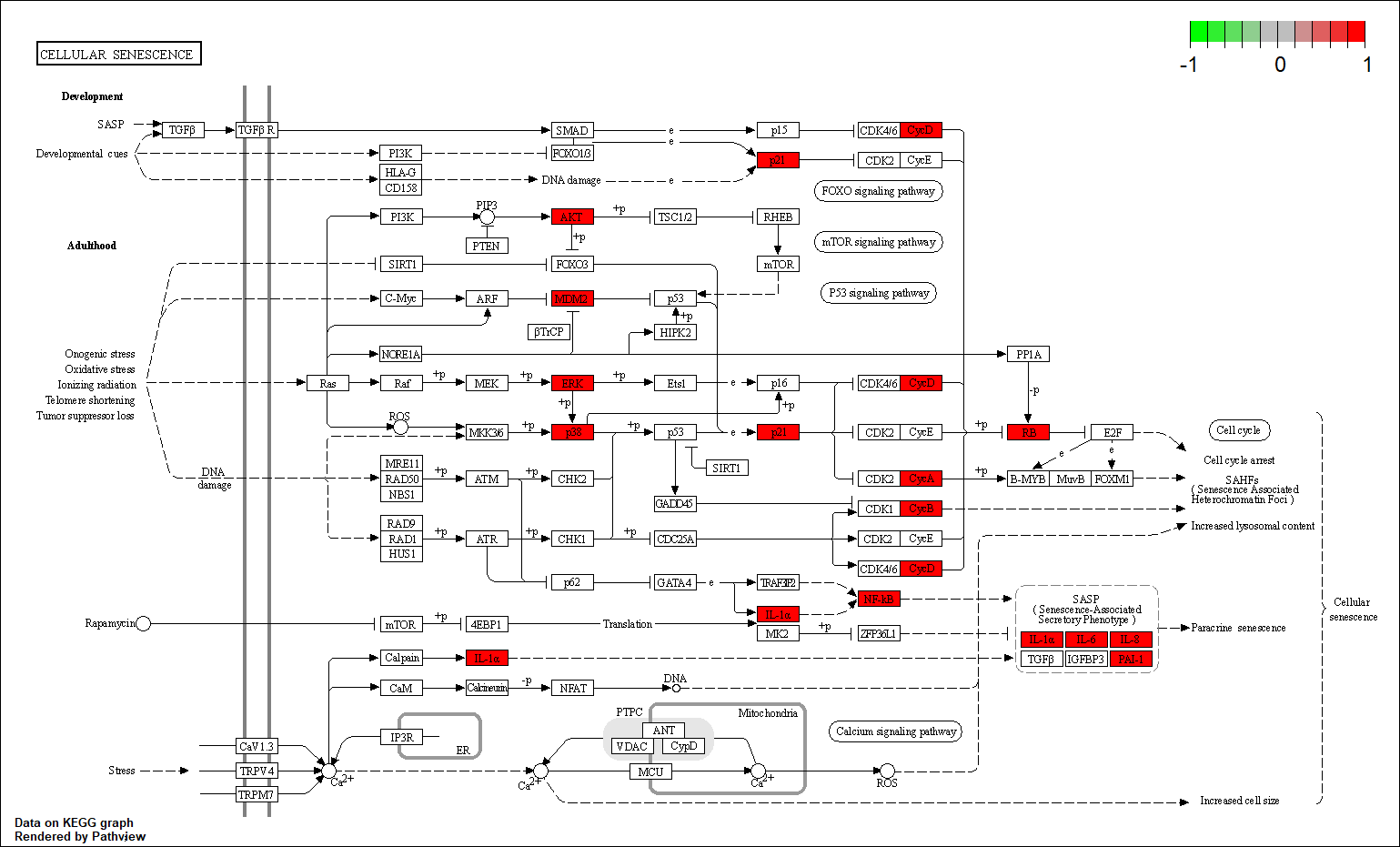

Supplement: S1 Data — (ZIP) [file pone.0274639.s001.zip › minimal data/GO+KEGG/R.KEGG/hsa04218.pathview.png]

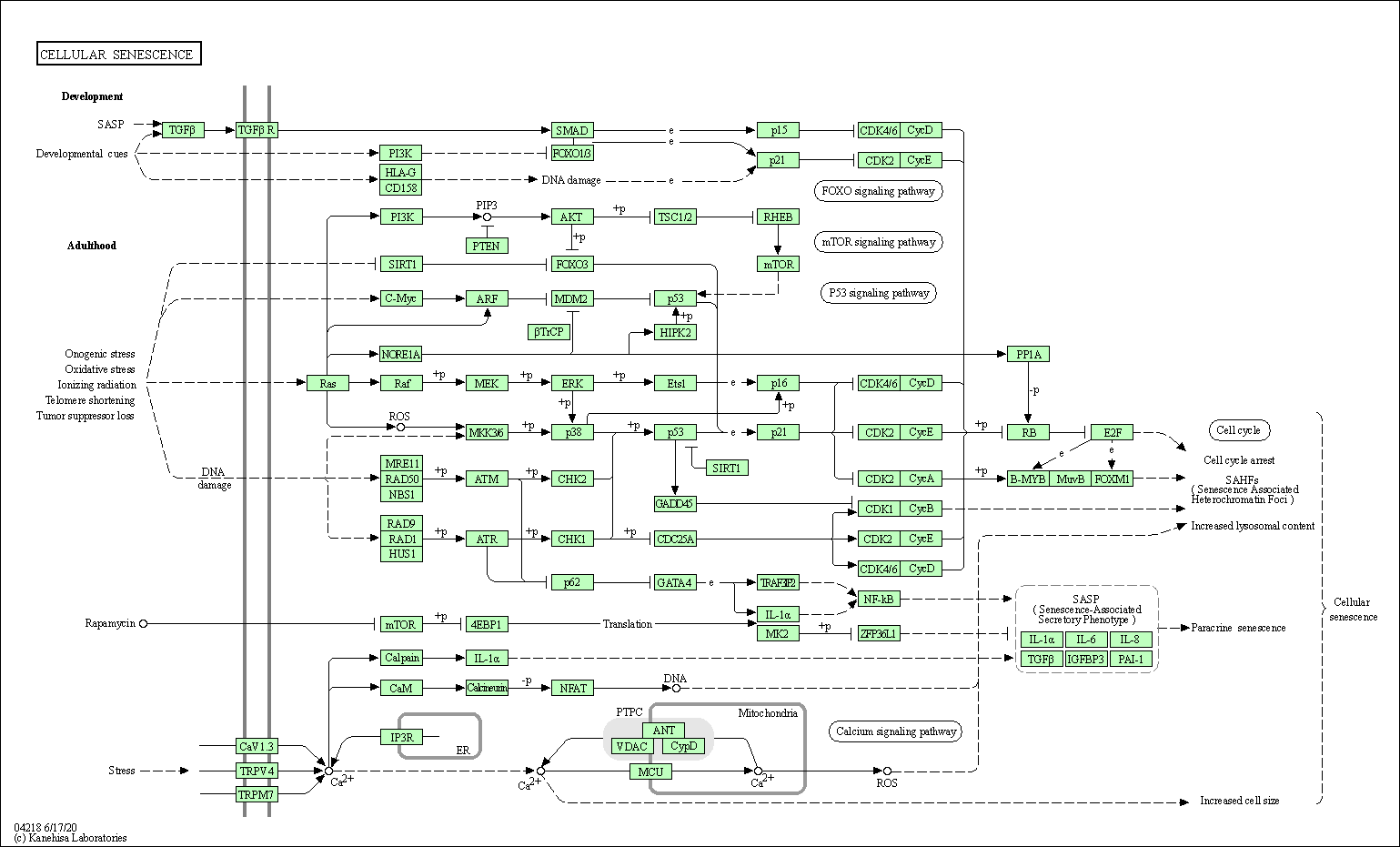

Supplement: S1 Data — (ZIP) [file pone.0274639.s001.zip › minimal data/GO+KEGG/R.KEGG/hsa04218.png]

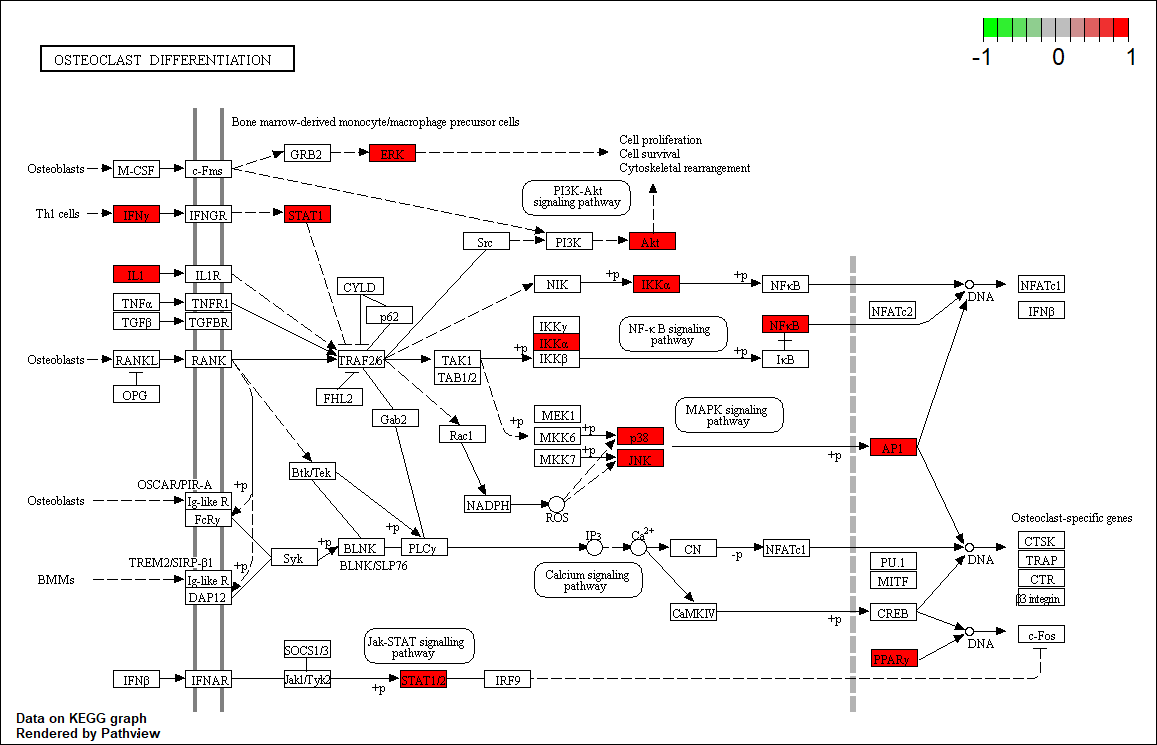

Supplement: S1 Data — (ZIP) [file pone.0274639.s001.zip › minimal data/GO+KEGG/R.KEGG/hsa04380.pathview.png]

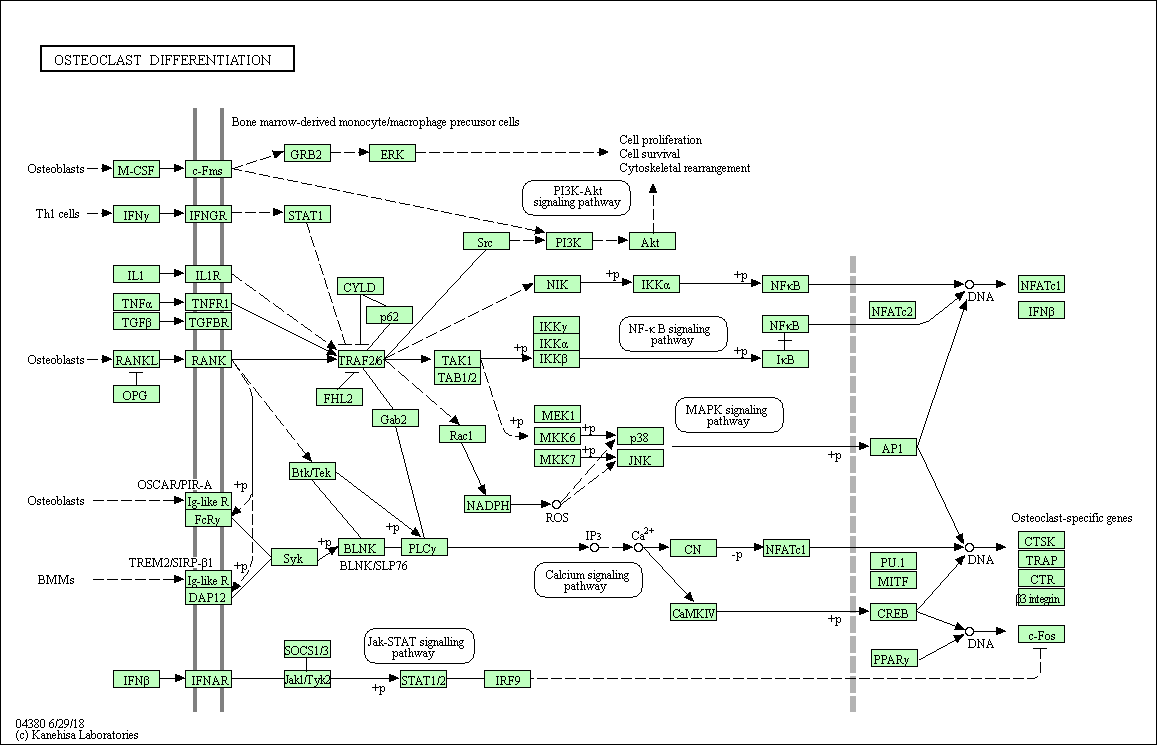

Supplement: S1 Data — (ZIP) [file pone.0274639.s001.zip › minimal data/GO+KEGG/R.KEGG/hsa04380.png]

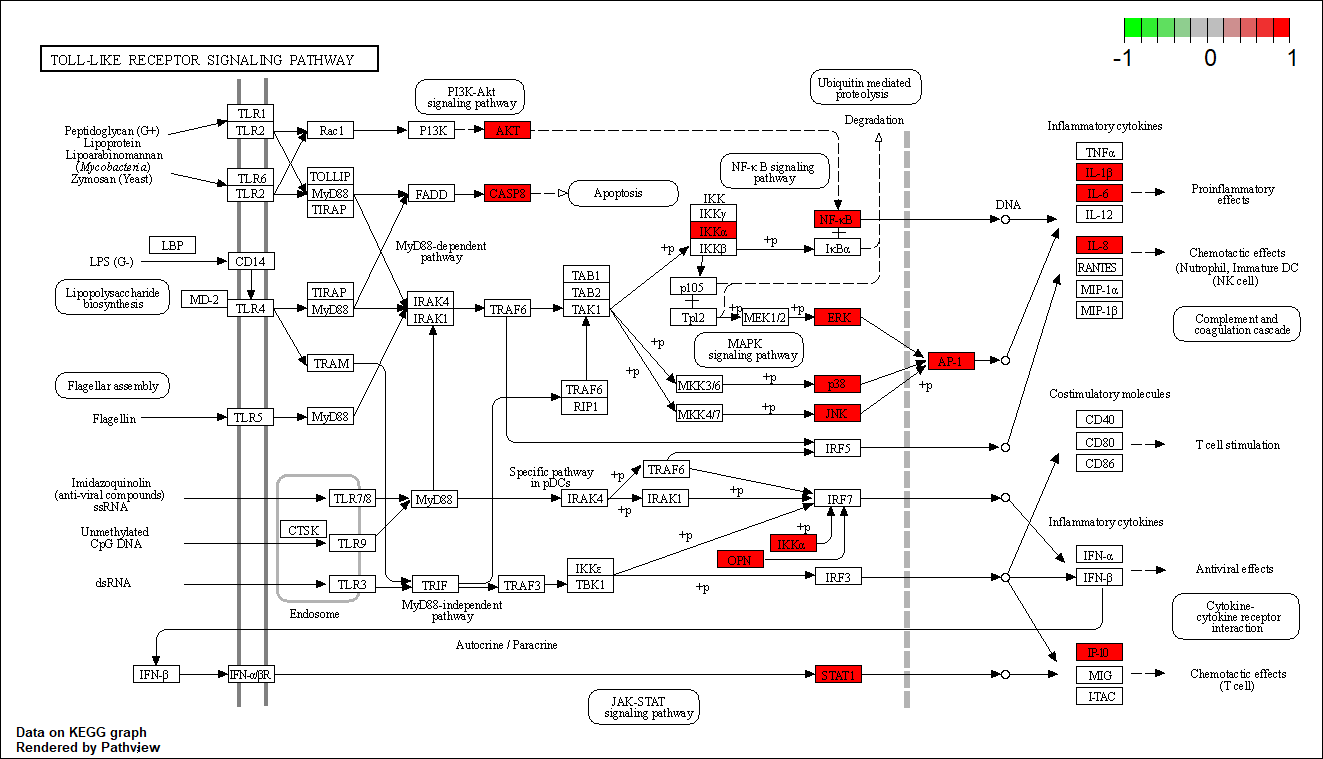

Supplement: S1 Data — (ZIP) [file pone.0274639.s001.zip › minimal data/GO+KEGG/R.KEGG/hsa04620.pathview.png]

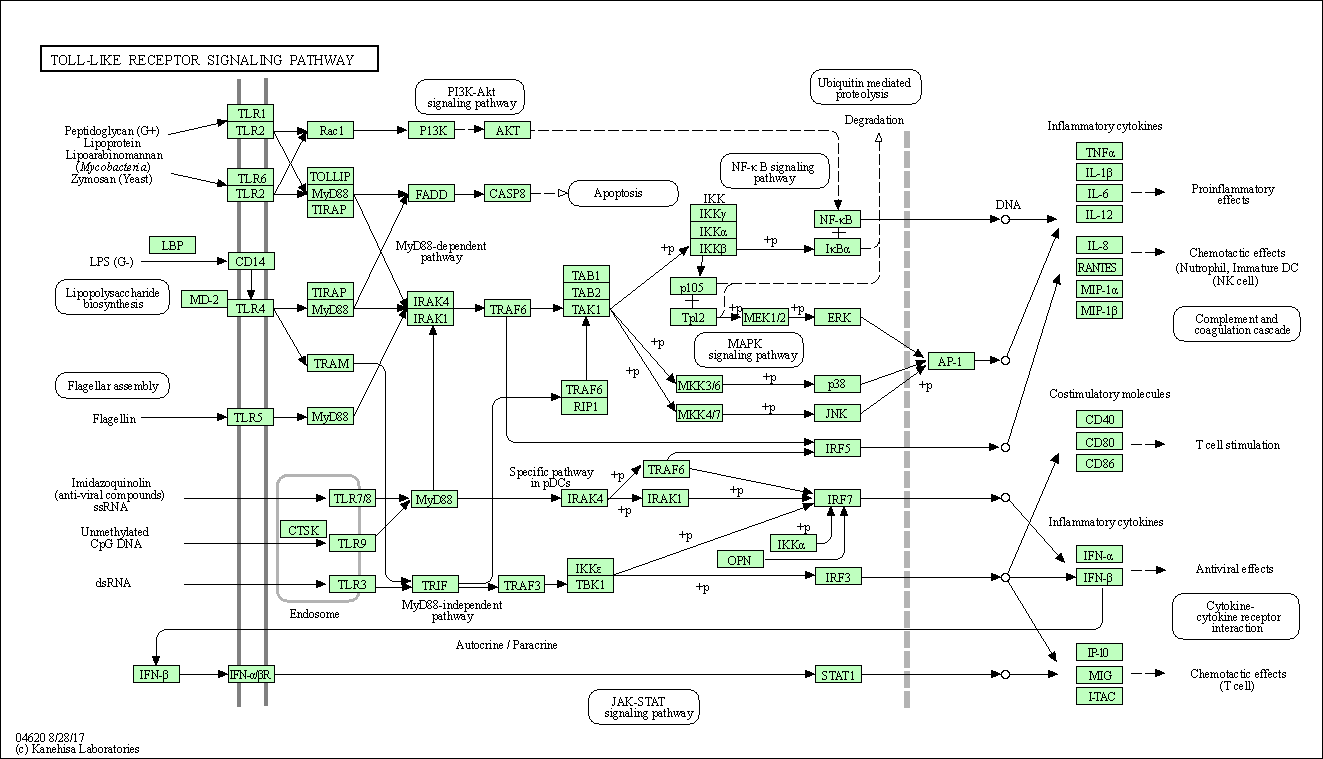

Supplement: S1 Data — (ZIP) [file pone.0274639.s001.zip › minimal data/GO+KEGG/R.KEGG/hsa04620.png]

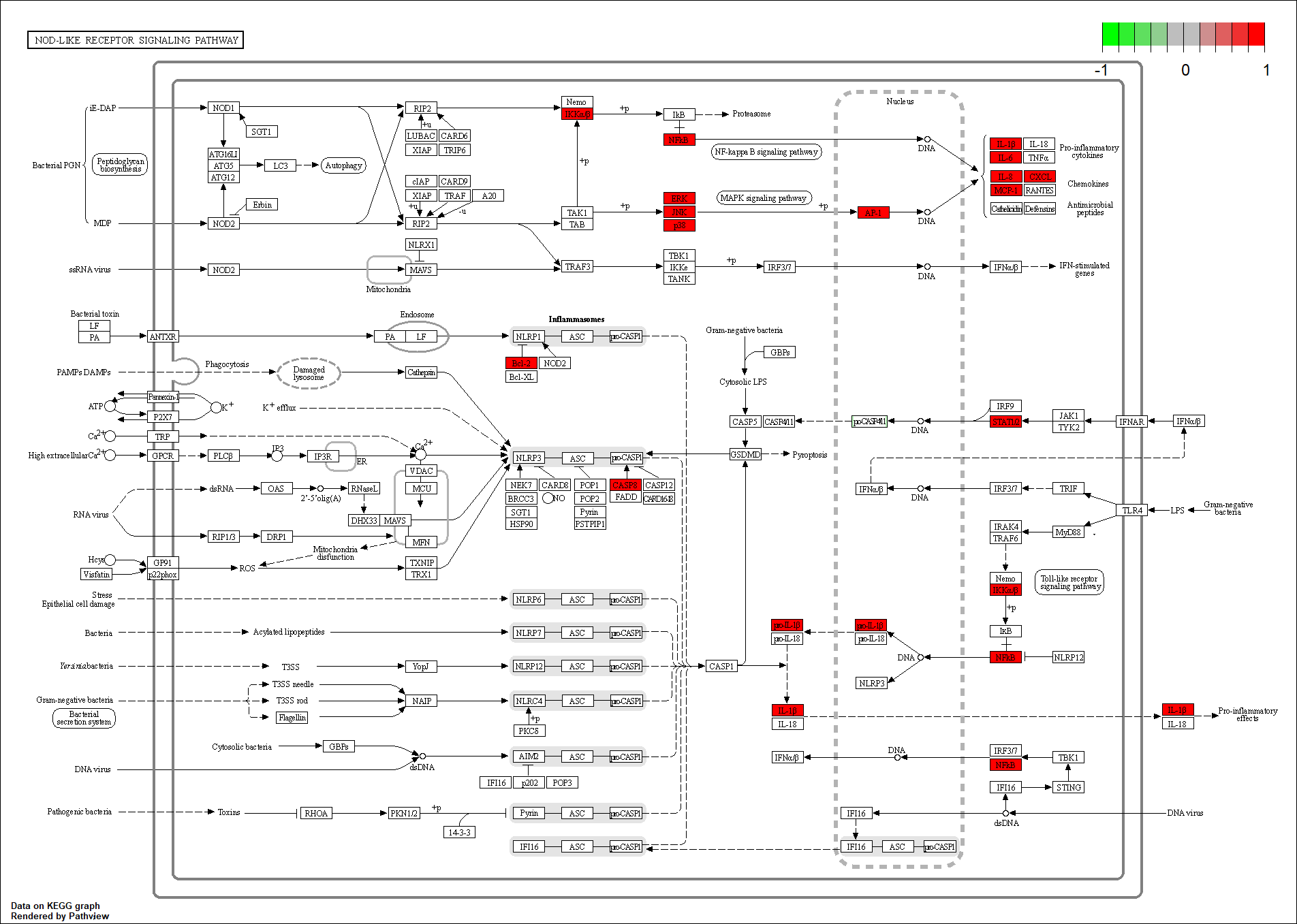

Supplement: S1 Data — (ZIP) [file pone.0274639.s001.zip › minimal data/GO+KEGG/R.KEGG/hsa04621.pathview.png]

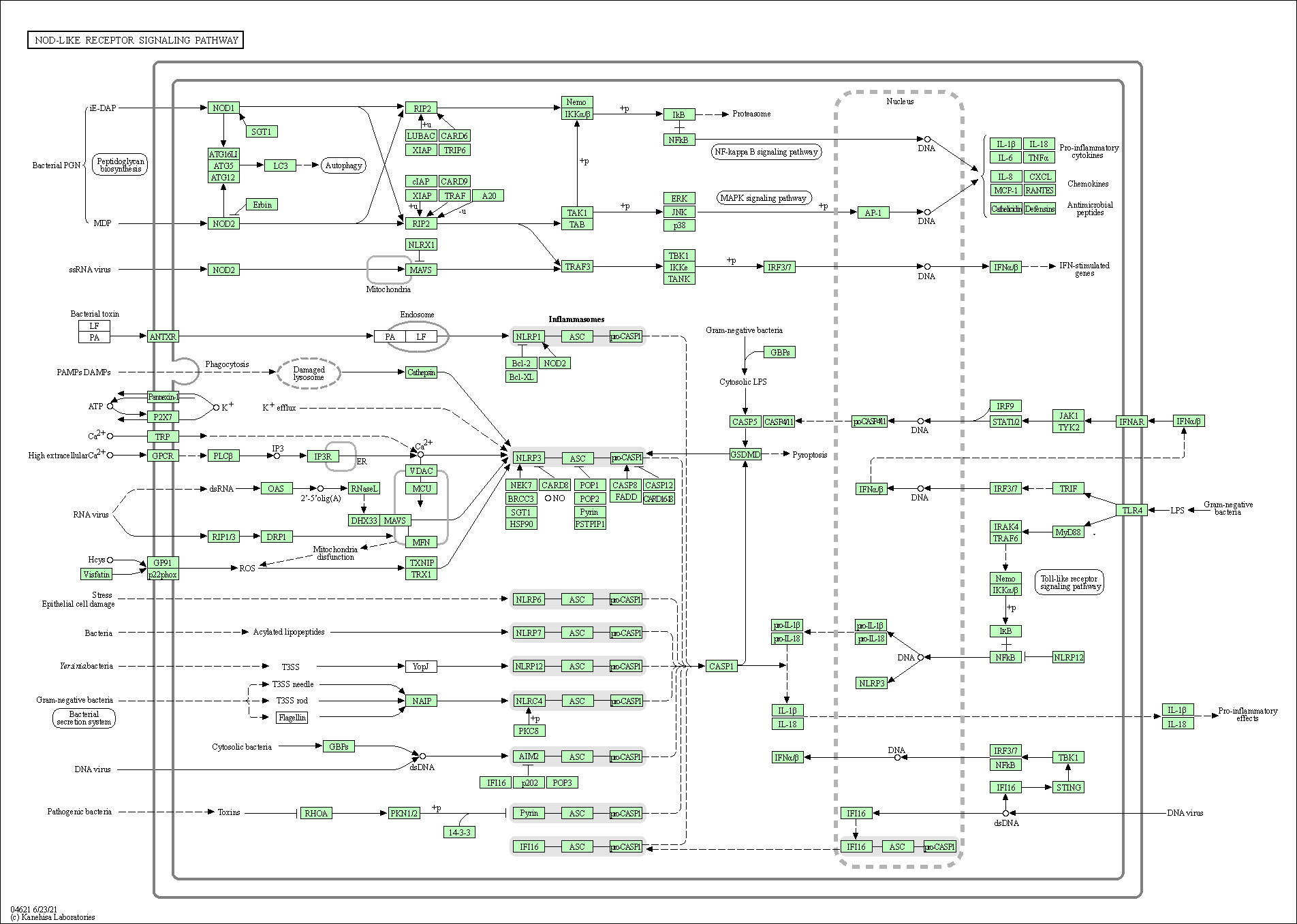

Supplement: S1 Data — (ZIP) [file pone.0274639.s001.zip › minimal data/GO+KEGG/R.KEGG/hsa04621.png]

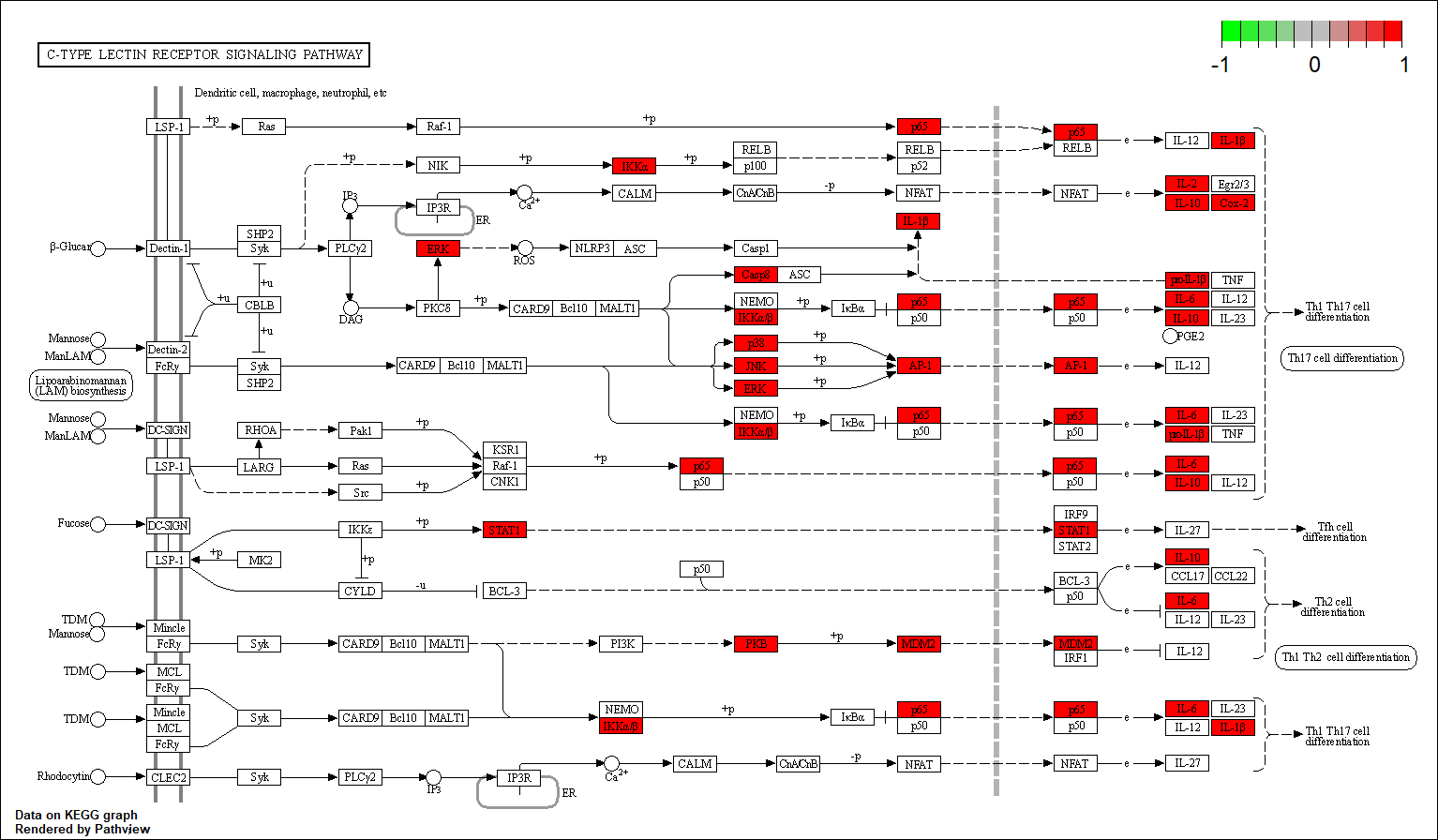

Supplement: S1 Data — (ZIP) [file pone.0274639.s001.zip › minimal data/GO+KEGG/R.KEGG/hsa04625.pathview.png]

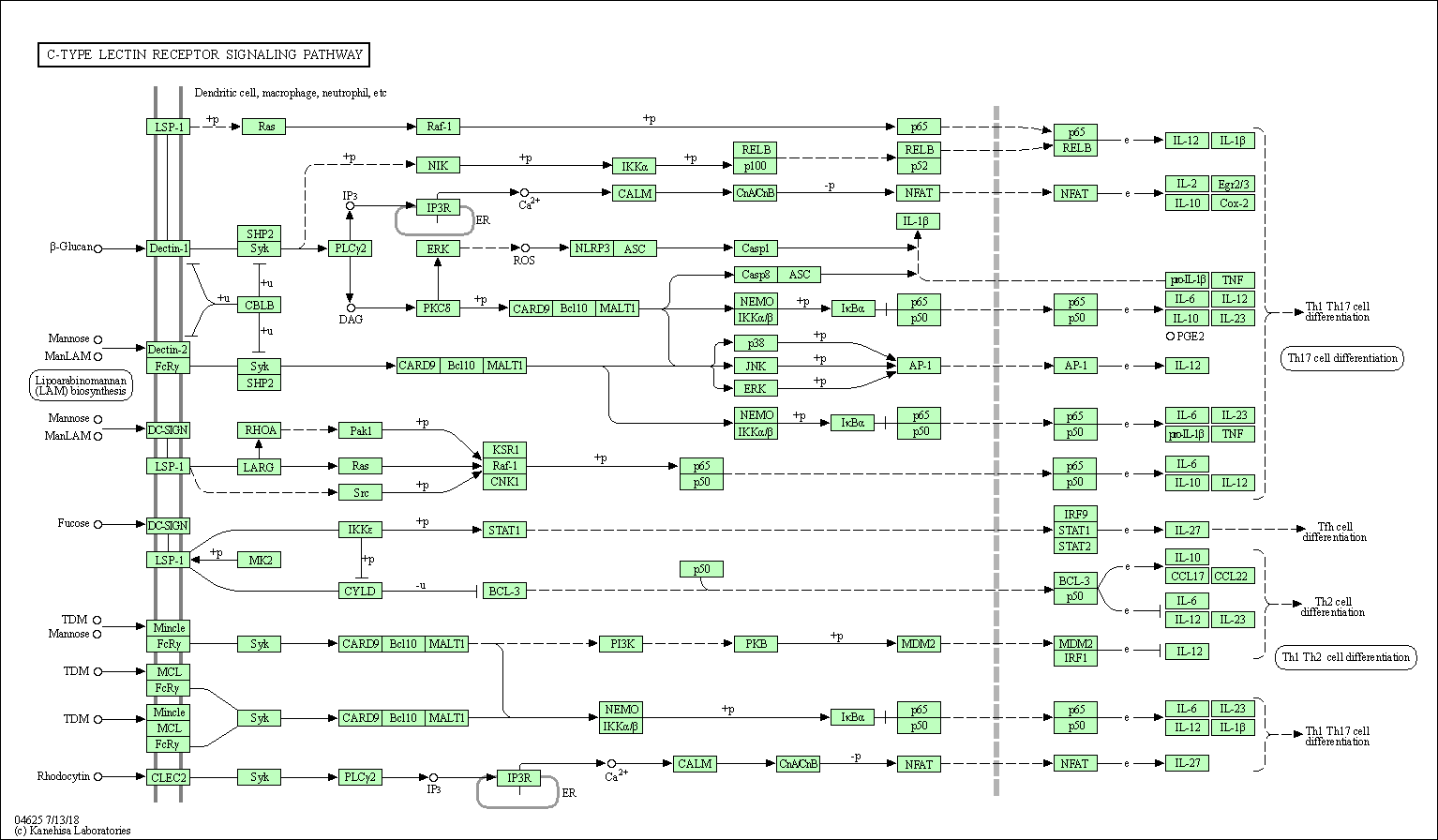

Supplement: S1 Data — (ZIP) [file pone.0274639.s001.zip › minimal data/GO+KEGG/R.KEGG/hsa04625.png]

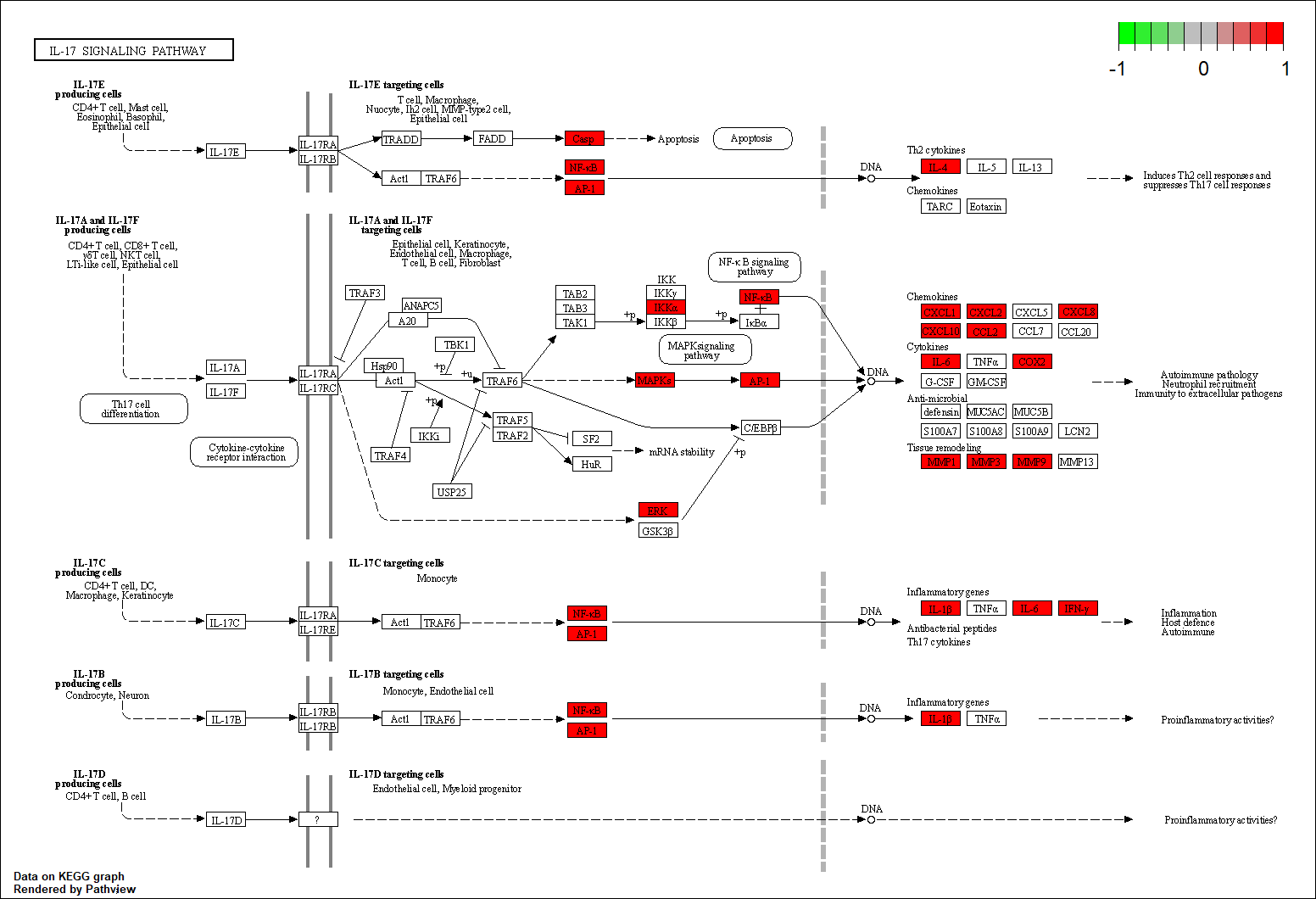

Supplement: S1 Data — (ZIP) [file pone.0274639.s001.zip › minimal data/GO+KEGG/R.KEGG/hsa04657.pathview.png]

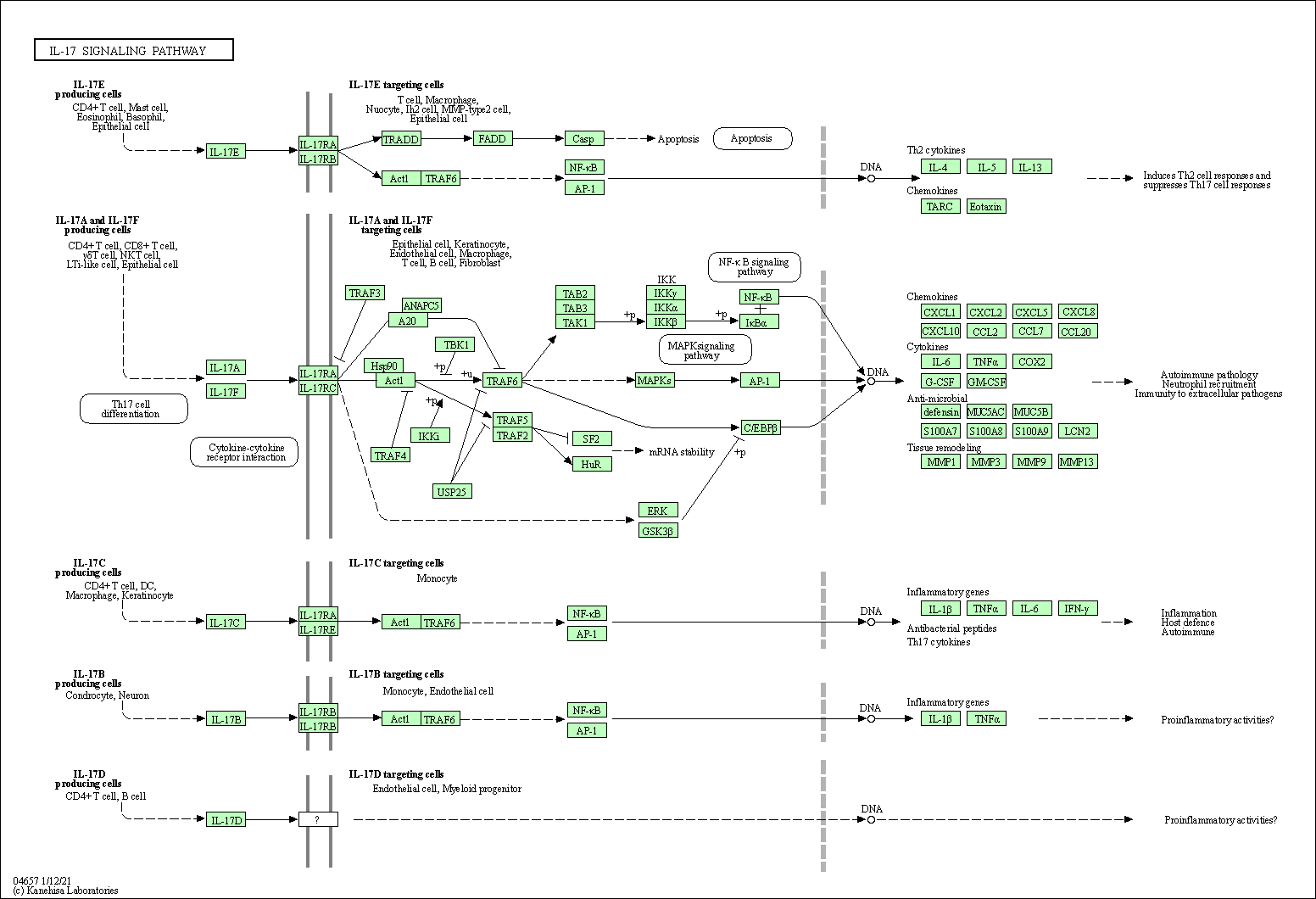

Supplement: S1 Data — (ZIP) [file pone.0274639.s001.zip › minimal data/GO+KEGG/R.KEGG/hsa04657.png]

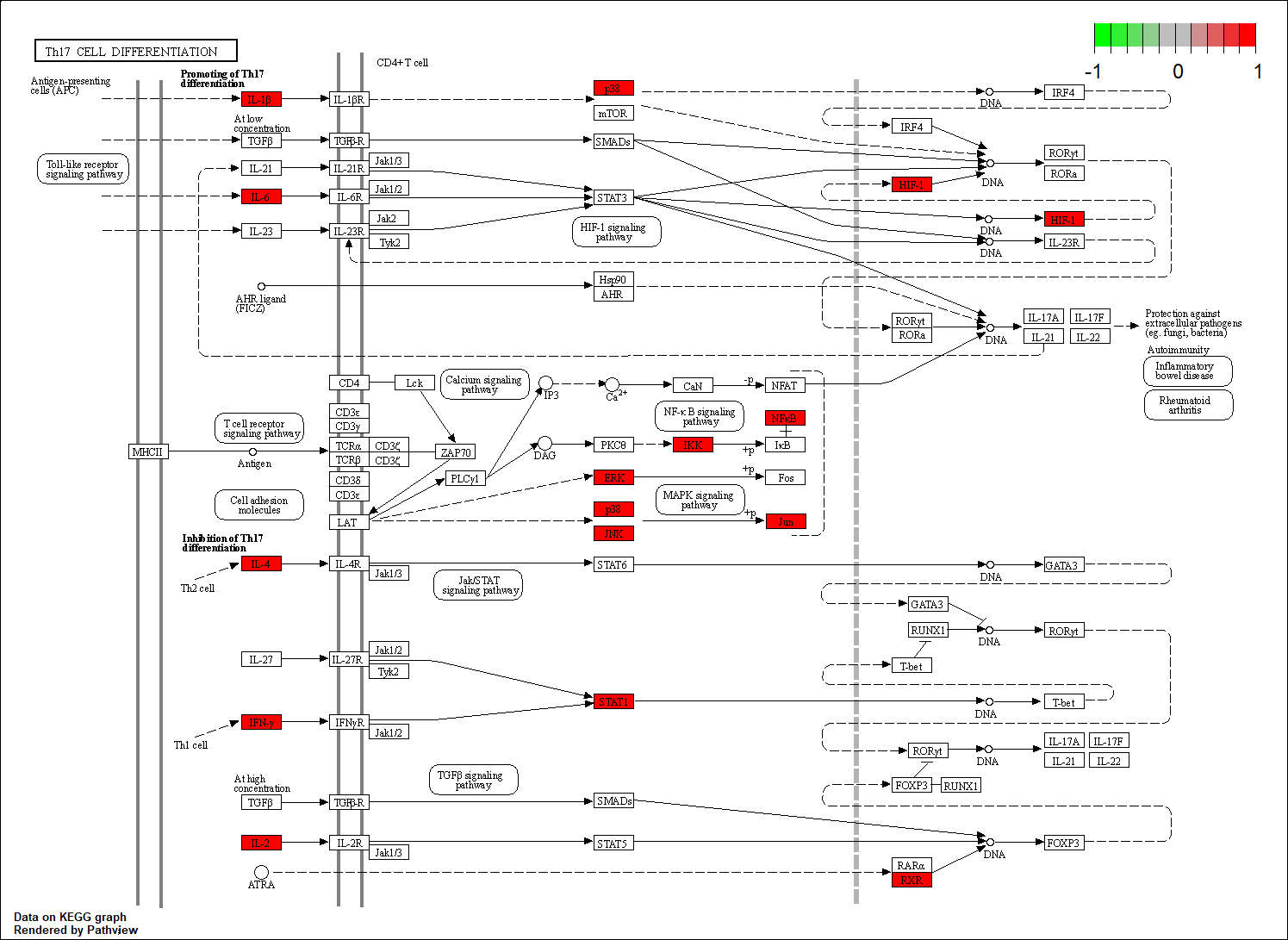

Supplement: S1 Data — (ZIP) [file pone.0274639.s001.zip › minimal data/GO+KEGG/R.KEGG/hsa04659.pathview.png]

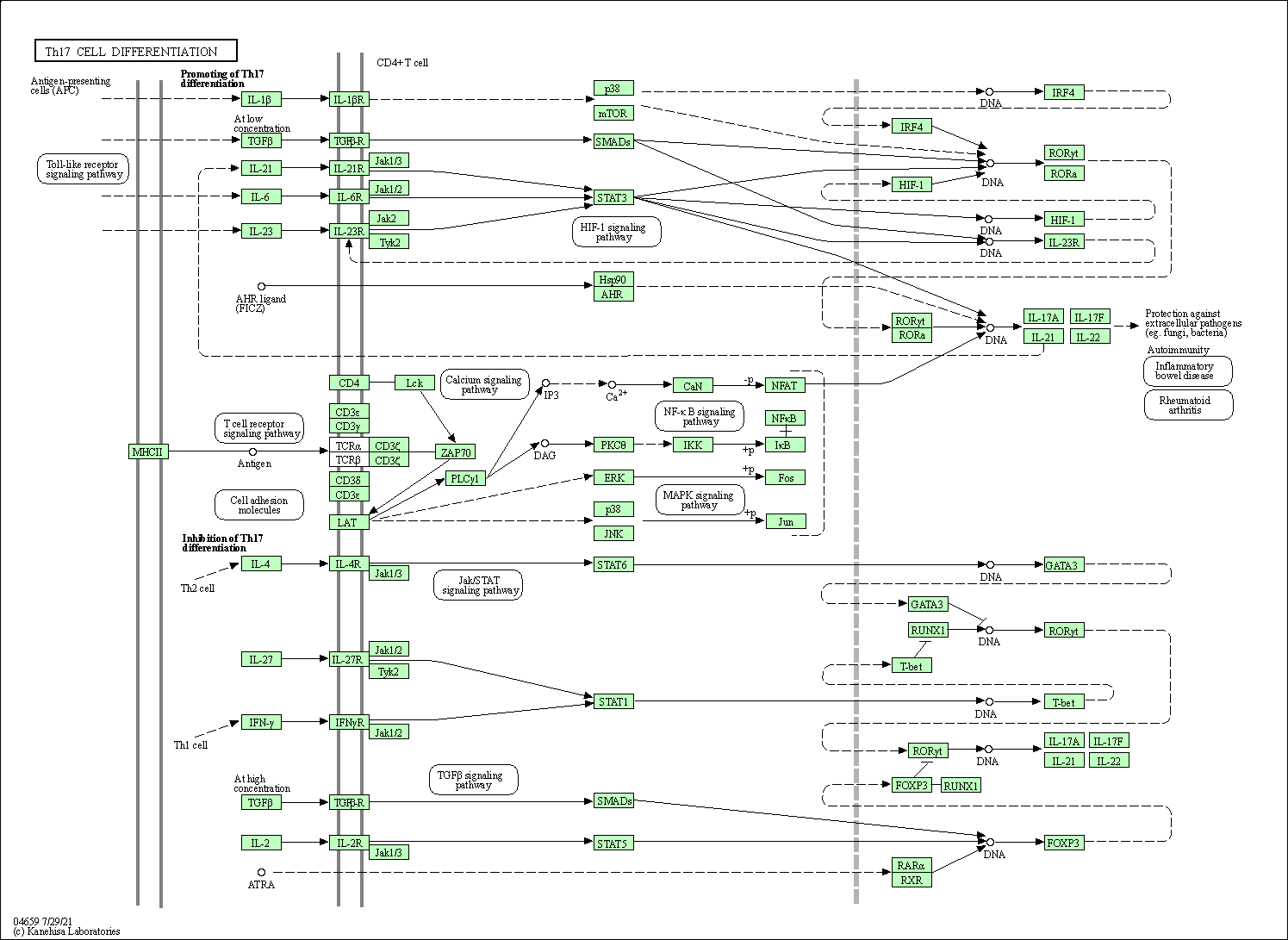

Supplement: S1 Data — (ZIP) [file pone.0274639.s001.zip › minimal data/GO+KEGG/R.KEGG/hsa04659.png]

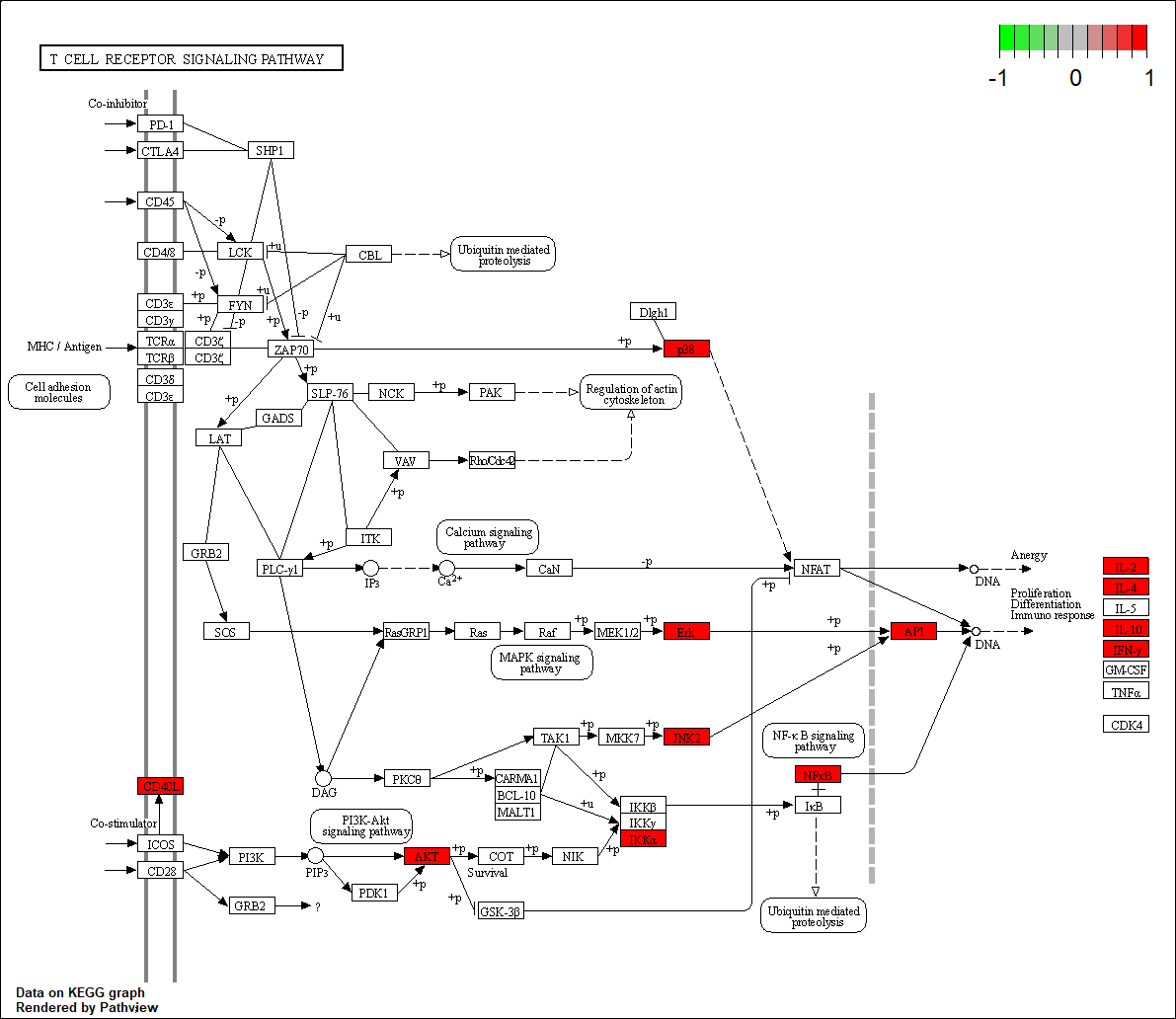

Supplement: S1 Data — (ZIP) [file pone.0274639.s001.zip › minimal data/GO+KEGG/R.KEGG/hsa04660.pathview.png]

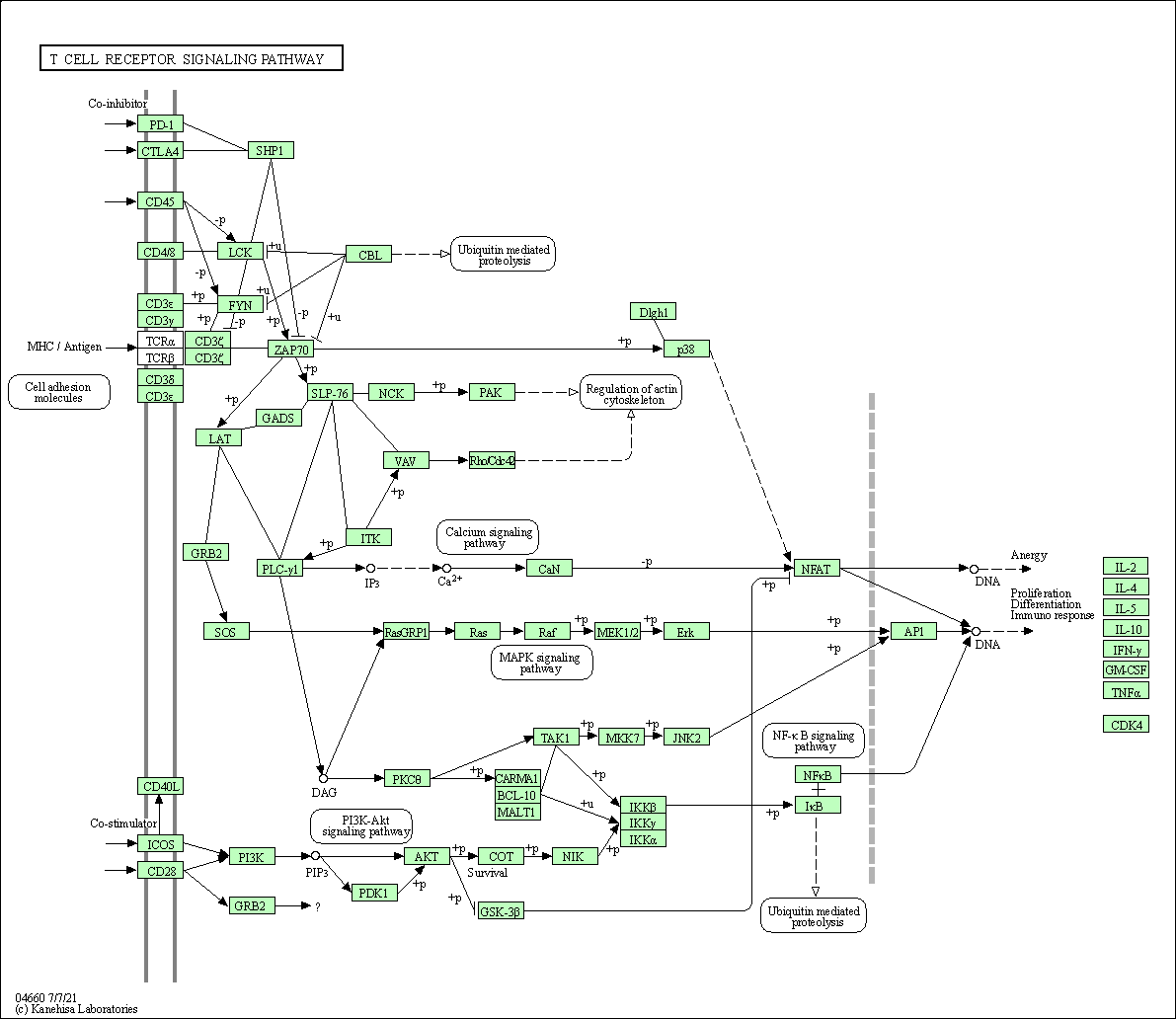

Supplement: S1 Data — (ZIP) [file pone.0274639.s001.zip › minimal data/GO+KEGG/R.KEGG/hsa04660.png]

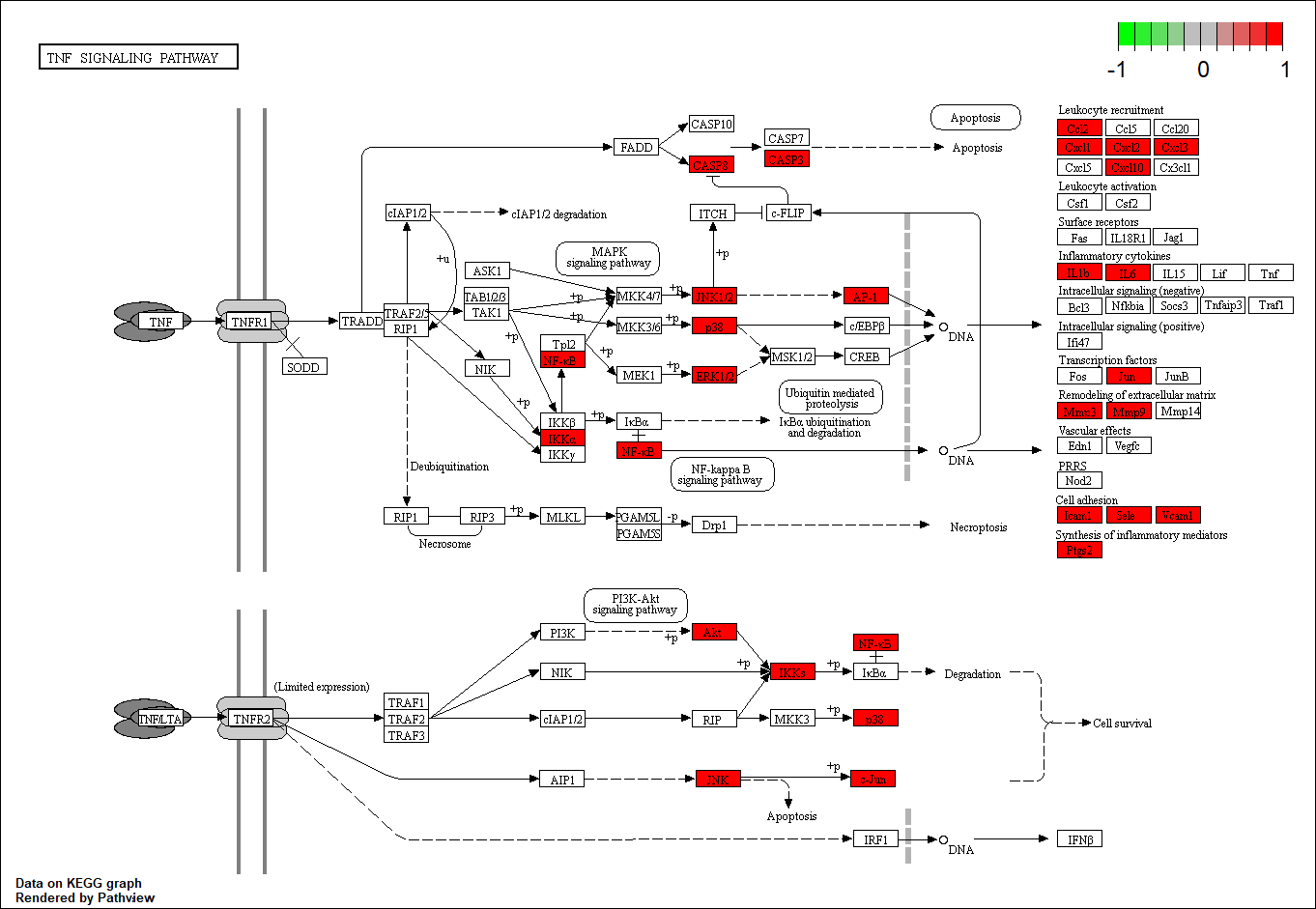

Supplement: S1 Data — (ZIP) [file pone.0274639.s001.zip › minimal data/GO+KEGG/R.KEGG/hsa04668.pathview.png]

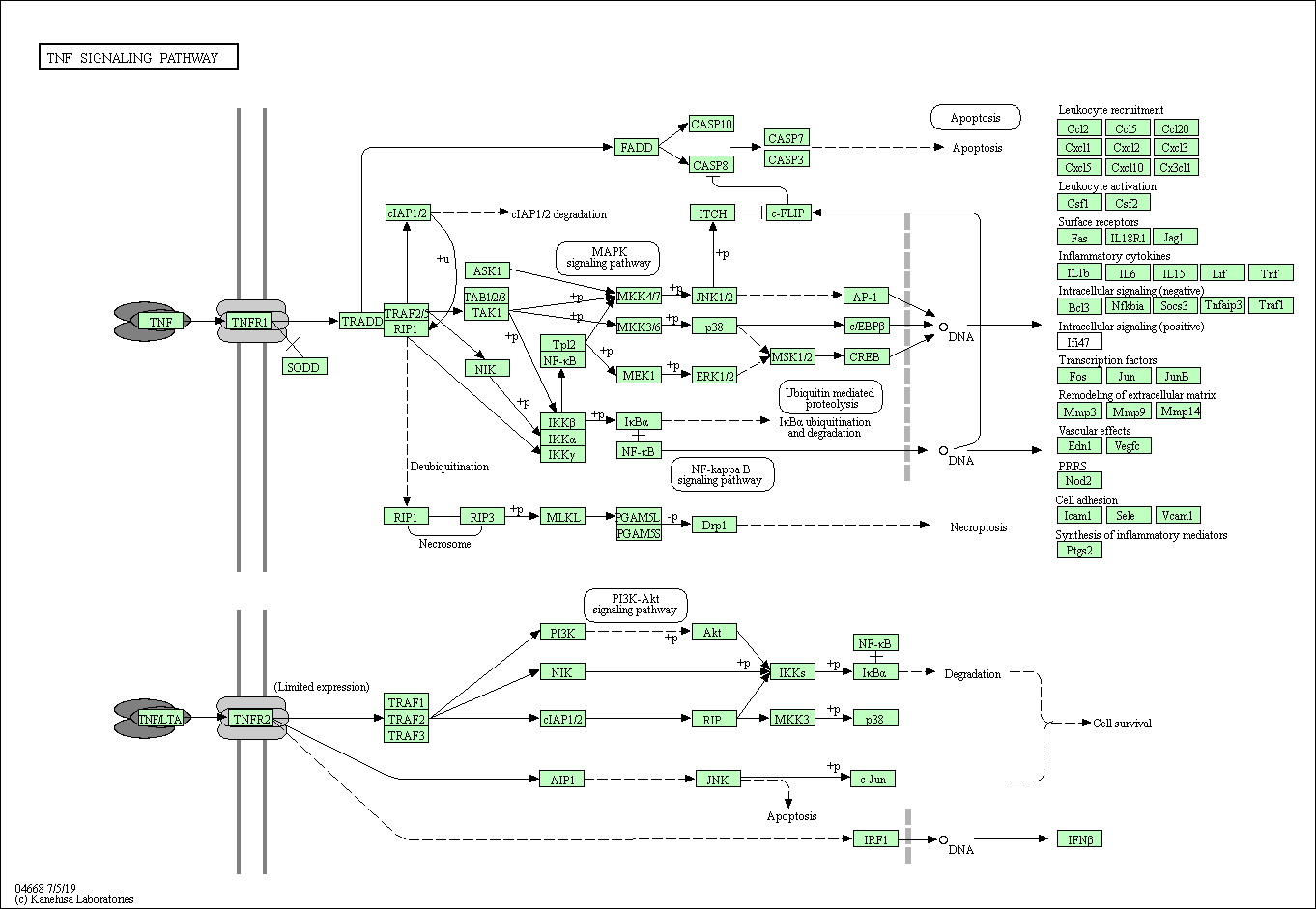

Supplement: S1 Data — (ZIP) [file pone.0274639.s001.zip › minimal data/GO+KEGG/R.KEGG/hsa04668.png]

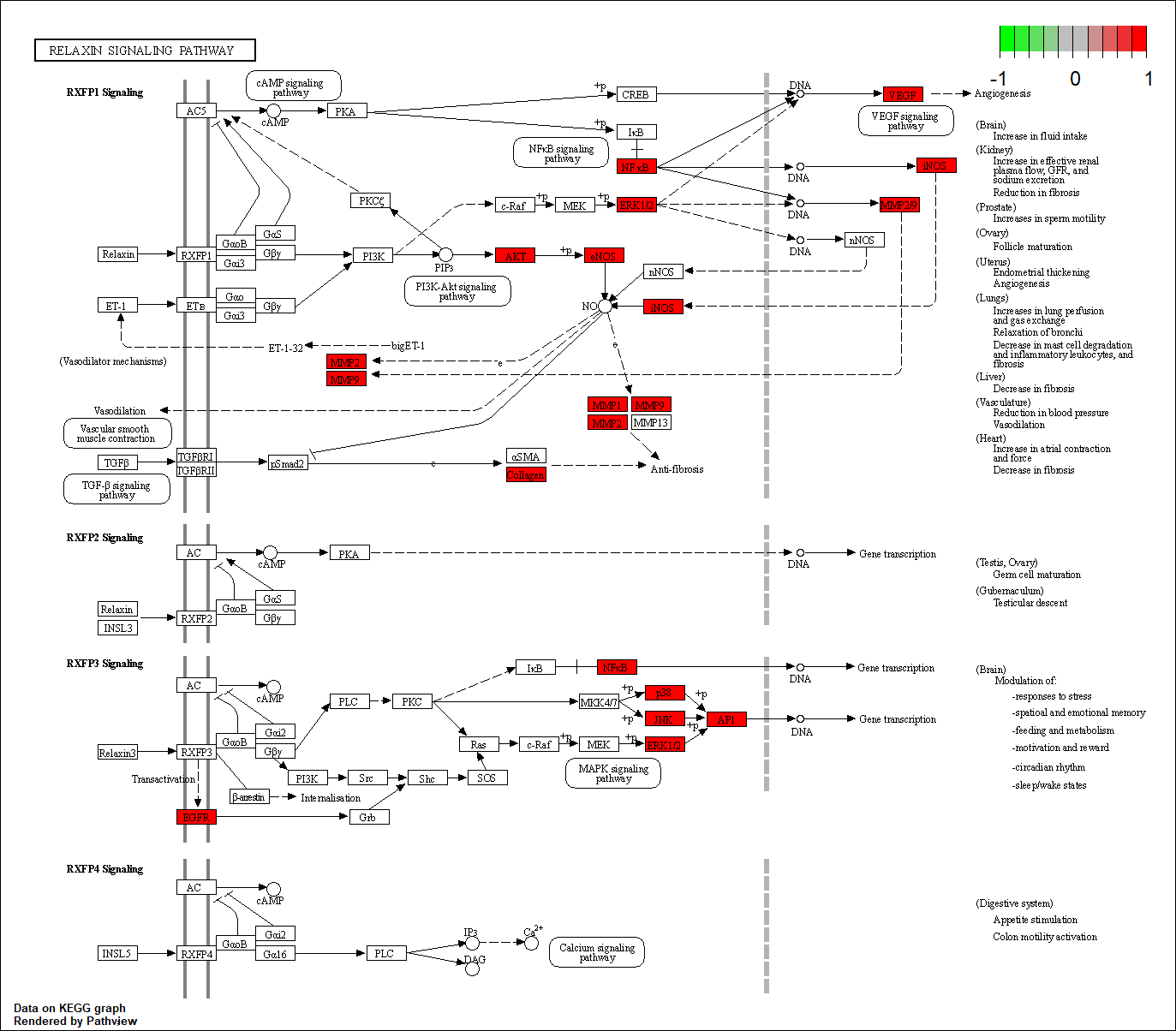

Supplement: S1 Data — (ZIP) [file pone.0274639.s001.zip › minimal data/GO+KEGG/R.KEGG/hsa04926.pathview.png]

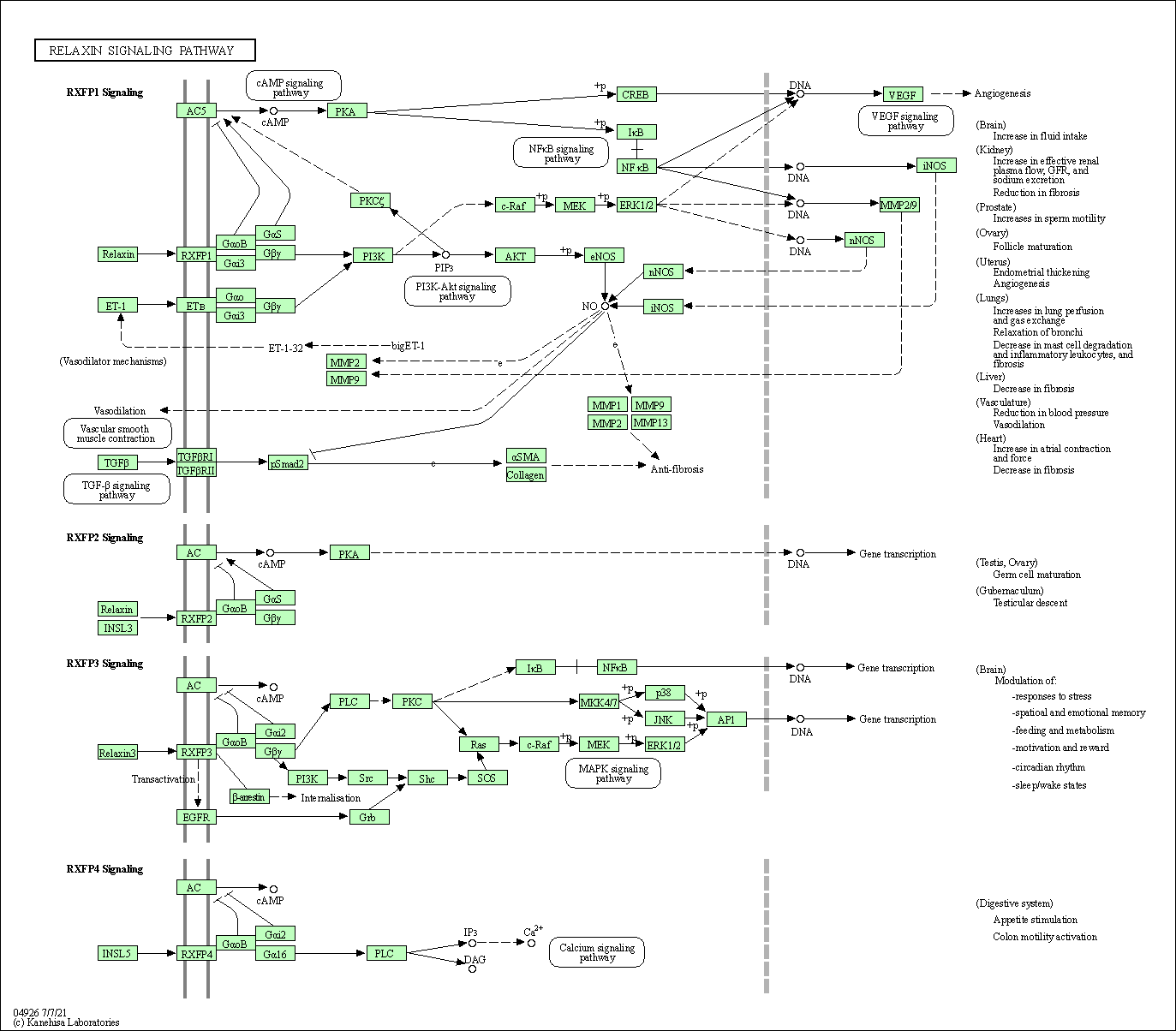

Supplement: S1 Data — (ZIP) [file pone.0274639.s001.zip › minimal data/GO+KEGG/R.KEGG/hsa04926.png]

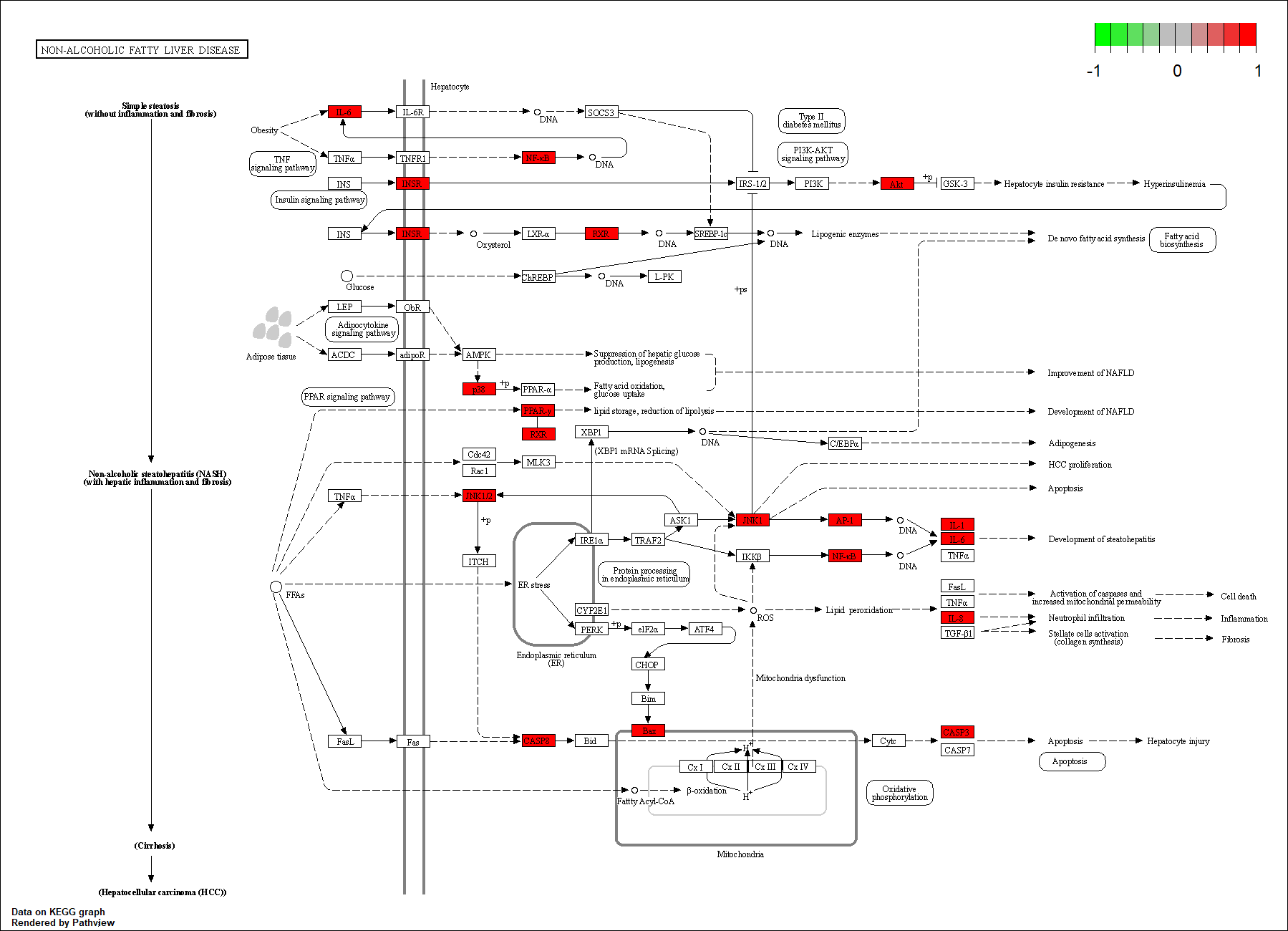

Supplement: S1 Data — (ZIP) [file pone.0274639.s001.zip › minimal data/GO+KEGG/R.KEGG/hsa04932.pathview.png]

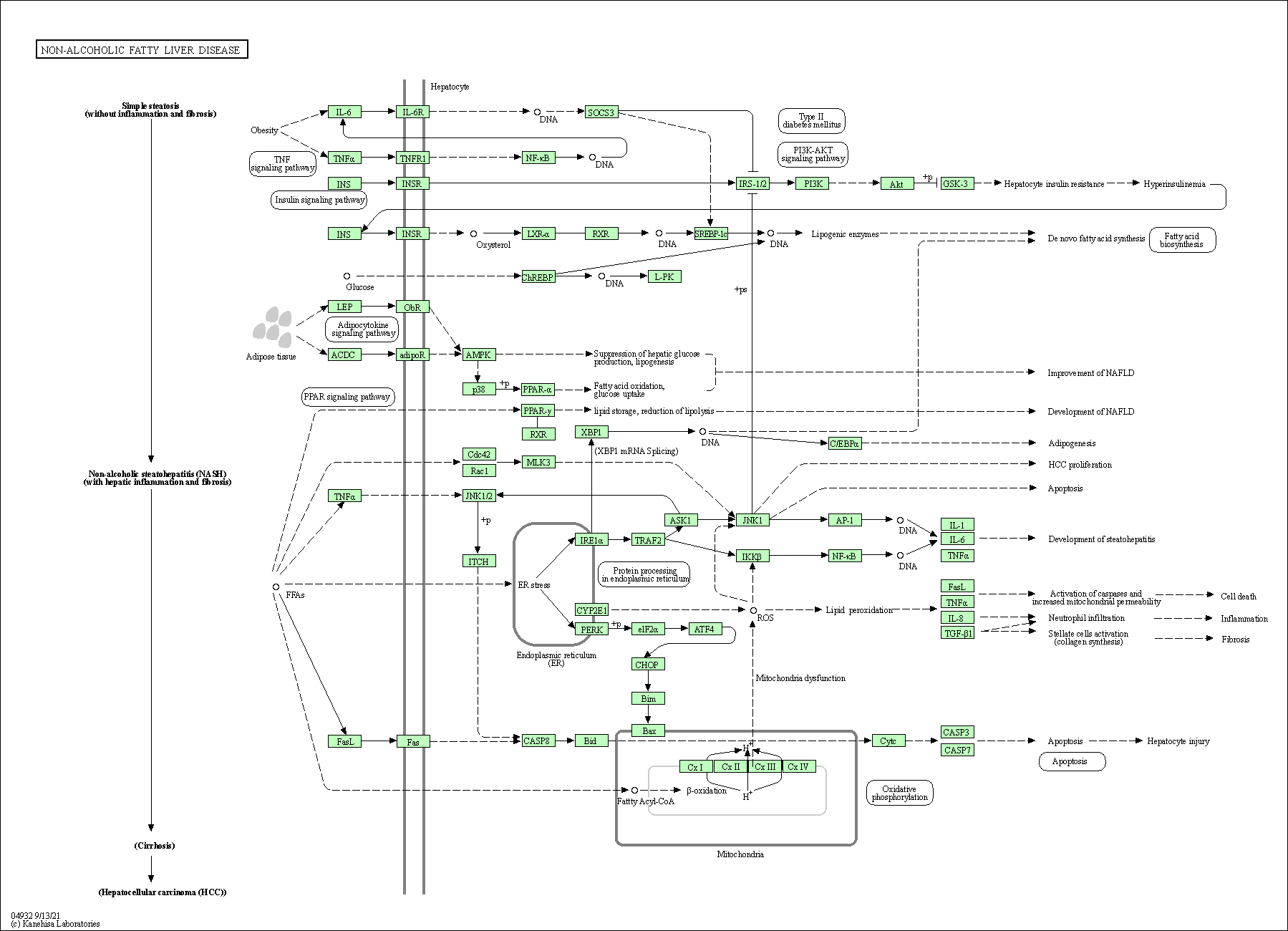

Supplement: S1 Data — (ZIP) [file pone.0274639.s001.zip › minimal data/GO+KEGG/R.KEGG/hsa04932.png]

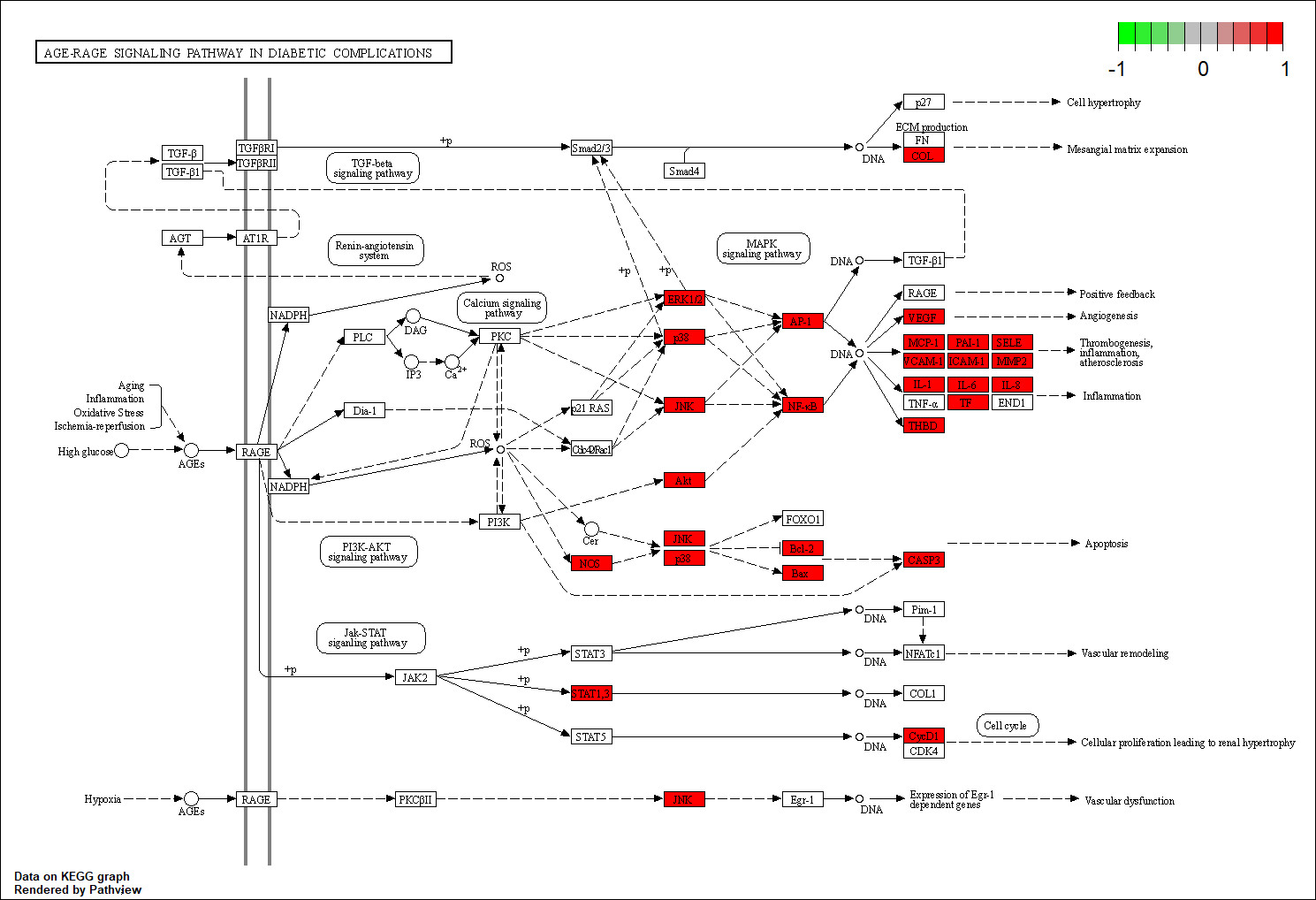

Supplement: S1 Data — (ZIP) [file pone.0274639.s001.zip › minimal data/GO+KEGG/R.KEGG/hsa04933.pathview.png]

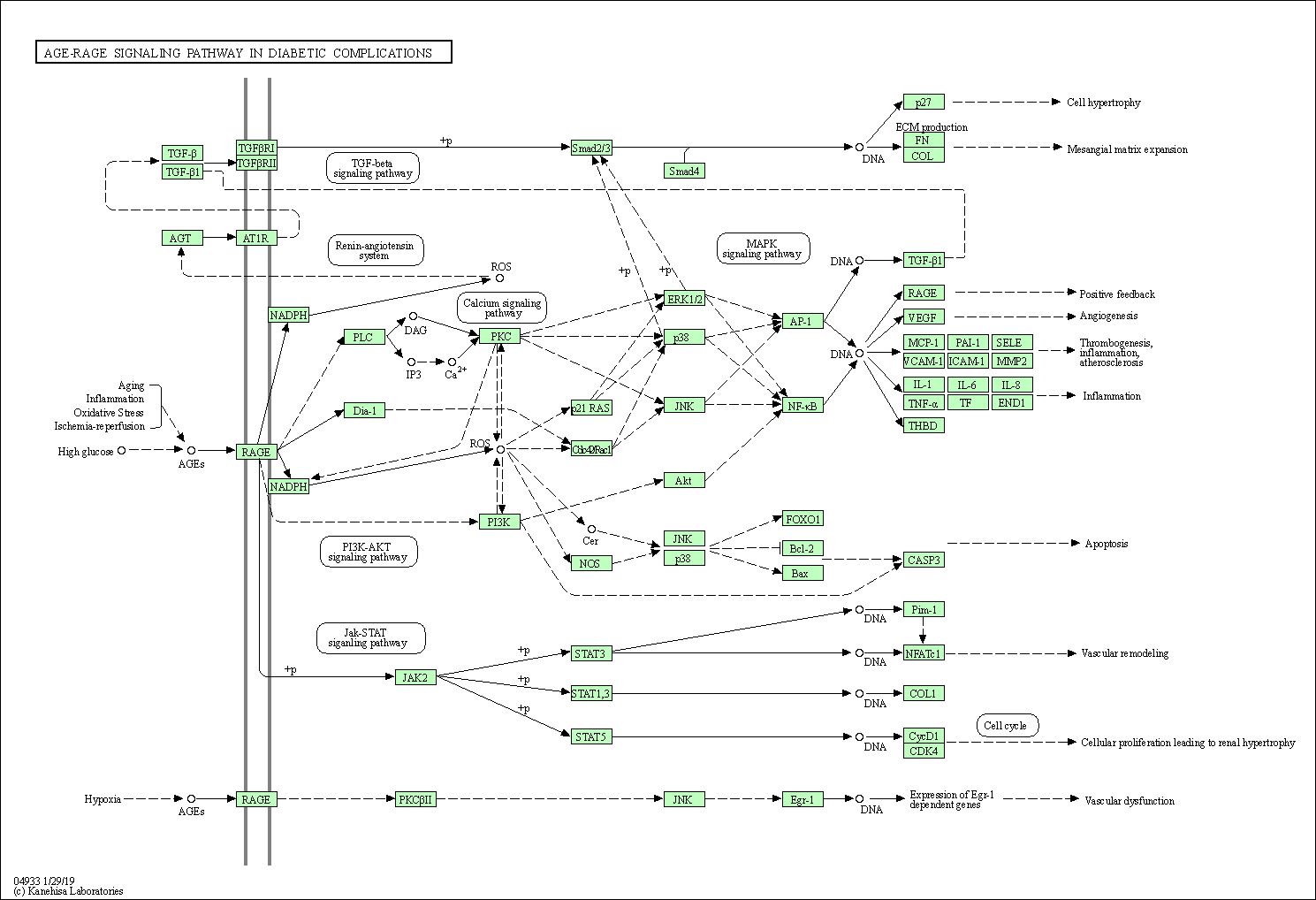

Supplement: S1 Data — (ZIP) [file pone.0274639.s001.zip › minimal data/GO+KEGG/R.KEGG/hsa04933.png]

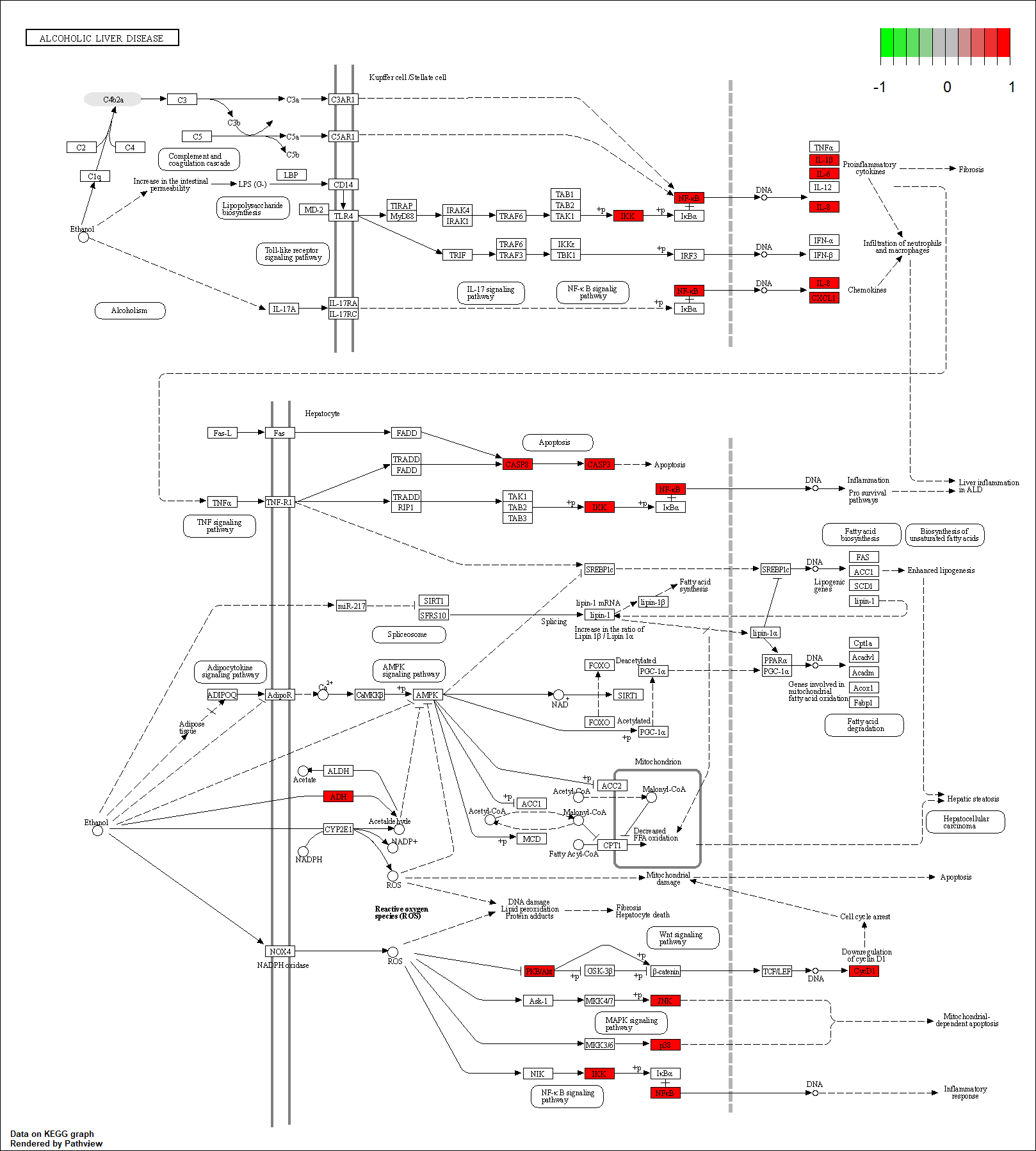

Supplement: S1 Data — (ZIP) [file pone.0274639.s001.zip › minimal data/GO+KEGG/R.KEGG/hsa04936.pathview.png]

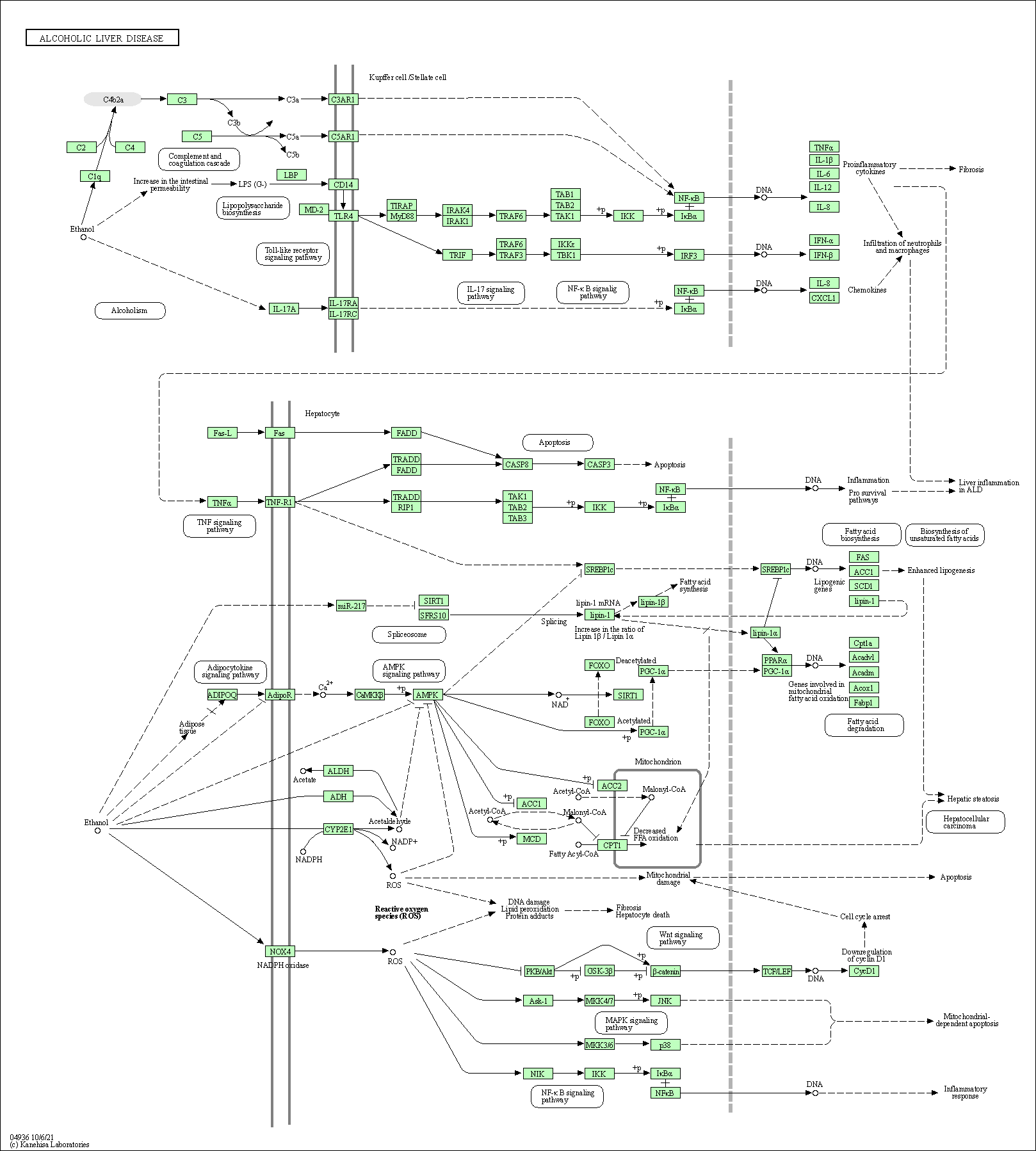

Supplement: S1 Data — (ZIP) [file pone.0274639.s001.zip › minimal data/GO+KEGG/R.KEGG/hsa04936.png]

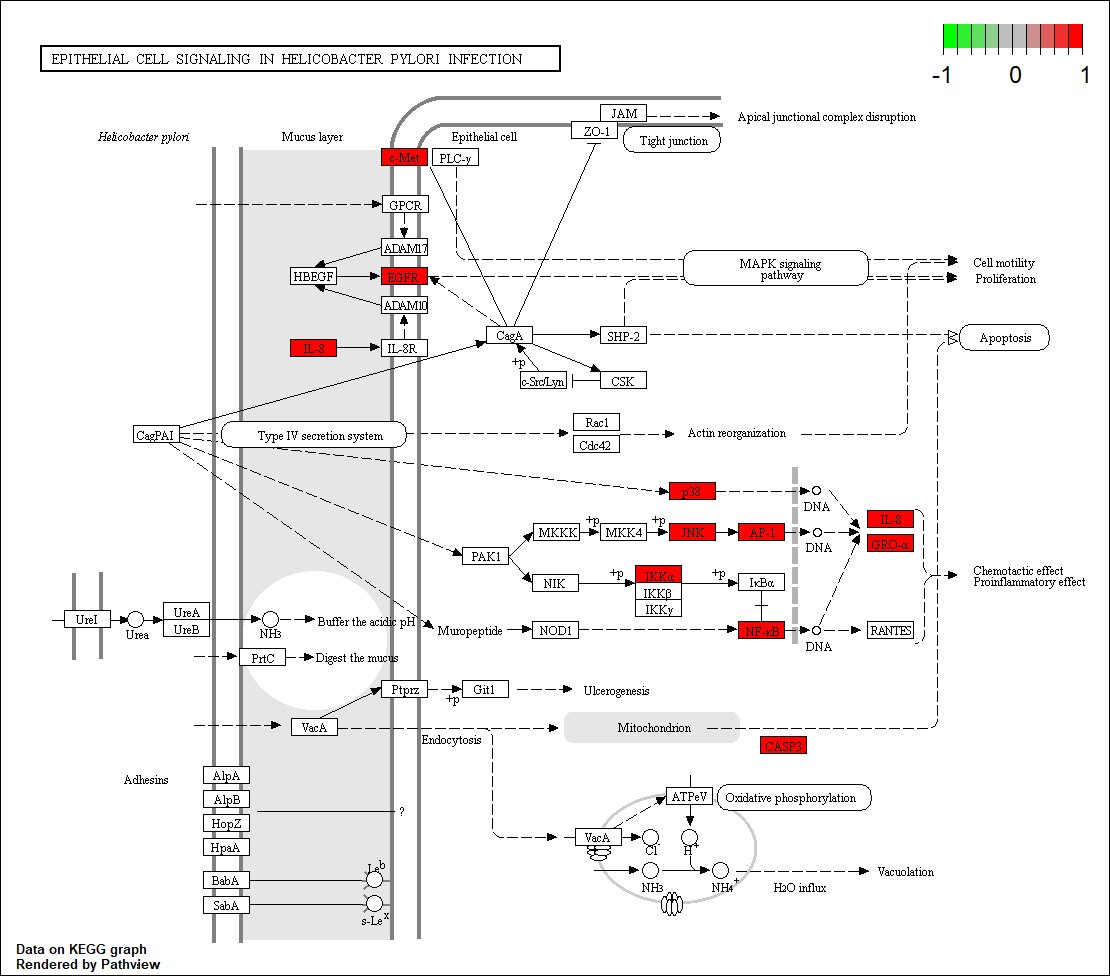

Supplement: S1 Data — (ZIP) [file pone.0274639.s001.zip › minimal data/GO+KEGG/R.KEGG/hsa05120.pathview.png]

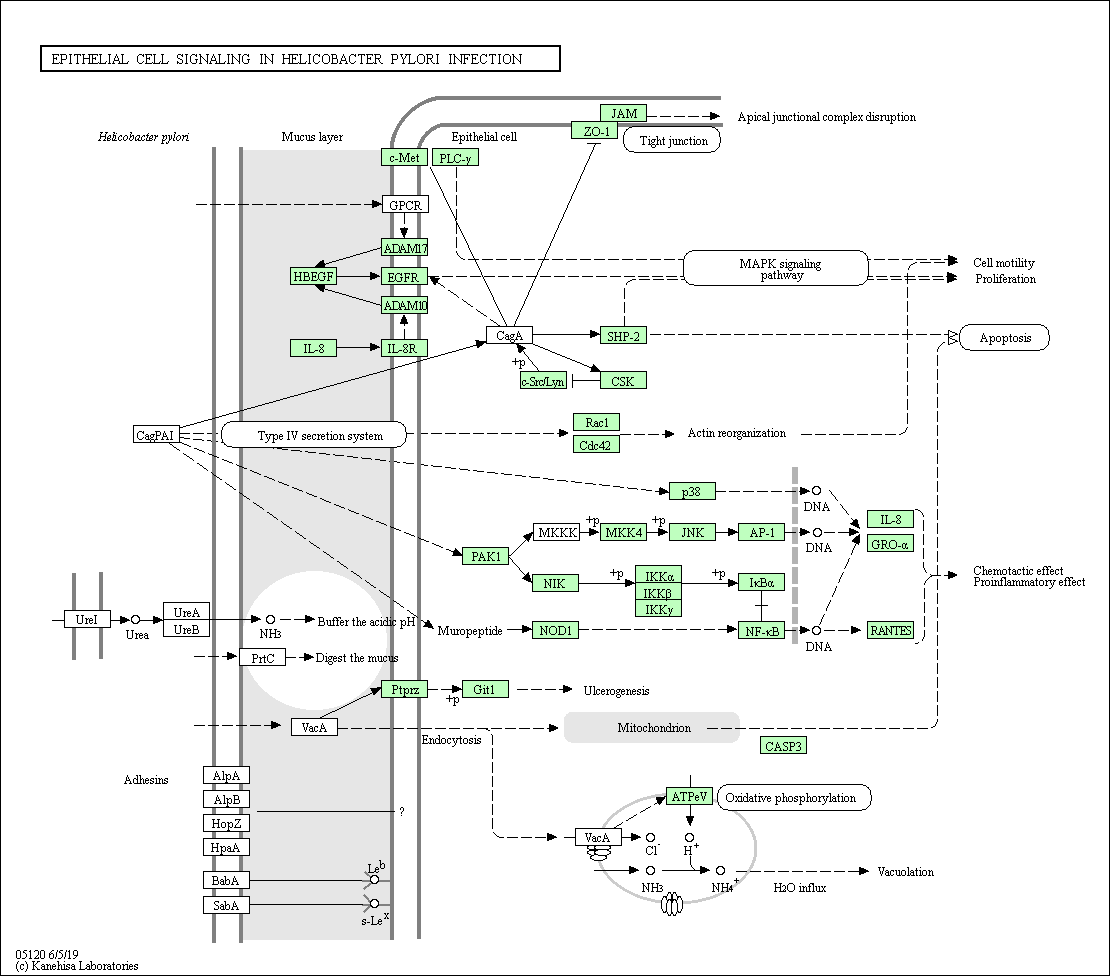

Supplement: S1 Data — (ZIP) [file pone.0274639.s001.zip › minimal data/GO+KEGG/R.KEGG/hsa05120.png]

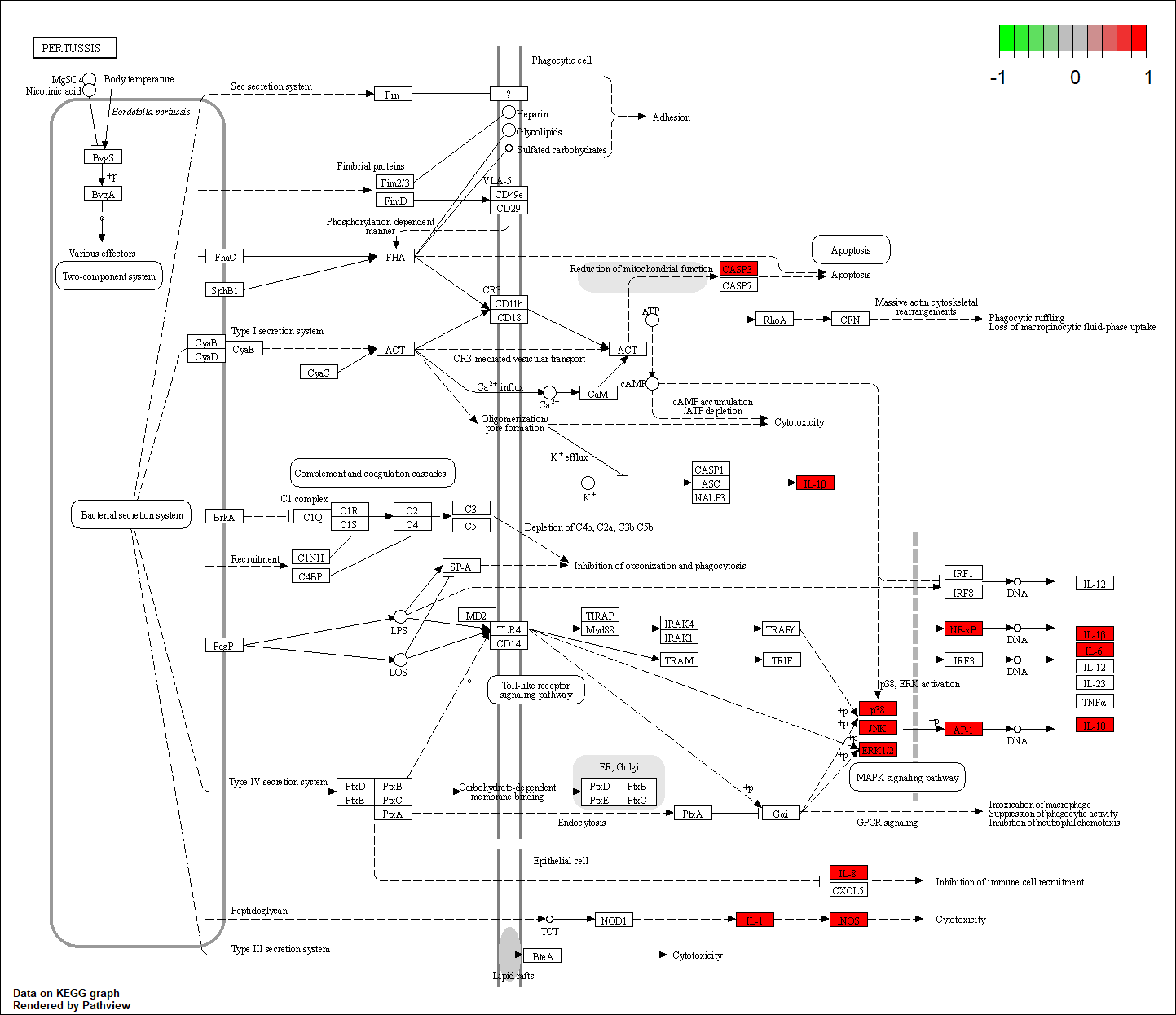

Supplement: S1 Data — (ZIP) [file pone.0274639.s001.zip › minimal data/GO+KEGG/R.KEGG/hsa05133.pathview.png]

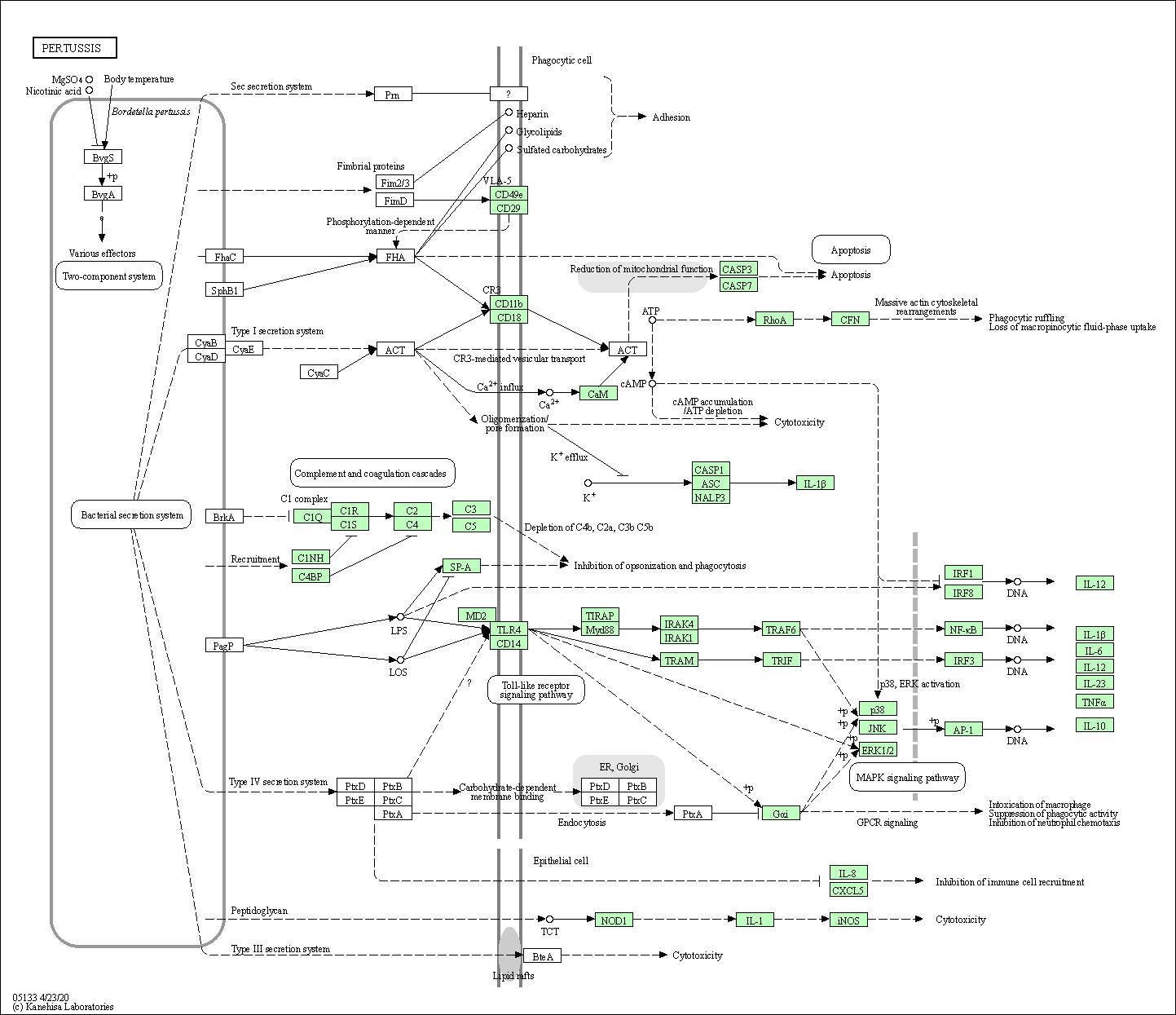

Supplement: S1 Data — (ZIP) [file pone.0274639.s001.zip › minimal data/GO+KEGG/R.KEGG/hsa05133.png]

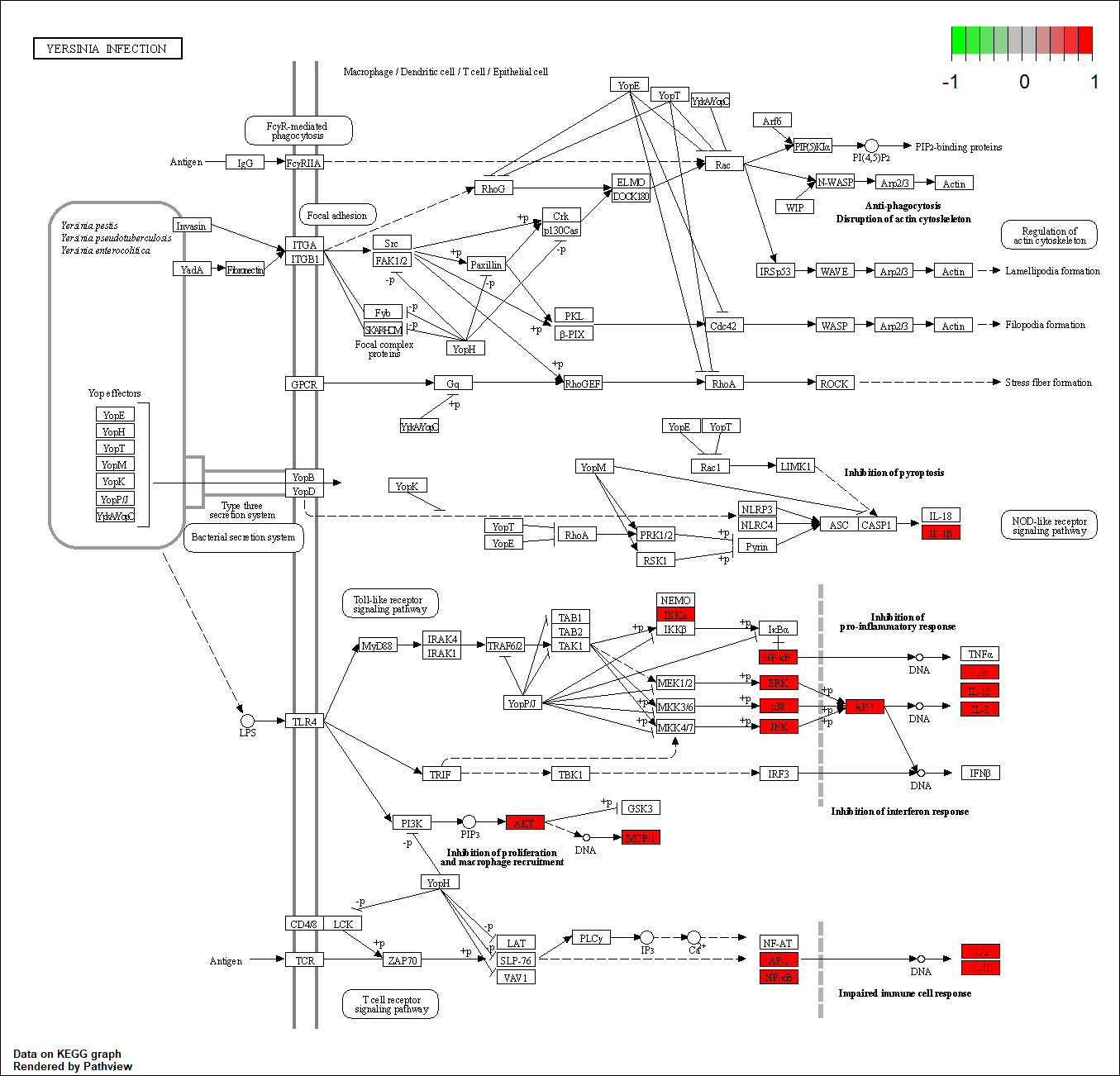

Supplement: S1 Data — (ZIP) [file pone.0274639.s001.zip › minimal data/GO+KEGG/R.KEGG/hsa05135.pathview.png]

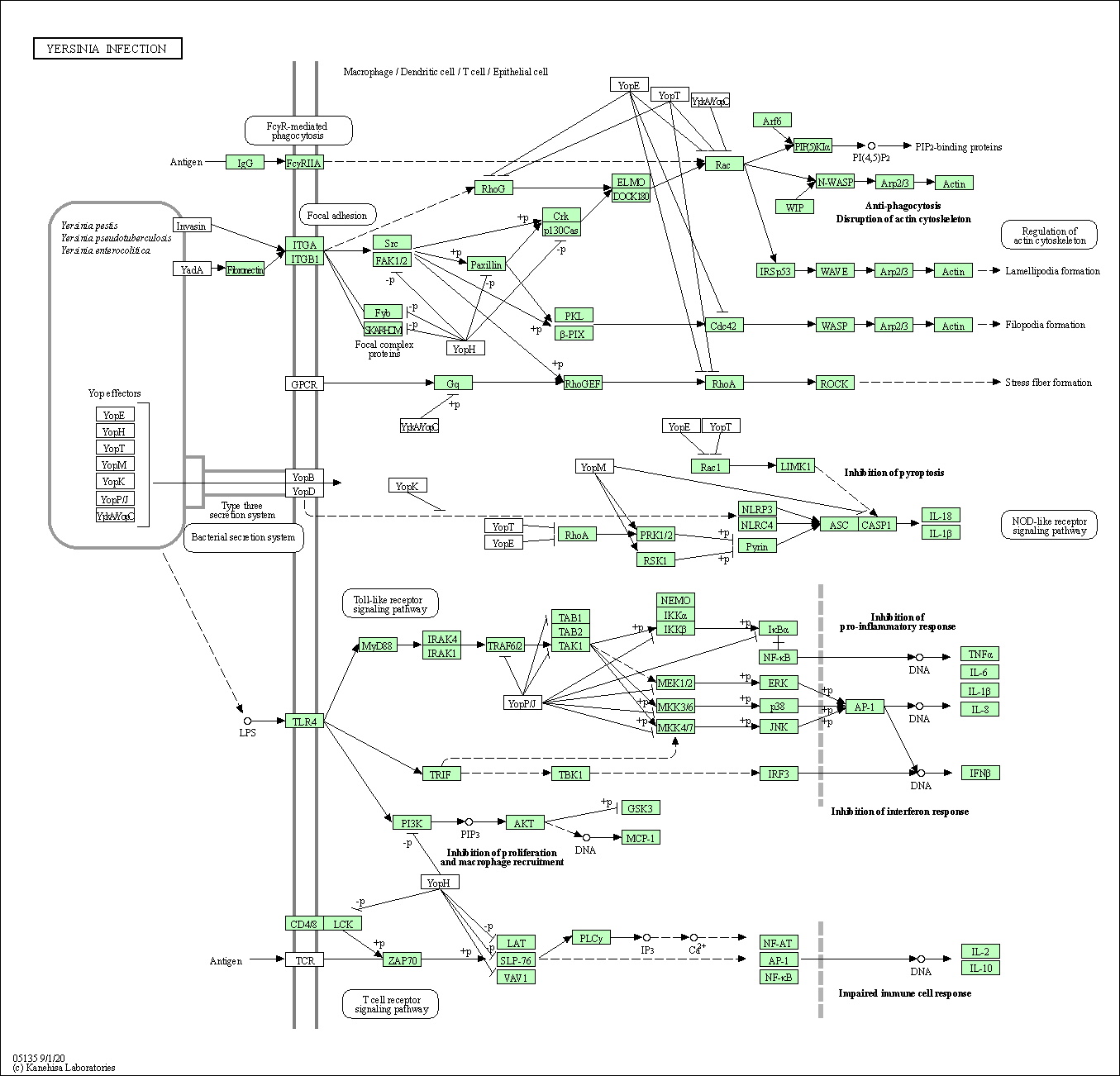

Supplement: S1 Data — (ZIP) [file pone.0274639.s001.zip › minimal data/GO+KEGG/R.KEGG/hsa05135.png]

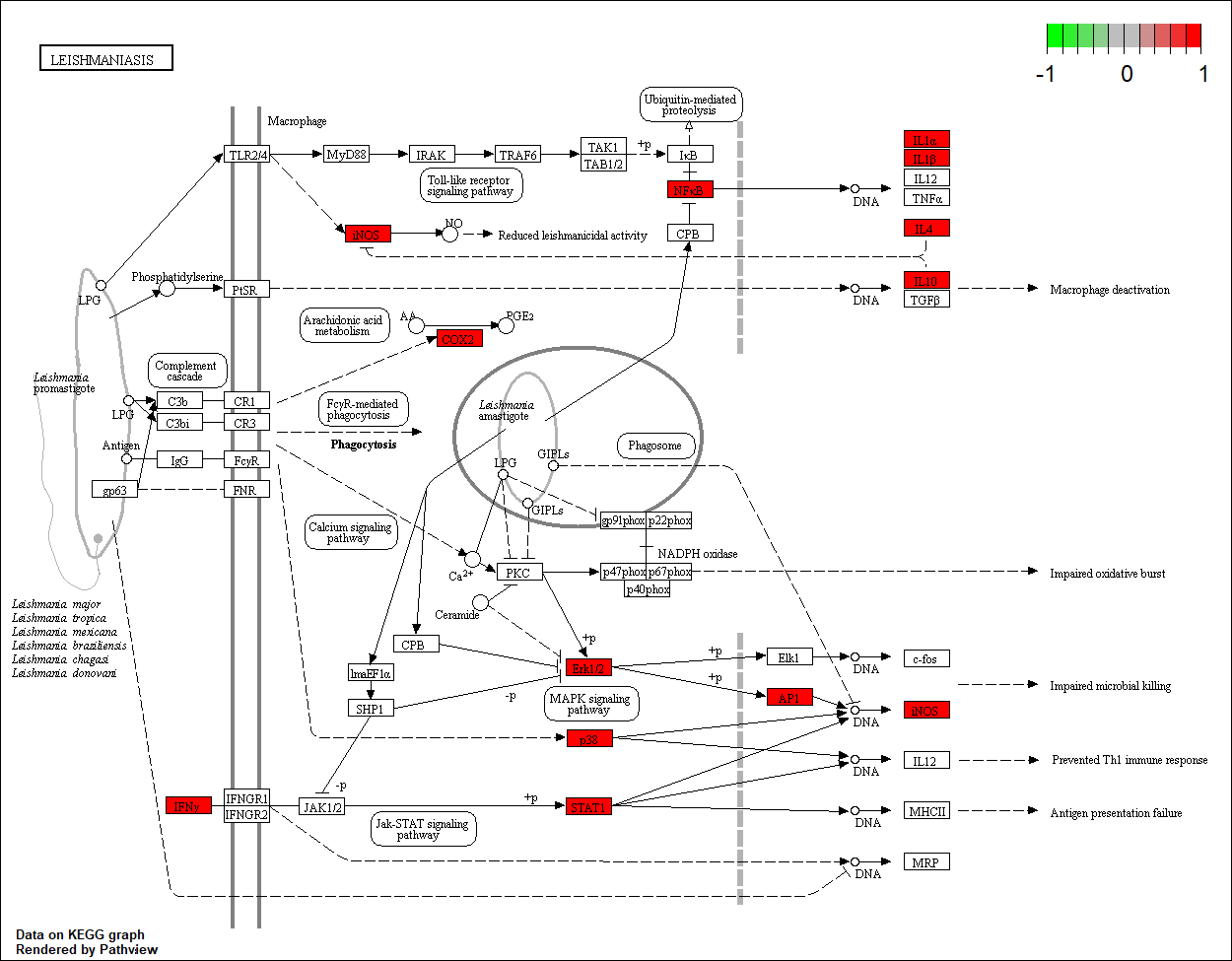

Supplement: S1 Data — (ZIP) [file pone.0274639.s001.zip › minimal data/GO+KEGG/R.KEGG/hsa05140.pathview.png]

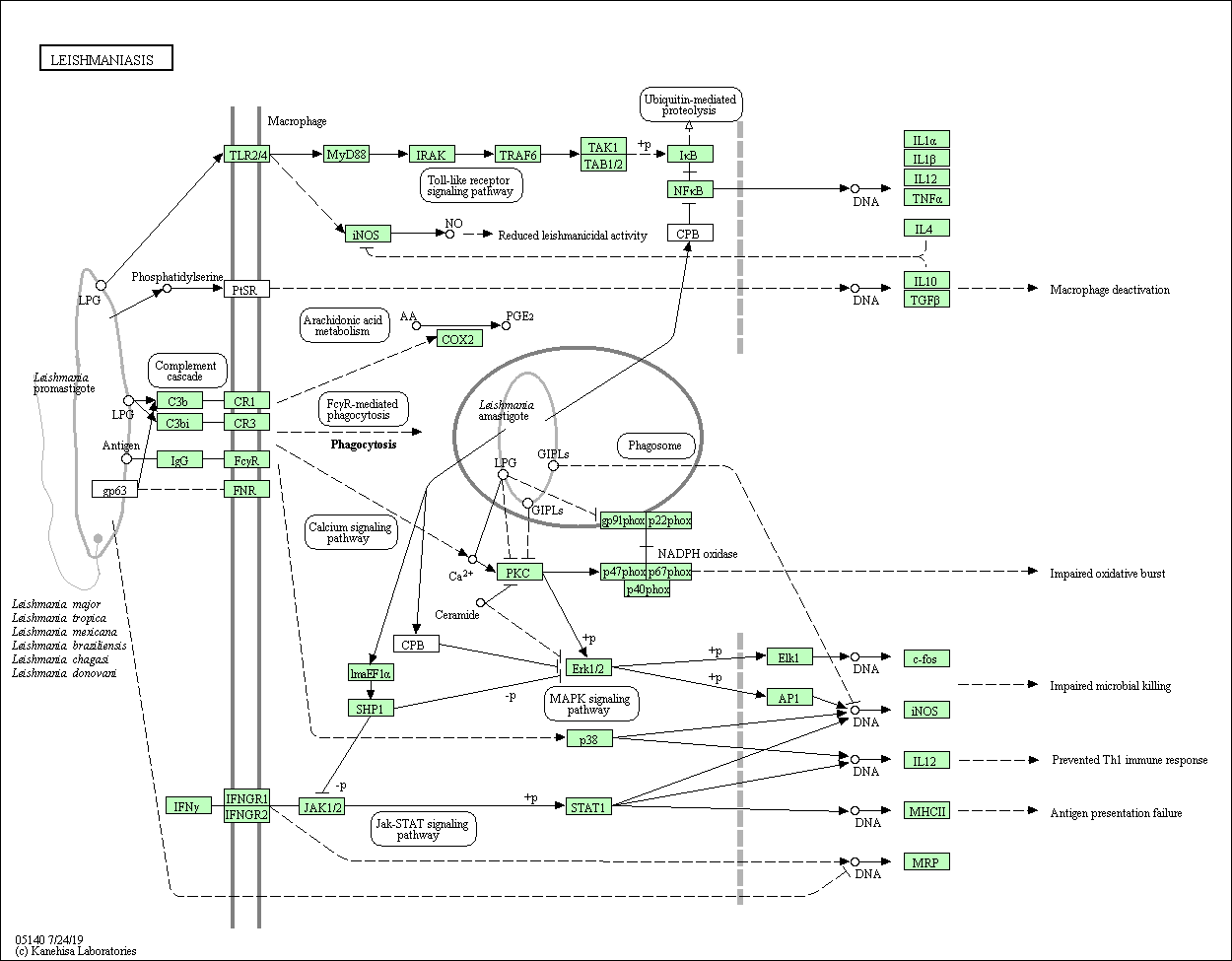

Supplement: S1 Data — (ZIP) [file pone.0274639.s001.zip › minimal data/GO+KEGG/R.KEGG/hsa05140.png]

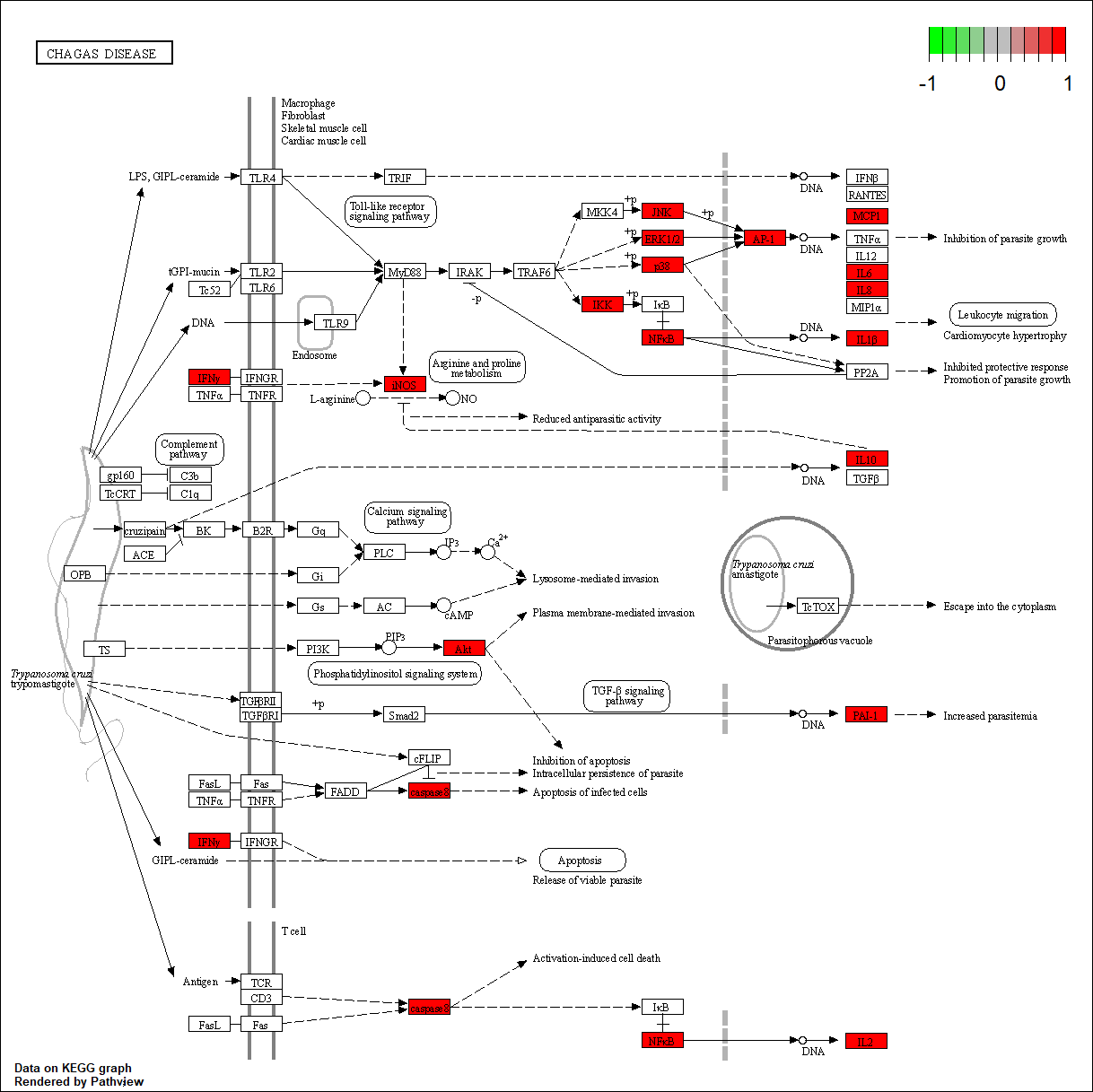

Supplement: S1 Data — (ZIP) [file pone.0274639.s001.zip › minimal data/GO+KEGG/R.KEGG/hsa05142.pathview.png]

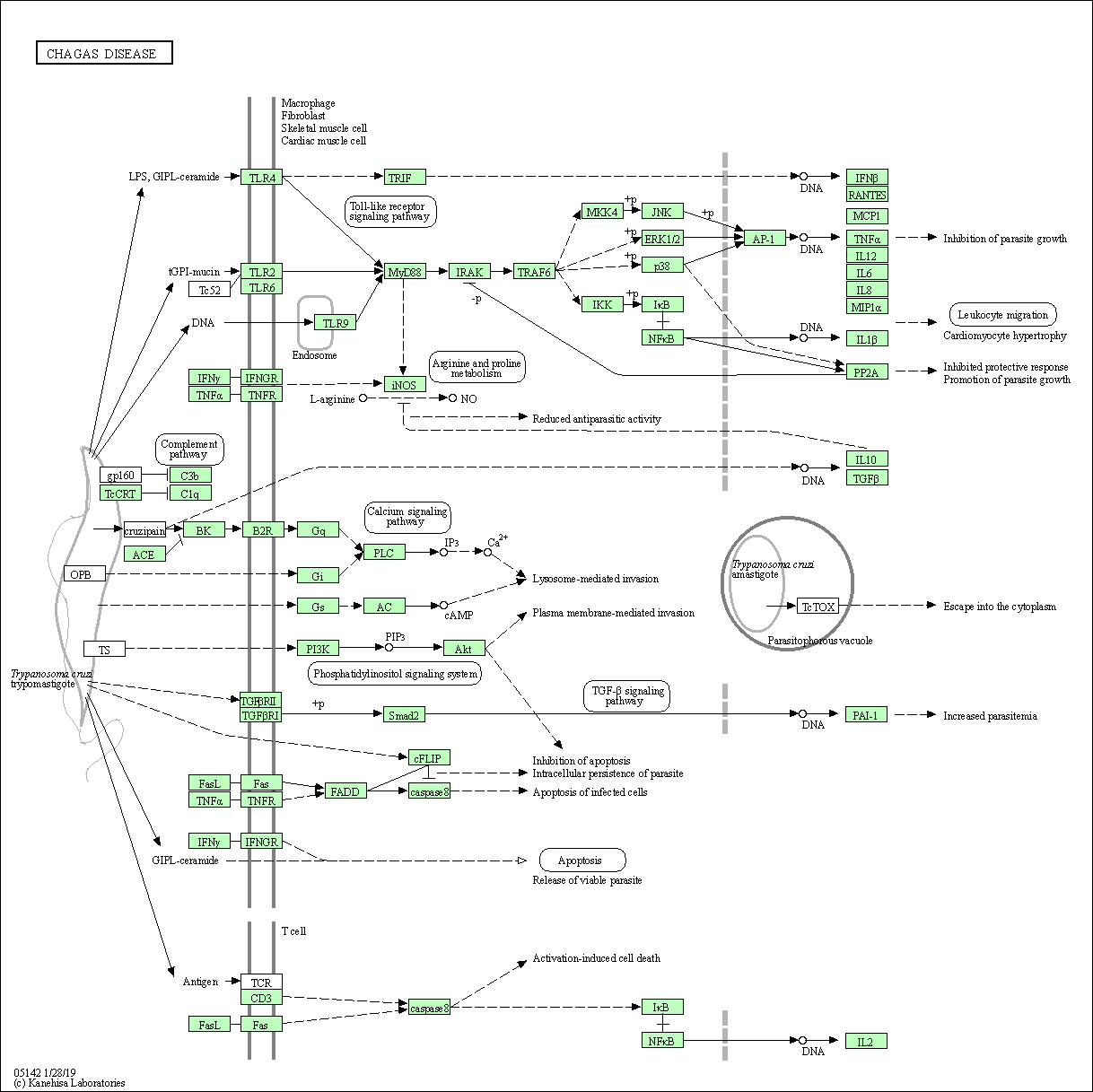

Supplement: S1 Data — (ZIP) [file pone.0274639.s001.zip › minimal data/GO+KEGG/R.KEGG/hsa05142.png]

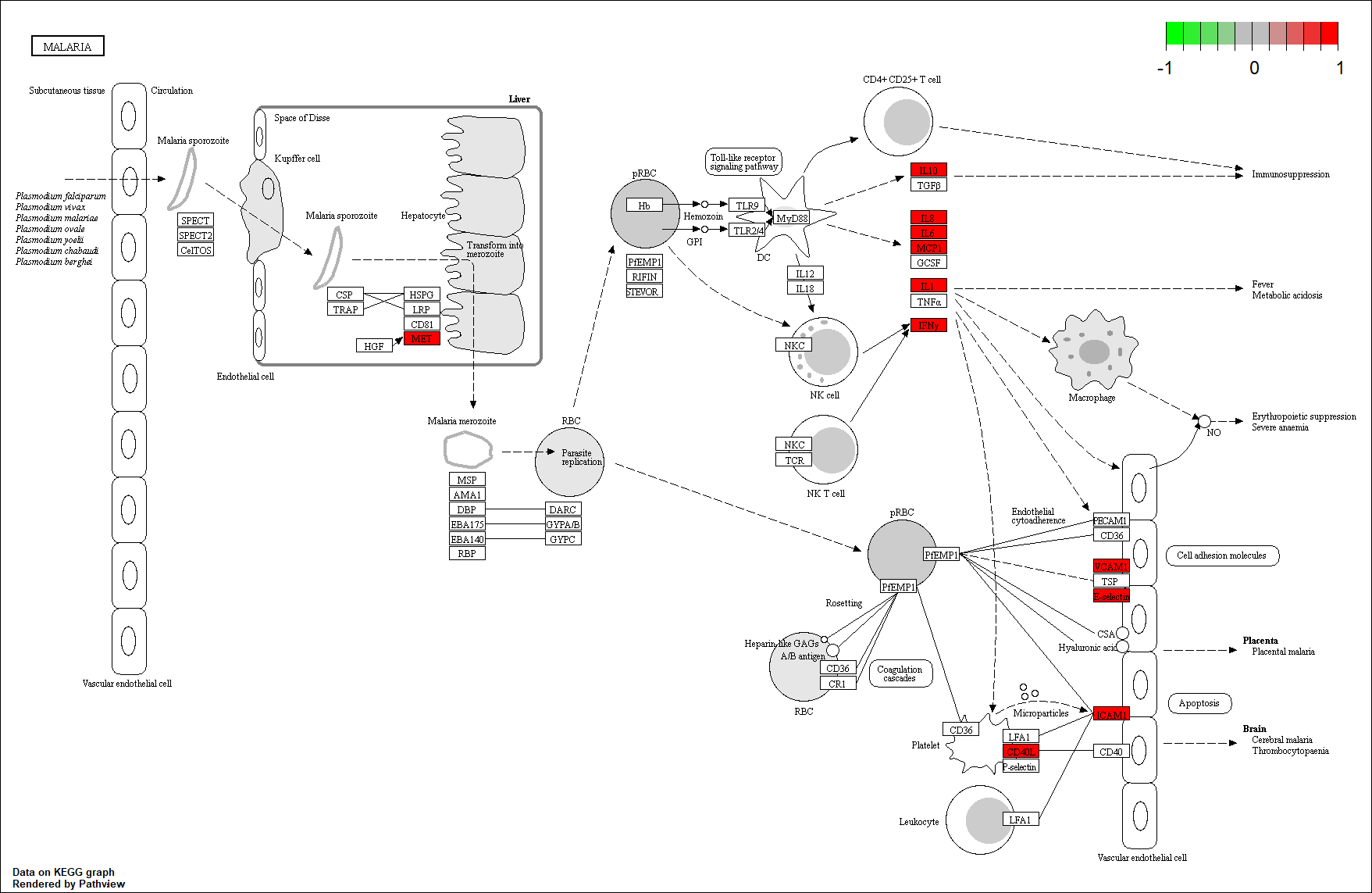

Supplement: S1 Data — (ZIP) [file pone.0274639.s001.zip › minimal data/GO+KEGG/R.KEGG/hsa05144.pathview.png]

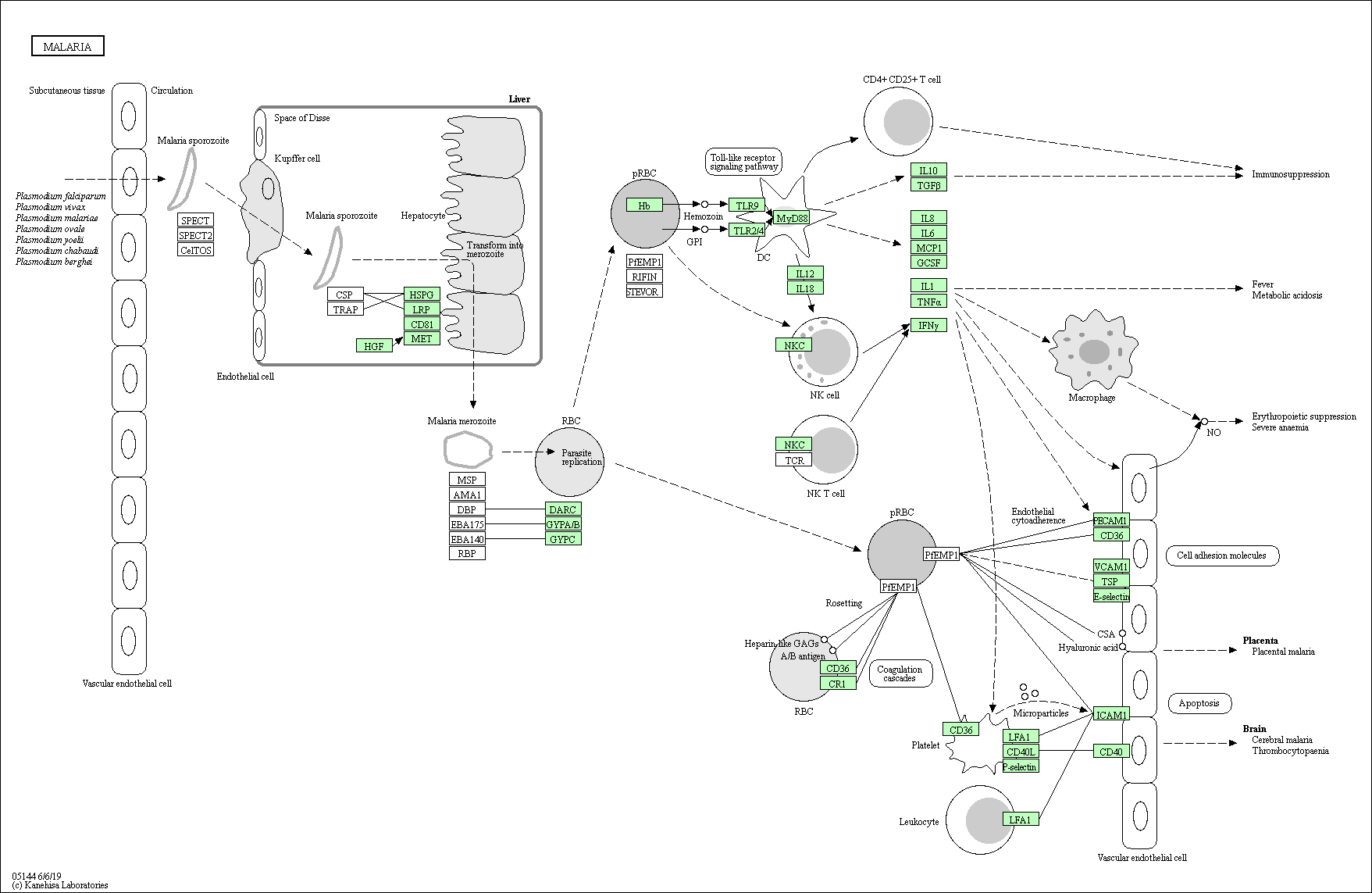

Supplement: S1 Data — (ZIP) [file pone.0274639.s001.zip › minimal data/GO+KEGG/R.KEGG/hsa05144.png]

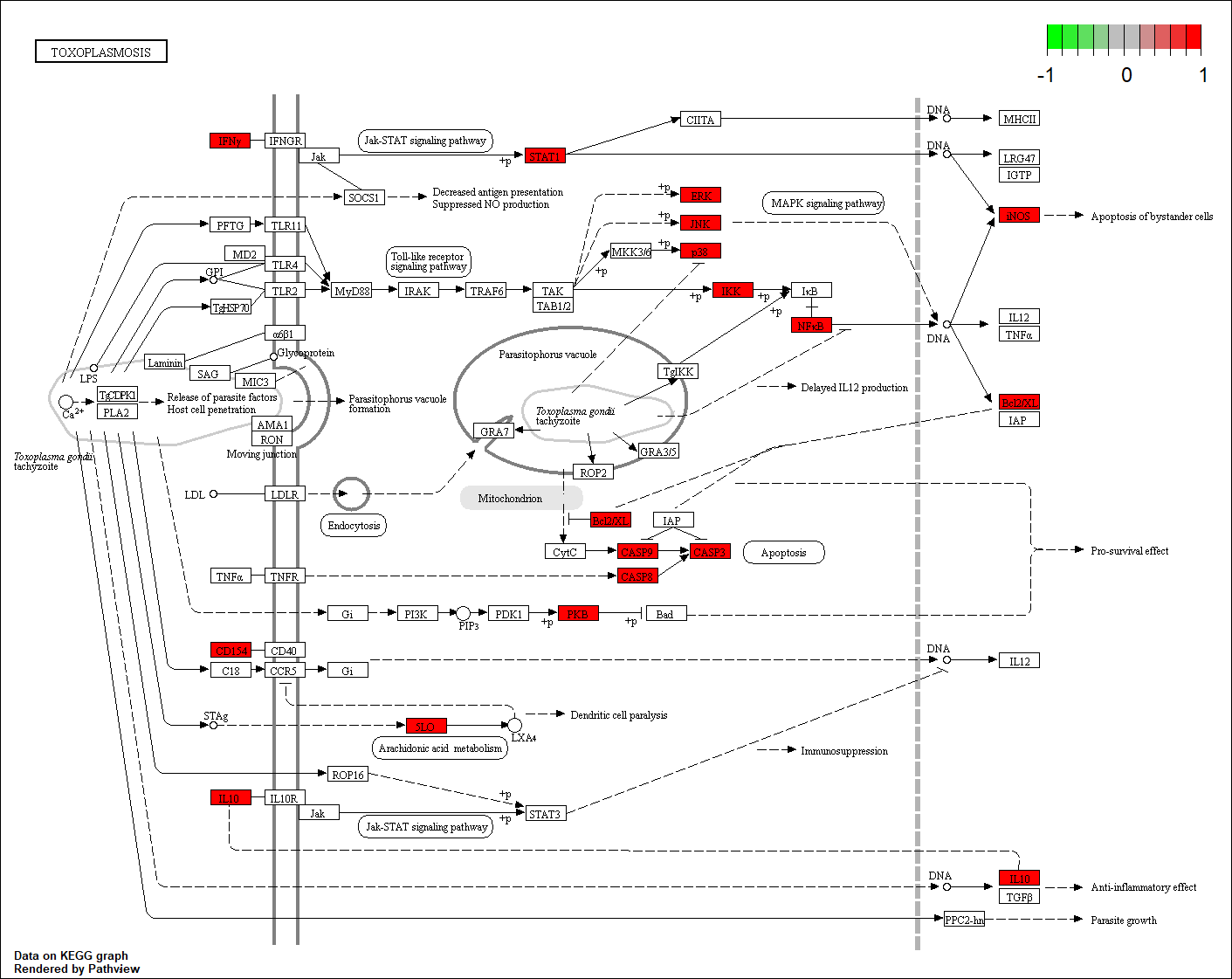

Supplement: S1 Data — (ZIP) [file pone.0274639.s001.zip › minimal data/GO+KEGG/R.KEGG/hsa05145.pathview.png]

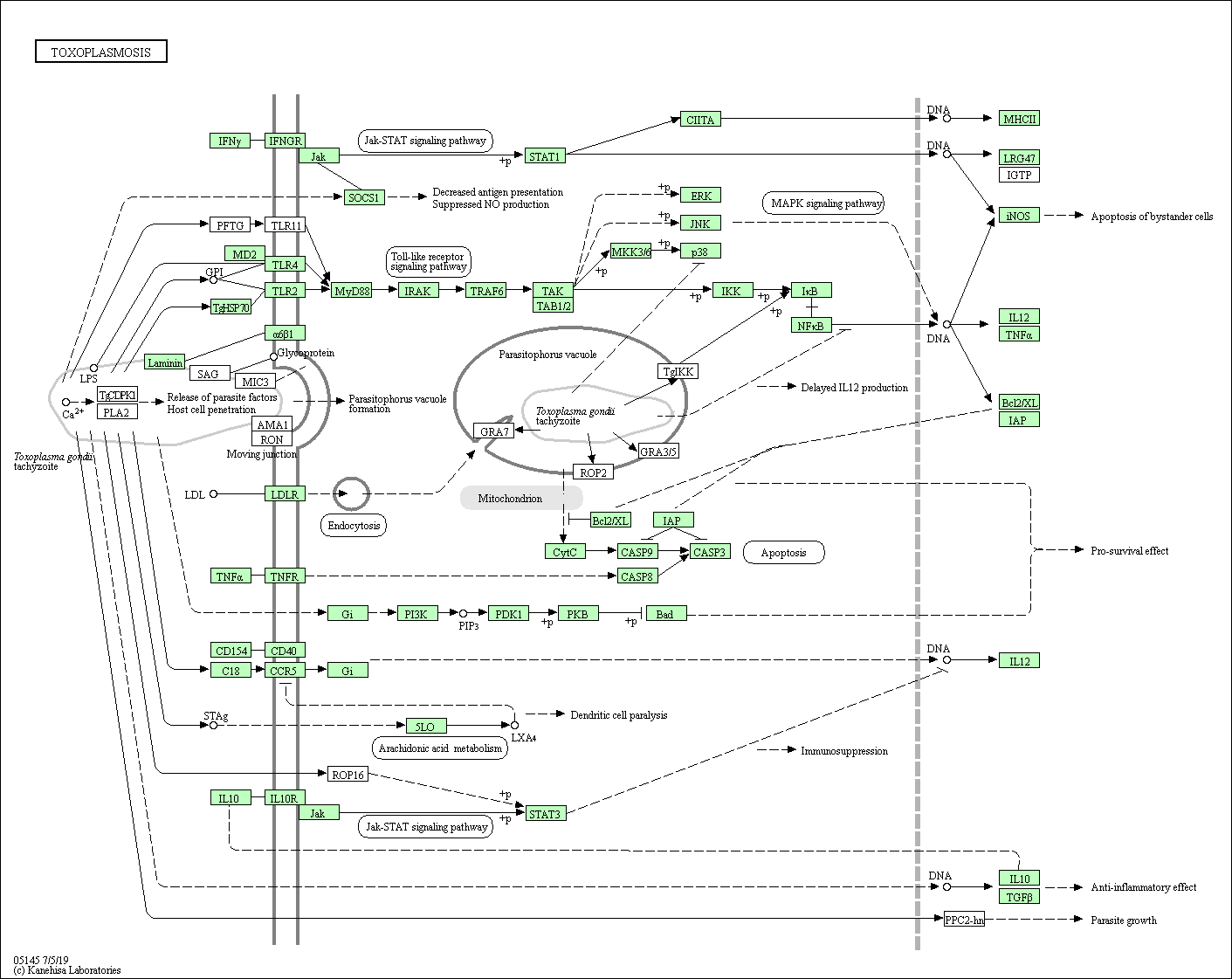

Supplement: S1 Data — (ZIP) [file pone.0274639.s001.zip › minimal data/GO+KEGG/R.KEGG/hsa05145.png]

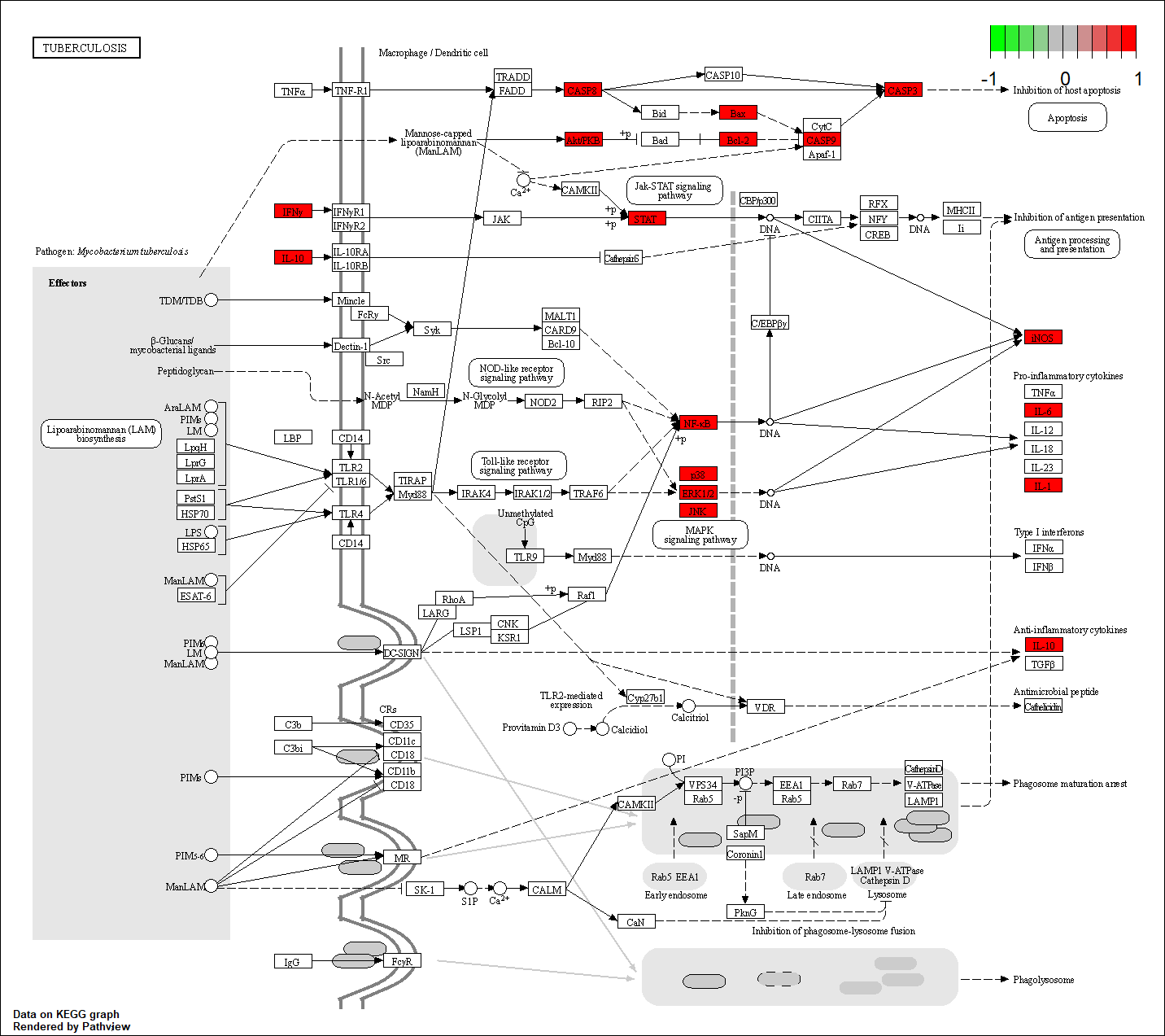

Supplement: S1 Data — (ZIP) [file pone.0274639.s001.zip › minimal data/GO+KEGG/R.KEGG/hsa05152.pathview.png]

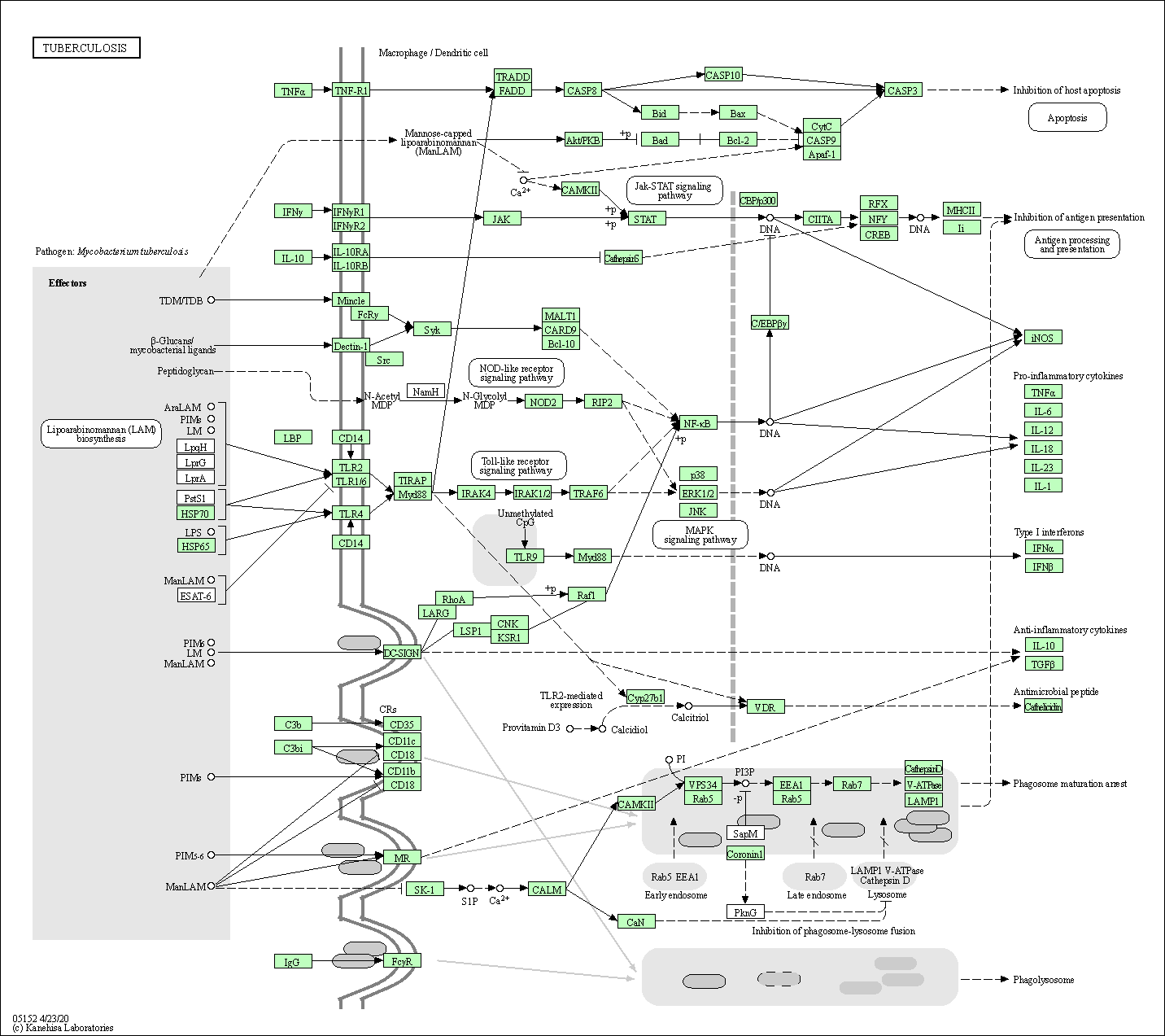

Supplement: S1 Data — (ZIP) [file pone.0274639.s001.zip › minimal data/GO+KEGG/R.KEGG/hsa05152.png]

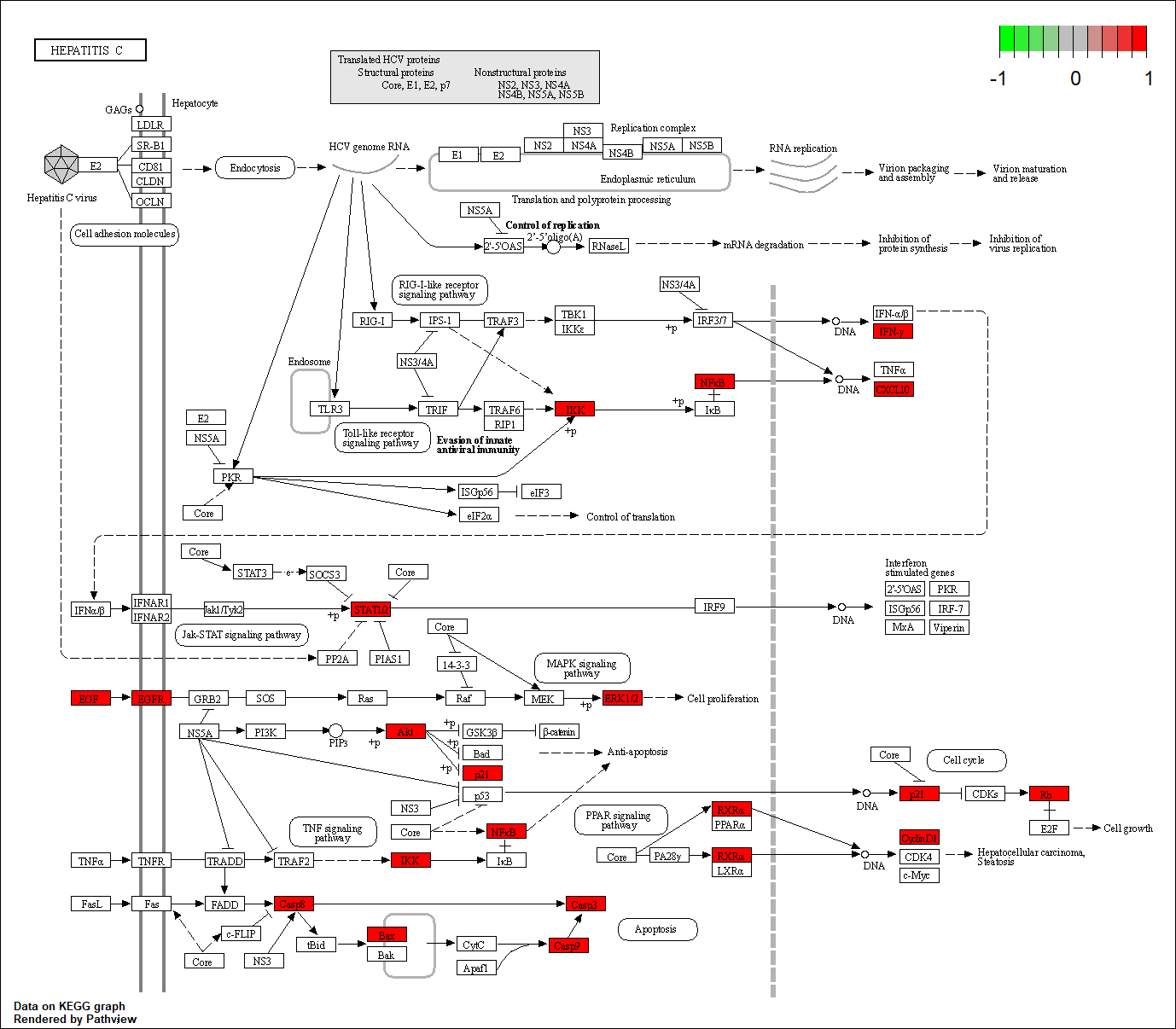

Supplement: S1 Data — (ZIP) [file pone.0274639.s001.zip › minimal data/GO+KEGG/R.KEGG/hsa05160.pathview.png]

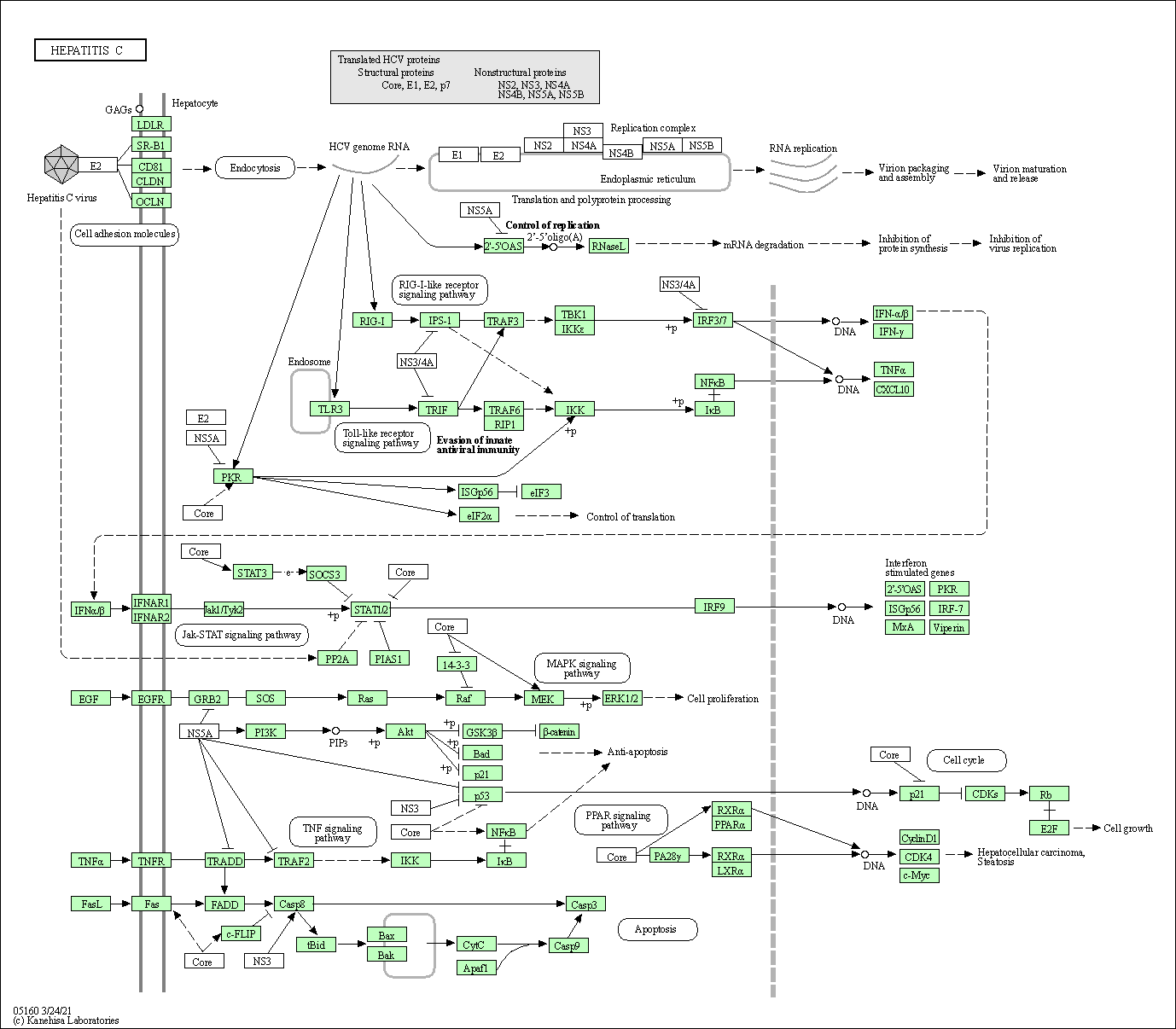

Supplement: S1 Data — (ZIP) [file pone.0274639.s001.zip › minimal data/GO+KEGG/R.KEGG/hsa05160.png]

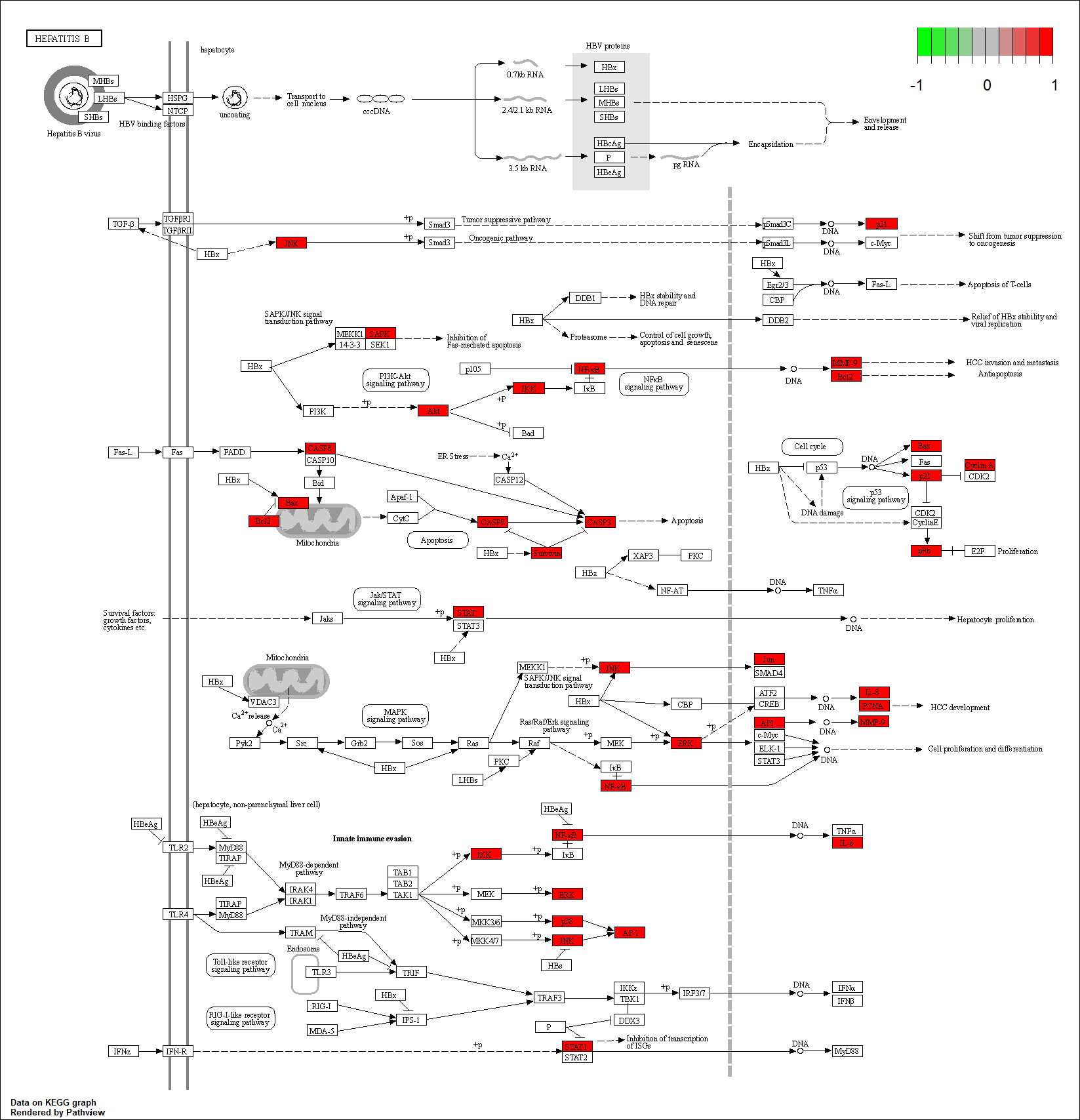

Supplement: S1 Data — (ZIP) [file pone.0274639.s001.zip › minimal data/GO+KEGG/R.KEGG/hsa05161.pathview.png]

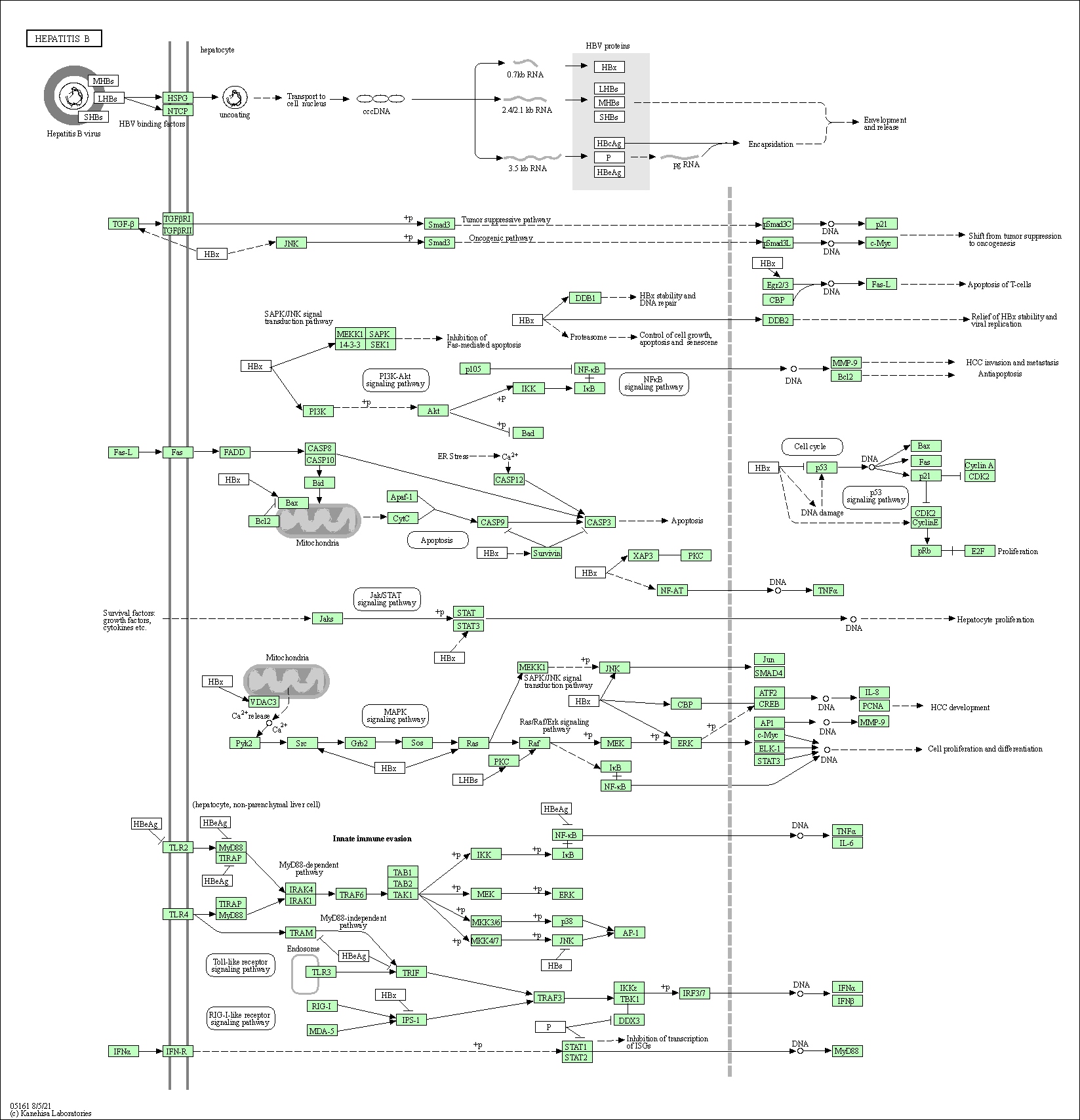

Supplement: S1 Data — (ZIP) [file pone.0274639.s001.zip › minimal data/GO+KEGG/R.KEGG/hsa05161.png]

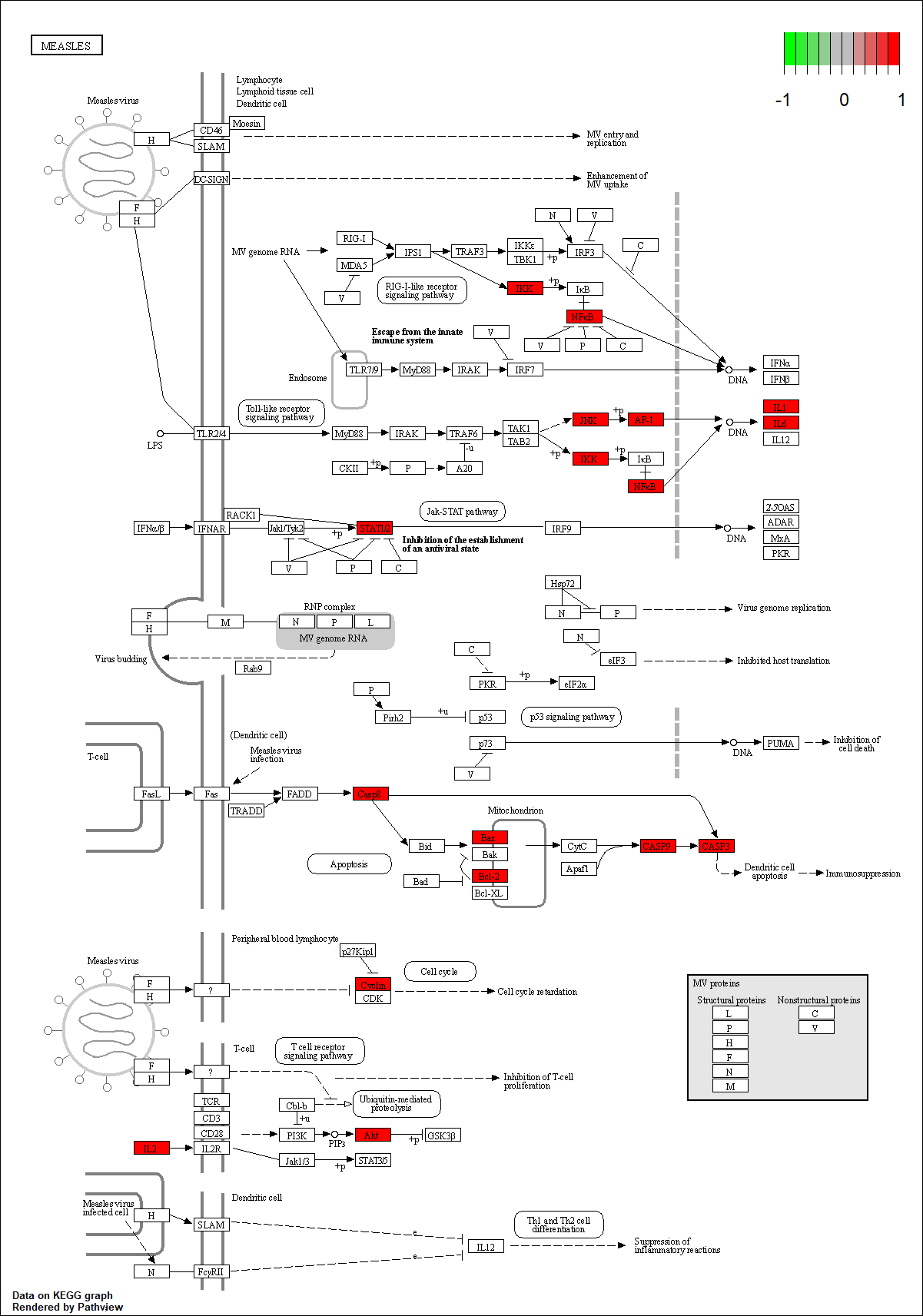

Supplement: S1 Data — (ZIP) [file pone.0274639.s001.zip › minimal data/GO+KEGG/R.KEGG/hsa05162.pathview.png]

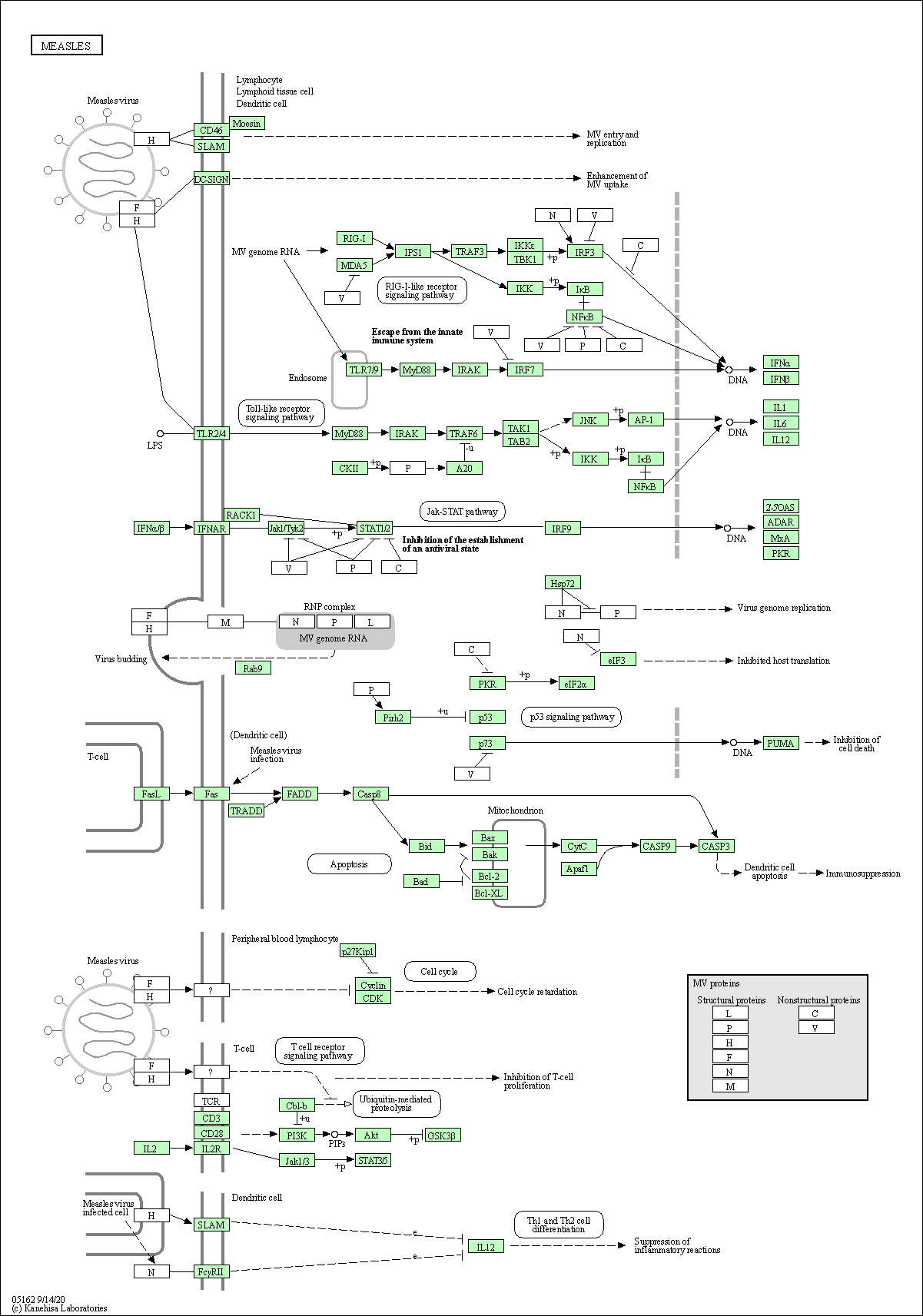

Supplement: S1 Data — (ZIP) [file pone.0274639.s001.zip › minimal data/GO+KEGG/R.KEGG/hsa05162.png]

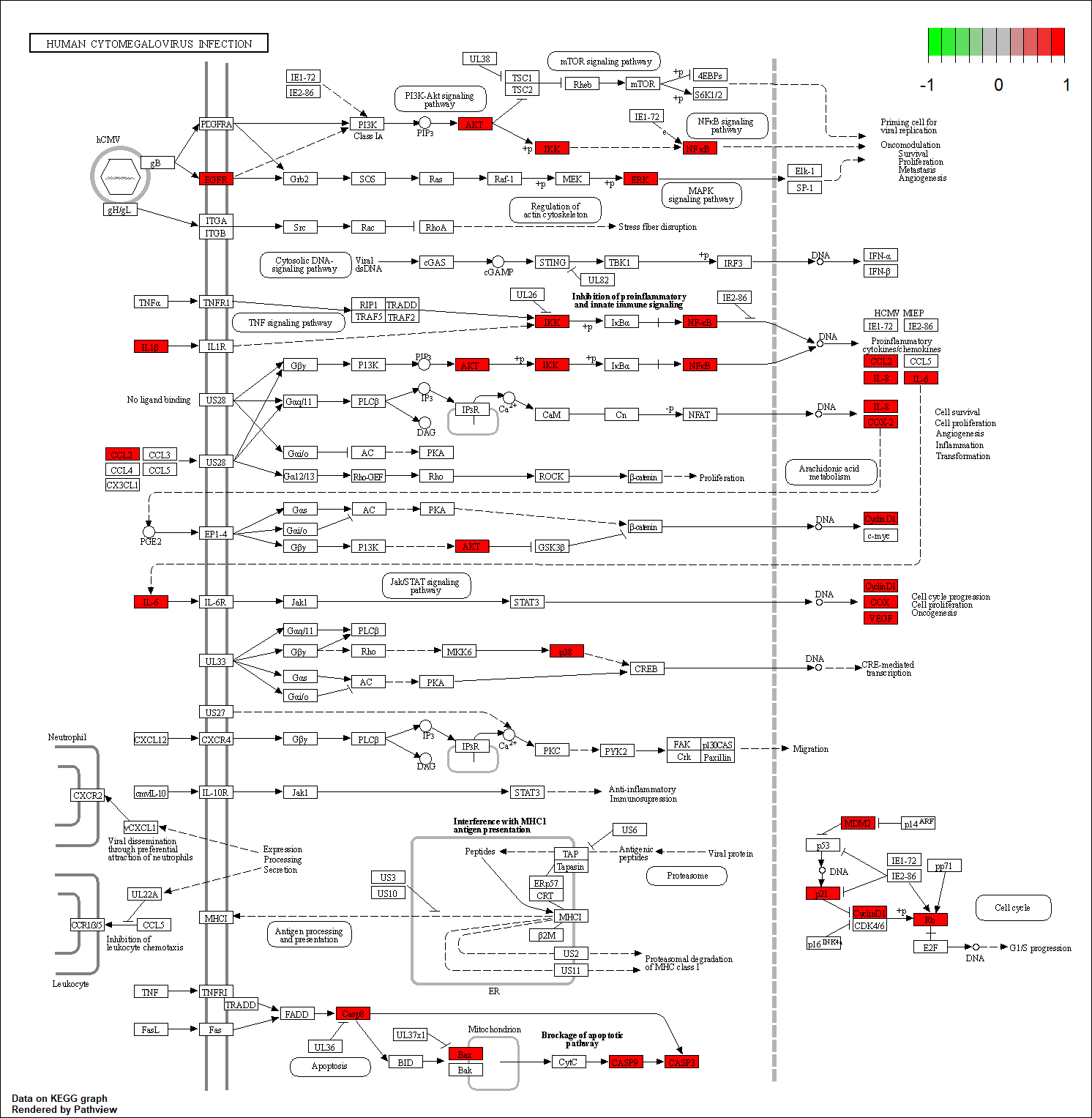

Supplement: S1 Data — (ZIP) [file pone.0274639.s001.zip › minimal data/GO+KEGG/R.KEGG/hsa05163.pathview.png]

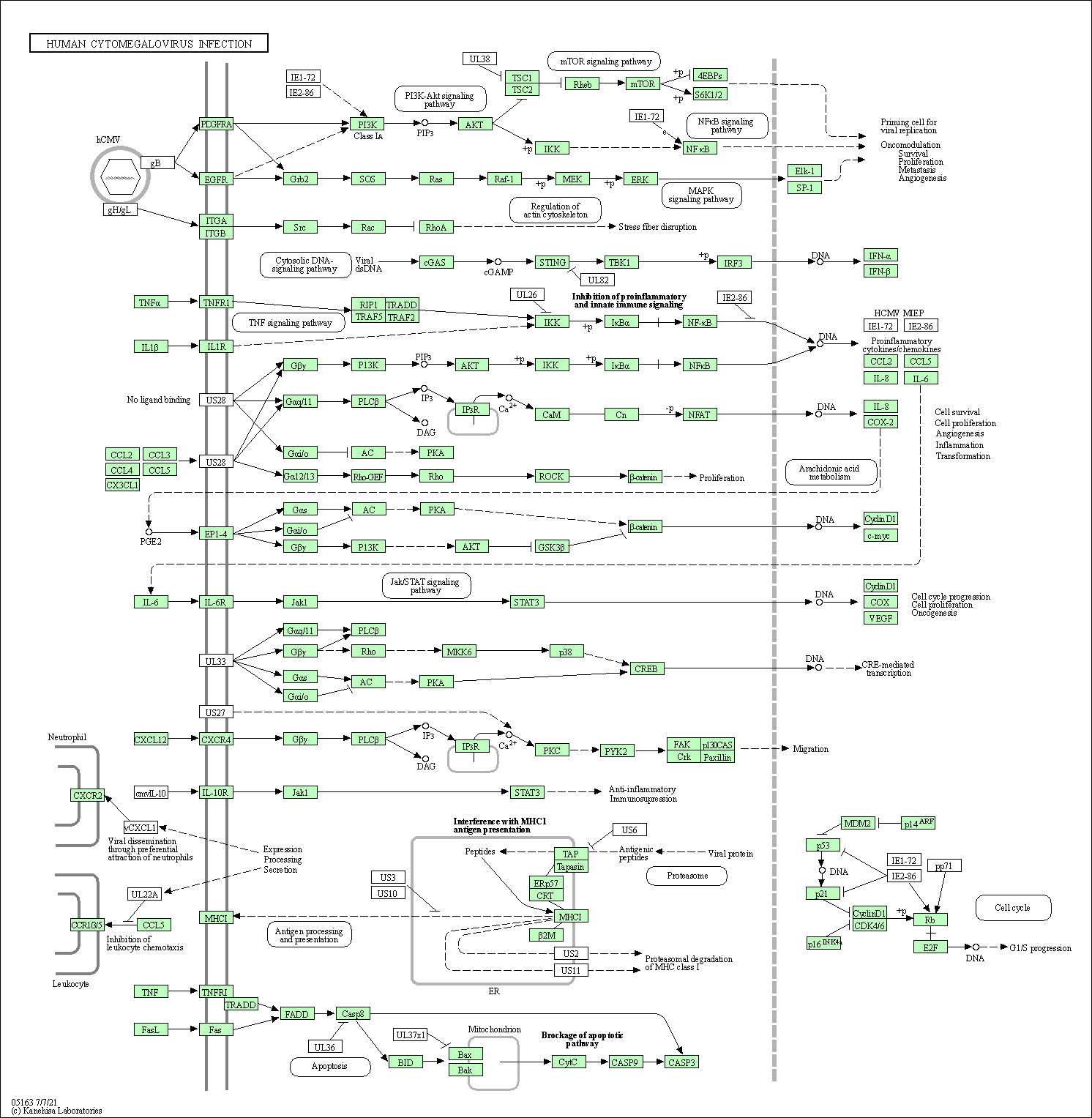

Supplement: S1 Data — (ZIP) [file pone.0274639.s001.zip › minimal data/GO+KEGG/R.KEGG/hsa05163.png]

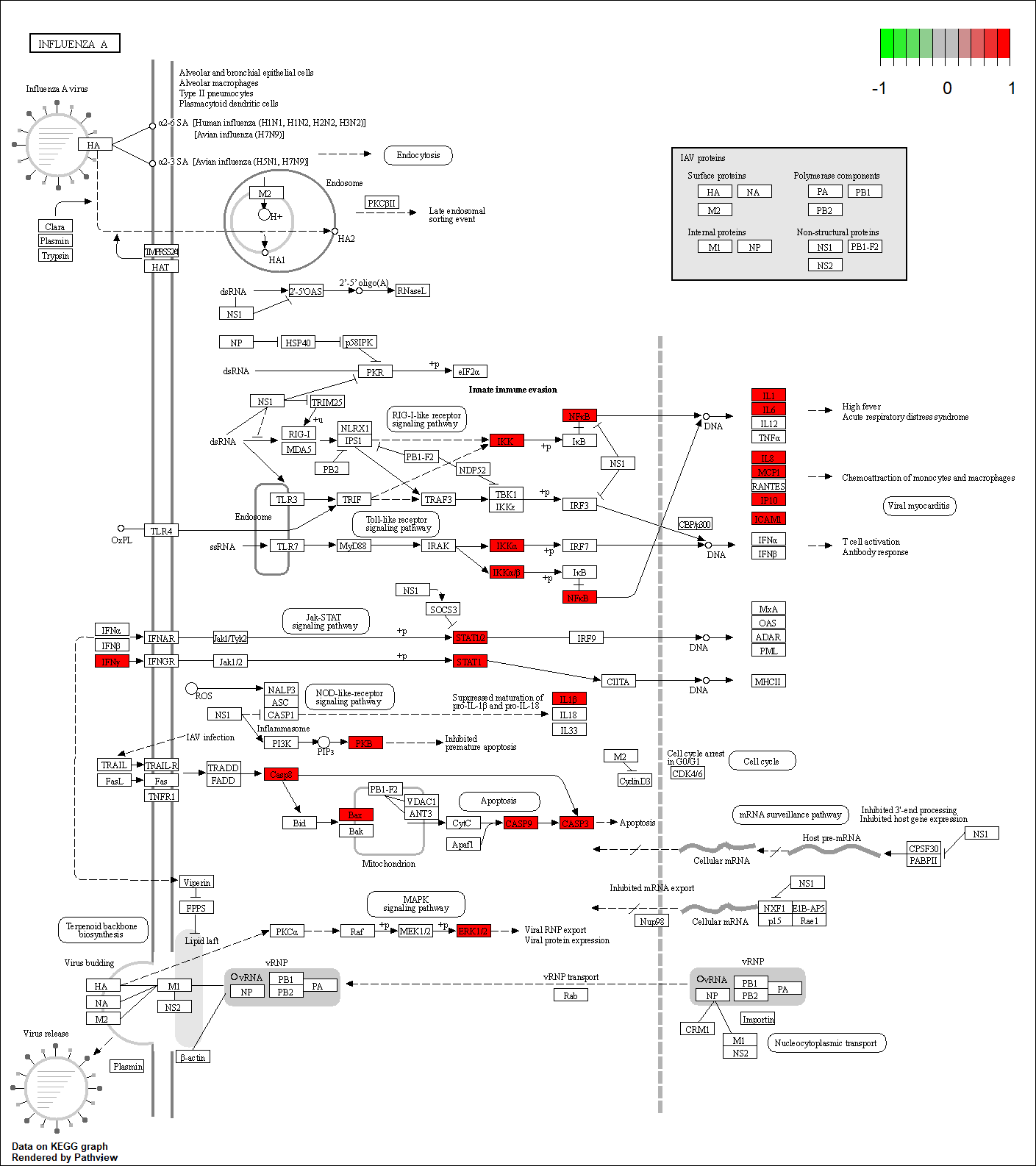

Supplement: S1 Data — (ZIP) [file pone.0274639.s001.zip › minimal data/GO+KEGG/R.KEGG/hsa05164.pathview.png]

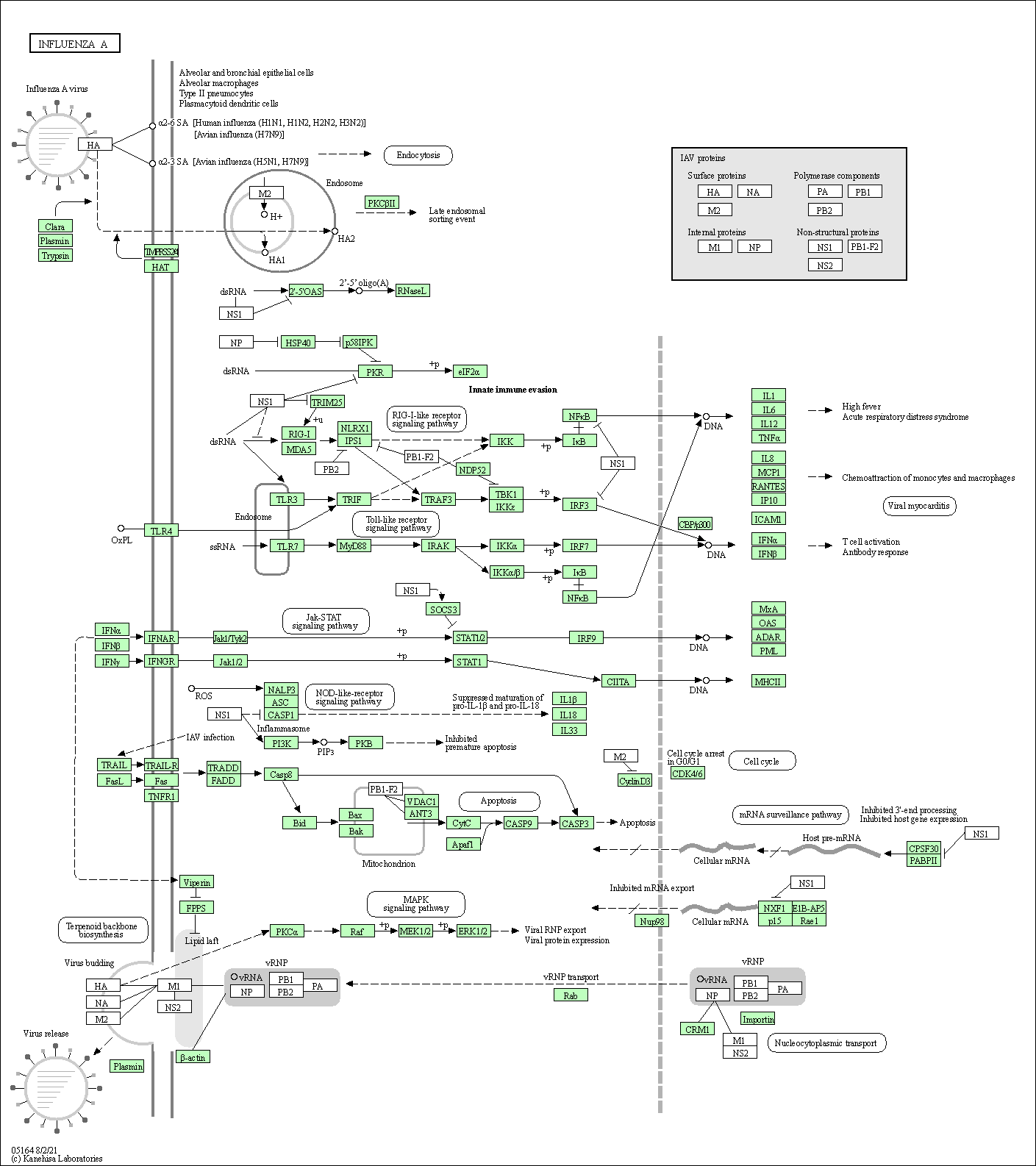

Supplement: S1 Data — (ZIP) [file pone.0274639.s001.zip › minimal data/GO+KEGG/R.KEGG/hsa05164.png]

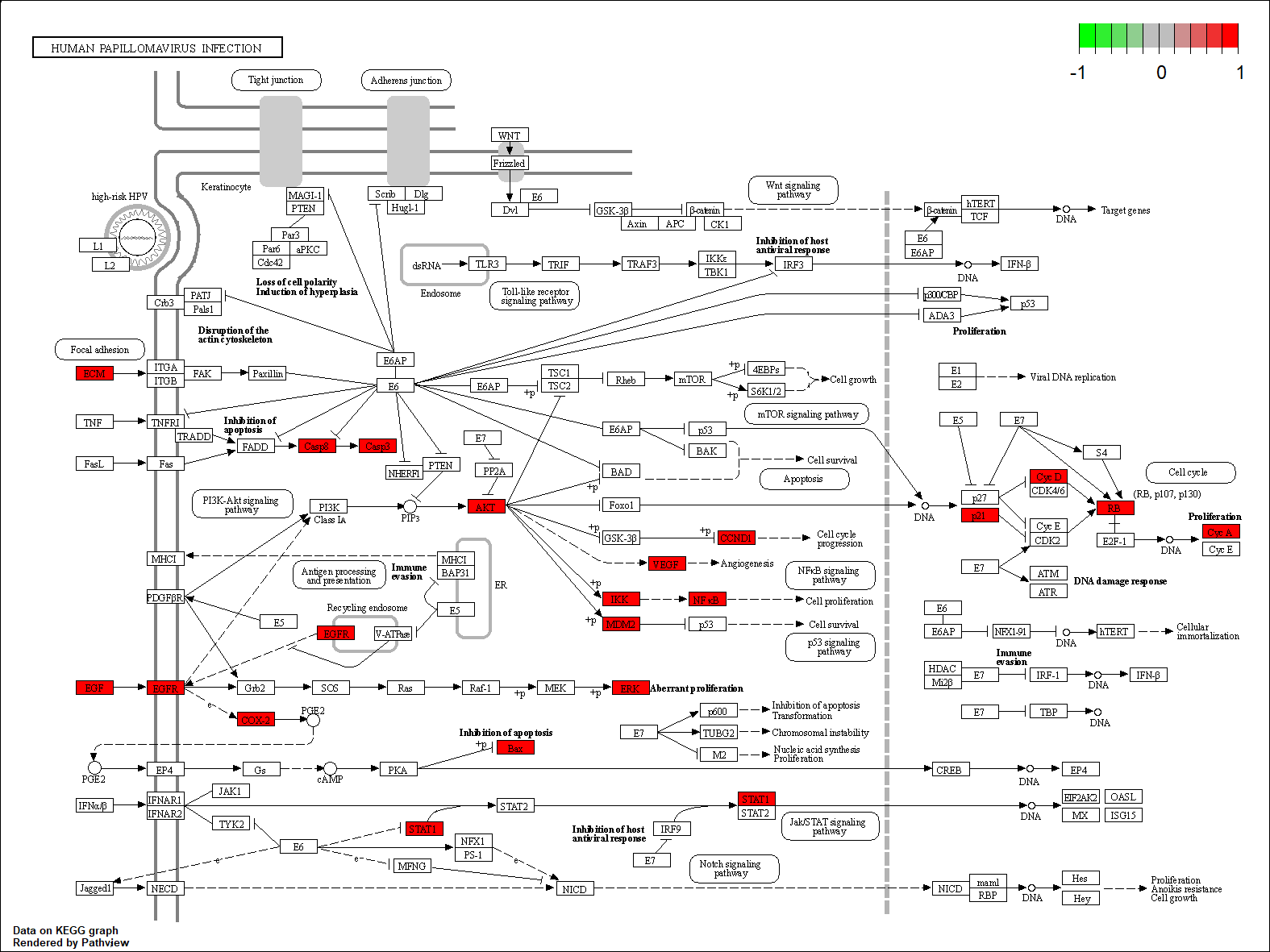

Supplement: S1 Data — (ZIP) [file pone.0274639.s001.zip › minimal data/GO+KEGG/R.KEGG/hsa05165.pathview.png]

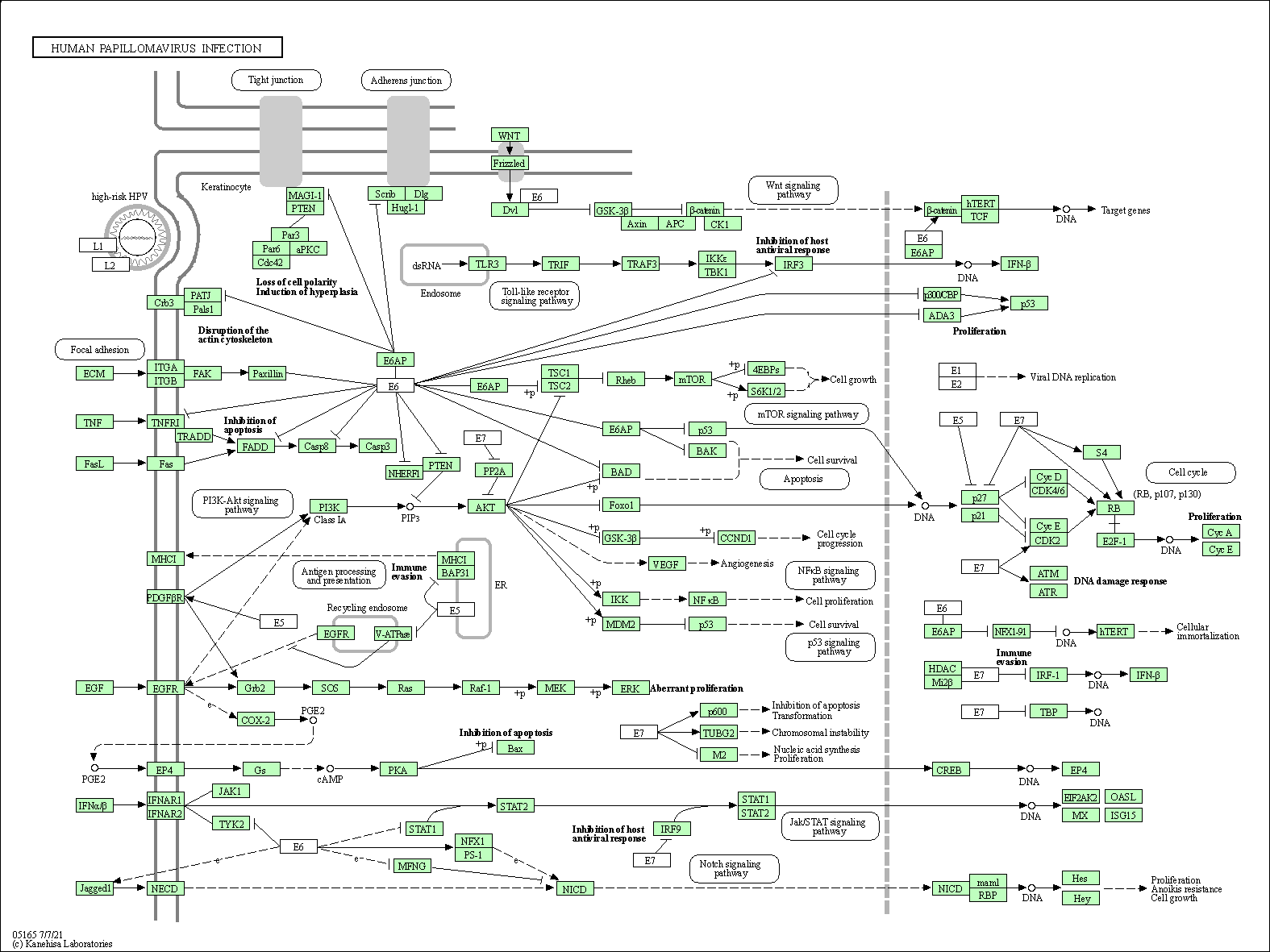

Supplement: S1 Data — (ZIP) [file pone.0274639.s001.zip › minimal data/GO+KEGG/R.KEGG/hsa05165.png]

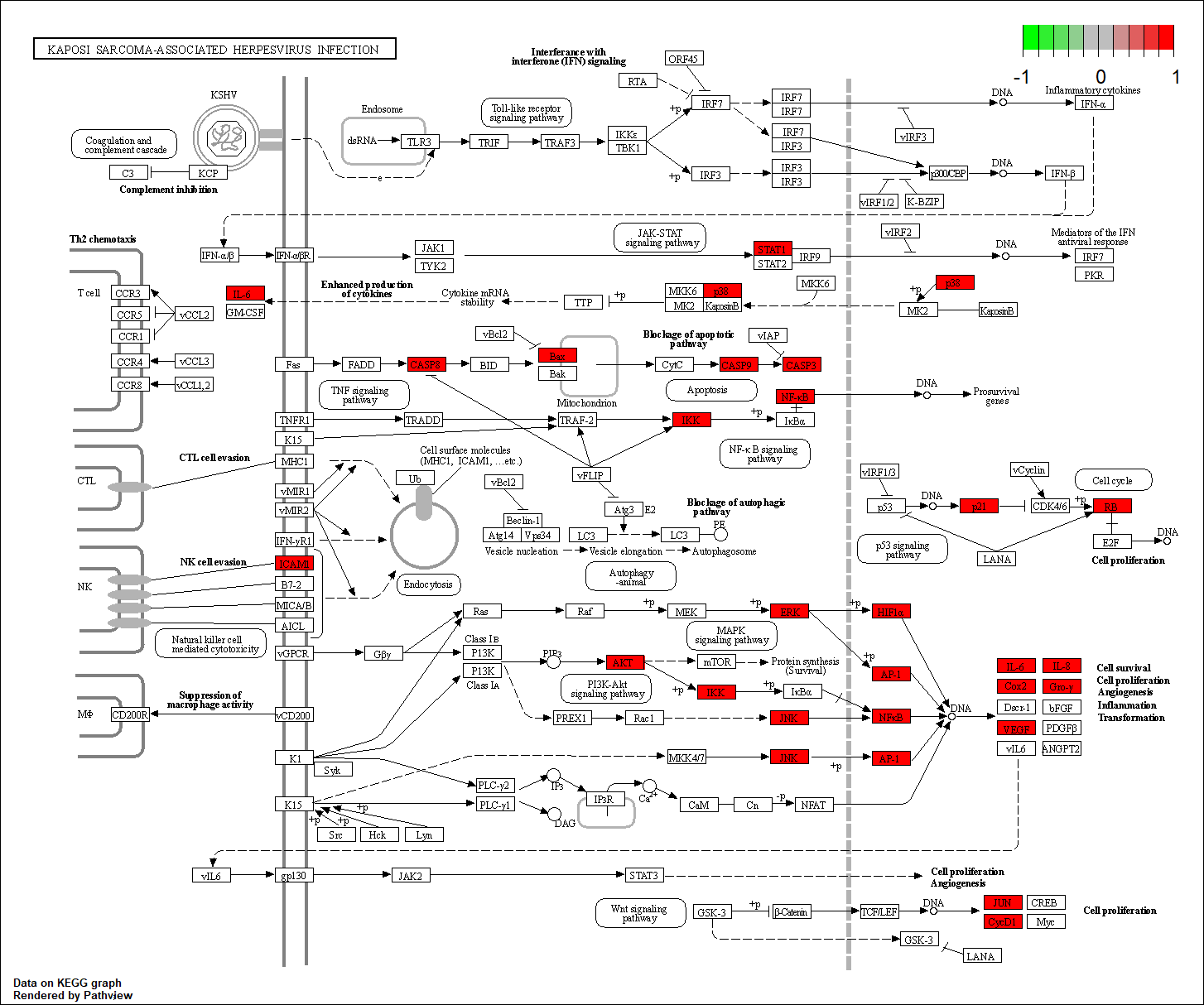

Supplement: S1 Data — (ZIP) [file pone.0274639.s001.zip › minimal data/GO+KEGG/R.KEGG/hsa05167.pathview.png]

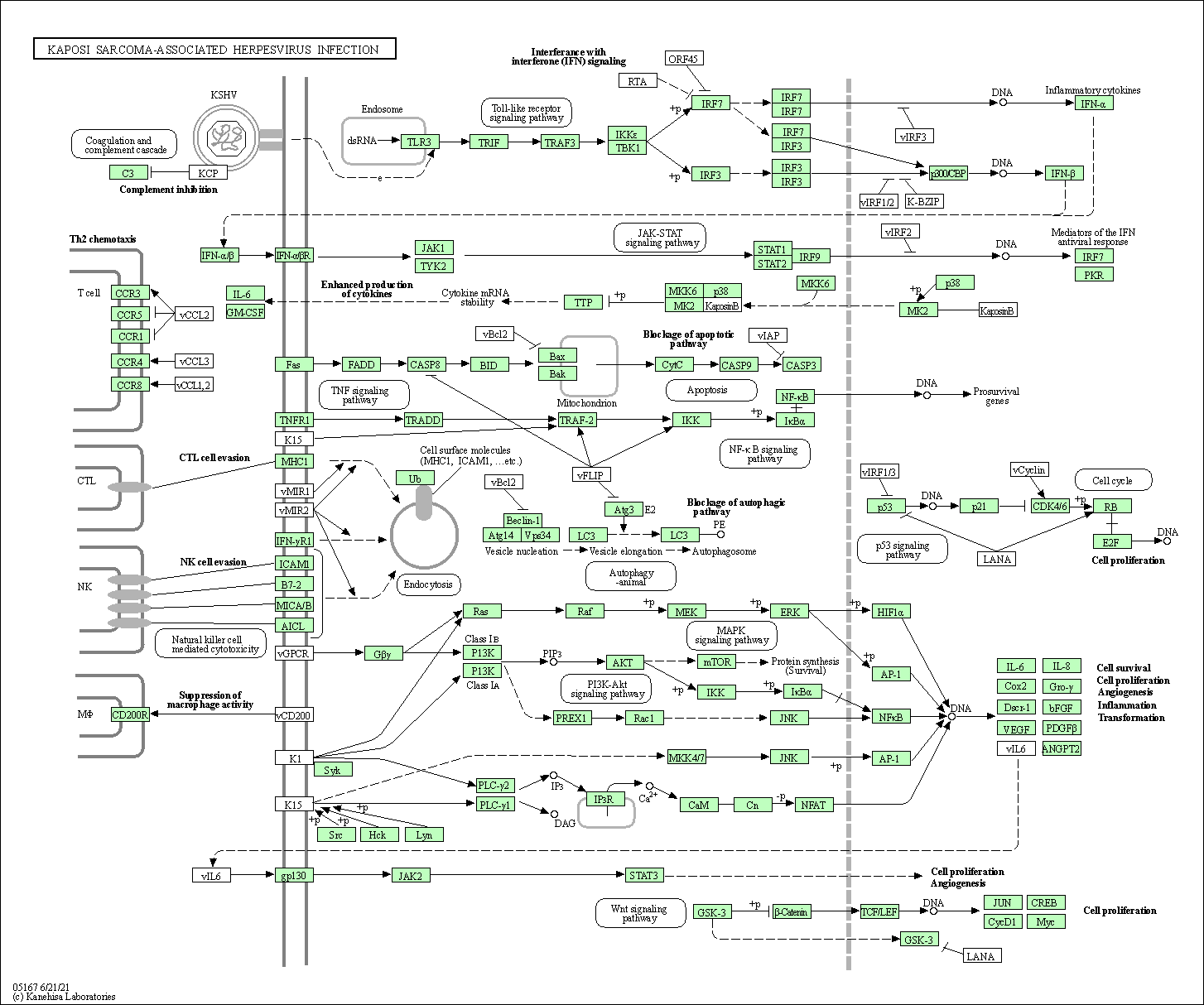

Supplement: S1 Data — (ZIP) [file pone.0274639.s001.zip › minimal data/GO+KEGG/R.KEGG/hsa05167.png]

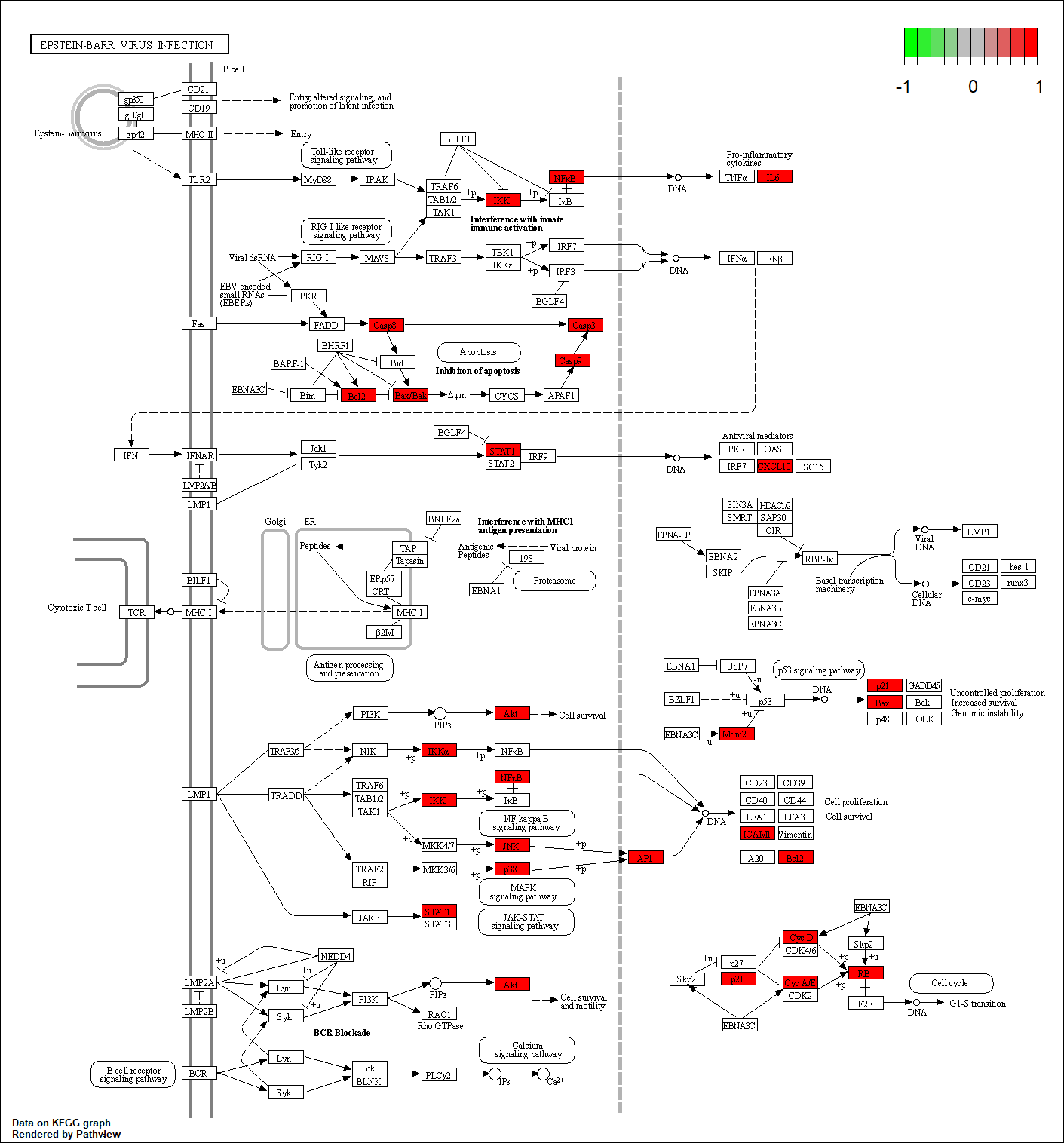

Supplement: S1 Data — (ZIP) [file pone.0274639.s001.zip › minimal data/GO+KEGG/R.KEGG/hsa05169.pathview.png]

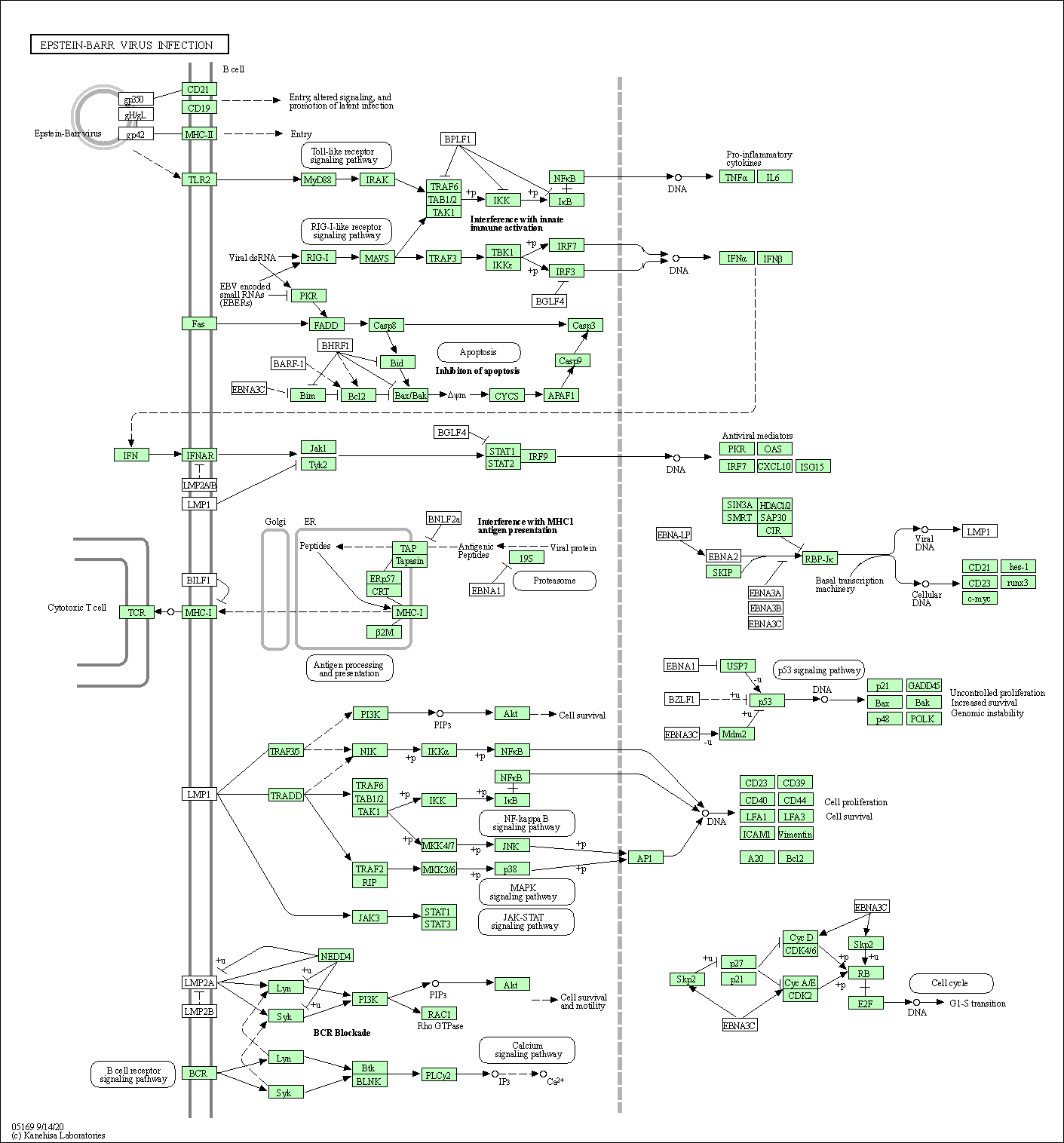

Supplement: S1 Data — (ZIP) [file pone.0274639.s001.zip › minimal data/GO+KEGG/R.KEGG/hsa05169.png]

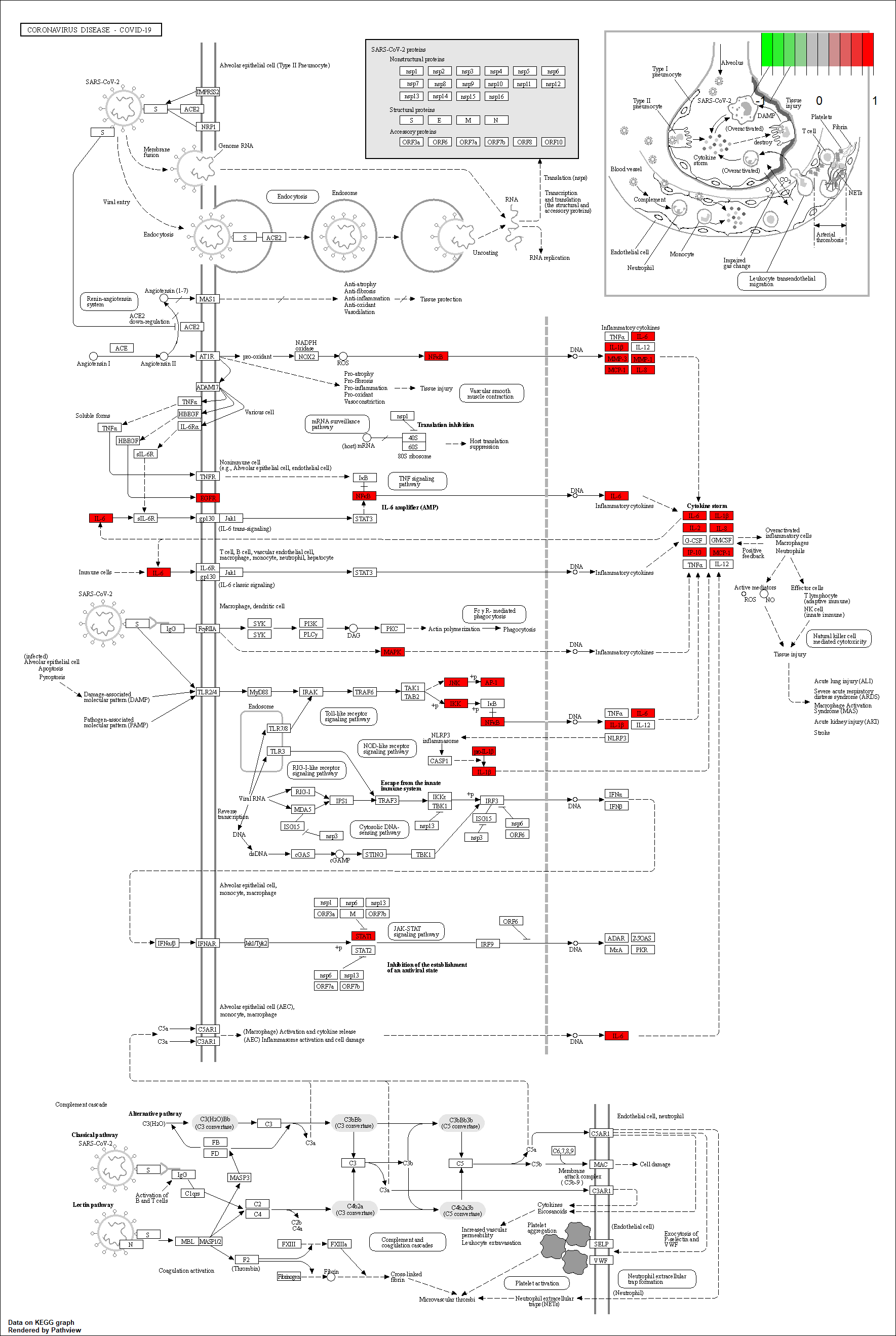

Supplement: S1 Data — (ZIP) [file pone.0274639.s001.zip › minimal data/GO+KEGG/R.KEGG/hsa05171.pathview.png]

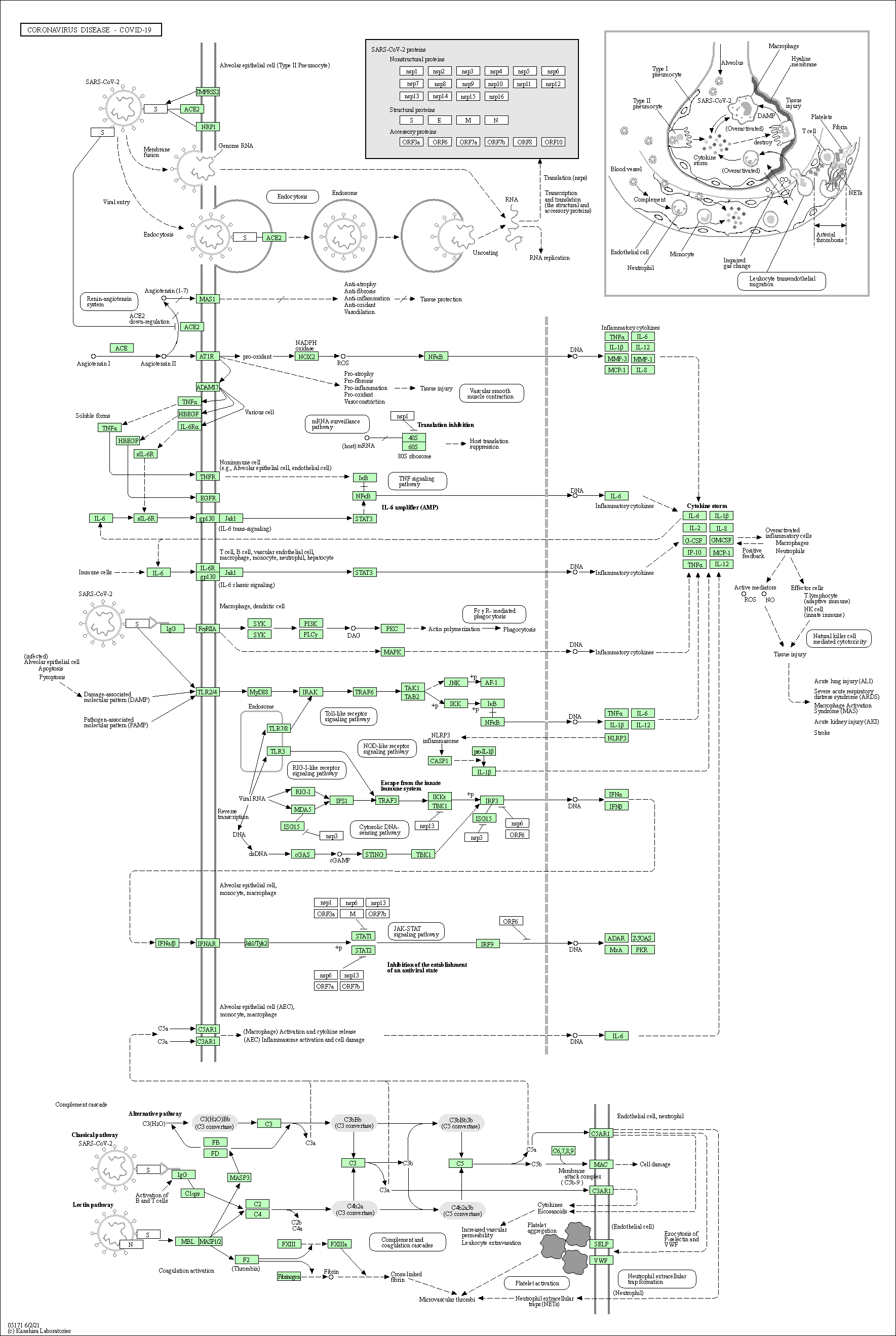

Supplement: S1 Data — (ZIP) [file pone.0274639.s001.zip › minimal data/GO+KEGG/R.KEGG/hsa05171.png]

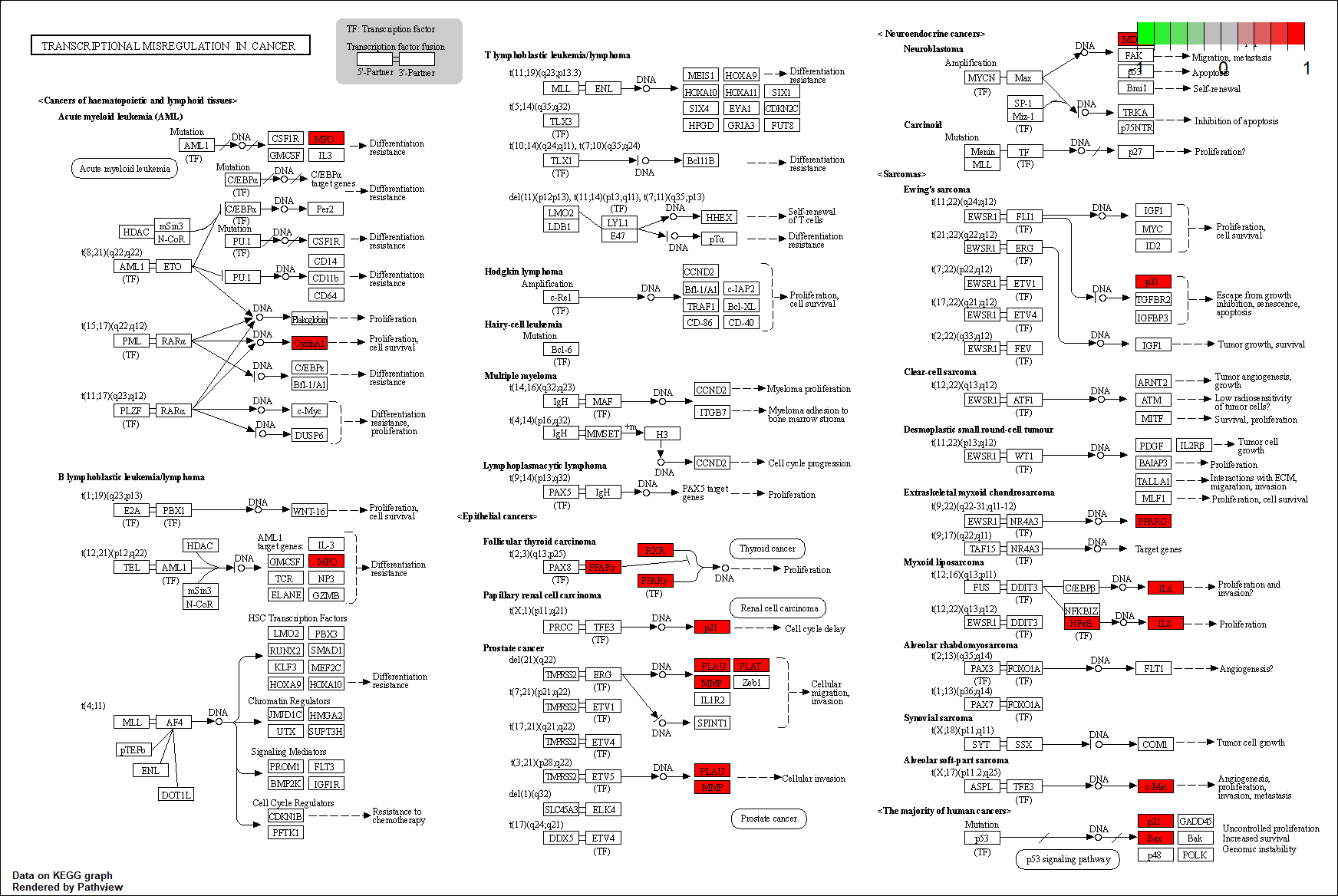

Supplement: S1 Data — (ZIP) [file pone.0274639.s001.zip › minimal data/GO+KEGG/R.KEGG/hsa05202.pathview.png]

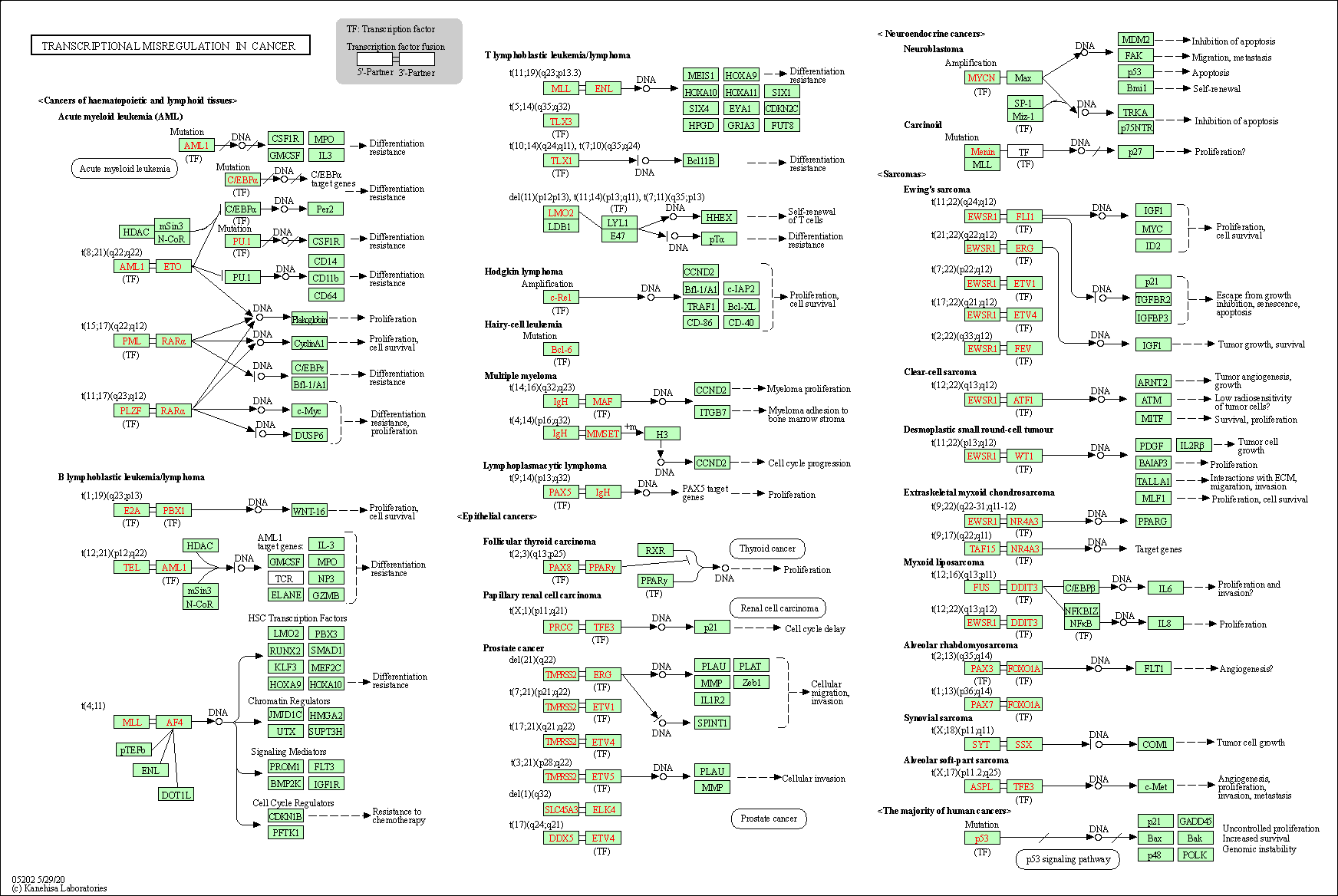

Supplement: S1 Data — (ZIP) [file pone.0274639.s001.zip › minimal data/GO+KEGG/R.KEGG/hsa05202.png]

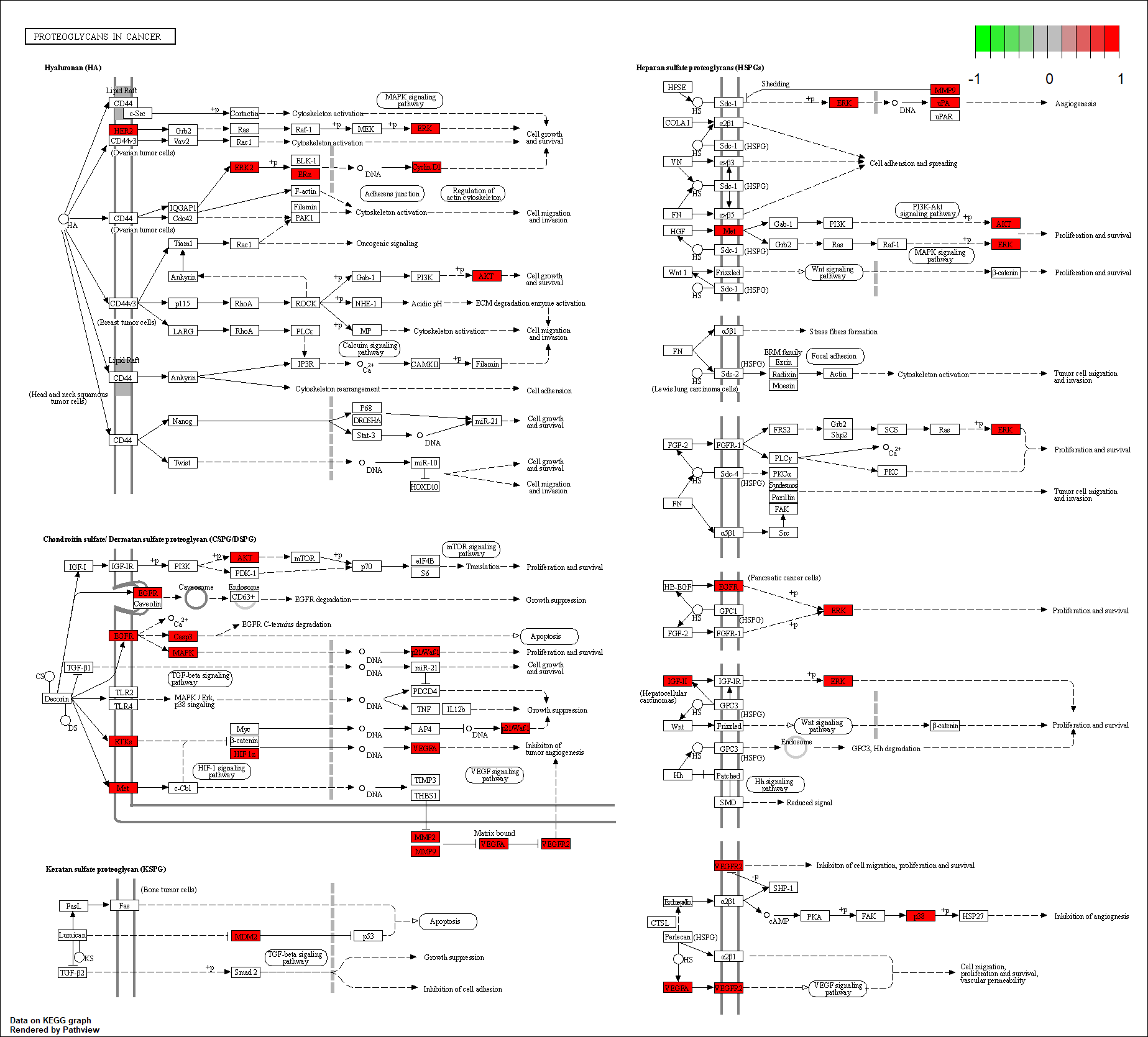

Supplement: S1 Data — (ZIP) [file pone.0274639.s001.zip › minimal data/GO+KEGG/R.KEGG/hsa05205.pathview.png]

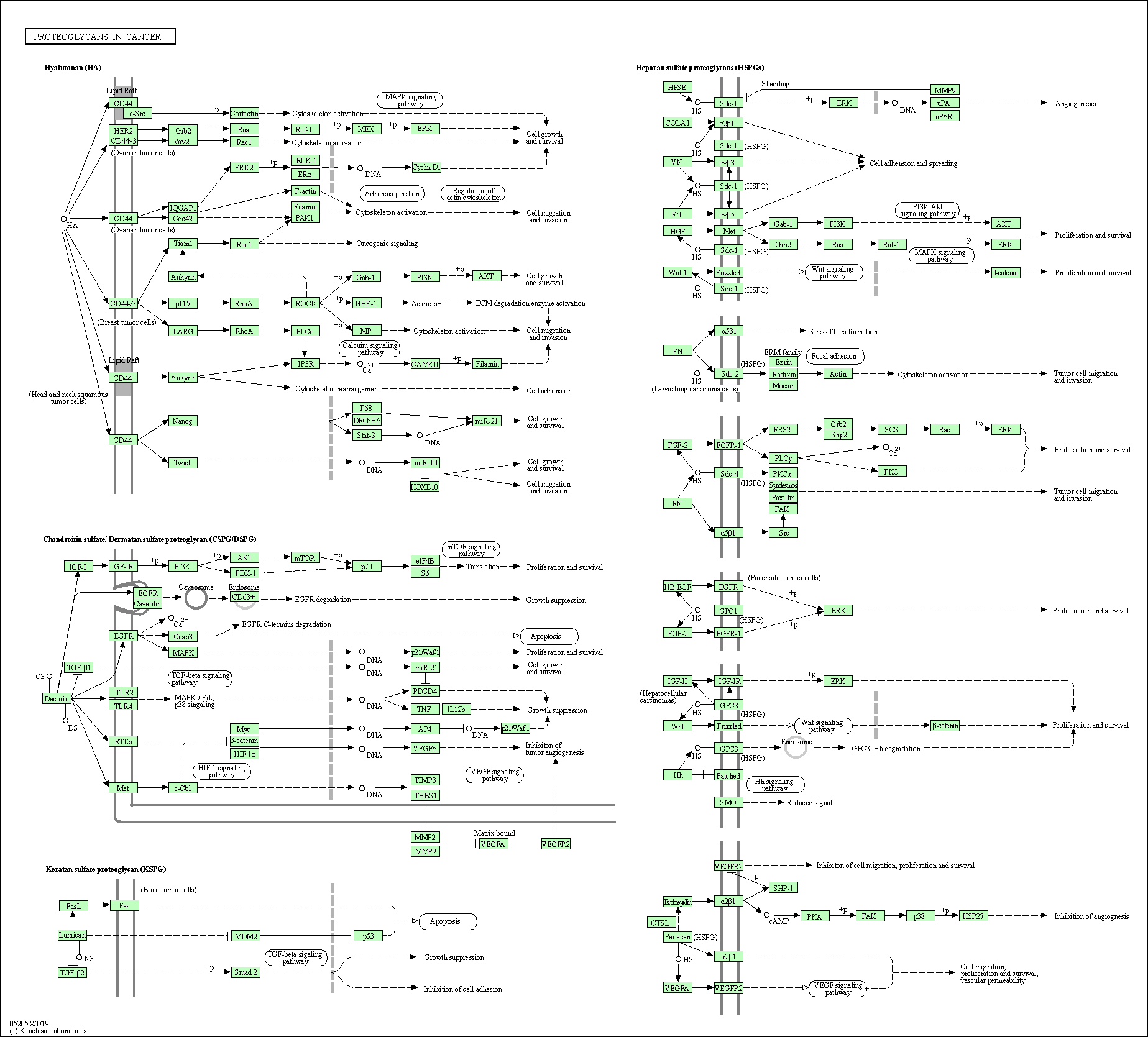

Supplement: S1 Data — (ZIP) [file pone.0274639.s001.zip › minimal data/GO+KEGG/R.KEGG/hsa05205.png]

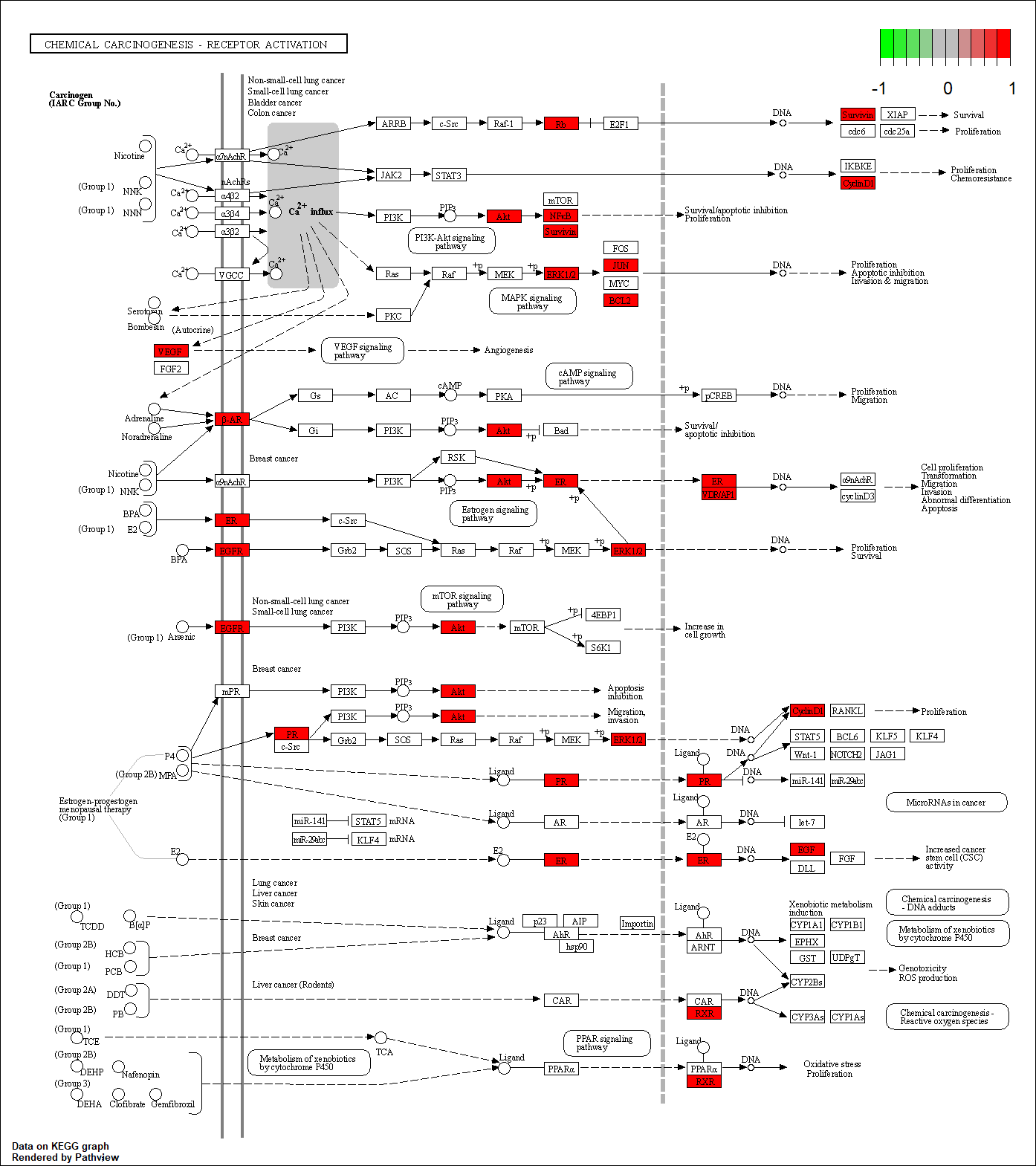

Supplement: S1 Data — (ZIP) [file pone.0274639.s001.zip › minimal data/GO+KEGG/R.KEGG/hsa05207.pathview.png]

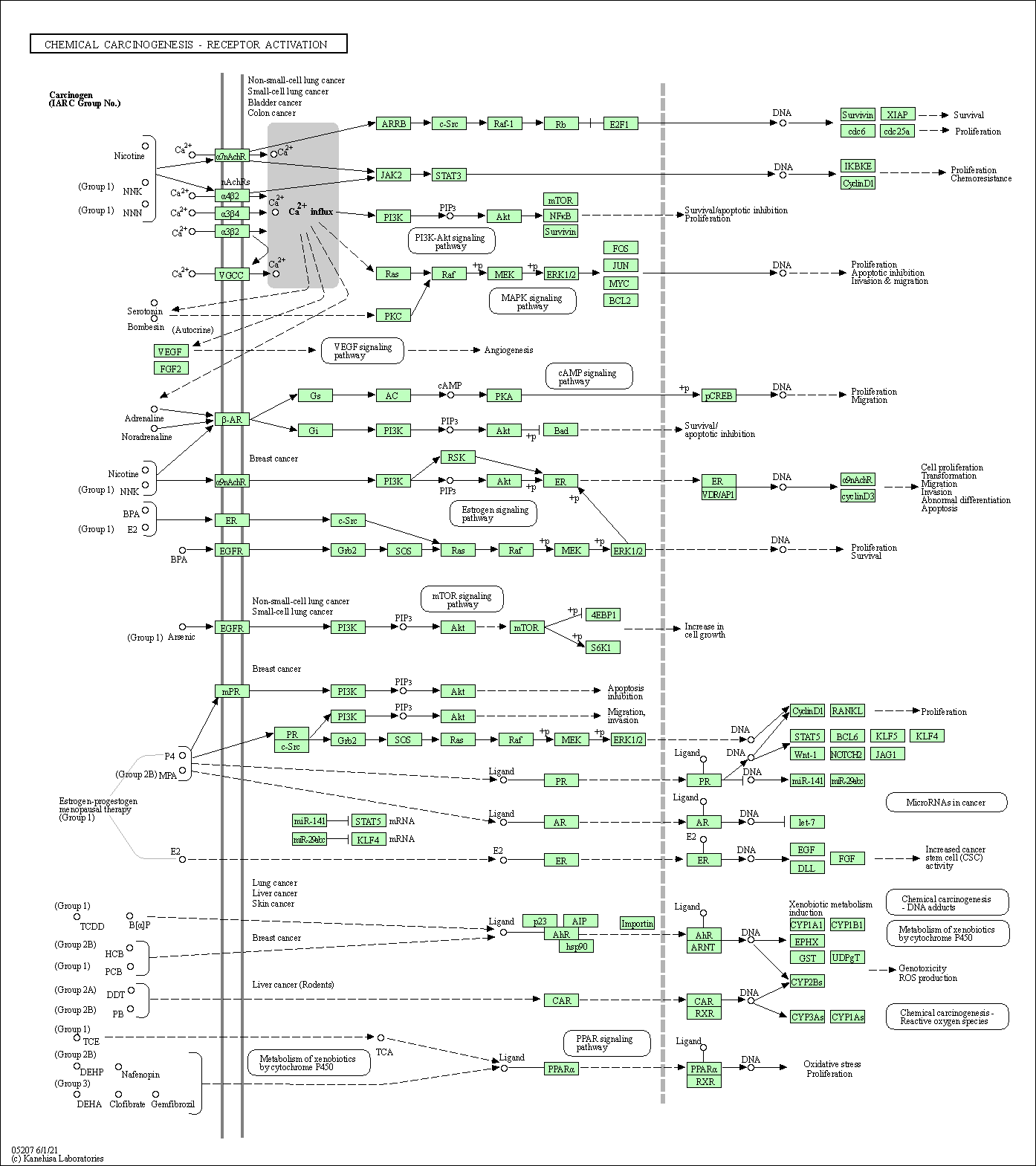

Supplement: S1 Data — (ZIP) [file pone.0274639.s001.zip › minimal data/GO+KEGG/R.KEGG/hsa05207.png]

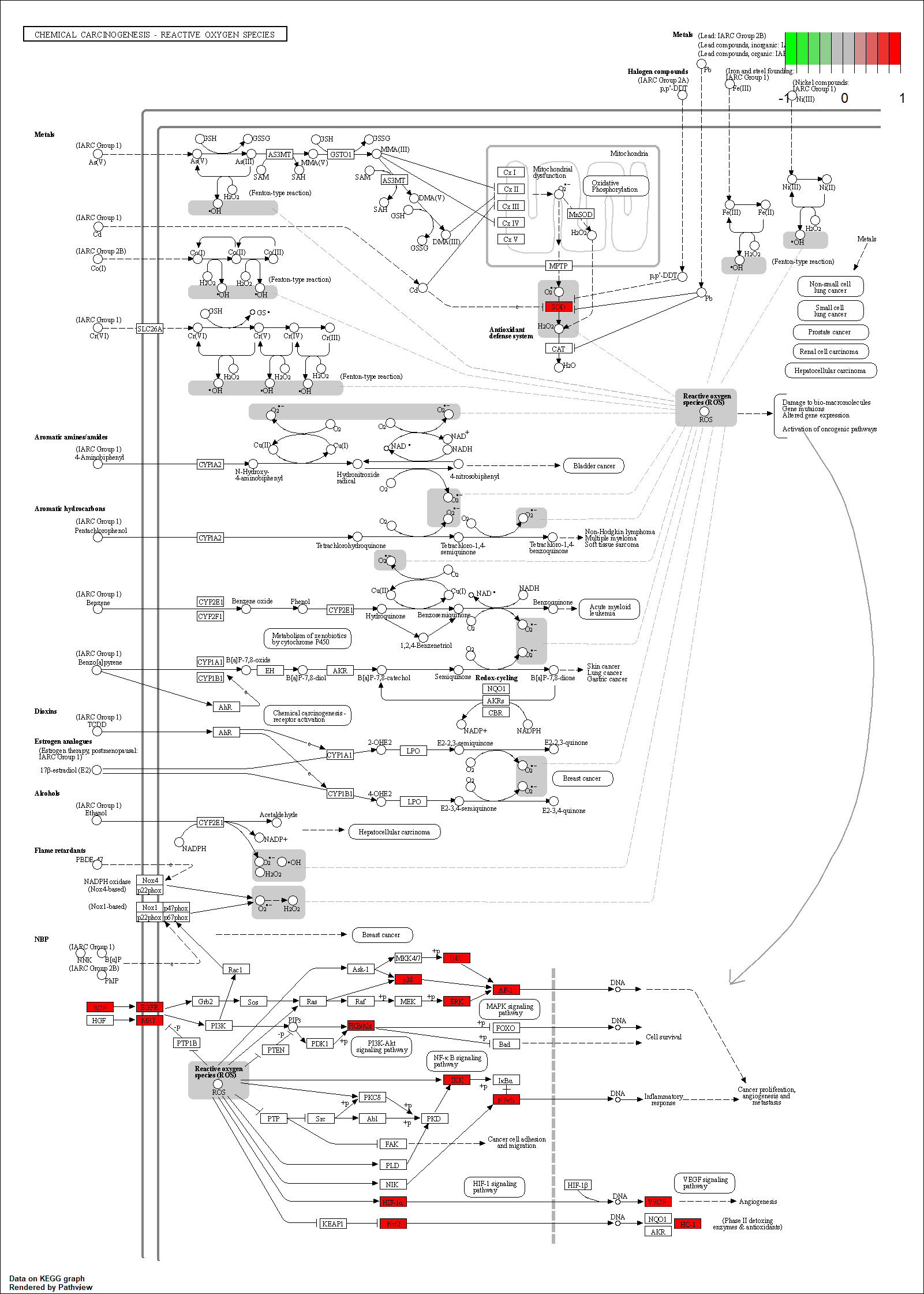

Supplement: S1 Data — (ZIP) [file pone.0274639.s001.zip › minimal data/GO+KEGG/R.KEGG/hsa05208.pathview.png]

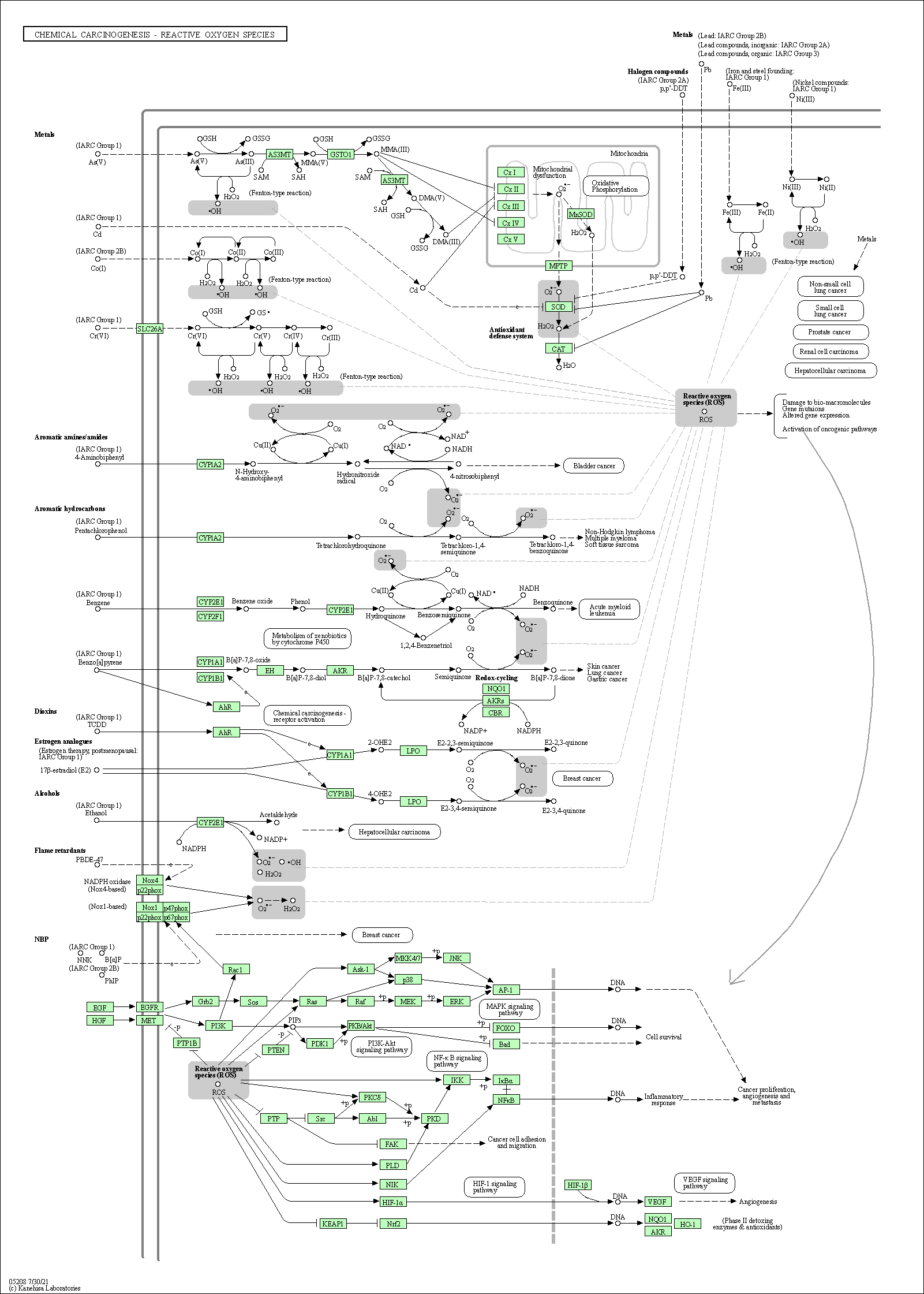

Supplement: S1 Data — (ZIP) [file pone.0274639.s001.zip › minimal data/GO+KEGG/R.KEGG/hsa05208.png]

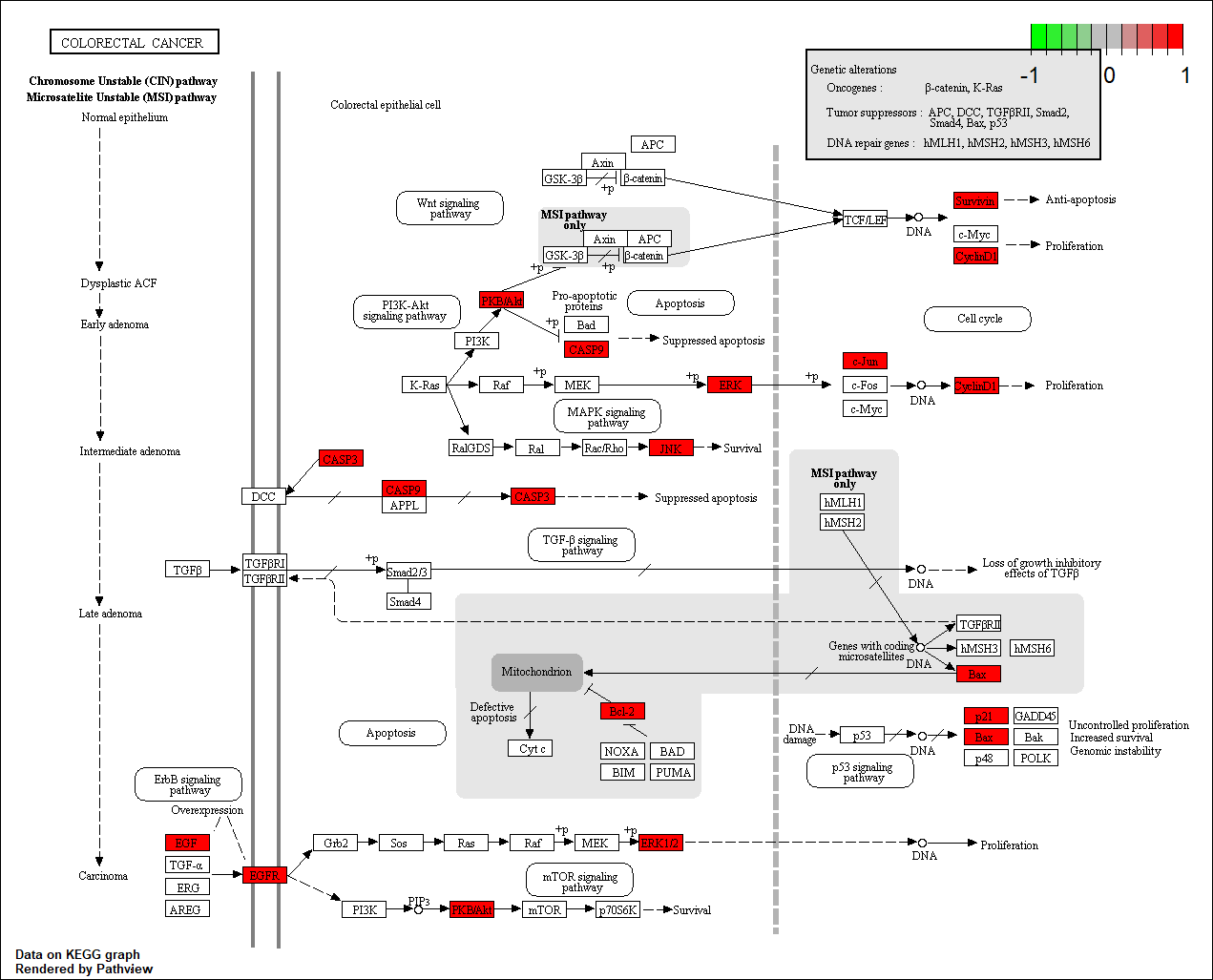

Supplement: S1 Data — (ZIP) [file pone.0274639.s001.zip › minimal data/GO+KEGG/R.KEGG/hsa05210.pathview.png]

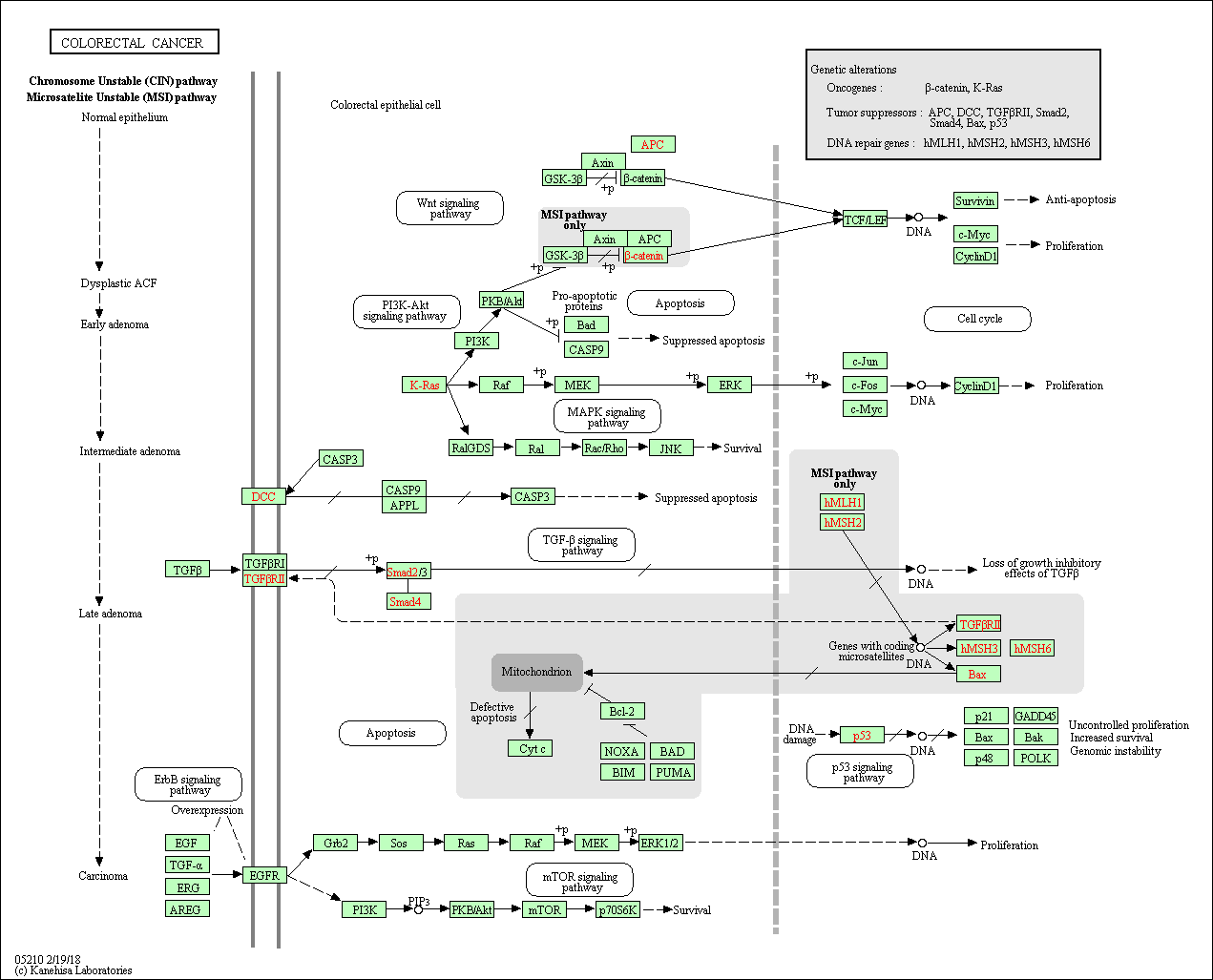

Supplement: S1 Data — (ZIP) [file pone.0274639.s001.zip › minimal data/GO+KEGG/R.KEGG/hsa05210.png]
