# Supplementary material for: Mechanism of gene network in the treatment of intracerebral hemorrhage by natural plant drugs in Lutong granules
Source: PLoS One. 2022 Nov 28;17(11):e0274639. doi: 10.1371/journal.pone.0274639 (PMC9704616; doi:10.1371/journal.pone.0274639)
Supplement: S1 Data — (ZIP) [file pone.0274639.s001.zip › minimal data/Raw data from TTC animal experiments/Statistical data/Percentage of TTC-stained infarct volume.doc]

ONEWAY 梗死体积 BY group
  /POLYNOMIAL=1
  /STATISTICS DESCRIPTIVES HOMOGENEITY
  /PLOT MEANS
  /MISSING ANALYSIS
  /POSTHOC=LSD T2 ALPHA(0.05).


单向


附注	
创建的输出	12-JUL-2022 20:40:56	
注释		
输入	活动的数据集	数据集0	
	过滤器	<none>	
	权重	<none>	
	拆分文件	<none>	
	工作数据文件中的 N 行	9	
缺失值处理	缺失定义	用户定义的缺失值以缺失对待。	
	使用的案例	每个分析的统计量都基于对于该分析中的任意变量都没有缺失数据的案例。	
语法	ONEWAY 梗死体积 BY group
  /POLYNOMIAL=1
  /STATISTICS DESCRIPTIVES HOMOGENEITY
  /PLOT MEANS
  /MISSING ANALYSIS
  /POSTHOC=LSD T2 ALPHA(0.05).	
资源	处理器时间	00:00:00.17	
	已用时间	00:00:00.16	


[数据集0] 


描述	
梗死体积  	
	N	均值	标准差	标准误	均值的 95% 置信区间	极小值	极大值	
					下限	上限			
sham	3	1.1467	.38559	.22262	.1888	2.1045	.82	1.57	
NS	3	7.3867	.90531	.52268	5.1377	9.6356	6.76	8.42	
LT	3	4.1067	.30065	.17358	3.3598	4.8535	3.76	4.30	
总数	9	4.2133	2.75170	.91723	2.0982	6.3285	.82	8.42	


方差齐性检验	
梗死体积  	
Levene 统计量	df1	df2	显著性	
4.068	2	6	.076	


单因素方差分析	
梗死体积  	
	平方和	df	均方	F	显著性	
组间	（组合）	58.458	2	29.229	82.828	.000	
	线性项	对比	13.142	1	13.142	37.243	.001	
		偏差	45.315	1	45.315	128.413	.000	
组内	2.117	6	.353			
总数	60.575	8				


在此之后检验


多重比较	
因变量:   梗死体积  	
	(I) group	(J) group	均值差 (I-J)	标准误	显著性	95% 置信区间	
						下限	上限	
LSD	sham	NS	-6.24000^*^	.48503	.000	-7.4268	-5.0532	
		LT	-2.96000^*^	.48503	.001	-4.1468	-1.7732	
	NS	sham	6.24000^*^	.48503	.000	5.0532	7.4268	
		LT	3.28000^*^	.48503	.001	2.0932	4.4668	
	LT	sham	2.96000^*^	.48503	.001	1.7732	4.1468	
		NS	-3.28000^*^	.48503	.001	-4.4668	-2.0932	
Tamhane	sham	NS	-6.24000^*^	.56812	.008	-9.2536	-3.2264	
		LT	-2.96000^*^	.28229	.002	-4.1119	-1.8081	
	NS	sham	6.24000^*^	.56812	.008	3.2264	9.2536	
		LT	3.28000^*^	.55075	.049	.0262	6.5338	
	LT	sham	2.96000^*^	.28229	.002	1.8081	4.1119	
		NS	-3.28000^*^	.55075	.049	-6.5338	-.0262	

*. 均值差的显著性水平为 0.05。	


均值图
