# Supplementary material for: Mechanism of gene network in the treatment of intracerebral hemorrhage by natural plant drugs in Lutong granules
Source: PLoS One. 2022 Nov 28;17(11):e0274639. doi: 10.1371/journal.pone.0274639 (PMC9704616; doi:10.1371/journal.pone.0274639)
Supplement: Attachment — Submitted filename: Comments on Manuscript Number PONE-D-22-12747.docx [file pone.0274639.s002.docx]

**Comments on PONE-D-22-12747**

The research aimed to evaluate the effects of six natural plant drugs in LT on ICH and the relevant mechanisms using bioinformatics and network pharmacology methods. Results showed that LT-6 are beneficial to ICH by participating in the post-ICH process through anti-inflammatory response, nerve repair, analgesia, anti-epilepsy and other aspects. The structure of the paper was well organized, the methods were described in detail and the results were convinced.

Although the paper is in a good quality, there are still some problems should be revised before the acceptance.

1. English language should be revised by native speaker.

2. Please add a space between number and unit. For example, 150 g instead of 150g(Line 157)

3. Line 160: change 100 degrees Celsius to 100 °C

4. Line 165: what is “they” indicates?

5. Line 193: No full name of ICH is needed.

6. What is the meaning of the words in red in Table 2?

7. Line 393: the full name of ICH has been shown at the very beginning of the paper, so no full name is needed here. Please check it through the whole paper.

8. All the figures are not in a high quality, please re-upload them.
