# Supplementary material for: Mechanism of gene network in the treatment of intracerebral hemorrhage by natural plant drugs in Lutong granules
Source: PLoS One. 2022 Nov 28;17(11):e0274639. doi: 10.1371/journal.pone.0274639 (PMC9704616; doi:10.1371/journal.pone.0274639)
Supplement: Attachment — Submitted filename: Response to Reviewers.docx [file pone.0274639.s003.docx]

**Response to Reviewers**

Journal Requirements:

1. The style of the paper has been modified according to the PLOS ONE style template.
2. An updated conflict of interest statement has been included with the cover letter.
3. The minimal data has been uploaded as an attachment.
4. ORCID iD. we have provided an ORCID and associated.
5. After checking, all figures and tables are mentioned in the text.

Review Comments to the Author

Reviewer #1:

1. English language should be revised by native speaker.

Answer: Full text has been retouched.

2. Please add a space between number and unit. For example, 150 g instead of 150g(Line 157).

Answer: Already revised(Line 151-152).

3. Line 160: change 100 degrees Celsius to 100 °C.

Answer: Already revised(Line 154).

4. Line 165: what is “they” indicates?

Answer: "They" means SD rats, already revised. (Line 159).

5. Line 193: No full name of ICH is needed.

Answer: Already revised. (Line 195).

6. What is the meaning of the words in red in Table 2?

Answer: List of genes in the top 10 KEGG pathways, where the red genes represent the distribution of the 6 HUB genes in each pathway. (Line238) .

7. Line 393: the full name of ICH has been shown at the very beginning of the paper, so no full name is needed here. Please check it through the whole paper.

Answer: Already revised.

8. All the figures are not in a high quality, please re-upload them.

Answer: We have re-uploaded.

Reviewer #2

This is a very complete research paper about the mechanism of gene network in the treatment of intracerebral hemorrhage by natural plant drugs in Lutong granules. However, authors still need to discuss more about the relationship between Results of Animal experiment and gene network.

Answer: We have further discussed the relationship between the results of the animal experiments and the gene network. (lines 438-457).

Reviewer #3:

In Line 153-162, the extract yield of Lutong granules is necessary to be shown.

Answer: Already added, The extract yield of Lutong granules is 25%. (Line 156).
2. Please combine pictures that illustrate the same issue into one figure. For example, Figure 2 and Figure 3 are suggested to be considered as two panels in one figure. Similarly, Fig 4-5, Fig 6-7, and Fig 8-9.

Answer: Images have been merged.

3. Signal pathway and biological progress are at different levels in the disease model. Therefore, It is recommended to rewrite the result with a more logical organization. (part “2.4 Gene ontology (GO) and KEGG signaling pathway analysis”).

Answer: Part 2.4 has been rewritten. (Line 206-229).
4. Please provide the representative pictures of the TTC stain in Fig 10.

Answer: Representative images of TTC staining have been provided(Line 290-296).
